# The Highly Potent AhR Agonist Picoberin Modulates Hh-Dependent Osteoblast Differentiation

**DOI:** 10.1021/acs.jmedchem.2c00956

**Published:** 2022-12-02

**Authors:** Jana Flegel, Saad Shaaban, Zhi Jun Jia, Britta Schulte, Yilong Lian, Adrian Krzyzanowski, Malte Metz, Tabea Schneidewind, Fabian Wesseler, Anke Flegel, Alisa Reich, Alexandra Brause, Gang Xue, Minghao Zhang, Lara Dötsch, Isabelle D. Stender, Jan-Erik Hoffmann, Rebecca Scheel, Petra Janning, Fraydoon Rastinejad, Dennis Schade, Carsten Strohmann, Andrey P. Antonchick, Sonja Sievers, Pedro Moura-Alves, Slava Ziegler, Herbert Waldmann

**Affiliations:** † Department of Chemical Biology, Max Planck Institute of Molecular Physiology, Dortmund 44227, Germany; ‡ Faculty of Chemistry, Chemical Biology, Technical University Dortmund, Dortmund 44227, Germany; § Faculty of Chemistry, Inorganic Chemistry, Technical University Dortmund, Dortmund 44227, Germany; ∥ Ludwig Institute for Cancer Research, Nuffield Department of Clinical Medicine, University of Oxford, Oxford OX3 7DQ, United Kingdom; ⊥ i3S-Instituto de Investigação e Inovação em Saúde, Universidade do Porto, 4200-135 Porto, Portugal; # IBMC-Instituto de Biologia Molecular e Celular, Universidade do Porto, 4200-135 Porto, Portugal; ∇ Compound Management and Screening Center, Dortmund 44227, Germany; ○ Nuffield Department of Medicine, Target Discovery Institute, University of Oxford, Oxford, OX3 7FZ, UK; ◆ Protein Chemistry Facility, Max Planck Institute of Molecular Physiology, Dortmund 44227, Germany; ¶ Dept. of Pharmaceutical & Medicinal Chemistry, Institute of Pharmacy, Christian-Albrechts-University of Kiel, Kiel 24118, Germany; & Faculty of Chemistry, Institute of Organic Chemistry, University of Vienna Währinger Str. 38, Vienna 1090, Austria; ● Key Laboratory of Birth Defects and Related Diseases of Women and Children, Evidence-Based Pharmacy Center, West China Second University Hospital, Sichuan University, Chengdu 610041, China; ◊ Department of Chemistry and Forensics, School of Science and Technology, Nottingham Trent University, Clifton Lane, Nottingham, NG11 8NS, United Kingdom

## Abstract

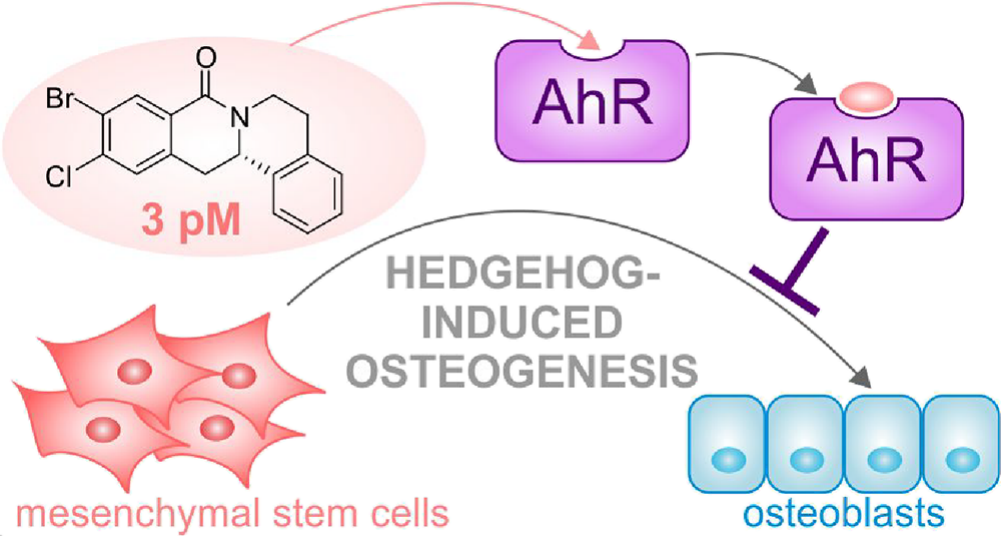

Identification and analysis of small molecule bioactivity in target-agnostic cellular assays and monitoring changes in phenotype followed by identification of the biological target are a powerful approach for the identification of novel bioactive chemical matter in particular when the monitored phenotype is disease-related and physiologically relevant. Profiling methods that enable the unbiased analysis of compound-perturbed states can suggest mechanisms of action or even targets for bioactive small molecules and may yield novel insights into biology. Here we report the enantioselective synthesis of natural-product-inspired 8-oxotetrahydroprotoberberines and the identification of Picoberin, a low picomolar inhibitor of Hedgehog (Hh)-induced osteoblast differentiation. Global transcriptome and proteome profiling revealed the aryl hydrocarbon receptor (AhR) as the molecular target of this compound and identified a cross talk between Hh and AhR signaling during osteoblast differentiation.

## Introduction

The discovery of novel, bioactive compounds and the identification of their molecular targets and mode of action are at the heart of Chemical Biology.^1,2^ Unbiased monitoring of phenotypic changes can lead to the identification of novel targets and biological networks.^3^ In the design of biologically relevant phenotypic assays, the use of a disease-relevant cell model, application of an appropriate stimulus, and monitoring of a disease marker or functional trait (also known as ″the rule of 3″) are crucial.^4^ To understand the underlying mode of action of bioactive small molecules and to link a molecular target to impaired biological networks,^2^ application of profiling approaches, like transcriptomics and proteomics, provides a more holistic view on bioactivity and may suggest a mechanism of action or even a target.^5^

Here we describe the design, synthesis, and biological characterization of natural-product-inspired 8-oxotetrahydroprotoberberines. A catalytic enantioselective coupling and cyclization sequence employing the chiral Rh*Jas*Cp catalyst enabled the efficient synthesis of a focused compound collection based on this tetracyclic scaffold. Biological investigation revealed 8-oxotetrahydroprotoberberines as novel inhibitors of Hh-induced osteoblast differentiation with activity in the picomolar range. Transcriptome and proteome profiling of the most potent inhibitor, termed Picoberin, identified the aryl hydrocarbon receptor (AhR) as the molecular target and suggested a role of AhR during Hh-induced osteogenesis and cross talk between AhR- and Hh signaling.

## Results and Discussion

**Figure 1:**
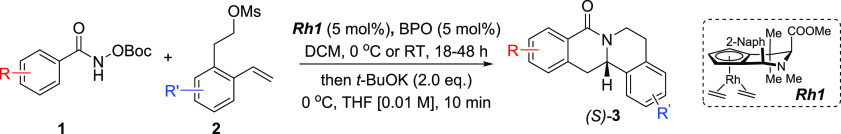
Enantioselective RhJasCp-catalyzed synthesis of 8-oxotetrahydroprotoberberines.

Natural products with 8-oxotetrahydroprotoberberine scaffold^6−8^ are endowed with antibacterial activity and cytotoxicity to specific cancer cell lines.^9,10^ Only a few methods have been developed for the asymmetric synthesis of the 8-oxotetrahydroprotoberberine scaffold,^11^ and they rely on the use of chiral auxiliaries or chiral reagents in stoichiometric amounts.^12−14^ Transition-metal catalyzed C–H functionalization and C–C bond formation may enable efficient syntheses of NP scaffolds,^15^ and to this end, chiral cyclopentadienyl (Cp^x^) complexes have proven to be powerful catalysts.^16−18^ We envisioned that application of the Rh*Jas*Cp catalysts^19,20^ to stereoselectively steer the reaction between *O*-protected hydroxamates with styrenes to yield 3,4-dihydroisoquinolinones^21,22^ might be applied for the development of a catalytic enantioselective synthesis of 8-oxotetrahydroprotoberberines. To this end, we investigated Rh*Jas*Cp-catalyzed coupling of *OBoc-*hyroxamates **1** and *ortho*-substituted styrenes **2**(23) followed by an *in situ* cyclization step (Figure 1). Screening of different catalysts and reaction conditions showed that catalyst **Rh1** gave the best results, and the desired cyclization products **3** were obtained with excellent yields and ee values under mild conditions (Figure 1). The introduction of substituents with different steric or electronic demands on the *OBoc*-hydroxamates **1** and on the styrene part **2** was well tolerated, and the highly enantioenriched products **3a**–**3u** were obtained with good yields and excellent enantioselectivity. The absolute configuration of the 8-oxotetrahydroprotoberberines was determined for **3l** by means of crystal structure analysis (Figure S1 and Table 1). Notably, analogues in which the piperidine ring is replaced by a smaller five-membered pyrrolidine ring (→ **4**) or a larger seven-membered ring (→ **5**) can also be obtained with this method with excellent enantioselectivity (see Table 2, entries 28 and 29).

**Table 1:** Crystallographic Data of Compound **3l**

compound	**3l**
empirical formula	C_17_H_13_BrClNO
formula weight	362.64
temperature/K	100.0
crystal system	orthorhombic
space group	*P*2_1_2_1_2_1_
*a*/Å	7.2772(3)
*b*/Å	14.0454(4)
*c*/Å	14.2457(6)
α/°	90
β/°	90
γ/°	90
volume/Å^3^	1456.07(10)
*Z*	4
ρ_calc_g/cm^3^	1.654
μ/mm^–1^	5.509
*F*(000)	728.0
crystal size/mm^3^	0.494 × 0.068 × 0.042
radiation	CuKα (λ = 1.54178)
2Θ range for data collection/°	8.842 to 154.866
index ranges	–8 ≤ h ≤ 9, –17 ≤ k ≤ 17, –18 ≤ l ≤ 17
reflections collected	20,326
independent reflections	3079 [*R*_int_ = 0.0348, *R*_sigma_ = 0.0234]
data/restraints/parameters	3079/0/190
goodness-of-fit on *F*^2^	1.072
final *R* indexes [*I* ≥ 2σ(*I*)]	*R*_1_ = 0.0218, w*R*_2_ = 0.0543
final *R* indexes [all data]	*R*_1_ = 0.0219, *wR*_2_ = 0.0544
largest diff. peak/hole/e Å^–3^	0.70/–0.29
Flack parameter	–0.003(6)

**Table 2:**
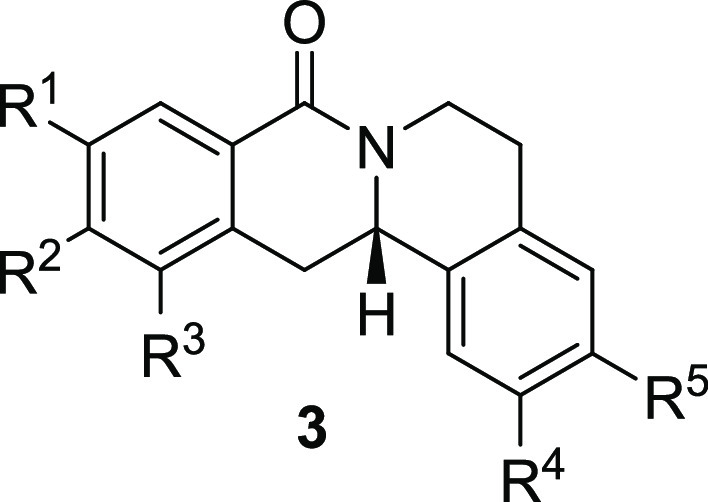
Structure–Activity Relationship (SAR) of 8-Oxotetrahydroprotoberberines (**3**)^a^

entry	derivative	R^1^	R^2^	R^3^	R^4^	R^5^	stereocenter	yield %	ee %	% osteogenesis at 10 μM	inhibition of osteogenesis IC_50_ (nM) ± SE
**1**	**3a**	H	H	H	H	H	S	76	97	100	
**2**	**3b**	H	*i*Pr	H	H	H	S	61	95	90.1	
**3**	**3c**	H	Me	H	H	H	S	84	91	93.2	
**4**	**3d**	H	OMe	H	H	H	S	61	97	114	
**5**	**3e**	H	F	H	H	H	S	45	97	61.2	
**6**	**3f**	H	Cl	H	H	H	S	45	97	30.4	1.2 ± 0.4
**7**	**3g**	H	Br	H	H	H	S	53	97	44.2	4.2 ± 1.6
**8**	**3h**	H	I	H	H	H	S	49	98	57.8	
**9**	**3i**	H	CF_3_	H	H	H	S	61	96	33.1	0.7 ± 0.2
**10**	**3j**	H	H	Cl	H	H	S	64	91	53.0	
**11**	**3k**	Cl	H	Cl	H	H	S	55	90	53.2	
**12**	**3l**	Br	Cl	H	H	H	S	11	96	29.1	0.003 ± 0.001
**13**	**3m**	I	Cl	H	H	H	S	13	97	30.7	0.004 ± 0.002
**14**	**3n**	OMe	Cl	H	H	H	S	69	96	13.4	0.038 ± 0.021
**15**	**3o**	H	Cl	F	H	H	S	47	95	67.2	
**16**	**3p**	H	Cl	Cl	H	H	S	55	90	36.0	1.1 ± 0.3
**17**	**3q**	Br	Cl	Br	H	H	S	41	95	27.9	0.36 ± 0.24
**18**	**3f′**	H	Cl	H	H	H	**R**	54	91	27.4	850 ± 420
**19**	**3l′**	Br	Cl	H	H	H	**R**	10	92	35.4	12.8 ± 4.4
**20**	**3r**	H	H	H	OMe	F	S	71	94	127	
**21**	**3s**	H	H	H	H	Cl	S	67	97	87	
**22**	**3t**	H	H	H	OMe	OMe	S	71	95	96.5	
**23**	**3u**	OMe	OMe	H	OMe	OMe	S	46	91	55.2	
**24**^b^	**4**	H	H	H	H	H	S	68	97	29.2	2900 ± 580
**25**^c^	**5**	H	H	H	H	H	S	42	96	134	

a Inhibition of Hh-dependent osteogenesis in C3H10T1/2 cells at 10 μM was investigated for all compounds. IC_50_ values were determined for derivatives that suppressed osteogenesis by more than 50%. Data are mean values ± SEM (*n* = 3).

b Five-membered ring analogue.

c Seven-membered ring analogue (see also Figure S2).

Investigation of the 8-oxotetrahydroprotoberberines in different cell-based assays identified potent inhibitors of Hh-induced osteogenesis in C3H10T1/2 cells that partially suppressed the differentiation process to the level of approx. 30% (Table 2). In vertebrates, Hh signaling is essential during embryonic development and is involved in tumorigenesis.^24^ Activation of Hh signaling through binding of an Hh ligand to the receptor Patched 1 (PTC1) results in the translocation of the receptor Smoothened (SMO) into the primary cilium and subsequent release of glioma-associated oncogenes 2 and 3 (GLI2/3) from the negative regulator suppressor of fused (SUFU) and its translocation to the nucleus to activate the expression of Hh target genes, e.g., *Gli1* and *Ptch1*.^24,25^ In the murine mesenchymal stem cell line C3H10T1/2, activation of Hh signaling using the SMO agonist purmorphamine induces osteoblast differentiation and, thus, expression of the osteogenesis marker alkaline phosphatase.^26,27^ Exploration of the underlying structure–activity relationship (SAR, Table 2 and Figure S2) reveals that introduction of a chloro- or bromo-substituent in the *OBoc*-hydroxymates (**1**) is required but not necessarily sufficient for the biological activity (see Table 2, entries 1–8, 15, and 19). The unsubstituted and *i*Pr-, Me-, and OMe-substituted cyclization products were inactive (Table 2, entries 1–4), but analogues halogenated at R^2^ clearly inhibited osteoblast differentiation. The biological activity decreased with increasing halogen size (Table 2, entries 6–8). The CF_3_-substituted derivative **3i** displayed similar activity as the Cl-substituted analogue (Table 2, entry 9). Halogen substitutions in the *para*-position are preferred over substitutions in the *meta-* and *ortho*-positions (Table 2, entry 10). Additional introduction of a halogen or of a methoxy group at R^1^ into the monochlorinated compound (Table 2, entries 11–14) or additional halogens at R^3^ (Table 2, entries 15–16) improves potency, whereby additional halogenation in R^1^ is preferred. Increasing the size of the halogen improves activity (Br and I *vs* Cl and F). However, simultaneous halogenation at R^1^ and R^3^ did not further increase the potency (Table 2, entry 17). The absolute configuration of the stereocenter is important for bioactivity because (*S*)*-*enantiomers show much higher potency than the respective *(R)-*enantiomers (Table 2, compare entries 6 and 18 as well as 12 and 19). Modifications on the styrene part strongly reduced biological activity (Table 2, entries 20–23). Analogues **4** and **5** in which the piperidine ring is replaced by a five- or seven-membered ring (see above) were also inactive (Table 2, entries 24 and 25).

**Figure 2:**
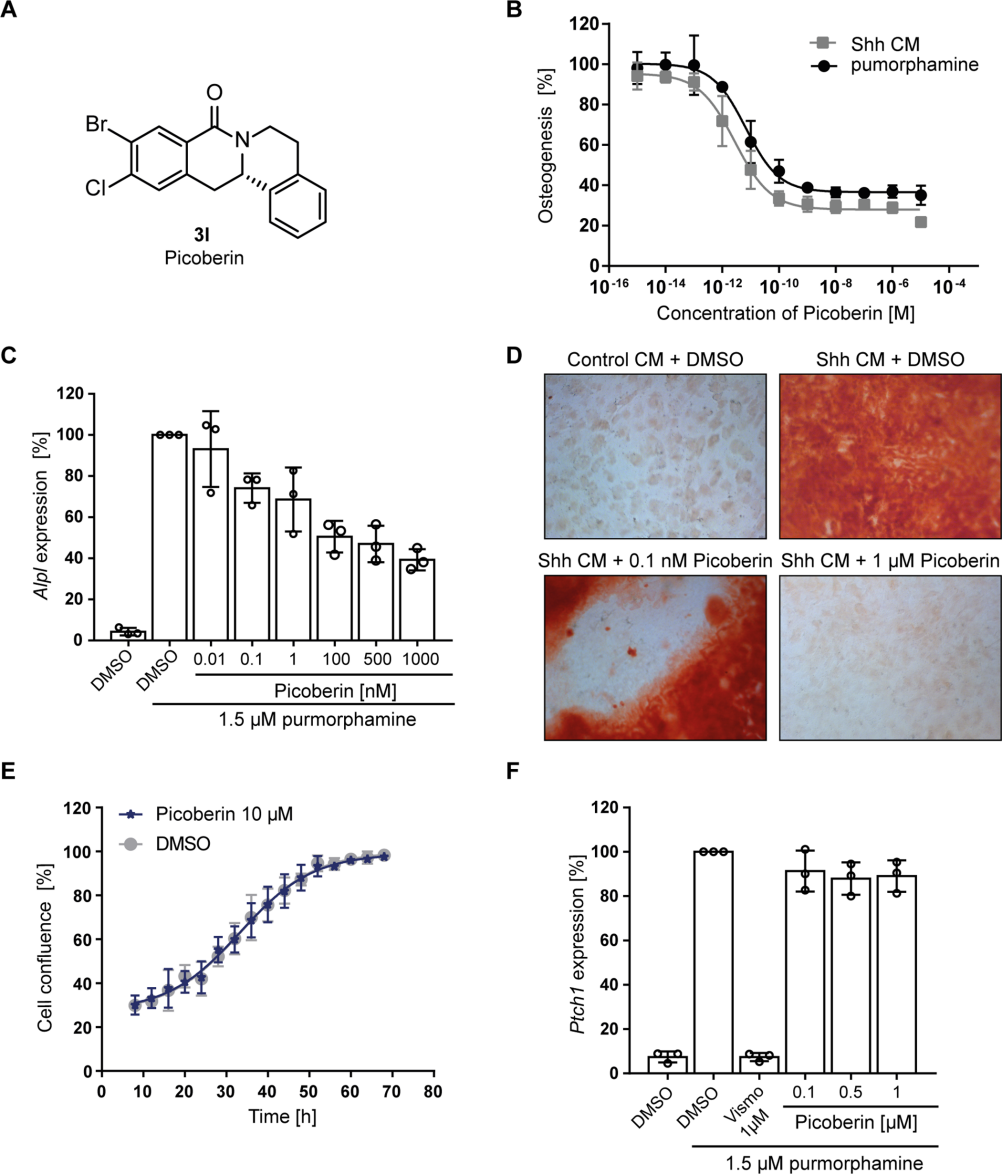
Characterization of Picoberin as an inhibitor of Hh-dependent osteoblast differentiation. (A) Structure of analogue **3l** (Picoberin). (B) Osteoblast differentiation. C3H10T1/2 cells were treated with 1.5 μM purmorphamine or Shh conditioned medium (CM) and Picoberin or DMSO for 96 h. The activity of alkaline phosphatase was determined as a measure of osteogenesis. (C) Expression of *Alpl*. C3H10T1/2 cells were treated with the compounds for 96 h, and *Alpl* expression levels were quantified by means of RT-qPCR. (D) Matrix mineralization assay. C3H10T1/2 cells were treated with Shh CM or control conditioned medium for 21 days. Calcium deposits were stained with Alizarin Red. Representative images (*n* = 3) acquired at 10-fold magnification are shown. (E) Influence on cell viability. C3H10T1/2 cells were treated with the compound, and cell confluence was monitored using IncuCyte ZOOM. (F) Expression of the Hh target gene *Ptch1* in C3H10T1/2 cells upon treatment with the compounds for 96 h. All data are mean values (*n* = 3, *N* = 3) ± SD.

Compound **3l** (Figure 2A) was identified as a highly potent inhibitor of purmorphamine- and Sonic Hedgehog (Shh)-induced osteogenesis with a low picomolar IC_50_ of 3 ± 1 pM and was therefore termed Picoberin (Figure 2B). The compound dose-dependently inhibited the expression of the alkaline phosphatase gene *Alpl* (Figure 2C) and suppressed bone mineralization (Figure 2D). Despite the high potency, Picoberin did not affect cell viability at concentrations up to 10 μM (Figure 2E). Picoberin neither modulated the expression of Hh target genes *Ptch1*, *Gli1*, and *Gli2* (Figure 2F and Figure S3A,B) nor altered GLI2/3-dependent reporter gene activity using Shh-LIGHT2 cells.^28^ Thus, Picoberin does not target canonical Hh signaling.

Besides Hh signaling, Wnt, BMP, Notch, and the TGFβ pathway are important during the differentiation cascade.^29^ Picoberin also inhibited Wnt3A-induced osteoblast differentiation of C3H10T1/2 cells, although alkaline phosphatase activity after stimulation with Wnt3A was only upregulated at later time points compared to stimulation with purmorphamine (Figure S3C,D). However, Picoberin did not modulate Wnt3A-induced TCF/LEF-dependent reporter activity (Figure S3E) and, thus, canonical Wnt signaling. Picoberin did not modulate TGF-β signaling (Figure S3F) or BMP-4-induced osteoblast differentiation of C2C12 cells (Figure S3G). In contrast, osteogenesis of C2C12 cells induced in the presence of BMP-4 and purmorphamine was partially inhibited in the presence of picomolar concentrations of Picoberin (Figure S3H), suggesting an Hh-dependent mode of action. G-protein coupled receptors (GPCRs) and nuclear receptors are involved in osteoblast differentiation and bone regeneration,^30,31^ but Picoberin did not modulate the activity of 168 GPCRs and 19 nuclear receptors (Tables S1 and S2).

**Figure 3:**
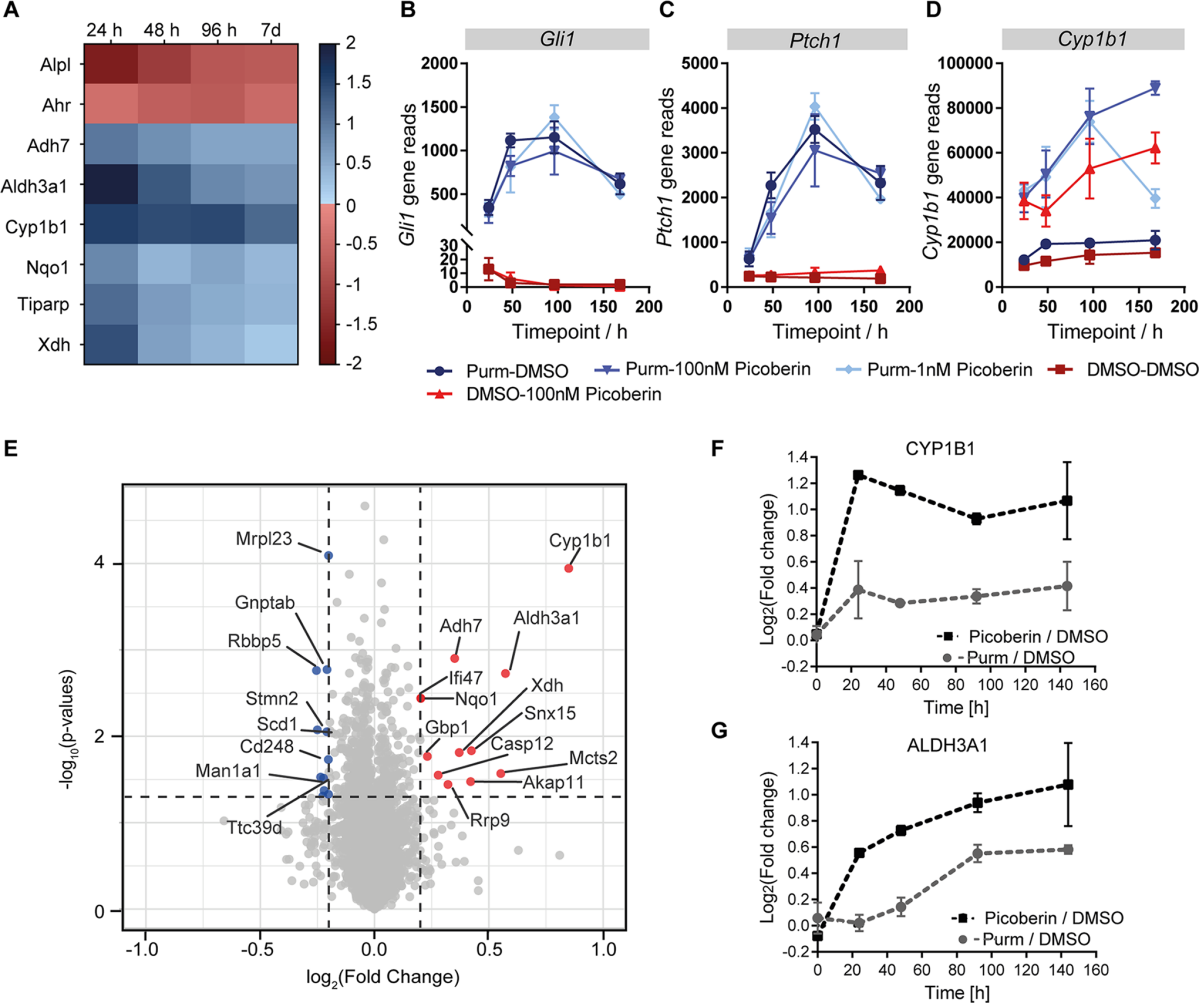
Global transcriptome and proteome profiling of C3H10T1/2 cells in the presence and absence of Picoberin. (A–D) Transcriptome analysis of C3H10T1/2 cells that were treated with 1.5 μM purmorphamine and Picoberin (1 or 100 nM) or DMSO. (A) Heatmap of fold changes of AhR target genes in cells that were treated with 1.5 μM purmorphamine and 1 nM Picoberin related to cells treated with 1.5 μM purmorphamine and DMSO. (B–D) Total gene reads obtained for *Gli1*, *Ptch1*, and *Cyp1b1*. (E–G) Global proteome analysis of C3H10T1/2 cells that were treated with 1.5 μM purmorphamine and 1 nM of Picoberin or DMSO. (E) Volcano plot of log_2_ fold changes for samples treated with 1.5 μM purmorphamine and 1 nM Picoberin related to samples treated with 1.5 μM purmorphamine and DMSO for 48 h. Red dots represent upregulated and blue dots downregulated genes. (F, G) Time-resolved fold changes for CYP1B1 and ALDH3A1 protein levels in samples treated with 1.5 μM purmorphamine and 1 nM Picoberin related to samples treated with 1.5 μM purmorphamine and DMSO. All data are mean values (*n* = 3) ± SD.

Structure variation did not identify a site in Picoberin that tolerates modification without loss of activity and that would be suitable for the attachment of a linker for affinity-based enrichment of target proteins. For this reason, time-dependent global transcriptome (Figure 3A–D) and global proteome (Figure 3E–G) analyses of C3H10T1/2 cells were performed in the presence and absence of Picoberin to identify the mode of action.

Transcriptome profiling confirmed reduced levels of the osteogenic marker *Alpl* upon treatment with Picoberin (Figure 3A). In line with our initial data, purmorphamine clearly upregulated the expression of Hh target genes (Figure S4), whereas no effect of Picoberin was observed (Figure 3B,C). Interestingly, Picoberin upregulated genes encoding phase I and II metabolic enzymes, such as *Cyp1b1*, *Aldh3a1*, *Adh7*, *Nqo1*, *Xdh*, and *Tiparp* (Figure 3A,D and Figure S5A). Pathway over-representation analysis linked the enrichment of these genes to the activation of the aryl hydrocarbon receptor (AhR) (Figure S5B). In line with this observation, global proteome profiling showed selective upregulation of cytochrome 1B1 (CYP1B1), aldehyde dehydrogenase 3 family member A1 (ALDH3A1), alcohol dehydrogenase 7 (ADH7), NAD(*P*)H quinone dehydrogenase 1 (NQO1), and other metabolic enzymes after 24, 48, and 96 h (Figure 3E–G and Figure S6A). These proteins are encoded by the genes, whose expression is regulated by AhR. Indeed, pathway over-representation analysis again strongly pointed toward activation of AhR signaling (Figure S6B). Thus, both profiling approaches suggest AhR as a molecular target of Picoberin.

AhR is a ligand-activated transcription factor that senses diverse ligands, including environmental toxins and endogenous microbial- and food-derived ligands, and induces the expression of various genes, such as detoxifying enzymes.^32,33^ Inactive AhR is located in the cytoplasm in a complex with two heat-shock proteins 90 (HSP90), AhR interacting protein (AIP), prostaglandin E synthase 3 (P23), and SRC.^33^ Upon ligand binding, AhR translocates into the nucleus, where it binds to xenobiotic response elements (XRE) in promoter sequences and activates transcription of species- and cell-type-specific target genes, including *Cyp1a1* and *Cyp1b1*.^34^

**Figure 4:**
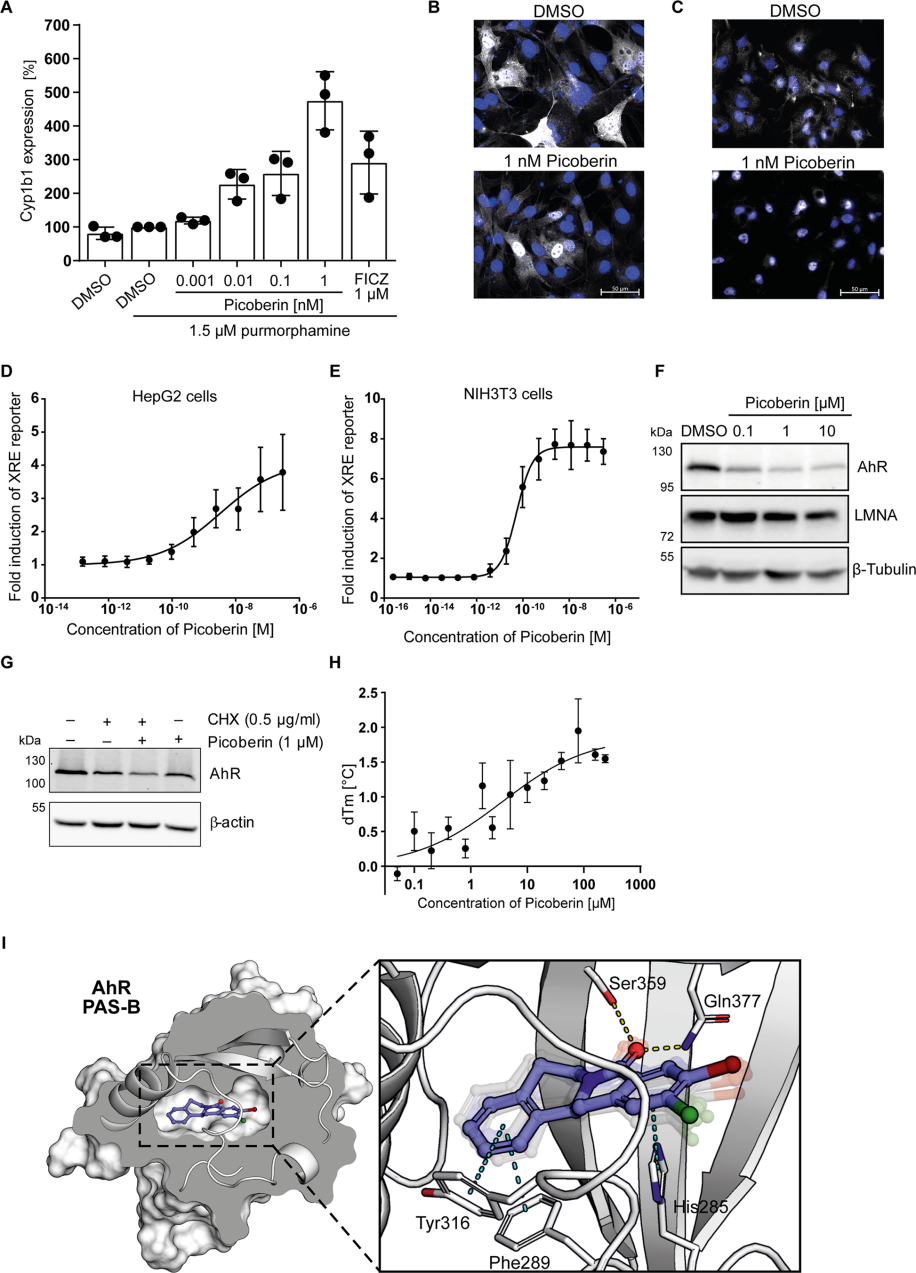
Characterization of Picoberin as an AhR agonist. (A) Influence on Cyp1b1 gene expression in C3H10T1/2 cells that were treated with 1.5 μM purmorphamine and Picoberin or DMSO for 96 h detected by RT-qPCR. (B, C) Localization of AhR in C3H10T1/2 expressing mouse AhR-FLAG (B) or HepG2 cells (C) that were treated with 1 nM Picoberin or DMSO for 24 h (B) or 15 min (C). AhR (white) was detected using an anti-FLAG antibody for B or an anti-AhR antibody for C. Nuclei were stained using DAPI (blue). (D) XRE-dependent reporter gene assay in HepG2 cells that were treated with Picoberin or DMSO for 4 h. (E) XRE-dependent reporter gene assay in NIH-3T3-XRE:fluc cells upon treatment with Picoberin or DMSO for 4 h. (F) AhR protein levels after treatment of HepG2 cells with Picoberin or DMSO for 4 h. The uncropped blots are shown in Figure S7. (G) Cycloheximide chase assay. HepG2 cells were pretreated with cycloheximide (CHX) for 60 min prior to the addition of Picoberin and further incubation for 18 h. The uncropped blots are shown in Figure S7. (H) Binding of Picoberin to the AhR-ARNT complex as detected using DSF. (I) Computational model of Picoberin bound to the PAS-B ligand-binding domain of mouse AhR. The presented complex is the final result of an induced fit ligand docking into an AlphaFold protein model followed by 100 ns of an all-atomic MD simulation. In the expanded view of the binding site, there are nine snapshots of ligand poses presented taken at regular intervals during the final 20 ns of the MD simulation. The solid ligand structure represents the orientation of Picoberin in the final frame of the simulation. Possible H-bonds and π–π interactions are represented by yellow and blue dashed lines, respectively. All data are mean values (*n* = 3) ± SD.

*Cyp1b1* mRNA levels were dose-dependently upregulated in C3H10T1/2 cells after 96 h. At 1 nM, Picoberin already strongly induced *Cyp1b1* expression, which exceeded the levels induced by the known AhR agonist 6-formylindolo[3,2-*b*]carbazole (FICZ) at 1 μM (Figure 4A).^35^ Picoberin induced translocation of AhR into the nucleus in C3H10T1/2 and in HepG2 cells (Figure 4B,C), revealing that Picoberin-mediated AhR activation is fast and, thus, most likely a direct effect. Picoberin dose-dependently induced XRE-dependent reporter activity after 4 h in murine and human cell lines with EC_50_ values of 3.2 ± 2.7 nM in human hepatocellular carcinoma HepG2 cells (Figure 4D), 58 ± 23 pM in murine embryonic fibroblasts (NIH-3T3 cells, Figure 4E), 0.7 ± 0.3 nM in human fibroblasts (HaCaT, Figure S7A), and 71.2 ± 35.6 nM in murine hepatoma cells (Hepa-1c1c7, Figure S7B).

Investigation of selected analogues for induction of XRE-dependent reporter activity in NIH-3T3 cells showed that overall activities matched well with the SAR observed in the osteoblast differentiation assay (Figure S7C). Known AhR agonists rapidly reduce cellular AhR protein levels via induction of proteasomal degradation, most likely to control the activity of the activated receptor.^36^ Picoberin dose-dependently decreased AhR protein levels in HepG2 cells after 4 h (Figure 4F and Figure S7D,E). Moreover, Picoberin reduced AhR levels in the presence of the protein synthesis inhibitor cycloheximide (Figure 4G and Figure S7F). Thus, Picoberin induces AhR degradation rather than inhibition of protein synthesis, which further confirms AhR activation by Picoberin.

A direct interaction of Picoberin with AhR was analyzed using differential scanning fluorimetry. Picoberin increased the melting temperature of the AhR–ARNT complex but not of ARNT alone (Figure 4H and Figure S7G,H). AhR interacts with a variety of structurally different agonists, all of which are thought to bind to the Per-ARNT-Sim B (PAS-B) domain.^37,38^ Insight into the possible interactions of Picoberin with AhR was gained by computational modeling and simulation.^32,39,40^ Significant structural similarities were noted between Picoberin and the prototypical AhR agonist 2,3,7,8-tetrachlorodibenzodioxin (TCDD);^37^ thus, both compounds might embody similar pharmacophores. The binding of TCDD to AhR has been well characterized in several biochemical and computational studies,^41−48^ allowing the use of this ligand as a reference and a control during the construction of a Picoberin–AhR interaction model. Because a crystal structure of the mouse AhR PAS-B has not been determined, we utilized two protein models of murine PAS-B, one based on the AlphaFold prediction^49^ and the other obtained from the SWISS-MODEL homology-modeling server, built using the Circadian Locomotor Output Cycles Kaput (CLOCK) protein template structure (PDB ID: 4F3L).^50^ AlphaFold predicted the structure of the ligand-binding domain with satisfactory confidence and low expected position error (Figure S8A,B). Further evaluation using structural quality estimating programs ProSA, QMEAN, and PROCHECK afforded satisfactory results (Table S3).^51−54^ Alignment of the obtained models with murine and human crystal structures of HIF2α and calculation of RMSD (root-mean-square deviation) between the structures showed that the resulting RMSD values were below 1.1 Å for all considered structures (Table S4). The recently released crystal structures of AhR PAS-B from *Drosophila melanogaster* (42% sequence identity with murine AhR PAS-B) are comparable to the yielded models with RMSD values below 1 Å, thus further increasing confidence in the predicted structures (Figure S9). RMSD between the AlphaFold and the homology model is equal to 0.842 Å, and a visual evaluation of the models revealed close structural similarity between the two predictions (Figure S8C).

TCDD and Picoberin were modeled into the ligand-binding pocket of the PAS-B domain using *ab initio* induced-fit docking (IFD). The best IFD poses of Picoberin and TCDD scored with Δ*G*_bind_ were similar for the AlphaFold and the SWISS-MODEL homology structure. All-atomic MD simulations were performed using explicit waters over 100 ns, giving enough time for adjustment of the ligand position inside the pocket (Figures S10 and S11). TCDD and Picoberin occupied comparable positions within the pocket and have very similar pharmacophores, forming notable interactions with His265, Phe289, Ile319, Phe345, Leu347, and Gln377. In addition, Picoberin might form an H-bond with Ser359 and potential π–π interactions with Tyr316 (Figure 4I and Figures S12). The key interactions were observed with both protein models used. The results obtained for the TCDD-AhR models agree with reported biochemical data identifying the TCDD binding-fingerprint as well as previous *in silico* binding studies,^41−44^ thus validating the adopted computational methodology and the afforded protein–ligand models.

The AhR agonist TCDD inhibits osteoblast differentiation induced by dexamethasone, β-glycerophosphate (BGP), and ascorbic acid.^55,56^ Furthermore, AhR^–/–^ mice exhibit impaired bone fracture healing with delayed endochondral ossification.^57^ The known AhR agonists FICZ, TCDD, YH439, benzo[*a*]pyrene (BaP), and Tapinarof inhibited Hh-induced osteoblast differentiation (Figure S13A–E), indicating the functional involvement of AhR during osteogenesis. Surprisingly, the AhR antagonist CH-223191^58^ also inhibited Hh-induced osteogenesis of C3H10T1/2 cells (Figure S13F). As both AhR agonists and antagonist impair osteoblast differentiation, a biphasic influence of AhR signaling is conceivable wherein inactive and activated AhR signaling is required at different phases during the differentiation process. Such temporal behavior is well known, e.g., for Wnt signaling and cardiomyocyte differentiation.^29,59,60^

For genetic validation, we depleted AhR using RNA interference and explored the influence of the knockdown on osteogenesis and on the activity of Picoberin.

**Figure 5:**
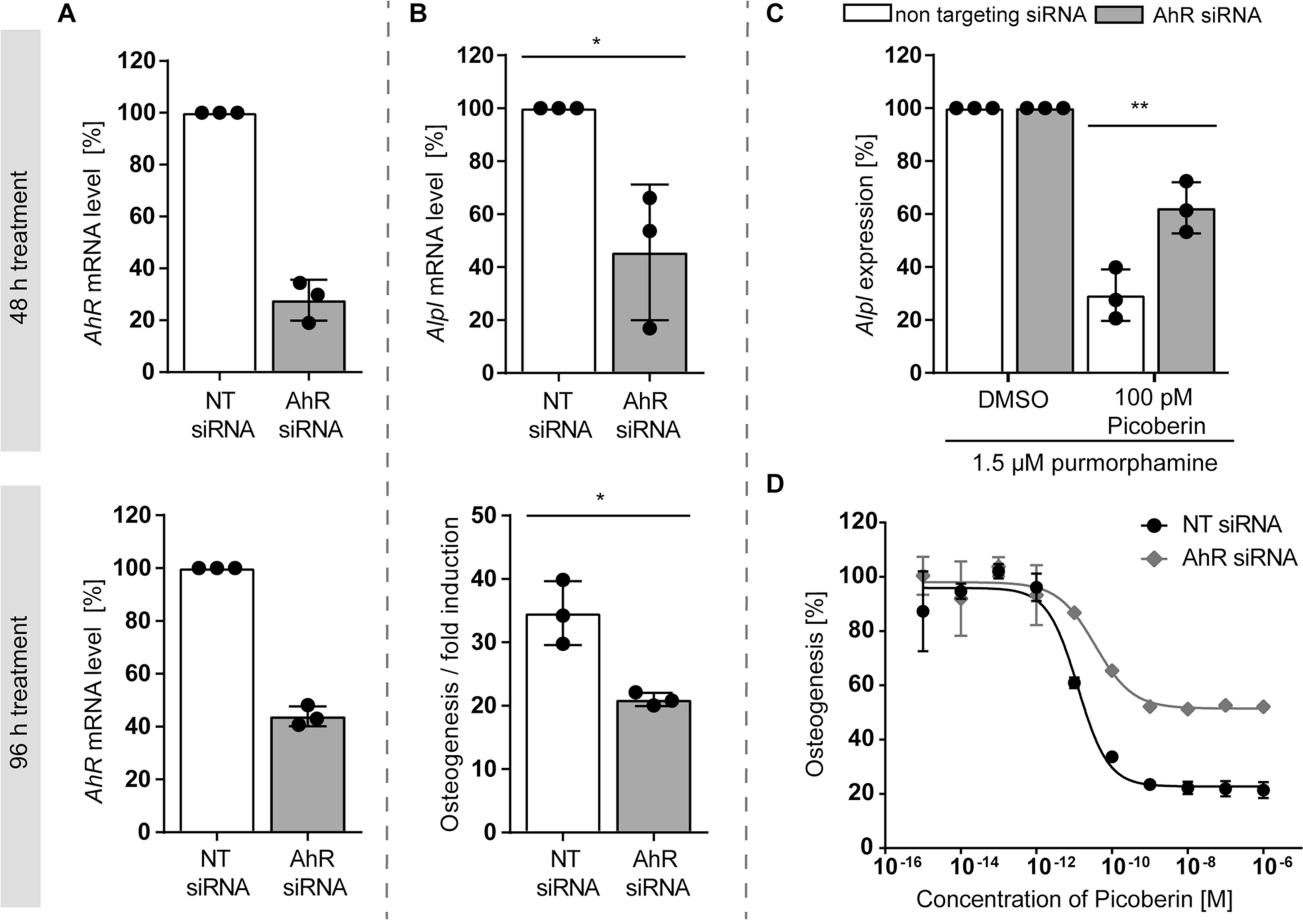
Influence of AhR knockdown on Hh-induced osteoblast. C3H10T1/2 cells were transfected with AhR-siRNA or nontargeting (NT)-siRNA for 24 h prior to treatment with 1.5 μM purmorphamine and Picoberin or DMSO for 48 or 96 h. (A) Knockdown efficiency based on AhR mRNA levels. (B) Effect of AhR knockdown on Hh-induced osteoblast differentiation. *p value < 0.05. (C, D) Osteoblast differentiation after 48 h (C) and 96 h (D). The activity of alkaline phosphatase was determined as a measure of osteogenesis. **p value < 0.05. All data are mean values (*n* = 3) ± SD.

An AhR knockdown of 67.2 ± 6.6 and 56.1 ± 3.1% was achieved 48 and 96 h post siRNA transfection, respectively (Figure 5A). Despite the partial knockdown, the *Alpl* mRNA level and alkaline phosphatase activity were reduced when compared to control cells (Figure 5B), suggesting a functional role of AhR signaling during Hh-induced osteoblast differentiation. These findings are in line with the negative impact of the AhR antagonist CH-223191 on osteogenesis and delayed bone healing in AhR^–/–^ mice.^61,62^ Interestingly, AhR knockdown also mitigated the inhibitory effect of Picoberin on osteoblast differentiation after 48 and 96 h (Figure 5C,D). These data strongly point toward AhR as the functional target of Picoberin during Hh-induced osteogenesis and reveal a tight regulation of AhR expression and signaling for proper osteoblast differentiation.

**Figure 6:**
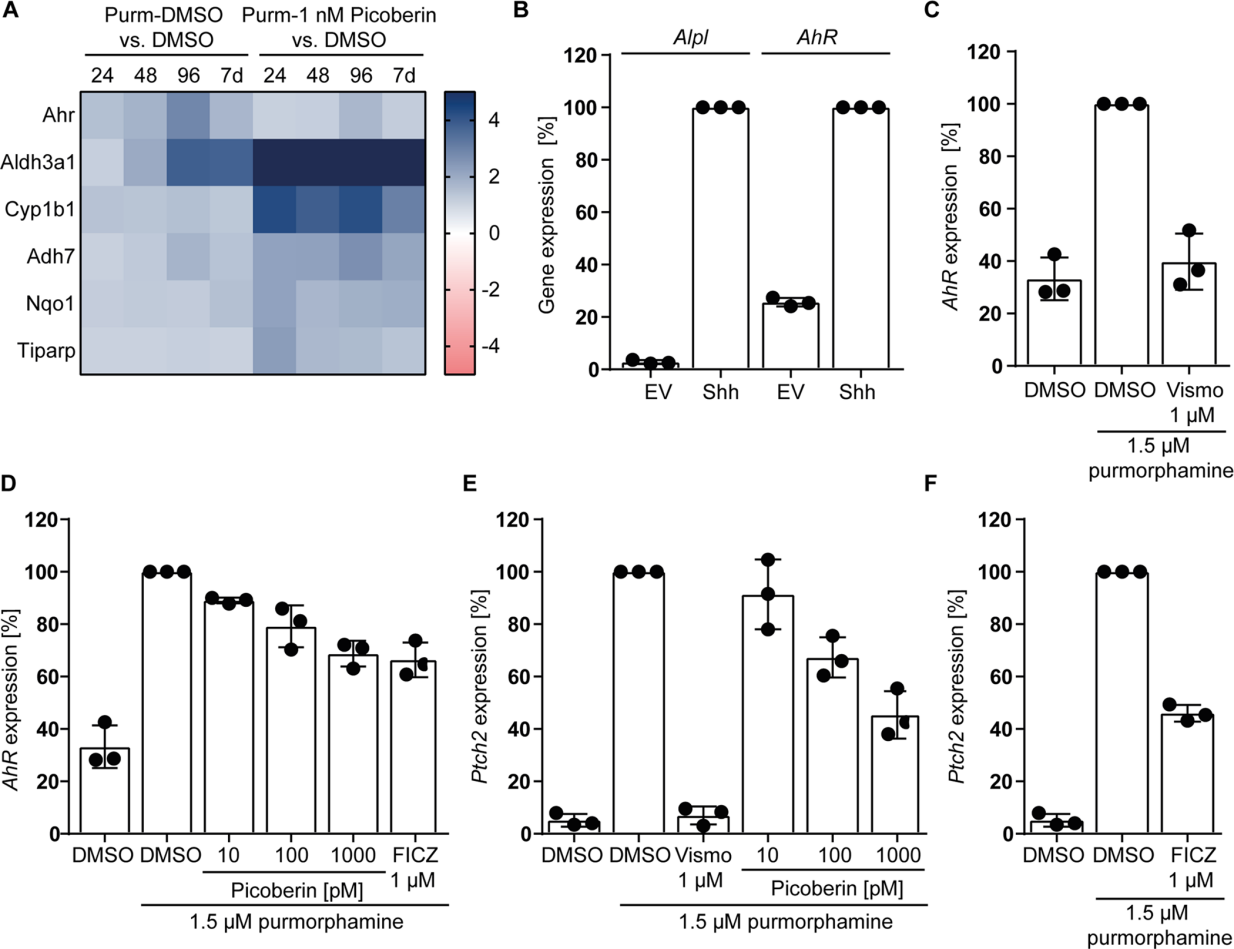
Regulation of *AhR* and *Ptch2* during Hh-induced osteoblast. (A) Heatmap of log2(fold changes) of selected AhR-related genes (NGS data). C3H10T1/2 cells were treated with 1.5 μM purmorphamine and 1 nM Picoberin or DMSO, or only with DMSO. (B) *Alpl* and *Ahr* expression. C3H10T1/2 cells were treated with Sonic Hedgehog (Shh) conditioned medium or empty vector (EV) conditioned medium for 96 h prior to RT-qPCR. (C-F) *AhR* and *Ptch2* expression. C3H10T1/2 cells were treated with 1.5 μM purmorphamine and Picoberin, Vismodegib, FICZ, or DMSO for 96 h (*AhR*, C–D) or 48 h (*Ptch2*, E–F) followed by RT-qPCR. All data are mean values (*n* = 3) ± SD.

Analysis of the whole transcriptome data revealed an upregulation of *AhR* expression in C3H10T1/2 cells upon stimulation with purmorphamine, which is reduced by co-treatment with 1 nM of Picoberin (Figures 4A and 6A). This finding is in line with increased *AhR* mRNA levels during BMP- and ascorbate-induced osteoblast differentiation of neural rat calvaria and supports a functional role of the AhR during osteogenesis.^62^ Shh strongly increased *AhR* mRNA levels (Figure 6B). Furthermore, the known Hh pathway inhibitor Vismodegib completely inhibited this effect (Figure 6C). These data confirm the Hh-dependent regulation of *AhR* expression in C3H10T1/2 cells. Picoberin and the AhR agonist FICZ partially reduced purmorphamine-induced AhR upregulation after 96 h (Figure 6D). Interestingly, a significant difference in *Ptch2* expression levels was detected after 48 h of treatment with purmorphamine and Picoberin. The murine *Ptch2* gene shares 57% sequence homology with murine *Ptch1* and is a target gene of Hh signaling (Figure 6E).^63^ The known AhR agonist FICZ also reduced *Ptch2* expression (Figure 6F). High levels of *Aldh3a1* and, to a much lower extent, *Cyp1b1* were retained throughout the co-treatment with purmorphamine and Picoberin, i.e., 96 h, whereas the level of *AhR* mRNA was reduced (Figure 6A). Aldehyde dehydrogenases (ALDHs) oxidize not only exogenous but also endogenous aldehydes and participate in the biosynthesis of molecules that are involved in cell homeostasis and have nonenzymatic function.^64,65^ More importantly, ALDH activity modulates osteogenesis dependent on the cell type and stimulus.^65^ Thus, the constantly high level of ALDH3A1 upon treatment with Picoberin may contribute to impaired osteoblast differentiation.

## Conclusions

We developed a new catalytic enantioselective C–H activation method for the synthesis of natural-product-inspired 8-oxotetrahydroprotoberberines that proved to be highly potent inhibitors of Hh-induced osteogenesis with a low picomolar IC_50_ value for the most potent derivative, termed Picoberin. Picoberin acts as an AhR agonist and induces the expression of AhR target genes, thereby suppressing osteoblast differentiation. Our data demonstrate a functional role of AhR during Hh-dependent osteoblast differentiation and cross talk between Hh and AhR signaling during osteogenesis. Despite the strikingly high potency, which allows the application of the compound at very low concentrations, Picoberin is not cytotoxic up to 10 μM. Although dioxin is linked to serious toxicological issues, activation of the AhR may not be per se undesired as, only recently, the AhR agonist Tapinarof was approved for the treatment of plaque psoriasis. In light of this development, AhR is currently being explored as a drug target, and Picoberin may not only be considered as a tool compound for the study of AhR biology but also open up novel avenues for the development of selective AhR agonists for therapeutic applications.

## Experimental Section

### Biology Methods

#### Materials

The used reagents were obtained from the following vendors or sources: mouse monoclonal anti-AhR: Cat# sc-133088; RRID: AB_2273721 (Santa Cruz); mouse monochlonal anti-Lamin A: Cat# sc-71481, RRID: AB_2136165 (Santa Cruz); mouse monochlonal anti- β-Tubulin: Cat# T5076, RRID: AB_532291 (Sigma-Aldrich); donkey anti-mouse infrared dye 800CW-labeled: Cat# 926-32212; RRID: AB_621847 (LI-COR); 4′,6-diamidino-2-phenylindol (DAPI): Cat# D9542, CAS: 28718-90-3 (Sigma-Aldrich); purmorphamine: Cat# 10009634, CAS: 483367-10-8 (Cayman Chemical); 6-formylindolo[3,2-*b*]carbazole (FICZ): Cat# SML1489-1MG, CAS: 172922-91-7 (Sigma-Aldrich); benzo(*a*)pyrene, Cat# 50328, CAS: 50-32-8 (Sigma-Aldrich); CH223191: Cat# C8124, CAS: 301326-22-7 (Sigma-Aldrich); Tapinarof: Cat# S9700, CAS: 79338-84-4 (Selleckchem); TCDD: Cat# ED-560, CAS: 1746-01-6 (Cambridge Isotope Laboratories, Inc.); TMT10ple: Cat# 90110 (Thermo Fisher Scientific); Vismodegib: Cat# S1082, CAS: 879085-55-9 (Selleckchem); YH439: Cat# HY-100242, CAS:130112-42-4 (MedChemExpress); NP-40 alternative: Cat# 492016, CAS: 9016-45-9 (Merck); β-glycerophosphate (BGP) disodium salt: Cat# G9422, CAS:154804-51-0 (Sigma-Aldrich); CDP-Star: Cat# 11685637001 (Roche Diagnostics); DC Protein Assay Kit II: Cat# 000112 (BioRad); Dual-Luciferase Reporter Assay System: Cat# E1960 (Promega); QuantiTect Reverse Transcription Kit: Cat# 205313 (Qiagen); Qubit RNA BR Assay Kit: Cat# Q10210 (Thermo Fisher); SsoAdvanced Universal SYBR Green Supermix: Cat# 1725274 (Bio-Rad); DMEM: Cat# P04-03550 (PAN); fetal bovine serum, Cat# 10270-106 (Gibco); fetal calf serum: Cat# SH30087 (GE Healthcare); and sodium pyruvate: Cat# P04-43100 (PAN).

#### Cell Lines

NIH/3T3 cells (murine fibroblasts, DSMZ, ACC-59) and C3H10T1/2 cells (murine embryonic stem cells, ATCC, #CCL-226) were cultured in Dulbecco’s modified Eagle’s medium (DMEM, high glucose, PAN, #P04-03550) supplemented with 10% FCS (Fisher Scientific, #10136253, heat-inactivated) and 1 mM sodium pyruvate (PAN, #P04-43100). Shh-LIGHT2 cells^28^ (NIH-3T3 cells stably transfected with GLI2/3 responsive firefly luciferase and pRL-TK constitutive Renilla-luciferase plasmid) were cultured in the same medium as the parental cell line NIH/3T3, and the medium was supplemented with 400 μg/mL Geneticin (Sigma Aldrich, #A1720) and 150 μg/mL Zeocin (InvivoGen, #ant-zn-1) as selecting agents. NIH-3T3 virally transfected with XRE-dependent firefly reporter and *Renilla* luciferase were cultured in the same medium as the parental cell line supplemented with 5 μg/mL puromycin (ChemCruz) and 5 μg/mL blasticidin (Gibco). HepG2 cells (human hepatocarcinoma cells, DZMS, ACC-180), Wnt3A-expressing L-cells and normal L-cells (ATCC), and HEK293T cells (human embryonic kidney cells ATCC, CRL1268) were cultured in DMEM (PAN, #P04-03550) supplemented with 10% FBS (Gibco, #10270-106) and 1 mM sodium pyruvate (PAN, #P04-43100) and MEM nonessential amino acids (NEAA) (PAN, #P08-32100). Hepa1c1c7 (mouse hepatocytes, ATCC CRL-2026) XRE reporter cells,^66^ HEK293T cells (human embryonic kidney epithelial cells, ATCC CRL-11268), and HaCaT cells were grown in DMEM with GlutaMAX supplement, 10% (v/v) heat-inactivated FBS (Bio-sera), and 1 mM sodium pyruvate (Gibco).

#### Osteoblast Differentiation Assays

The Hh-dependent osteoblast differentiation assay was performed in C3H10T1/2 cells.^26^ Briefly, 6000 C3H10T1/2 were seeded per well in white 96-well plates with clear bottoms (Corning, #353075) and allowed to attach for 6 h. Afterward, cells were treated with 1.5 μM purmorphamine, Shh-conditioned medium, or Wnt3A-conditioned medium and different concentrations of the compounds or DMSO as a control. After incubation for 96 h, the cell culture medium was aspirated, and 40 μL per well of the commercial luminogenic ALP substrate CDP-Star (Roche, #11685627001) was added. After 1 h of incubation at room temperature and in the dark, the luminescence signal was read using the Spark plate reader (Tecan). To calculate IC_50_ values, a four-parameter fit was performed using GraphPad Prism 7 (GraphPad Software, USA). ALP activity was set to 100% for cells that were treated with purmorphamine or an Shh-conditioned medium.

For osteoblast differentiation assays with C2C12 cells, 2000 cells/well were seeded in DMEM; supplemented with 6% heat-inactivated FBS, GlutaMAX, and penicillin–streptomycin in 384-well plates (Greiner); and grown for 16 h. Compounds/DMSO were added in triplicates with the Echo Liquid Handler (Labcyte). BMP-4 (7.5 ng/mL) and purmorphamine (1 μM) were added to the wells to reach a final volume of 50 μL/well, and plates were incubated for 72 h. To monitor cellular ALP activity, substrate-containing CDP-Star (Roche) was premixed with a lysis buffer (1:100), and 15 μL of this mix was added per well after aspiration. The plates were shaken for 5 min, shortly centrifugated, and incubated at room temperature in the dark for 1 h. The luminescence was measured on a Paradigm Reader (excitation 0.2 s), and data were exported to Excel. For analysis, the ALP activity of inductive BMP-4 or synergistic BMP-4/purmorphamine was set to 100% to calculate the activity of tested compounds.

#### Cell Viability and Growth

To investigate the influence of 8-oxotetrahydroprotoberberines on cell growth and viability, 2000 C3H10T1/2 cells were seeded per well in 96-well plates (Corning #353075) and incubated at 37 °C and 5% CO_2_ overnight. The next day, cells were treated with different concentrations of the compounds or DMSO as a control. Cells were imaged every 2 h for a total of 72 h using the live-cell imaging system IncuCyte ZOOM (Sartorius). Confluence data were obtained from the time-lapse image acquisition using the IncuCyte ZOOM software (2018A).

#### Matrix Mineralization Assay

To investigate the effect of Picoberin on matrix mineralization, 6 × 10^4^ C3H10T1/2 cells per well were seeded into 12-well plates (Sarstedt, #83.3921) and incubated at 37 °C and 5% CO_2_ for 48 h to a confluence of 80%. Cells were then treated with mineralization medium (Shh-conditioned medium containing 50 μg/mL l-ascorbic-3-phosphate (Sigma-Aldrich, #8960), 10 mM β-glycerophosphate (Sigma-Aldrich, #G9422), and 10% heat-inactivated FCS) or control medium (empty vector-conditioned medium containing 50 μg/mL l-ascorbic-3-phosphate, 10 mM β-glycerophosphate, and 10% heat-inactivated FCS) and different concentrations of the compounds or DMSO as a control. The medium was refreshed every 3–4 days for 21 days. Cells were then fixed using 3.7% formaldehyde for 10 min and washed twice with ddH_2_O. Afterward, mineralized nodules were stained with 40 mM Alizarin Red S solution (ChemCruz, #sc-205998A, pH = 4.1, adjusted using 10% ammonium hydroxide) for 40 min. To remove unspecific staining, cells were washed five times with ddH_2_O prior to microscopy analysis using the Zeiss Observer Z1 (Carl Zeiss, Germany).

#### Generation of Wnt3A-Conditioned Medium

The Wnt3A-conditioned medium was prepared as described by Friese et al.^67^ Briefly, 2.5 × 10^5^ L-cells stably transfected with a Wnt3A plasmid (ATCC, CRL-2647) were seeded in 25 mL growth medium (DMEM containing 10% FCS and 1 mM sodium pyruvate) into a T75 cell culture flask and incubated for 4 days at 37 °C and 5% CO_2_. The medium was collected and centrifuged for 15 min at 1000*g* and passed through a 0.2 μm syringe filter to remove any residual cells and debris. Cells were supplemented with 25 mL fresh growth medium. After another 3 days, the conditioned medium was harvested for a second time, as described above. Both parts of the conditioned medium were combined and stored at 4 °C until further usage. For the control conditioned medium, untransfected L-cells (ATCC, CRL-2648) were treated as described above.

#### Preparation of Shh-Conditioned Medium

For the generation of Shh conditioned medium, HEK293T cells (ATCC, CRL-11268) were transiently transfected with an Shh-encoding plasmid (pcDNA3.1 ShhN was a gift from Philip Beachy; Addgene plasmid #37680; http://n2t.net/addgene:37680; RRID: Addgene_37680) or with an empty vector (Invitrogen, #V79020) as a control. Briefly, 2 × 10^6^ cells were seeded into a T-75 cell culture flask (Sarstedt, #83.3911) and grown overnight. Afterward, cells were transfected with the plasmids using the FuGENE HD transfection reagent (Promega, #E2311). After incubation for 24 h, the medium was replaced by serum-reduced medium (DMEM, supplemented with 0.5% FCS and 1 mM sodium pyruvate (PAN, #P04–43100)). Another 24 h later, the medium was harvested by 20 min centrifugation at 2000*g*, sterile filtered using a 0.2 μm syringe filter (Sarstedt, #83.1826.001), and stored at 4 °C. Cells were supplied with fresh serum-reduced medium and were again incubated for 24 h. Afterward, the Shh-conditioned medium was harvested for a second time as described above. For assays, both fractions were combined and diluted with a fresh serum-reduced medium in a ratio of 3:1 and supplemented with 10% FCS. The Shh-conditioned medium was stored at 4 °C until further usage.

#### GLI-Dependent Reporter Gene Assays

The GLI-dependent reporter gene expression was detected using Shh-LIGHT2 cells.^28^ Briefly, 2.5 × 10^4^ cells per well were seeded into 96-well plates (Corning, #353075) and grown overnight. Afterward, cells were treated with 2 μM purmorphamine (Cayman Chemical, #10009634) in serum-reduced medium to activate Hh signaling. Additionally, cells were treated with different concentrations of Picoberin or DMSO as a control. After 48 h incubation, firefly and *Renilla* luciferase activity was measured by means of the Dual-Luciferase Reporter Assay System (Promega, #E1960) using the Spark plate reader (Tecan, Austria). Firefly luciferase signals were normalized to the corresponding *Renilla* luciferase signals, and the obtained ratio for DMSO and 2 μM purmorphamine treated samples was set to 100%. To calculate IC_50_ values, a nonlinear regression was performed using a four-parameter fit using GraphPad Prism 7 (GraphPad Software, USA).

#### TCF/LEF-Dependent Reporter Gene Assay

HEK293-T cells were transfected with plasmids encoding for Wnt-3A, a TCF/LEF-dependent SuperTop-Flash luciferase (pSTF, Addgene, #12456), and a constitutively expressed Renilla luciferase (pRL-TK-Rluc, Promega) and incubated for 6 h at 37 °C and 5% CO_2_. Afterward, transfected cells were harvested, and 2.5 × 10^4^ cells per well were seeded into a transparent 96-well cell culture plate in a volume of 90 μL. After 1 h of incubation at 37 °C and 5% CO_2_, cells were treated with the compounds. The final DMSO concentration in all wells was 0.3%. After 24 h, firefly and *Renilla* luciferase activity was measured by means of the Dual-Luciferase Reporter Assay System (Promega, #E1960) using the Spark plate reader (Tecan, Austria). Firefly luciferase signals were normalized to the corresponding *Renilla* luciferase signals, and the obtained ratio for DMSO-treated samples was set to 100%. To calculate IC_50_ values, a nonlinear regression was performed using a four-parameter fit using GraphPad Prism 7 (GraphPad Software, USA).

#### SBE-4-Dependent Reporter Gene Assay

A total of 3 × 10^6^ HEK293-T cells were bulk transfected with plasmids encoding for an SBE-4-dependent firefly luciferase (SBE4-Luciferase, Addgene, #16495) and a constitutively expressed *Renilla* luciferase (pRL-TK-Rluc, Promega). After incubation for 12 h at 37 °C and 5% CO_2_, cells were collected, and 2 × 10^4^ cells per well were seeded into a transparent 96-well cell culture plate. Cells were allowed to attach for 1 h and were then treated with the compounds or DMSO as a control. To induce TGFβ signaling, cells were additionally treated with 20 ng/mL TGFβ-I or DMSO as a control. The final DMSO concentration in all wells was 0.3%. After 24 h, firefly and *Renilla* luciferase activity was measured by means of the Dual-Luciferase Reporter Assay System (Promega, #E1960) using the Spark plate reader (Tecan, Austria). Firefly luciferase signals were normalized to the corresponding *Renilla* luciferase signals, and the obtained ratio for DMSO-treated samples was set to 100%. To calculate IC_50_ values, a nonlinear regression was performed using a four-parameter fit using GraphPad Prism 7 (GraphPad Software, USA).

#### Reverse Transcriptase-Quantitative PCR (RT-qPCR)

For gene expression analysis, C3H10T1/2 cells were seeded into 6-well plates (100,000 cell/well) or 12-well plates (60,000 cells/well), and cells were incubated for 48 h until they reached a confluence of about 80%. Cells were then treated with 1.5 μM purmorphamine and different concentrations of the compounds or DMSO as a control for 48 or 96 h. The total RNA was isolated using the RNAeasy Kit (Qiagen, #74104) including the optional DNase digestion step. RNA concentrations were determined with the RNA BR assay kit (Thermo Fisher, #Q10210) using the Qubit4.0 (Thermo Fisher). cDNA was obtained using the QuantiTect Reverse Transcription Kit (Qiagen, #205313). The relative mRNA amount of the genes of interest was evaluated using the QuantiFast SYBR Green PCR Kit (Qiagen, #204054). The SYBR Green signal was detected using the CFX96 Real-Time PCR Detection System (Bio-Rad, Germany), and relative expression levels were calculated using the ΔΔCt method,^68^ with *Gapdh* and *Ap3d1* as reference genes. Gene expression levels for samples that were treated with DMSO and 1.5 μM purmorphamine were set to 100%. The expression levels of all transcripts of interest were related to the respective positive control.

Employed primers (obtained from Sigma-Aldrich) were as follows: mouse *Gli-1* (NM_010296.2) fw: 5′-CACCGTGGGAGTAAACAGGCCTTCC-3′, rv: 5′-CCAGAGCGTTACACACCTGCCCTTC-3′; mouse *Ptch1* (NM_008957.3) fw: 5′-CTCTGGAGCAGATTTCCAAGG-3′, rv: 5′-TGCCGCAGTTCTTTTGAATG-3′; mouse *Alpl* (NM_007431.3) fw: 5′-ATCTTTGGTCTGGCTCCCATG-3′, rv: 5′-TTTCCCGTTCACCGTCCAC-3′; mouse *Gapdh* (NM_008084.3) fw: 5′-CAGTGCCAGCCTCGTC-3′*,* rv: 5′-CAATCTCCACTTTGCCACTG-3′; mouse *Ap3d1* (NM_007460) fw: 5′-CAGAGGGCTCATCGGTACAC-3′, rv: 5′-GCCGGAAGTCCAACTTCTCA-3′; mouse *AhR* (NM_013464) fw: 5′-CTGGTTGTCACAGCAGATGCCT-3′, rv: 5′-CGGTCTTCTGTATGGATGAGCTC-3′; mouse *Ptch2* (NM_008958.3) fw: 5′-GGCACTCACATCCGTCAACAAC-3′, rv: 5′-GAAGACGAGCATTACCGCTGCA-3′; and mouse *Cyp1b1* (NM_009994.2) fw: 5′-GGATGTGCCTGCCACTATTA-3′, rv: 5′-TGAACATCCGGGTATCTGGT-3′.

#### GPCR Panel

The gpcrMAX panel was performed by the LeadHunter service at Eurofins DiscoverX. The panel contained in total 168 GPCRs. The influence of Picoberin on these GPCRs was tested at a single concentration of 500 nM using the PathHunter β-arrestin assay in agonist and antagonist mode.

#### Nuclear Receptor Panel

The nhrMAX Biosensor panel was performed by the LeadHunter service at Eurofins DiscoverX. The panel contained in total 19 nuclear receptors. The influence of Picoberin on these nuclear receptors was tested at a single concentration of 500 nM using the PathHunter Nuclear hormone receptor assay in agonist and antagonist mode.

#### Whole Transcriptome Profiling (Next-Generation Sequencing)

For whole transcriptome profiling, 100,000 C3H10T1/2 cells/well were seeded into six-well plates and incubated at 37 °C and 5% CO_2_ for 48 h until they reached 80% confluence. Next, cells were treated with 1.5 μM purmorphamine in the presence of DMSO or different concentrations of Picoberin for 24 h, 48 h, 96 h, or 7 days. Total RNA was isolated using the RNAeasy Kit (Qiagen, #74104), including a DNase digestion step using the RNase-Free DNase Set (Qiagen, #79256). RNA was quantified using the RNA BR assay kit (Thermo Fisher, #Q10210) and the Qubit4.0 device (Thermo Fisher). Library preparation, Illumina sequencing, and data processing were performed at the NGS service at the Max Planck Genome Center Cologne. RNA quality and quantity were assessed by the Agilent Bioanalyzer Nanochip. Next, Poly-A RNA was enriched from 500 ng of total RNA by the Poly(A) mRNA Magnetic Isolation Module (New England Biolabs), and directional RNA-seq libraries were prepared as described in the Ultra II Directional RNA Library Prep Kit for Illumina (New England Biolabs). Library concentration was enriched by PCR with dual barcoded primers. Concentrations of the libraries were determined by the Qubit HS kit (Thermo), and the fragment size and quality of libraries were screened by Agilent TapeStation. Libraries were then diluted and pooled on a liquid handling station (Biomek i7, Beckman Coulter). Sequencing-by-synthesis was done on an Illumina HiSeq 3000 device at the Max Planck Genome Center Cologne in paired-end read mode 2 × 150 bp with a HiSeq 3000 PE Cluster Kit and a HiSeq 3000 SBS Kit (300 cycle). Finally, data were trimmed to 1 × 150 bp.

Data analysis was performed via CLC Workbench (Qiagen, version 12.0 and CLC Genomics Server version 11.0). For this purpose, the operations ″Trim Reads″, ″RNA-Seq Analysis″ (″Strand-specific = Reverse″), and ″Differential Expression for RNA-Seq″ functions were applied to the data in that order. Mouse genome version GRCm38 was used as reference. The obtained differential gene expression data were further analyzed using the Ingenuity Pathway Analysis (IPA) software (Qiagen, Version 70750971).

#### Protein Expression and Purification

Human AHR (aa 28-414)-ARNT (aa 85-465) (bHLH-PASA-PASB) heterodimer was expressed in the *Escherichia coli* strain Rosetta 2 (DE 3) with 0.1 mM IPTG and 20 μM β-Naphthoflavone overnight induction. Constructs were purified sequentially by affinity chromatography on Ni Sepharose resin and cation exchange in a buffer containing 20 μM β-Naphthoflavone. β-Naphthoflavone was then removed by size exclusion on a Superdex 200 PG gel-filtration column. Purified protein constructs were concentrated and stored in a buffer with 20 mM Tris pH 7.5, 300 mM NaCl, and 0.5 mM TCEP.

Mouse His-ARNT (mARNT 85-465 Δ274–297 C256S Δ351–358)^69^ expression was adapted from Huang et al. and Schulte et al.^70,71^ Protein expression was induced by addition of 0.5 mM IPTG and incubation overnight. Bacterial pellets were resuspended in 5× volume of lysis buffer (50 mM Na_3_PO_4_, 300 mM NaCl, 15 mM imidazole, 10% (v/v) glycerol, 1 mM TCEP, 0.2% Triton X-100 with 0.5 mM AEBSF, and 5 U/mL benzonase) and lysed using a cell disrupter (TS0.75, Constant System) at 1.35 kbar and one pass. The supernatant was incubated with 1.5 mL Ni-NTA agarose beads (Qiagen) for 3 h on ice under gentle shaking before loading into a gravity flow filter column and washed with 100 mL wash buffer (50 mM Na_3_PO_4_, 300 mM NaCl, 15 mM imidazole, 10% (v/v) glycerol, 1 mM TCEP, and 0.01% Triton X-100). The protein was eluted with an elution buffer (50 mM Na_3_PO_4_, 300 mM NaCl, 300 mM imidazole, 10% (v/v) glycerol, 1 mM TCEP, and 0.01% Triton X-100). The elution fractions were concentrated and further purified without protease cleavage by size exclusion chromatography (SEC) using a HiLoad Superdex S200 26/60 column and 20 mM HEPES, 200 mM NaCl, 5% (v/v) glycerol, and 1 mM TCEP as an SEC buffer. The ARNT fractions were concentrated to 800 μL with a concentration of 3.7 mg/mL and stored in the SEC buffer for further use.

#### Differential Scanning Fluorimetry (DSF)

DSF was performed using Thermo Fisher QuantStudio 7 Flex Real-Time PCR System. Experiments were carried out in 384-well plates with 5 μL reaction volumes. The assay buffer was 20 mM Tris pH 7.5, 300 mM NaCl, 0.5 mM TCEP, and 2.4% v/v DMSO. AHR-ARNT (0.2 mg/mL, 2.22 μM) was mixed with 14-point serial dilutions of Picoberin ranging from 50 nM to 240 μM. The Tm shifts were calculated with the Protein Thermal Shift software from the RT-PCR instrument.

To test a potential binding of Picoberin to ARNT alone, 0.5 mg/mL His-ARNT was incubated with 10 or 200 μM Picoberin (1% (v/v) DMSO) for 10 min (10 μM) or 30 min (200 μM) at 22 °C in HEPES buffer (20 mM HEPES, 200 mM NaCl, 1 mM TCEP, and 5% (v/v) glycerol). The thermal protein stability from 20 to 90 °C (1 °C/min) was measured by means of the intrinsic tryptophan/tyrosine fluorescence using the Prometheus NT.48 (NanoTemper Technologies, DE). Melting scans, first derivatives of melting scans, and melting temperatures were analyzed using the PR.ThermControl software (NanoTemper Technologies, DE).

Graphs were generated with GraphPad Prism 9.0 (GraphPad Software, Inc., USA).

#### Global Proteome Profiling

For whole proteome profiling 8.5 × 10^5^ C3H10T1/2 cells were seeded into T75 cell culture flasks (Sarstedt, #83.3911.002) and incubated at 37 °C and 5% CO_2_ for 48 h to reach a confluence of 80%. Cells were then treated with 1.5 μM purmorphamine and 1 nM Picoberin, 1.5 μM purmorphamine and DMSO, or only DMSO as a control. The final DMSO concentration was 0.25% in all samples. Cells were incubated with the compounds for 0 h, 24 h, 48 h, 96 h, or 7 days at 37 °C and 5% CO_2_. For cell lysis, the medium was removed, and cells were washed with 10 mL PBS prior to detachment using trypsin/solution EDTA (PAN-Biotech, #P10-023100) Cells were collected, and the cell pellet was washed in 10 mL ice-cold PBS. Cell pellets were resuspended in 1 mL ice-cold PBS, transferred to low-binding Eppendorf tubes (Eppendorf, #0030108116), and centrifuged again. The supernatant was again discarded, and the cell pellet was resuspended in 100 μL ice-cold PBS containing 0.4% NP-40 and protease and phosphatase inhibitors (Sigma Aldrich, #11873580001 and #04906837001, respectively) followed by four freeze–thaw cycles in liquid nitrogen. Finally, the samples were centrifuged at 16,000*g* for 15 min, and the supernatants were transferred to fresh low-binding Eppendorf tubes. The protein concentrations of the samples were determined using the DC protein assay kit (BioRad, #000112) according to the instructions of the manufacturer.

For each sample, dilutions of 200 μg protein in a total volume of 75 μL lysis buffer were prepared, and 75 μL of a 100 mM triethylammonium bicarbonate (TEAB) solution was added. Next, per sample, 7.5 μL of a 200 mM Tris(2-carboxyethyl)phosphine hydrochloride (TCEP) solution in 200 mM TEAB was added, and the samples were mixed properly by vortexing. Samples were collected by short centrifugation and incubated at 55 °C for 1 h in the dark. Afterward, 7.5 μL of a 375 mM solution of iodoacetamide in 200 mM TEAB buffer was added to each sample, and samples were incubated for 30 min at room temperature and in the dark. For protein precipitation, 900 μL ice-cold acetone (proteomics grade) per sample was added followed by overnight incubation at −20 °C. The samples were centrifuged for 10 min at 8000*g*, the supernatants were removed, and the pellets were dried for 45 min. For protein digestion, 107.5 mL of 0.4 mg/mL trypsin in 100 mM TEAB (HPLC grade) was added to each sample, and the samples were vortexed vigorously to dissolve the pellets completely. The digestion proceeded overnight at 37 °C and 300 rpm and in the absence of light. Afterward, samples were labeled with TMT labels (TMT10plex, Thermo Fisher Scientific #90110) according to the manufacturer’s instruction.

To reduce the complexity of the samples and thereby increase the number of quantified proteins, the samples were fractionated into 10 fractions using a C18 column and high pH conditions prior to nanoHPLC-MS/MS analysis. Briefly, the samples were dissolved in 120 μL of a 20 mM ammonium formate (HCOONH_4_) solution (pH 11.0) followed by ultrasonication (15 min), rotating (15 min), and centrifugation (8000*g*, 3 min, room temperature). For sample separations, 50 μL of the supernatants was injected onto an XBridge C18 column (130 A°, 3.5 mm, 1 × 150 mm) using a U3000 capHPLC system (Thermo Fisher Scientific) and a flow rate of 50 μL/min. Solvent compositions were as follows: solvent A: 20 mM HCOONH_4_ (pH 11) in H_2_O; solvent B: 40% 20 mM HCOONH_4_ (pH 11) in H_2_O premixed with 60% acetonitrile. Steps for sample separation were as follows: step 1, 10 t/min, 95% solvent A, 5% solvent B, isocratic; step 2, 5 t/min, linear gradient up to 25% solvent B; step 3, 60 t/min, linear gradient up to 65% solvent B; step 4, 10 t/min, linear gradient up to 100% solvent B; and step 5, 14 t/min, 100% solvent B.

For detection of the proteins, a wavelength of 214 nm was used, and the eluate between 15 and 100 min was fractionated into 10 fractions (30 s per fraction, circular fractionation using 10 vials). The collected fractions were completely dried in a SpeedVac at 30 °C and were then subjected to nanoHPLC-MS/MS analysis.

For nanoHPLC-MS/MS analysis, samples were dissolved in 20 μL 0.1% TFA in water (HPLC grade), and 3 μL of these solutions was injected into the UltiMate 3000 RSL Cnano system (Thermo Fisher Scientific) online coupled to a Q Exactive Plus Hybrid Quadrupole-Orbitrap Mass Spectrometer equipped with a nanospray source (Nanospray Flex Ion Source, Thermo Scientific). For desalting, the samples were injected onto a precolumn cartridge (5 μm, 100 A°, 300 mm ID × 5 mm, Dionex, Germany) using 0.1% TFA in water as eluent with a flow rate of 30 mL/min. Afterward, desalting was performed with eluent flow to waste followed by back-flushing of the sample during the whole analysis from the precolumn to the PepMap100 RSLC C18 nano-HPLC column (2 μm, 100 A°, 75 mm ID × 50 cm length, nanoViper, Dionex, Germany) using a flow rate of 300 nL/min and the steps indicated below. Solvent compositions were as follows: solvent A: 0.1% formic acid in H_2_O (HPLC-grade); solvent B: acetonitrile containing 0.1% formic acid (HPLC-grade). Steps for separation were as follows: step 1: 0 t/min, 95% solvent A, 5% solvent B; step 2: 120 t/min, linear gradient up to 40% solvent B; step 3: 5 t/min, linear gradient up to 60% solvent B; step 4: 5 t/min, linear gradient up to 95% solvent B; step 5: 5 t/min, 95% solvent B; step 6: 1 t/min, linear gradient back to starting conditions; and step 7: 14 t/min, re-equilibration to starting conditions.

The nanoHPLC was online coupled to a Quadrupole-Orbitrap Mass Spectrometer using a standard coated SilicaTip (ID 20 mm, Tip-ID 10 mM, New Objective, Woburn, MA, USA). The mass range of *m*/*z* 300 to 1650 was acquired with a resolution of 70,000 for a full scan followed by up to 10 high-energy collision dissociation (HCD) MS/MS scans of the most intense at least doubly charged ions using an NCE energy of 35% and a resolution of 35,000. The MaxQuant software^72^ including the Andromeda search algorithm was used for data analysis. The protein assignment was performed based on the murine reference proteome taken from the Uniprot database (UP000000589). The search was performed for full enzymatic trypsin cleavages allowing two missed cleavages. For protein modifications, oxidation of methionine and acetylation of the N-terminus were considered as variable modifications, and carbamidomethylation was chosen as fixed. For relative quantification, the type ″reporter ion MS2″ was chosen, and TMT labels were defined for all lysines and peptide N-termini. The mass accuracy for full mass spectra was set to 20 ppm (first search) and 4.5 ppm (second search), and that for MS/MS spectra was set to 20 ppm. The false discovery rates for peptide and protein identification were set to 1%. For further validations, only proteins for which at least two peptides were quantified were selected. Relative quantification of proteins was performed using the reporter ion MS2 algorithm implemented in MaxQuant and stored as a proteinGroups.txt file, which was used for further analysis. Proteins which were not identified with at least two razor and unique peptides in at least one biological replicate were filtered off. To identify differentially regulated proteins, the ″Reporter intensity corrected″ of the compound treated samples (1.5 μM purmorphamine and DMSO or 1.5 μM purmorphamine and 1 nM Picoberin) for each time point was divided by the ″Reporter intensity corrected″ of the corresponding vehicle control (DMSO or 1.5 μM purmorphamine and DMSO treated samples) of the respective time point, and the results were written into new columns. This file was stored under a different file name in txt format. For further data analysis, Perseus version 1.6.2.3^75^ was used. The calculated ratios of the above-mentioned file were defined as main columns and proteins resulting from the reverse database search, just identified by site, typical contaminants and not quantified in at least three out of three replicates were filtered off. Afterward, the ratios of the ″Reporter intensities corrected″ were logarithmized (log2) and normalized to the median. The mean of the replicates was calculated, and to assess the significances of the calculated log2 ratios, the outlier test ″Significance A″ was used. Only proteins with a *p* value < 0.05 were considered as statistically significantly up- or downregulated depending on the direction of change. Pathway-overrepresentation analysis was performed using the Ingenuity Pathway Analysis tool (IPA, Qiagen Version 70750971). Volcano plots were generated using the web-based tool VolcaNoseR.^74^

#### Immunostaining

For immunostaining of AhR in C3H10T1/2 cells, 10,0000 cells were seeded per well into 24-plates (Sarstedt, #83.3922) equipped with glass coverslips (Ø 12 mm, Thermo Fisher Scientific, #12-545-80) in 500 μL growth medium (DMEM, supplemented with 10% FCS and 1 mM sodium pyruvate) and incubated for 24 h at 37 °C and 5% CO_2_. The next day, cells were transiently transfected with a FLAG-mAhR plasmid (Origene, #MR227590) using the FuGENE HD transfection reagent (Promega, #E2311) and again incubated at 37 °C and 5% CO_2_. After 24 h, cells were treated with different concentrations of compounds or DMSO as a control. For this purpose, the compounds or DMSO was diluted by a dilution factor of 400 with a fresh growth medium to achieve a final DMSO concentration of 0.25% in all conditions. After incubation for 24 h, cells were fixed using 3.7% formaldehyde in PBS for 10 min followed by permeabilization using 0.3% Triton X-100 in PBS for 5 min. Afterward, cells were washed three times for 5 min with PBS followed by incubation with 3% BSA in PBS for 40 min to block unspecific binding sites. Next, the samples were incubated with primary antibodies detecting AhR (Santa Cruz, sc-133088, 1:200 dilution in 3% BSA) at 4 °C overnight. The next day, the primary antibody was discarded, and samples were washed three times for 5 min with PBS followed by incubation with an anti-mouse Alexa488 antibody (Invitrogen, #A21202, 1:1000 dilution in 3% BSA) and DAPI (Sigma Aldrich, #D9542) in the absence of light. After 1 h, samples were again washed three times with PBS for 5 min per well and were then mounted onto glass slides (Diagonal, #021102) using Aqua Polymount (Polysciences, #18606-20). Dried samples were imaged using the Zeiss Observer Z1 (Carl Zeiss, Germany) and a Plan-Apochromatic 63×/1.40 Oil DIC M27 objective.

#### Immunoblotting

For quantification of the AhR protein levels, 2 × 10^6^ HepG2 cells per well were seeded into six-well plates After incubation for 24 h at 37 °C and 5% CO_2_, cells were treated with different concentrations of compounds or DMSO as a control. After incubation for 4 h at 37 °C and 5% CO_2_, cells were washed with PBS followed by an incubation of the cells with cell dissociation solution (Gibco, #13151-014) for 10 min at 37 °C. Detached cells were resuspended and collected in 1.5 mL low-binding Eppendorf tubes (Eppendorf, #0030108116). Samples were then centrifuged for 3 min at 340*g*, and the pellets were washed with ice-cold PBS and suspended in a lysis buffer (150 mM sodium chloride, 1% NP-40, 0.5% sodium deoxycholate, and 50 mM Tris (pH 8.0)). Afterward, three freeze–thaw cycles using liquid nitrogen were performed. To remove cell debris, samples were centrifuged at 16,000*g* and 4 °C for 30 min. The supernatants were transferred to fresh low-binding Eppendorf tubes, and protein concentrations were determined using the DC protein assay according to the instructions of the manufacturer. Proteins were separated via an SDS-PAGE and were transferred onto a PVDF membrane using the wet blotting technique. Membranes were stained for AhR (Santa Cruz, #sc-133088, 1:500 dilution), lamin A (Santa Cruz, #sc-71481, 1:1000), and β-tubulin (Sigma Aldrich, #T5076). For protein visualization, secondary antibodies coupled to IR dye 800CW were employed and incubated with the membranes for 1 h at room temperature prior to the detection of band intensities using the Odyssey Fc Imaging System. Quantification of band intensities was performed using Image Studio Version 5.2.

#### Lentivirus Production and Transduction

Lentiviruses were produced using described TRC lentiviral proceedings (https://www.broadinstitute.org/genome_bio/trc/publicProtocols.html). Briefly, HEK293T cells were seeded into a 96-well plate at a density of 2 × 10^4^ cells/well. After 24 h, cells were transfected with the MISSION lentiviral packaging mix (Sigma-Aldrich) and 100 ng of luciferase lentiviral vectors using FuGENE HD (Promega). The lentiviral construct for the generation of AhR reporter cell lines was obtained from SABiosciences (http://www.sabiosciences.com/reporter_assay_product/HTML/CLS-9045L.html). Lentiviral constructs for constitutive expression of *Renilla*, pLenti.PGK.blast-*Renilla*_Luciferase, were provided by Reuben Shaw (Addgene plasmid #74444; http://n2t.net/addgene:74444; RRID: Addgene_74444).^75^

Lentiviral infection was performed according to protocols available at the RNAi Consortium website (https://www.broadinstitute.org/genome_bio/trc/publicProtocols.html). HaCaT cells were seeded in a 96-well plate at 2 × 10^4^ cells per well. NIH-3T3 cells were seeded in a 48-well plate at 2 × 10^4^ cells per well. Media containing lentivirus and 8 μg/mL polybrene (Sigma-Aldrich) were added to the wells, and cells were transduced for 1.5 h at 37 °C. Virus-containing media were subsequently replaced with plain media. After 48 h, transduced cells were selected for firefly luciferase and *Renilla* luciferase constructs using 5 μg/mL puromycin (ChemCruz) and 5 μg/mL blasticidin (Gibco), respectively.

#### AhR Reporter Gene Assays

For AhR reporter gene assays, HepG2 cells were transiently transfected with plasmids encoding for XRE-dependent Firefly luciferase (Promega, #9PIE412) and constitutively expressed *Renilla* luciferase (Promega, #E2241) for 24 h. Afterward, 50,000 cells were seeded per well into 96-well plates (Corning, #353075) and incubated for 5 h prior to compound treatment. For reporter gene assays with NIH/3T3 cells, HaCaT cells, and Hepa1c1c7 cells which were stably transfected with XRE-dependent Firefly luciferase and *Renilla* luciferase reporter constructs, 10,000 (NIH/3T3 cells) or 20,000 (HaCaT, Hepa1c1c7, or THP1 cells) cells were seeded per well into 96-well plates and incubated for 24 h prior to compound treatment. AhR reporter cell lines were treated with specified concentrations of ligands for either 4 or 24 h as described in the figure legends. Luciferase activities were determined using the Dual-Glo Luciferase Assay System (Promega, #E2940) for THP-1 and Hepa1c1c7 cells according to the manufacturer’s instructions. For NIH-3T3 cells, HepG2 cells, and HaCaT cells, the Dual-Luciferase Assay System (Promega, #E1960) was used according to the manufacturer’s instructions. Values obtained for firefly luciferase were normalized to the corresponding *Renilla* luciferase values. Results are shown as fold induction determined upon normalization to the luciferase values of the respective control.^76^ All values were related to values obtained for samples treated with DMSO (set to 1).

#### Genetic Knockdown of AhR

For AhR knockdown experiments, 600,000 C3H10T1/2 cells were seeded in T25 cell culture flasks (Sarstedt, #83.3910.002) and incubated for 24 h at 37 °C and 5% CO_2_. Afterward, cells were bulk-transfected with 30 nM AhR siRNA (Dharmacon, 1:1 mixture of #J-044066-06-0005 and #J-044066-07-0005) or control siRNA (Dharmacon, # D-001810-01-05) using the DharmaFECT1 transfection reagent (Dharmacon, #T-2001-02) according to the instructions of the manufacturer. After 24 h of incubation at 37 °C and 5% CO_2_, transfected cells were trypsinized and seeded in 96-well plates (6000 cells/well) for osteoblast differentiation assays or in 12-well plates (60,000 cells/well) for RT-qPCR analysis. Cells were incubated for 5 h at 37 °C and 5% CO_2_ until they were attached to the bottom of the wells and were then treated with 1.5 μM purmorphamine in the presence of DMSO or the compounds for 48 or 96 h.

#### Software and Algorithms

The following software and algorithms were used: CLC Workbench (Version 12.0 and CLC Genomics Server version 11.0) (Qiagen) RRID: SCR_011853, https://digitalinsights.qiagen.com/products-overview/discovery-insights-portfolio/analysis-and-visualization/qiagen-clc-main-workbench/; CFX Manager (Bio-Rad) RRID: SCR_003375, http://www.bioconductor.org/packages/release/bioc/html/HTqPCR.html; Prism 7 (GraphPad) RRID:SCR_002798, https://www.graphpad.com/; IncuCyte ZOOM 2015/2016A (EssenBioscience, Satorius) https://www.sartorius.com/en/products/live-cell-imaging-analysis/live-cell-analysis-software; VolcaNose R, (Joachim Goedhart^74^), https://huygens.science.uva.nl/VolcaNoseR/; Perseus version 1.6.2.3,^73^ https://maxquant.net/perseus/, http://coxdocs.org/doku.php?id=perseus:start; Ingenuity Pathway Analysis (Version 70750971) (Qiagen) RRID: SCR_008653; and Schrodinger suite, release 2021-1 (Schrödinger Inc., USA).

#### Statistical Analysis

If not stated differently, three biological replicates (*n* = 3) were performed for each experiment, with three technical replicates (*N* = 3) per biological replicate. Statistical analysis was performed for at least three biological replicates via an unpaired *t* test using the software GraphPad Prism 7 (GraphPad Software, USA). Only data that resulted in *p* values < 0.05 were considered to be significant.

#### Data Availability

The generated NGS data sets are available at GEO: GSE197970. The generated proteomics data sets are available at MassIVE: MSV000089308. The X-ray structure of compound **3l** is available at CCDC: 2152125.

#### Computational Analysis

##### Protein Models

The AlphaFold AhR PAS-B model was obtained from the AlphaFold Protein Structure Database developed by DeepMind and EMBL-EBI (model AF-P30561-F1 version 1 for murine AhR) (https://alphafold.ebi.ac.uk/entry/P30561). The model in the database was generated using AlphaFold v2.0.^49^ The homology model was afforded from the Swiss-Model homology-modeling server repository of the Swiss Institute of Bioinformatics and the Biozentrum of the University of Basel,^50^ where the structure of murine AhR was produced based on the X-ray structure of the CLOCK protein (PDB ID: 4F3L).^71^ Both PAS-B models were based on the same amino acid sequence reported for the UniProt entry P30561. The structural quality of the predicted AhR PAS-B domains, corresponding to the amino acid residues 276–380, was evaluated using ProSA, QMEAN, and PROCHECK programs.^52^ The models were also cross-validated against X-ray structures of HIF2α, as well as structures of AhR PAS-B (7VNA, 7VNH, and 7VNI) for *Drosophila melanogaster*, calculating the RMSD values between the predictions and the experimental constructs using PyMOL (Schrödinger Inc., USA).

##### Molecular Docking

Computational docking and simulations were performed using the Maestro environment, version 12.7, and the Schrodinger suite of software, release 2021-1 (Schrödinger Inc., USA). The protein structures were prepared for docking using the Protein Preparation Wizard (Schrödinger), refining the amino acid protonation states with PROPKA at pH set to 7.0 and applying restrained minimization with an OPLS4 force field, where the heavy atom convergence was set to 0.3 Å RMSD.^77^ Ligand preparation was performed with LigPrep (Schrödinger) using the OPLS4 force field and Epik (v5.5) ionization at pH 7.0 ± 2.0.^78^ Ligands were docked using the *ab initio* induced-fit docking (IFD; Schrödinger) strategy.^79^ The docking grid was centered inside the AhR PAS-B ligand-binding pocket, the Glide (v9.0) docking was set to XP (extra precision), and the residue refinement with Prime (v6.3) was set to 5 Å within the ligand poses with side chain optimization.^80^ Redocking into structures was performed within 30 kcal/mol of the best structure and within the top 20 structures overall. The afforded ligand poses from IFD were further scored using Prime MM-GBSA, where the solvation model was set to VSGB,^81^ the force field was set to OPLS4, protein residue flexibility was allowed within 5 Å of the ligand, and the sampling method was set to minimization. The pose with the best MM-GBSA energy for each ligand–protein complex was used further in the molecular dynamics (MD) simulations.

##### Molecular Dynamics Simulations

The MD calculations were performed with Desmond (v6.5; D.E. Shaw Research, USA; Schrödinger)^82^ using an NVIDIA GeForce RTX 2070 GPU. System Builder (Schrödinger) was used to prepare the protein–ligand complex for the simulations. The TIP3P solvent model was applied,^83^ with an orthorhombic box shape, a buffer padding of 12 Å, and the OPLS4 force field. Charges were neutralized with Cl^–^ ions, and NaCl was added at a concentration of 0.15 M to mimic the physiological conditions. The simulation time was set to 100 ns, with recording intervals of 25 ps for the trajectories and 1.2 ps for the energy. The NPT ensemble was chosen, with the temperature set to 300 K and the pressure to 1.01325 bar. The RESPA integrator step was 2 fs, and a Nose–Hoover chain thermostat (relaxation time 1.0 ps) and a Martyna–Tobias–Klein barostat (relaxation time 2.0 ps) were used. The short-range Coulombic interaction cutoff radius was set to 9 Å. The system was relaxed with Maestro’s build-in relaxation protocol before the commencement of the production run. The obtained results were analyzed using the Simulation Interactions Diagram tools (Schrödinger).

##### X-ray Analysis of **3m**

The crystal structure of compound **3l** was determined using the Bruker D8 Venture four-circle diffractometer equipped with a PHOTON II CPAD detector by Bruker AXS GmbH. The X-ray radiation was generated by the IμS/IμS microfocus source Cu (λ = 1.54178 Å) from Incoatec GmbH equipped with HELIOS mirror optics and a single-hole collimator by Bruker AXS GmbH. The selected single crystal of **3l** was covered with an inert oil (perfluoropolyalkyl ether) and mounted on the MicroMount from MiTeGen. The APEX 3 Suite (v.2018.7-2) software integrated with SAINT (integration) and SADABS (adsorption correction) programs by Bruker AXS GmbH was used for data collection. The processing and finalization of the crystal structure were performed using the Olex2 program.^84^ The crystal structures were solved by the ShelXT^85^ structure solution program using the Intrinsic Phasing option, which were further refined by the ShelXL^86^ refinement package using least squares minimization. The non-hydrogen atoms were anisotropically refined. The C-bound H atoms were placed in geometrically calculated positions, and a fixed isotropic displacement parameter was assigned to each atom according to the riding-model: C–H = 0.95–1.00 Å with *U*iso (H) = 1.2*U*eq (CH_2_, CH) for other hydrogen atoms. The crystallographic data for the structure of **3l** have been published as supplementary publication number 2152125 in the Cambridge Crystallographic Data Centre. A copy of these data can be obtained for free by applying to CCDC, 12 Union Road, Cambridge CB2 IEZ, UK, fax: 144-(0)1223-336033 or e-mail: deposit@ccdc.cam.ac.uk.

#### Pan-assay Interference

Picoberin and its derivatives have been screened in several cell-based assays and displayed activity only in the Hh-mediated osteogenesis assay. All compounds used in this study do not contain PAINS^87^ (determined using PAINS filters;^88,89^ PAINS filters were implemented with Pipeline Pilot (BioVia)). Picoberin has picomolar potency and is not predicted to act as an aggregator using the Aggregator Advisor^90^ (http://advisor.bkslab.org/). Picoberin was assayed against 168 GPCRs and 19 nuclear and inhibited only one GPCR >50%, thus ruling out general interference with these assays. The purity of Picoberin was determined to be >95% (98%). Picoberin did not directly inhibit alkaline phosphatase (AP) activity (AP was used as a reporter protein of osteogenesis and, thus, active Hedgehog signaling). Moreover, Picoberin did not affect the enzymatic activity of firefly and *Renilla* luciferase.

### Chemistry

#### Chemical Synthesis: General Information

Solvents for chromatography were technical grade. Analytical thin-layer chromatography (TLC) was performed on Merck silica gel aluminum plates with an F-_254_ indicator. Compounds were visualized by irradiation with UV light or potassium permanganate staining. Column chromatography was performed using silica gel Merck 60 (particle size 0.040–0.063 mm). Purity was determined by ^1^H NMR analysis and chiral HPLC, and all presented compounds are ≥95% pure.

^1^H NMR and ^13^C NMR were recorded on a Bruker DRX400 (400 MHz), Bruker DRX500 (500 MHz), INOVA500 (500 MHz), and Bruker DRX700 using CDCl_3_ as solvent. Data are reported in the following order: chemical shift (δ) values are reported in ppm with the solvent resonance as internal standard (CDCl_3_: δ = 7.26 ppm for ^1^H, δ = 77.16 ppm for ^13^C). Multiplicities are indicated as br s (broadened singlet), s (singlet), d (doublet), t (triplet), q (quartet), and m (multiplet); coupling constants (J) are given in hertz (Hz).

High-resolution mass spectra were recorded on an LTQ Orbitrap mass spectrometer coupled to an Accela HPLC-System (HPLC column: Hypersyl GOLD, 50 × 1 mm, particle size 1.9 μm, ionization method: electron spray ionization). Fourier transform infrared spectroscopy (FT-IR) spectra were obtained with a Bruker Tensor 27 spectrometer (ATR, neat) and are reported in terms of frequency of absorption (cm^–1^). Optical rotations were measured in a Schmidt + Haensch Polartronic HH8 polarimeter.

The enantiomeric excesses were determined by HPLC analysis using a chiral stationary phase column (CHIRALCEL IC, CHIRALCEL IA; eluent: (DCM/EtOH = 100/2)/*iso*-hexane, *i-*PrOH/*iso*-hexane; 4.6 × 250 mm, particle size 5 μm). The chiral HPLC methods were calibrated with the corresponding racemic mixtures. The ratio of regioisomers was determined by ^1^H NMR analysis via the integration of characteristic signals. Chemical yields refer to isolated substances. Yields and enantiomeric excesses are given in the tables.

Rhodium catalyst **Rh1**, aryl hydroxymates **1**, and styrenes **2** were synthesized according to previously reported procedures.^19,23^

#### General Procedure for the Synthesis of 8-Oxotetrahydroprotoberberines **3**

Without protection from air and moisture, catalyst **Rh1** (2.53 mg, 5.00 μmol, 0.05 equiv), dibenzoylperoxide (75 wt %, 1.62 mg, 5.00 μmol, 0.05 equiv), and hydroxamates **1** (0.10 mmol, 1 equiv) were dissolved in 200 μL of DCM. The mixture was stirred at rt for 2 min. Subsequently, the corresponding styrene **2** (0.20 mmol, 2.00 equiv) was added, and the reaction mixture was stirred at room temperature for 48 h. Then, 2 mL of THF was added, and the mixture was cooled to 0 °C. A solution of *t-*BuOK (2 equiv, 0.20 mmol) in THF was added dropwise, and the reaction mixture was stirred for 15 min. The mixture was quenched with sat. NaHCO_3_ solution. The organic layers were dried over Na_2_SO_4_, and the solvent was evaporated under a vacuum. The crude was purified on a silica gel column using a mix of pentane/EtOAc (typically, 5:1) to afford desired products **3**.
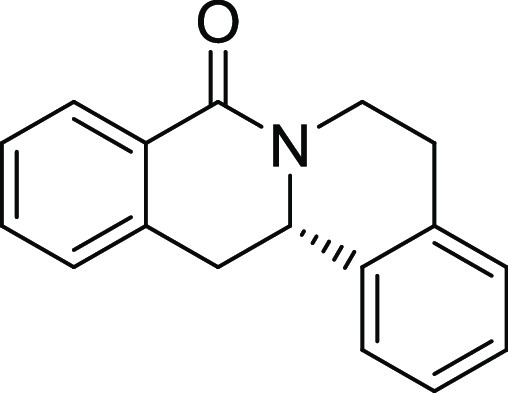


##### (*S*)-5,6,13,13a-Tetrahydro-8*H*-isoquinolino[3,2-*a*]isoquinolin-8-one (**3a**)

76% yield, 97% ee; **^1^H NMR (400 MHz, CDCl_3_):** δ 8.15 (dd, *J* = 7.5, 1.5 Hz, 1H), 7.47 (td, *J* = 7.5, 1.5 Hz, 1H), 7.39 (t, *J* = 7.5 Hz, 1H), 7.30–7.20 (m, 5H), 5.03–4.90 (m, 2H), 3.26 (dd, *J* = 15.7, 3.7 Hz, 1H), 3.08–2.96 (m, 3H), 2.93–2.82 ppm (m, 1H); **^13^C NMR (101 MHz, CDCl_3_):** δ 164.7, 137.4, 136.0, 135.2, 131.9, 129.2, 129.1, 128.7, 127.4, 127.0, 126.9, 126.8, 126.0, 55.3, 38.8, 38.0, 29.8 ppm; **FT-IR:** ν̃ = 2933, 2886, 1637, 1600, 1579, 1463, 1402, 1363, 1287, 1149 cm^–1^; **HRMS:** calc. for [M + H]^+^ C_17_H_16_NO: 250.12264, found: 250.12292; [α]_*D*_^*RT*^= −440.4 (CH_2_Cl_2_, *c* = 2.00); **HPLC conditions**: CHIRAPAK IC column, (CH_2_Cl_2_/EtOH = 100/2)/*iso*-hexane = 50/50, flow rate = 0.5 mL min^–1^, major enantiomer: *t*_R_ = 32.6 min; minor enantiomer: *t*_R_ = 31.3 min.
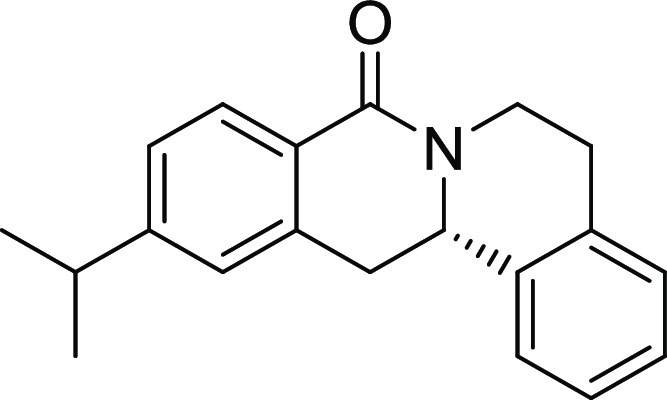


##### (*S*)-11-Isopropyl-5,6,13,13a-tetrahydro-8*H*-isoquinolino[3,2-*a*]isoquinolin-8-one (**3b**)

61% yield, 95% ee; **^1^H NMR (500 MHz, CDCl_3_):** δ = 7.99 (d, *J* = 7.9 Hz, 1H), 7.26–7.11 (m, 5H), 7.03 (s, 1H), 4.98–4.84 (m, 2H), 3.16 (dd, *J* = 15.6, 3.7 Hz, 1H), 3.00–2.84 (m, 4H), 2.86–2.74 (m, 1H), 1.21 (dd, *J* = 6.9, 2.4 Hz, 6H); **^13^C NMR (126 MHz, CDCl_3_):** δ = 164.79, 153.21, 137.44, 136.13, 135.17, 129.03, 128.76, 126.85, 126.83, 126.73, 126.01, 125.65, 124.90, 55.34, 38.60, 38.12, 34.25, 29.80, 23.83, 23.80 ppm; **FT-IR:** ν̃ = 3060, 2905, 1570, 1313, 1201, 1121, 933, 893 cm^–1^; **HRMS**: calc. for [M + H]^+^ C_20_H_22_NO: 292.1701, found: 292.1705; [α]_*D*_^*RT*^= −181.9 (CH_2_Cl_2_, *c* = 0.50); **HPLC conditions:** CHIRAPAK IC column, *iso*-propanol/*iso*-hexane = 30/70, flow rate = 0.5 mL min^–1^, major enantiomer: *t*_R_ = 39.8 min; minor enantiomer: *t*_R_ = 35.1 min.
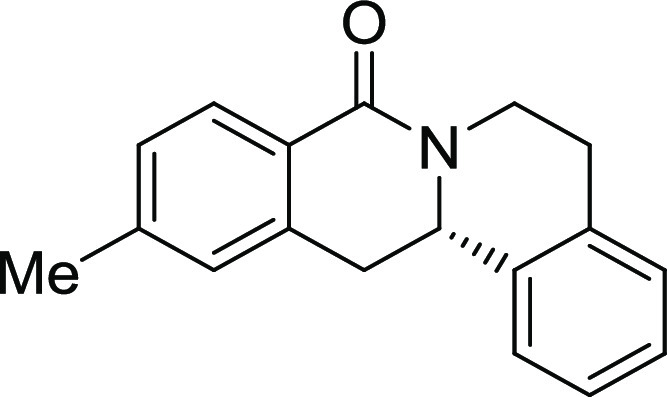


##### (*S*)-11-Methyl-5,6,13,13a-tetrahydro-8*H*-isoquinolino[3,2-*a*]isoquinolin-8-one (**3c**)

84% yield, 91% ee; **^1^H NMR (500 MHz, CDCl_3_):** δ = 8.03 (d, *J* = 7.9 Hz, 1H), 7.34–7.15 (m, 5H), 7.07 (s, 1H), 5.01–4.94 (m, 1H), 4.92 (dd, *J* = 13.5, 3.6 Hz, 1H), 3.20 (dd, *J* = 15.7, 3.7 Hz, 1H), 3.07–2.92 (m, 3H), 2.87 (dd, *J* = 12.4, 2.4 Hz, 1H), 2.40 ppm (s, 3H); **^13^C NMR (126 MHz, CDCl_3_):** δ = 164.9, 142.4, 137.4, 136.1, 135.25, 129.1, 128.7, 128.3, 127.6, 126.9, 126.8, 126.5, 126.1, 55.4, 38.7, 38.0, 29.8, 21.7 ppm; **FT-IR:** ν̃ = 3061, 2932, 1628, 1612, 1577, 1429, 1401, 1300, 1206 cm^–1^; **HRMS:** calc. for [M + H]^+^ C_18_H_18_NO: 264.13829, found: 264.13825; [α]_*D*_^*RT*^= −323.7 (CH_2_Cl_2_, *c* = 1.00); **HPLC conditions:** CHIRAPAK IC column, (CH_2_Cl_2_/EtOH = 100/2)/*iso*-hexane = 50/50, flow rate = 0.5 mL min^–1^, major enantiomer: *t*_R_ = 34.1 min; minor enantiomer: *t*_R_ = 30.7 min.
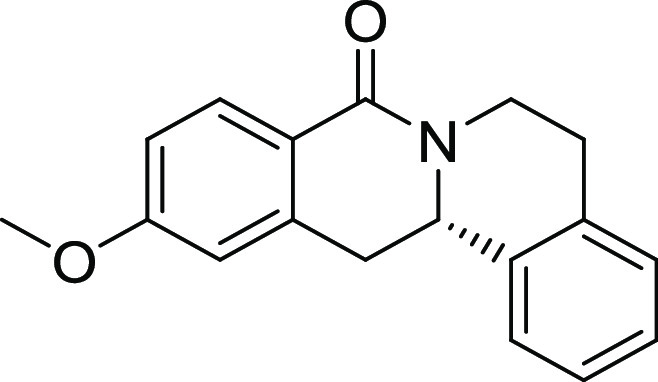


##### (*S*)-11-Methoxyl-5,6,13,13a-tetrahydro-8*H*-isoquinolino[3,2-*a*]isoquinolin-8-one (**3d**)

61% yield, 97% ee; **^1^H NMR (500 MHz, CDCl_3_)** δ 8.09 (d, *J* = 8.6 Hz, 1H), 7.31–7.20 (m, 4H), 6.90 (dd, *J* = 8.6, 2.1 Hz, 1H), 6.74 (d, *J* = 1.7 Hz, 1H), 5.00–4.95 (m, 1H), 4.92 (dd, *J* = 13.4, 3.5 Hz, 1H), 3.87 (s, 3H), 3.20 (dd, *J* = 15.7, 3.7 Hz, 1H), 3.03–2.94 (m, 3H), 2.89–2.83 ppm (m, 1H); **^13^C NMR (126 MHz, CDCl_3_):** δ = 164.8, 162.4, 139.5, 136.0, 135.2, 130.8, 129.1, 126.9, 126.8, 126.0, 122.0, 112.9, 112.0, 55.5, 55.3, 38.6, 38.3, 29.8 ppm; **FT-IR:** ν̃ = 2962, 2929, 1639, 1605, 1493, 1409, 1366, 1275 cm^–1^; **HRMS:** calc. for [M + H]^+^ C_18_H_18_NO_2_: 280.13321, found: 280.13311; [α]_*D*_^*RT*^= −287.1 (CH_2_Cl_2_, *c* = 1.00); **HPLC conditions:** CHIRAPAK IC column, *iso*-propanol/*iso*-hexane = 50/50, flow rate = 0.5 mL min^–1^, major enantiomer: *t*_R_ = 41.7 min; minor enantiomer: *t*_R_ = 39.5 min.
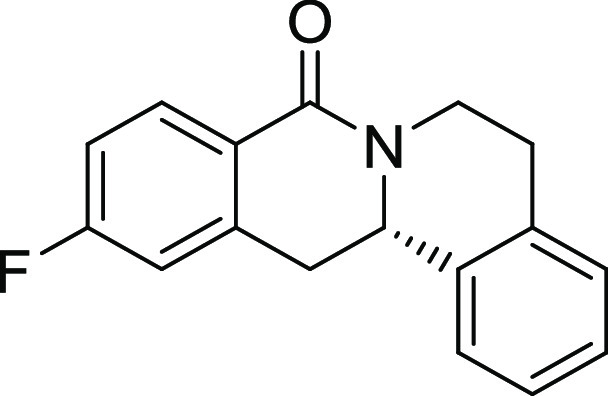


##### (*S*)-11-Fluoro-5,6,13,13a-tetrahydro-8*H*-isoquinolino[3,2-*a*]isoquinolin-8-one (**3e**)

45% yield, 97% ee; **^1^H NMR (700 MHz, CDCl_3_):** δ = 8.15 (dd, *J* = 8.6, 5.8 Hz, 1H), 7.32–7.28 (m, 1H), 7.27–7.21 (m, 3H), 7.10–7.04 (m, 1H), 6.98–6.94 (m, 1H), 5.00–4.90 (m, 2H), 3.23 (dd, *J* = 15.8, 3.7 Hz, 1H), 3.06–2.96 (m, 3H), 2.93–2.83 ppm (m, 1H); **^13^C NMR (176 MHz, CDCl_3_):** δ = 164.8 (d, *J* = 252.8 Hz, 1C), 163.9, 140.2 (d, *J* = 8.8 Hz, 1C), 135.7, 135.2, 131.5 (d, *J* = 9.4 Hz, 1C), 129.2, 127.1, 126.9, 126.0, 125.5 (d, *J* = 2.7 Hz, 1C), 114.7 (d, *J* = 21.7 Hz, 1C), 113.8 (d, *J* = 22.0 Hz, 1C), 55.2, 38.8, 37.9, 37.9, 29.8 ppm; **FT-IR:** ν̃ = 3058, 2928, 2864, 1607, 1587, 1394, 1201, 1144, 974 cm^–1^; **HRMS:** calc. for [M + H]^+^ C_17_H_15_FNO: 268,11,322, found: 268.11318; [α]_*D*_^*RT*^= −367.7(CH_2_Cl_2_, *c* = 1.00); **HPLC conditions:** CHIRAPAK IC column, *iso*-propanol/*iso*-hexane = 50/50, flow rate = 0.5 mL min^–1^, major enantiomer: *t*_R_ = 23.6 min; minor enantiomer: *t*_R_ = 19.9 min.
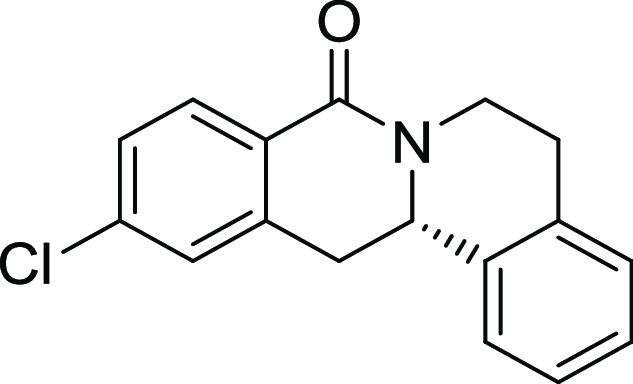


##### (*S*)-11-Chlorol-5,6,13,13a-tetrahydro-8*H*-isoquinolino[3,2-*a*]isoquinolin-8-one (**3f**)

49% yield, 98% ee; **^1^H NMR (500 MHz, CDCl_3_):** δ = 8.07 (d, *J* = 8.3 Hz, 1H), 7.36 (ddd, *J* = 8.3, 1.9, 0.7 Hz, 1H), 7.32–7.28 (m, 1H), 7.25 (dd, *J* = 10.0, 2.3 Hz, 4H), 4.95 (ddd, *J* = 13.8, 9.6, 3.4 Hz, 2H), 3.22 (dd, *J* = 15.8, 3.7 Hz, 1H), 3.07–2.95 (m, 3H), 2.92–2.81 ppm (m, 1H); **^13^C NMR (126 MHz, CDCl_3_):** δ = 163.9, 139.1, 137.9, 135.5, 135.1, 130.3, 129.2, 127.8, 127.6, 127.1, 127.1, 126.9, 126.0, 55.1, 38.8, 37.7, 29.7 ppm; **FT-IR:** ν̃ = 3211, 2926, 2516, 1629, 1454, 1363, 1326 cm^–1^; **HRMS:** calc. for [M + H]^+^ C_17_H_15_ClNO:284.08367, found: 284.08369; [α]_*D*_^*RT*^= −149.4 (CH_2_Cl_2_, *c* = 0.50); **HPLC conditions:** CHIRAPAK IC column, (CH_2_Cl_2_/EtOH = 100/2)/*iso*-hexane = 30/70, flow rate = 0.5 mL min^–1^, major enantiomer: *t*_R_ = 47.8 min; minor enantiomer: *t*_R_ = 50.1 min.
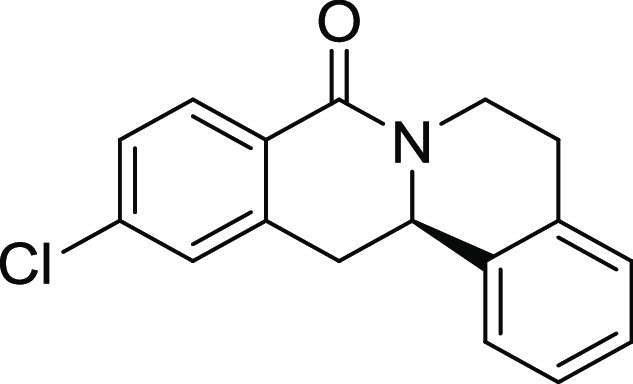


##### (*R*)-11-Chlorol-5,6,13,13a-tetrahydro-8*H*-isoquinolino[3,2-*a*]isoquinolin-8-one (**3f′**)

54% yield, −91% ee (the slightly decreased ee is caused by the less enantiopure catalyst); **HPLC conditions:** CHIRAPAK IC column, (CH_2_Cl_2_/EtOH = 100/2)/*iso*-hexane = 30/70, flow rate = 0.5 mL min^–1^, major enantiomer: *t*_R_ = 48.0 min; minor enantiomer: *t*_R_ = 50.2 min.
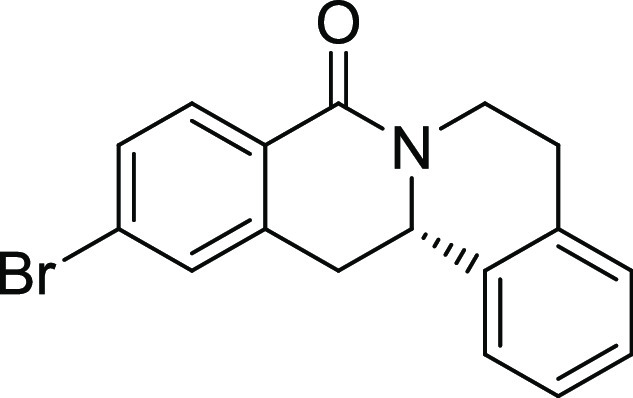


##### (*S*)-11-Bromo-5,6,13,13a-tetrahydro-8*H*-isoquinolino[3,2-*a*]isoquinolin-8-one (**3g**)

53% yield, 97% ee; **^1^H NMR (500 MHz, CDCl_3_):** δ = 8 7.93 (d, *J* = 8.3 Hz, 1H), 7.46 (ddd, *J* = 8.3, 2.1, 1.0 Hz, 1H), 7.37 (t, *J* = 1.6 Hz, 1H), 7.27–7.13 (m, 4H), 4.96–4.84 (m, 2H), 3.15 (dd, *J* = 15.8, 3.7 Hz, 1H), 3.01–2.88 (m, 3H), 2.88–2.76 (m, 1H); **^13^C NMR (126 MHz, CDCl_3_):** δ = 163.94, 139.18, 135.46, 135.02, 130.72, 130.39, 129.94, 129.11, 128.00, 127.06, 126.89, 126.48, 125.93, 55.09, 38.75, 37.56, 29.64 ppm; **FT-IR:** ν̃ = 3053, 2921, 2855, 1604, 1576, 1403, 1199, 1101, 970 cm^–1^; **HRMS**: calc. for [M + H]^+^ C_17_H_15_BrNO: 328.0337 and 330.0317, found: 328.0335 and 330.0312; [α]_*D*_^*RT*^= −169.1 (CH_2_Cl_2_, *c* = 0.50); **HPLC conditions:** CHIRAPAK IC column, *iso*-propanol/*iso*-hexane = 50/50, flow rate = 0.5 mL min^–1^, major enantiomer: *t*_R_ = 27.2 min; minor enantiomer: *t*_R_ = 22.6 min.
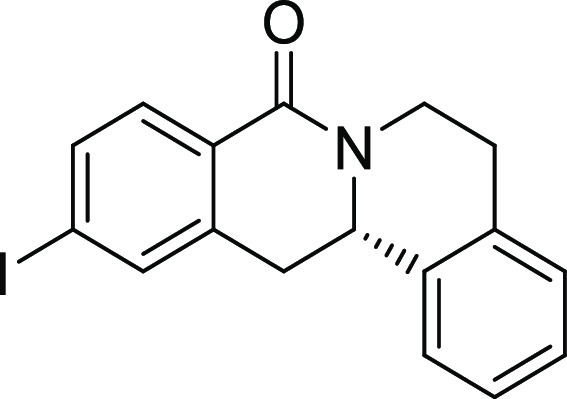


##### (*S*)-11-Iodo-5,6,13,13a-tetrahydro-8*H*-isoquinolino[3,2-*a*]isoquinolin-8-one (**3h**)

49% yield, 98% ee; **^1^H NMR (500 MHz, CDCl_3_):** δ = 7.78 (d, *J* = 8.2 Hz, 1H), 7.68 (ddd, *J* = 8.1, 1.7, 0.9 Hz, 1H), 7.59 (t, *J* = 1.4 Hz, 1H), 7.22 (qd, *J* = 7.3, 1.7 Hz, 1H), 7.17 (ddt, *J* = 8.6, 5.2, 2.3 Hz, 3H), 4.94–4.83 (m, 2H), 3.12 (dd, *J* = 15.8, 3.7 Hz, 1H), 3.00–2.89 (m, 2H), 2.85–2.77 (m, 1H); **^13^C NMR (126 MHz, CDCl_3_):** δ = 139.13, 136.72, 135.91, 135.46, 135.02, 130.24, 129.11, 127.05, 126.89, 125.93, 99.09, 55.07, 38.74, 37.37, 29.64 ppm; **FT-IR:** ν̃ = 3067, 2915, 2799, 1586, 1314, 1151, 1141, 960 cm^–1^; **HRMS**: calc. for [M + H]^+^ C_17_H_15_INO: 376.0198, found: 376.0198; [α]_*D*_^*RT*^= −101.9 (CH_2_Cl_2_, *c* = 0.50); **HPLC conditions:** CHIRAPAK IC column, *iso*-propanol/*iso*-hexane = 50/50, flow rate = 0.5 mL min^–1^, major enantiomer: *t*_R_ = 28.6 min; minor enantiomer: *t*_R_ = 23.7 min.
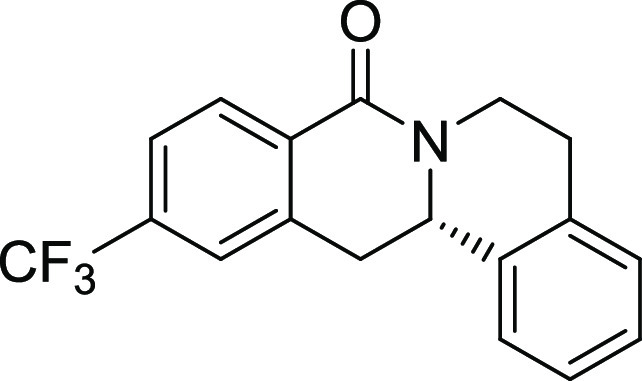


##### (*S*)-11-(Trifluoromethyl)-5,6,13,13a-tetrahydro-8*H*-isoquinolino[3,2-*a*]isoquinolin-8-one (**3i**)

61% yield, 96% ee; **^1^H NMR (500 MHz, CDCl_3_):** δ = 8.19 (d, *J* = 8.1 Hz, 1H), 7.61–7.56 (m, 1H), 7.47 (s, 1H), 7.28–7.15 (m, 4H), 4.97–4.86 (m, 2H), 3.26 (dd, *J* = 15.9, 3.7 Hz, 1H), 3.04–2.92 (m, 3H), 2.88–2.80 (m, 1H); **^13^C NMR (126 MHz, CDCl_3_):** δ = 163.36, 137.96 (d, *J*CF = 257.1 Hz), 135.27, 134.95, 132.10, 129.26 (d, *J*CF = 32.5 Hz), 129.14, 127.16, 126.97, 125.92, 124.26, 124.23, 124.06, 124.03, 55.02, 38.89, 37.70, 29.60 ppm; **^19^F NMR (470 MHz, CDCl_3_):** δ −62.90 ppm; **HRMS**: calc. for [M + H]^+^ C_18_H_15_F_3_NO: 318.1106, found: 318.1101; [α]_*D*_^*RT*^= −41.4 (CH_2_Cl_2_, *c* = 0.50); **HPLC conditions:** CHIRAPAK IC column, *iso*-propanol/*iso*-hexane = 30/70, flow rate = 0.5 mL min^–1^, major enantiomer: *t*_R_ = 17.8 min; minor enantiomer: *t*_R_ = 21.4 min.
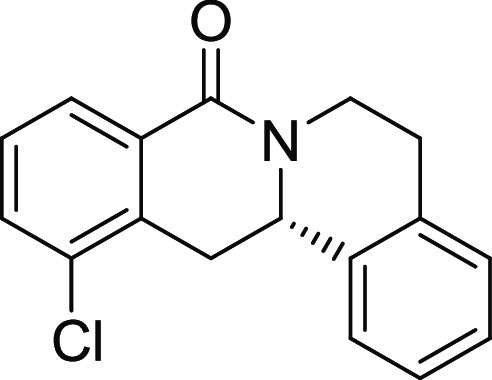


##### (*S*)-12-Chloro-5,6,13,13a-tetrahydro-8*H*-isoquinolino[3,2-*a*]isoquinolin-8-one (**3j**)

64% yield, 91% ee; **^1^H NMR (500 MHz, CDCl_3_):** δ = 8.02 (dd, *J* = 7.8, 1.2 Hz, 1H), 7.46 (dd, *J* = 8.0, 1.3 Hz, 1H), 7.31–7.23 (m, 3H), 7.26–7.17 (m, 1H), 7.20–7.14 (m, 1H), 4.94–4.83 (m, 2H), 3.59 (dd, *J* = 16.4, 3.9 Hz, 1H), 3.02–2.91 (m, 2H), 2.87–2.77 (m, 1H), 2.79–2.70 (m, 1H); **^13^C NMR (126 MHz, CDCl_3_):** δ = 135.49, 132.43, 129.07, 128.07, 127.31, 127.09, 126.93, 126.11, 54.49, 38.87, 34.80, 29.58 ppm; **FT-IR:** ν̃ = 3055, 2917, 1575, 1355, 1218, 1141, 1027, 930, 893 cm^–1^; **HRMS**: calc. for [M + H]^+^ C_17_H_15_NOCl: 284.0842, found: 284.0839; [α]_*D*_^*RT*^= −211.7 (CH_2_Cl_2_, *c* = 0.50); **HPLC conditions:** CHIRAPAK IC column, *iso*-propanol/*iso*-hexane = 30/70, flow rate = 0.5 mL min^–1^, major enantiomer: *t*_R_ = 37.6 min; minor enantiomer: *t*_R_ = 29.0 min.
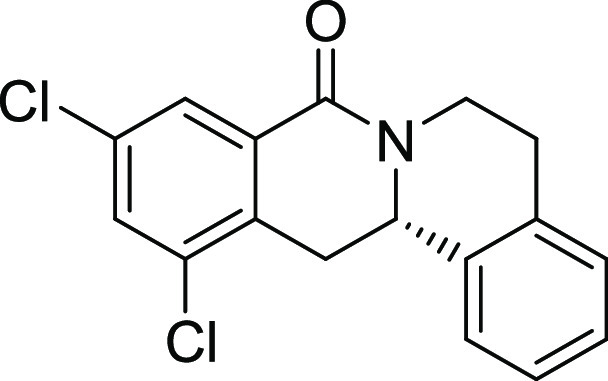


##### (*S*)-10,12-Dichloro-5,6,13,13a-tetrahydro-8*H*-isoquinolino[3,2-*a*]isoquinolin-8-one (**3k**)

55% yield, 90% ee; **^1^H NMR (500 MHz, CDCl_3_):** δ = 8.07 (d, *J* = 2.2 Hz, 1H), 7.53 (d, *J* = 2.2 Hz, 1H), 7.36–7.20 (m, 4H), 4.95–4.85 (m, 2H), 3.60 (dd, *J* = 16.5, 3.9 Hz, 1H), 3.11–2.96 (m, 2H), 2.95–2.84 (m, 1H), 2.76 (dd, *J* = 16.5, 13.4 Hz, 1H); **^13^C NMR (126 MHz, CDCl_3_):** δ = 135.10, 134.87, 133.70, 133.63, 133.03, 132.04, 131.92, 129.12, 127.47, 127.23, 127.02, 126.04, 53.47, 39.07, 34.37, 29.48 ppm; **FT-IR:** ν̃ = 3201, 2920, 2519, 1628, 1454, 1323, 1116, 1031, 923 cm^–1^; **HRMS**: calc. for [M + H]^+^ C_17_H_14_Cl_2_NO: 318.0452, found: 318.0448; [α]_*D*_^*RT*^= −235.4 (CH_2_Cl_2_, *c* = 0.50); **HPLC conditions:** CHIRAPAK IC column, *iso*-propanol/*iso*-hexane = 30/70, flow rate = 0.5 mL min^–1^, major enantiomer: *t*_R_ = 36.8 min; minor enantiomer: *t*_R_ = 25.7 min.
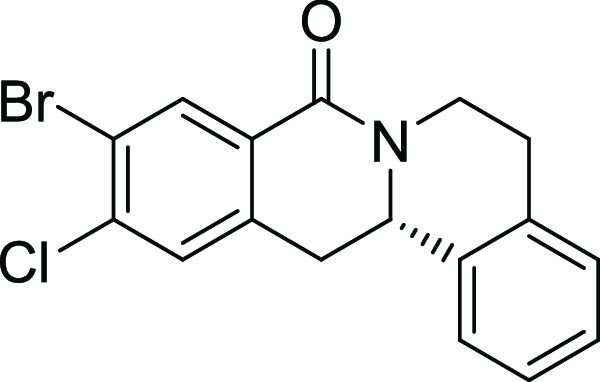


53% overall yield (5.2:1 r.r.).

##### Minor Isomer: (*S*)-10-Bromo-11-chloro-5,6,13,13a-tetrahydro-8*H*-isoquinolino[3,2-*a*]isoquinolin-8-one (**3l**)

96% ee; **^1^H NMR (500 MHz, CDCl_3_):** δ = 8.29 (s, 1H), 7.30 (s, 1H), 7.24–7.20 (m, 2H), 7.17–7.15 (m, 3H), 4.89 (ddd, *J* = 13.8, 9.6, 3.4 Hz, 2H), 3.12 (dd, *J* = 15.8, 3.7 Hz, 1H), 2.98–2.79 (m, 4H); **^13^C NMR (126 MHz, CDCl_3_):** δ = 162.7, 137.9, 137.5, 135.1, 134.9, 133.9, 129.1, 128.9, 128.7, 127.1, 126.9, 125.8, 121.3, 55.0, 38.9, 37.0, 29.5, 27.6 ppm; **FT-IR:** ν̃ = 3199, 2924, 2510, 1579, 1404, 1344, 1112, 998 cm^–1^; **HRMS**: calc. for [M + H]^+^ C_17_H_14_BrClNO: 361.9947 and 363.9927, found: 361.9947 and 363.9922; [α]_*D*_^*RT*^= −132.7 (CH_2_Cl_2_, *c* = 0.50); **HPLC conditions:** CHIRAPAK IC column, *iso*-propanol/*iso*-hexane = 30/70, flow rate = 0.5 mL min^–1^, major enantiomer: *t*_R_ = 36.4 min; minor enantiomer: *t*_R_ = 28.4 min.
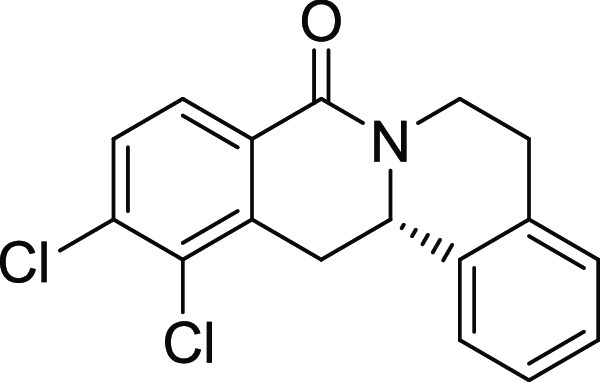


##### Major Isomer: (*S*)-11,12-Dichloro-5,6,13,13a-tetrahydro-8*H*-isoquinolino[3,2-*a*]isoquinolin-8-one (**3p**)

61% overall yield, (9.1:1 r.r.), 90% ee; **^1^H NMR (500 MHz, CDCl_3_):** δ = 7.97 (d, *J* = 8.4 Hz, 1H), 7.44 (dd, *J* = 8.4, 1.0 Hz, 1H), 7.30–7.23 (m, 2H), 7.26–7.17 (m, 1H), 7.20–7.14 (m, 1H), 4.92–4.80 (m, 2H), 3.63 (dd, *J* = 16.6, 3.9 Hz, 1H), 3.02–2.91 (m, 2H), 2.91–2.73 (m, 2H); **^13^C NMR (126 MHz, CDCl_3_):** δ = 163.20, 137.29, 136.72, 135.14, 134.91, 130.60, 129.14, 129.07, 129.02, 127.83, 127.21, 127.01, 126.06, 54.47, 38.86, 35.71, 29.50 ppm; **FT-IR:** ν̃ = 3181, 2926, 1703, 1404, 1311, 1188, 1021, 997 cm^–1^; **HRMS**: calc. for [M + H]^+^ C_17_H_14_Cl_2_NO: 318.0452, found: 318.0443; [α]_*D*_^*RT*^= −99.6 (CH_2_Cl_2_, *c* = 0.50); **HPLC conditions:** CHIRAPAK IC column, *iso*-propanol/*iso*-hexane = 30/70, flow rate = 0.5 mL min^–1^, major enantiomer: *t*_R_ = 36.8 min; minor enantiomer: *t*_R_ = 29.1 min.
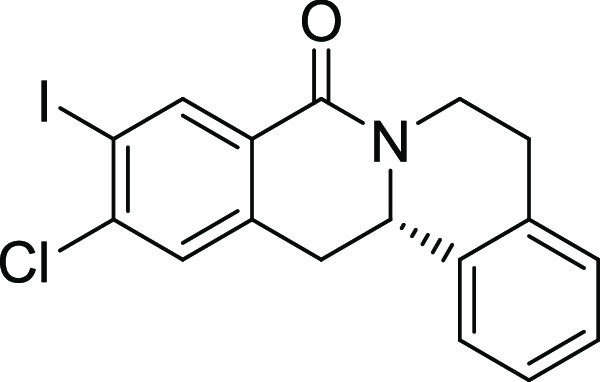


49% overall yield (3.7: 1 r.r.).

##### Minor Isomer: (*S*)-10-Iodo-11-chloro-5,6,13,13a-tetrahydro-8*H*-isoquinolino[3,2-*a*]isoquinolin-8-one (**3m**)

97% ee; **^1^H NMR (500 MHz, CDCl_3_):** δ = 8.51 (s, 1H), 7.30 (s, 1H), 7.22–7.15 (m, 5H), 4.90–4.84 (m, 2H), 3.11 (dd, *J* = 15.8, 3.7 Hz, 1H), 2.95–2.76 (m, 4H); **^13^C NMR (126 MHz, CDCl_3_):** δ = 162.55, 141.91, 140.42, 138.61, 135.16, 134.94, 129.14, 128.78, 127.65, 127.17, 126.94, 125.87, 96.17, 54.97, 38.92, 37.18, 29.55 ppm; **FT-IR:** ν̃ = 3191, 2856, 1599, 1434, 1242, 1116, 1081, 897 cm^–1^; **HRMS**: calc. for [M + H]^+^ C_17_H_14_IClNO: 409.9809, found: 409.9803; [α]_*D*_^*RT*^= −201.1 (CH_2_Cl_2_, *c* = 0.50); **HPLC conditions:** CHIRAPAK IC column, *iso*-propanol/*iso*-hexane = 30/70, flow rate = 0.5 mL min^–1^, major enantiomer: *t*_R_ = 33.7 min; minor enantiomer: *t*_R_ = 42.3 min.
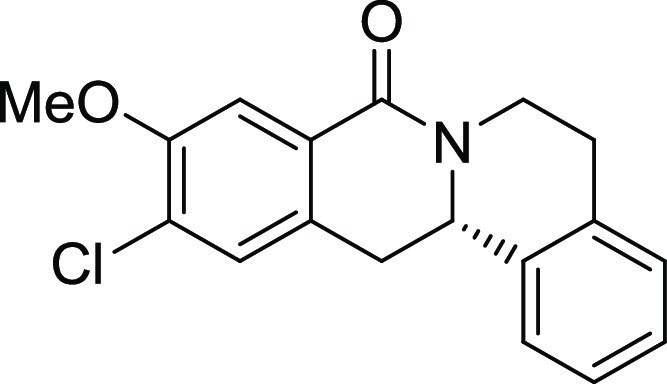


##### (*S*)-10-Methoxy-11-chloro-5,6,13,13a-tetrahydro-8*H*-isoquinolino[3,2-*a*]isoquinolin-8-one (**3n**)

69% yield (only one isomer), 96% ee; **^1^H NMR (500 MHz, CDCl_3_):** δ = 7.64 (s, 1H), 7.26–7.20 (m, 2H), 7.20–7.13 (m, 4H), 4.93–4.82 (m, 2H), 3.91 (s, 3H), 3.10 (dd, *J* = 15.6, 3.8 Hz, 1H), 3.00–2.90 (m, 2H), 2.89–2.77 (m, 2H); **^13^C NMR (126 MHz, CDCl_3_):** δ = 163.97, 154.39, 135.63, 134.99, 130.26, 129.05, 128.66, 128.55, 127.01, 126.86, 126.26, 125.92, 111.58, 56.50, 55.41, 38.90, 36.65, 29.66 ppm; **FT-IR:** ν̃ = 3201, 1622, 1451, 1355, 1306, 1224, 1118, 1021, 987 cm^–1^; **HRMS**: calc. for [M + H]^+^ C_18_H_17_ClNO_2_: 314.0948, found: 314.0944; [α]_*D*_^*RT*^= −206.1 (CH_2_Cl_2_, *c* = 0.50); **HPLC conditions:** CHIRAPAK IC column, *iso*-propanol/*iso*-hexane = 20/80, flow rate = 0.5 mL min^–1^, major enantiomer: *t*_R_ = 45.0 min; minor enantiomer: *t*_R_ = 40.6 min.
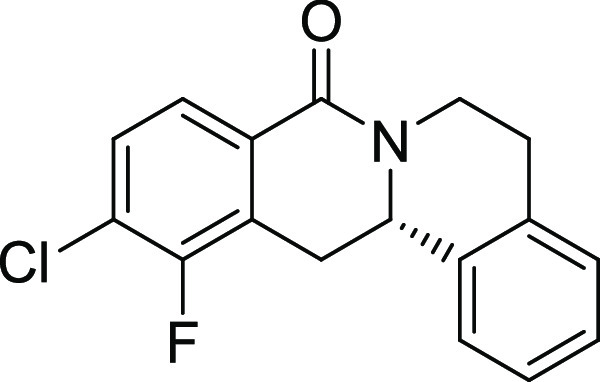


##### Major Isomer: (*S*)-12-Fluoro-11-chloro-5,6,13,13a-tetrahydro-8*H*-isoquinolino[3,2-*a*]isoquinolin-8-one (**3o**)

47% yield (8.1: 1 r.r.), 95% ee; **^1^H NMR (500 MHz, CDCl_3_):** δ = 7.84 (dd, *J* = 8.4, 1.2 Hz, 1H), 7.35 (ddd, *J* = 8.2, 7.0, 1.0 Hz, 1H), 7.29–7.14 (m, 5H), 4.93–4.82 (m, 2H), 3.50 (dd, *J* = 16.2, 3.8 Hz, 1H), 3.01–2.90 (m, 2H), 2.87–2.79 (m, 1H), 2.76–2.66 (m, 1H); **^13^C NMR (126 MHz, CDCl_3_):** δ = 163.11, 135.15, 134.93, 129.27, 129.11 (t, *J*CF = 2.4 Hz), 127.8, (d, *J*CF = 291.4 Hz), 127.22, 127.01, 126.01, 124.82, 124.79 (d, *J*CF = 21.3 Hz), 54.67, 38.84, 30.57, 29.53, 26.93 ppm; **^19^F NMR (470 MHz, CDCl_3_):** δ −121.41 (d, *J* = 7.1 Hz) ppm; **FT-IR:** ν̃ = 3207, 2926, 2516, 1629, 1363, 1226, 1081, 997, 873 cm^–1^; **HRMS**: calc. for [M + H]^+^ C_17_H_14_ClFNO: 302.0748, found: 302.0741; [α]_*D*_^*RT*^= −119.1 (CH_2_Cl_2_, *c* = 0.50); **HPLC conditions:** CHIRAPAK IC column, *iso*-propanol/*iso*-hexane 30/70, flow rate = 0.5 mL min^–1^, major enantiomer: *t*_R_ = 33.5 min; minor enantiomer: *t*_R_ = 27.2 min.
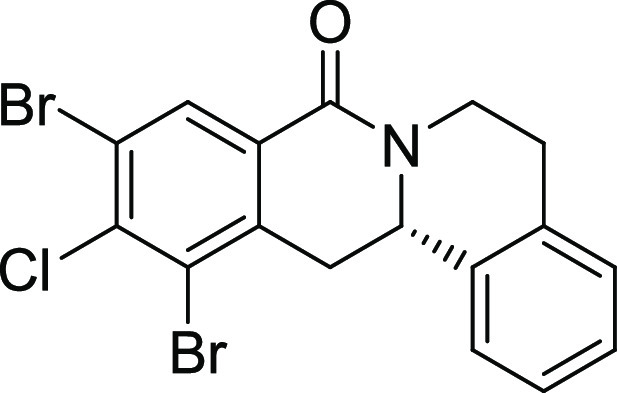


##### (*S*)-10,12-Dibromo-11-chloro-5,6,13,13a-tetrahydro-8*H*-isoquinolino[3,2-*a*]isoquinolin-8-one (**3q**)

41% yield, 95% ee; **^1^H NMR (500 MHz, CDCl_3_):** δ = 8.35 (s, 1H), 7.34–7.16 (m, 4H), 4.86–4.76 (m, 2H), 3.57 (dd, *J* = 15.8, 3.7 Hz, 1H), 2.98–2.94 (m, 2H), 2.96–2.86 (m, 2H), 2.75 (d, *J* = 3.3 Hz, 1H); **^13^C NMR (126 MHz, CDCl_3_):** δ = 162.11, 138.15, 134.83, 134.80, 132.63, 129.59, 129.18, 127.33, 127.08, 126.03, 124.00, 122.20, 54.46, 39.05, 38.92, 29.41 ppm; **FT-IR:** ν̃ = 3210, 2926, 1703, 1514, 1353, 1226, 1114, 973 cm^–1^; **HRMS**: calc. for [M + H]^+^ C_17_H_13_Br_2_ClNO: 439.9052 and 441.9032, found: 439.9054 and 441.9030; [α]_*D*_^*RT*^= −88.4 (CH_2_Cl_2_, *c* = 0.50); **HPLC conditions:** CHIRAPAK IC column, *iso*-propanol/*iso*-hexane = 20/80, flow rate = 0.5 mL min^–1^, major enantiomer: *t*_R_ = 40.5 min; minor enantiomer: *t*_R_ = 35.8 min.
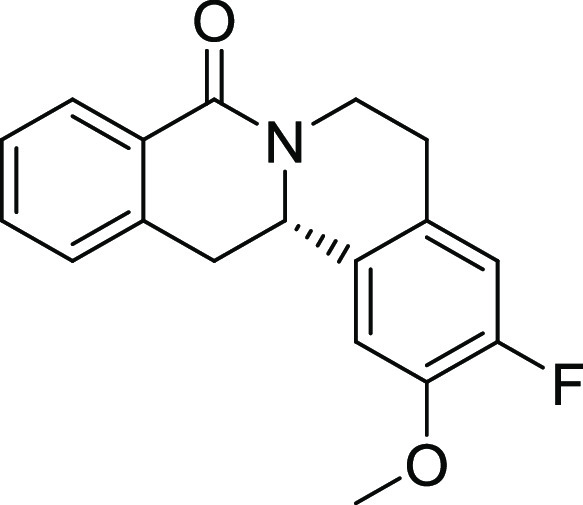


##### (*S*)-3-Fluoro-2-methoxy-5,6,13,13a-tetrahydro-8*H*-isoquinolino[3,2-*a*]isoquinolin-8-one (**3r**)

71% yield, 94% ee; **^1^H NMR (700 MHz, CDCl_3_):** δ = 8.14 (dd, *J* = 7.8, 1.4 Hz, 1H), 7.49–7.45 (m, 1H), 7.42–7.38 (m, 1H), 7.28–7.23 (m, 2H), 6.93 (d, *J* = 11.4 Hz, 1H), 6.82 (d, *J* = 8.1 Hz, 1H), 5.02–4.95 (m, 1H), 4.87 (dd, *J* = 13.6, 3.6 Hz, 1H), 3.92 (s, 3H), 3.21 (dd, *J* = 15.6, 3.7 Hz, 1H), 3.03–2.96 (m, 1H), 2.95–2.90 (m, 2H), 2.80–2.72 ppm (m, 1H); **^13^C NMR (176 MHz, CDCl_3_):** δ = 164.7, 151.3 (d, *J* = 247.0 Hz, 1C), 146.7 (d, *J* = 11.2 Hz, 1C), 137.1, 132.0, 131.7 (d, *J* = 3.6 Hz, 1C), 129.0, 128.7, 128.1 (d, *J* = 6.5 Hz, 1C), 127.5, 126.9, 116.3 (d, *J* = 18.0 Hz, 1C), 111.3 (d, *J* = 2.2 Hz, 1C), 56.7, 55.1, 38.7, 38.2, 29.0 ppm; **FT-IR:** ν̃ = 2928, 2853, 2515, 1605, 1462, 1401, 1314, 1103 cm^–1^; **HRMS:** calc. for [M + H]^+^ C_18_H_17_FNO_2_: 298.12378, found: 29812376; [α]_*D*_^*RT*^= −380.9 (CH_2_Cl_2_, *c* = 1.00); **HPLC conditions:** CHIRAPAK IC column, *iso*-propanol/*iso*-hexane = 50/50, flow rate = 0.5 mL min^–1^, major enantiomer: *t*_R_ = 37.3 min; minor enantiomer: *t*_R_ = 30.1 min.
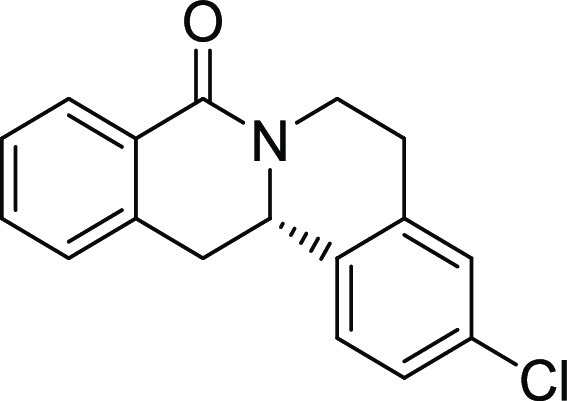


##### (*S*)-3-Chloro-5,6,13,13a-tetrahydro-8*H*-isoquinolino[3,2-*a*]isoquinolin-8-one (**3s**)

67% yield, 97% ee; **^1^H NMR (500 MHz, CDCl_3_):** δ = 8.14 (dd, *J* = 7.7, 1.1 Hz, 1H), 7.47 (td, *J* = 7.4, 1.4 Hz, 1H), 7.40 (t, *J* = 7.5 Hz, 1H), 7.28–7.24 (m, 2H), 7.24–7.19 (m, 2H), 5.03–4.94 (m, 1H), 4.91 (dd, *J* = 13.5, 3.7 Hz, 1H), 3.22 (dd, *J* = 15.7, 3.7 Hz, 1H), 3.04–2.92 (m, 3H), 2.89–2.79 ppm (m, 1H); **^13^C NMR (176 MHz, CDCl_3_):** δ = 164.6, 137.1, 137.1, 134.6, 132.7, 132.0, 129.0, 128.9, 128.7, 127.6, 127.5, 127.1, 127.0, 77.3, 77.1, 76.9, 55.0, 38.5, 37.8, 29.7 ppm; **FT-IR:** ν̃ = 3049, 2948, 2527, 1606, 1581, 1443, 1337, 1324, 1213, 1198 cm^–1^; **HRMS:** calc. for [M + H]^+^ C_17_H_15_ClNO: 284.08367, found: 284.08358; [α]_*D*_^*RT*^= −320.3 (CH_2_Cl_2_, *c* = 1.00); **HPLC conditions:** CHIRAPAK IC column, *iso*-propanol/*iso*-hexane = 50/50, flow rate = 0.5 mL min^–1^, major enantiomer: *t*_R_ = 31.3 min; minor enantiomer: *t*_R_ = 24.8 min.
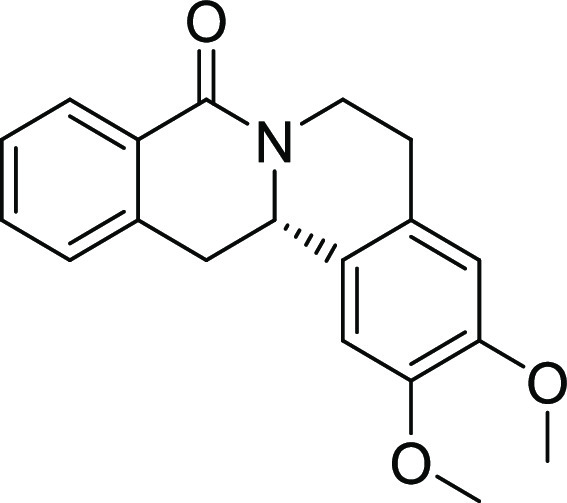


##### (*S*)-2,3-Dimethoxy-5,6,13,13a-tetrahydro-8*H*-isoquinolino[3,2-*a*]isoquinolin-8-one (**3t**)

71% yield, 95% ee; **^1^H NMR (400 MHz, CDCl_3_):** δ 8.14 (dd, *J* = 7.7, 1.4 Hz, 1H), 7.46 (td, *J* = 7.4, 1.4 Hz, 1H), 7.38 (t, *J* = 7.4 Hz, 1H), 7.29–7.22 (m, 1H), 6.72 (s, 1H), 6.69 (s, 1H), 5.03–4.95 (m, 1H), 4.86 (dd, *J* = 13.4, 3.7 Hz, 1H), 3.91 (s, 3H), 3.89 (s, 3H), 3.21 (dd, *J* = 15.7, 3.6 Hz, 1H), 3.02–2.90 (m, 3H), 2.81–2.72 ppm (m, 1H); **^13^C NMR (101 MHz, CDCl_3_):** δ 164.75, 148.20, 148.15, 137.42, 131.91, 129.24, 128.73, 127.80, 127.47, 127.46, 126.97, 111.66, 109.06, 56.33, 56.09, 55.14, 38.88, 38.27, 29.36 ppm; **FT-IR:** ν̃ = 2927, 2858, 1644, 1602, 1580, 1516, 1460, 1399, 1285, 1202, 1112, 1022 cm^–1^; **HRMS:** calc. for [M + H]^+^ C_19_H_20_NO_3_: 310.14377, found: 310.14441; [α]_*D*_^*RT*^= −353.6 (CH_2_Cl_2_, *c* = 1.00); **HPLC conditions:** CHIRAPAK IC column, *iso*-propanol/*iso*-hexane = 40/60, flow rate = 0.5 mL min^–1^, major enantiomer: *t*_R_ = 34.7 min; minor enantiomer: *t*_R_ = 30.8 min.
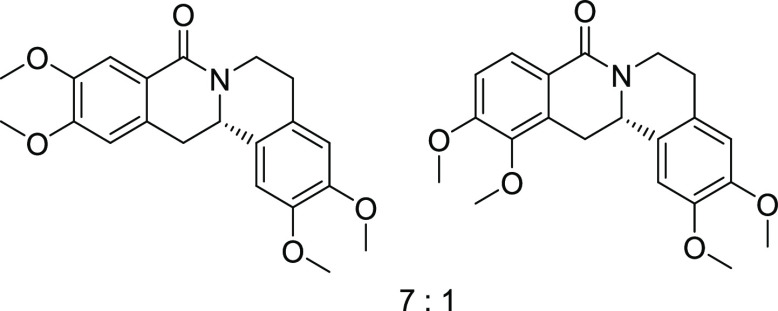


##### (*S*)-2,3,11,12-Tetramethoxy-5,6,13,13a-tetrahydro-8*H*-isoquinolino[3,2-*a*]isoquinolin-8-one (**3u**)

46% yield, 91% ee for the major; r.r. = 7:1, Major product, **^1^H NMR (400 MHz, CDCl_3_):** δ 7.65 (s, 1H), 6.72 (s, 1H), 6.71 (s, 1H), 6.69 (s, 1H), 4.98 (dd, *J* = 8.0, 2.6 Hz, 1H), 4.84 (dd, *J* = 13.7, 3.9 Hz, 1H), 3.95 (s, 3H), 3.94 (s, 3H), 3.91 (s, 3H), 3.90 (s, 3H), 3.14 (dd, *J* = 15.6, 3.9 Hz, 1H), 2.97–2.90 (m, 3H), 2.81–2.73 (m, 1H); **^13^C NMR (101 MHz, CDCl_3_):** δ 164.87, 152.09, 148.42, 148.18, 148.14, 131.12, 127.95, 127.55, 121.86, 111.67, 110.96, 109.36, 109.01, 56.31, 56.22, 56.10, 55.45, 38.88, 37.86, 29.43 ppm; **FT-IR:** ν̃ = 2932, 2837, 1644, 1602, 1514, 1455, 1429, 1256, 1221, 1098 cm^–1^; **HRMS:** calc. for [M + H]^+^ C_21_H_24_NO_5_: 370.16490, found: 370.16608; [α]_*D*_^*RT*^= −191.4 (CH_2_Cl_2_, *c* = 0.50); **HPLC conditions:** CHIRAPAK IC column, (CH_2_Cl_2_/EtOH = 100/2)/*iso*-hexane = 70/30, flow rate = 0.5 mL min^–1^, major enantiomer: *t*_R_ = 29.8 min; minor enantiomer: *t*_R_ = 41.4 min.
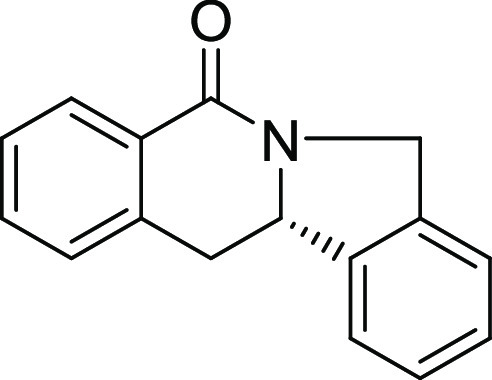


##### (*S*)-11b,12-Dihydroisoindolo[2,1-*b*]isoquinolin-5(7*H*)-one (**4**)

68% yield, 97% ee; **^1^H NMR (700 MHz, CDCl_3_)** δ 8.14–8.09 (m, 1H), 7.47 (td, *J* = 7.4, 1.2 Hz, 1H), 7.42–7.38 (m, 2H), 7.38–7.34 (m, 2H), 7.34–7.31 (m, 1H), 7.30 (d, *J* = 7.5 Hz, 1H), 5.24 (dd, *J* = 13.5, 3.5 Hz, 1H), 5.15 (d, *J* = 15.7 Hz, 1H), 4.82 (d, *J* = 15.6 Hz, 1H), 3.35 (dd, *J* = 15.0, 4.0 Hz, 1H), 3.08 ppm (t, *J* = 14.3 Hz, 1H); **^13^C NMR (176 MHz, CDCl_3_)** δ 163.16, 140.11, 137.19, 137.09, 132.00, 130.48, 128.44, 128.07, 127.92, 127.59, 127.55, 123.50, 122.05, 61.57, 50.84, 34.72 ppm; **FT-IR:** ν̃ = 3049, 2926, 2861, 1604, 1588, 1573, 1462, 1365, 1341 cm^–1^; **HRMS:** calc. for [M + H]^+^ C_16_H_14_NO: 236.10699, found: 236.10698; [α]_*D*_^*RT*^= −294.0 (CH_2_Cl_2_, *c* = 1.00); **HPLC conditions:** CHIRAPAK IC column, *iso*-propanol/*iso*-hexane = 50/50, flow rate = 0.5 mL min^–1^, major enantiomer: *t*_R_ = 34.1 min; minor enantiomer: *t*_R_ = 27.7 min.
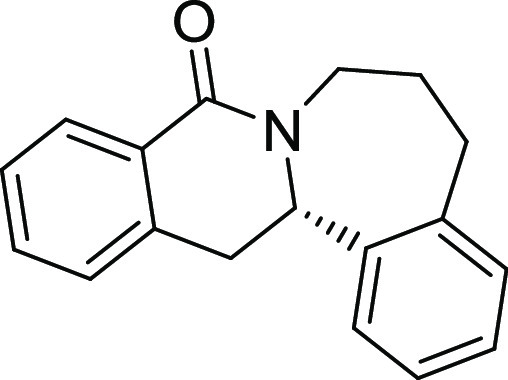


##### (*S*)-6,7,14,14a-Tetrahydrobenzo[3,4]azepino[1,2-*b*]isoquinolin-9(5*H*)-one (**5**)

42% yield, 96% ee; **^1^H NMR (700 MHz, CDCl_3_):** δ = 8.04 (d, *J* = 7.8 Hz, 1H), 7.46 (td, *J* = 7.5, 1.4 Hz, 1H), 7.34 (td, *J* = 7.6, 1.1 Hz, 1H), 7.28–7.24 (m, 1H), 7.21–7.13 (m, 2H), 7.12–7.04 (m, 2H), 5.00 (dd, *J* = 8.2, 5.1 Hz, 1H), 4.77–4.71 (m, 1H), 3.42 (dd, *J* = 16.1, 8.2 Hz, 1H), 3.30–3.24 (m, 1H), 3.13–3.05 (m, 2H), 2.89 (ddd, *J* = 14.3, 6.9, 4.2 Hz, 1H), 2.02–1.97 (m, 1H), 1.85–1.78 ppm (m, 1H); **^13^C NMR (176 MHz, CDCl_3_):** δ = 163.55, 139.92, 138.95, 137.26, 132.00, 130.42, 129.55, 128.58, 128.12, 127.27, 126.96, 126.88, 126.66, 60.47, 45.08, 33.78, 32.91, 26.28 ppm; **FT-IR:** ν̃ = 3271, 3058, 2890, 1603, 1576, 1450, 1422, 1290 cm^–1^; **HRMS:** calc. for [M + H]^+^ C_18_H_18_NO: 264.13829, found: 264.13832; [α]_*D*_^*RT*^= 52.2 (CH_2_Cl_2_, *c* = 0.50); **HPLC conditions:** CHIRAPAK IC column, *iso*-propanol/*iso*-hexane = 50/50, flow rate = 0.5 mL min^–1^, major enantiomer: *t*_R_ = 27.8 min; minor enantiomer: *t*_R_ = 22.8 min.

## Supporting Information Available

Structure of compound **3l** (Figure S1); dose–response curves in the osteoblast differentiation assay for 8-oxotetrahydroprotoberberine (Figure S2); influence of Picoberin on Hh, Wnt, TGFβ, and BMP4 signaling (Figure S3); analysis of gene expression upon treatment with Picoberin (Figures S4 and S5); GPCR panel for Picoberin (Table S1; nuclear receptor panel (Table S2); proteome profiling for upon treatment with Picoberin (Figure S6); influence of Picoberin on XRE reporter activity in different cell lines (Figure S7); docking of Picoberin (Figures S8–S12, Tables S3–S4) influence of known AhR agonists on Hh-dependent osteogenesis (Figure S13); and NMR and HPLC spectra (PDF)
Molecular formula strings (CSV)
Homology models (ZIP)

## Abbreviations Used

ADH7: alcohol dehydrogenase 7
AhR: aryl hydrocarbon receptor
AIP: AhR interacting protein
ALDH: aldehyde dehydrogenase
ALDH3A1: aldehyde dehydrogenase 3 family member A1
Alpl: alkaline phosphatase gene
BGP: β-glycerophosphate
BMP: bone morphogenetic factor
CLOCK: circadian locomotor output cycles kaput
Cp^x^: chiral cyclopentadienyl ligand
CYP1B1: cytochrome 1B1
FICZ: 6-formylindolo[3,2-*b*]carbazole
GLI: glioma-associated oncogene
GPCR: G-protein coupled receptor
Hh: Hedgehog
HSP90: heat-shock protein 90
IFD: induced-fit docking
NQO1: NAD(*P*)H quinone dehydrogenase 1
P23: prostaglandin E synthase 3
PTC1: Patched 1
Shh: Sonic hedgehog
SUFU: suppressor of fused
TCDD: 2,3,7,8-tetrachlorodibenzodioxin
TGFβ: transforming growth factor-beta
XRE: xenobiotic response element
