# Supplementary material for: The Highly Potent AhR Agonist Picoberin Modulates Hh-Dependent Osteoblast Differentiation
Source: J Med Chem. 2022 Dec 2;65(24):16268–89. doi: 10.1021/acs.jmedchem.2c00956 (PMC9791665; doi:10.1021/acs.jmedchem.2c00956)
Supplement: Supplementary file 1 — jm2c00956_si_001.pdf [file jm2c00956_si_001.pdf]

# Supporting Information

## The highly potent AhR agonist Picoberin modulates Hh-dependent osteoblast differentiation

Jana Flegel[1,2], Saad Shaaban[1, 11], Zhi Jun Jia[1,12], Britta Schulte[1,2], Yilong Lian[4], Adrian Krzyzanowski[1,2], Malte Metz[1], Tabea Schneidewind[1,2], Fabian Wesseler[1,2], Anke Flegel[1], Alisa Reich[1], Alexandra Brause[1], Gang Xue[1], Minghao Zhang[8], Lara Dötsch[1,2], Isabelle D. Stender[9], Jan-Erik Hoffmann[9], Rebecca Scheel[3], Petra Janning[1], Fraydoon Rastinejad[8], Dennis Schade[10], Carsten Strohmann[3], Andrey P. Antonchick[1,2,13], Sonja Sievers[1,7], Pedro Moura-Alves[4,5,6], Slava Ziegler[1], Herbert Waldmann[1,2]*

[1] Max Planck Institute of Molecular Physiology, Department of Chemical Biology, Dortmund 44227, Germany

[2] Technical University Dortmund, Faculty of Chemistry, Chemical Biology, Dortmund 44227, Germany

[3] Technical University Dortmund, Faculty of Chemistry, Inorganic Chemistry, Dortmund 44227, Germany

[4] Ludwig Institute for Cancer Research, Nuffield Department of Clinical Medicine, University of Oxford, Oxford OX3 7DQ, United Kingdom

[5] i3S-Instituto de Investigação e Inovação em Saúde, Universidade do Porto, 4200-135 Porto, Portugal

[6] IBMC-Instituto de Biologia Molecular e Celular, Universidade do Porto, 4200-135 Porto, Portugal

[7] Compound Management and Screening Center, Dortmund 44227, Germany

[8] Nuffield Department of Medicine, Target Discovery Institute, University of Oxford, Oxford, OX3 7FZ, UK

[9] Protein Chemistry Facility, Max Planck Institute of Molecular Physiology, Dortmund 44227, Germany

[10] Dept. of Pharmaceutical & Medicinal Chemistry, Institute of Pharmacy, Christian-Albrechts-University of Kiel, Kiel 24118, Germany

[11] Faculty of Chemistry, Institute of Organic Chemistry, University of Vienna Währinger Str. 38, Vienna 1090, Austria

[12] Key Laboratory of Birth Defects and Related Diseases of Women and Children, Evidence-Based Pharmacy Center, West China Second University Hospital, Sichuan University Chengdu 610041, China

[13] Department of Chemistry and Forensics, School of Science and Technology, Nottingham Trent University, Clifton Lane, Nottingham, NG11 8NS, United Kingdom

[*] corresponding author

# Contents

Supporting Figures ..... S3

Supporting Tables ..... S18

NMR and HPLC spectra ..... S24

## Supporting Figures

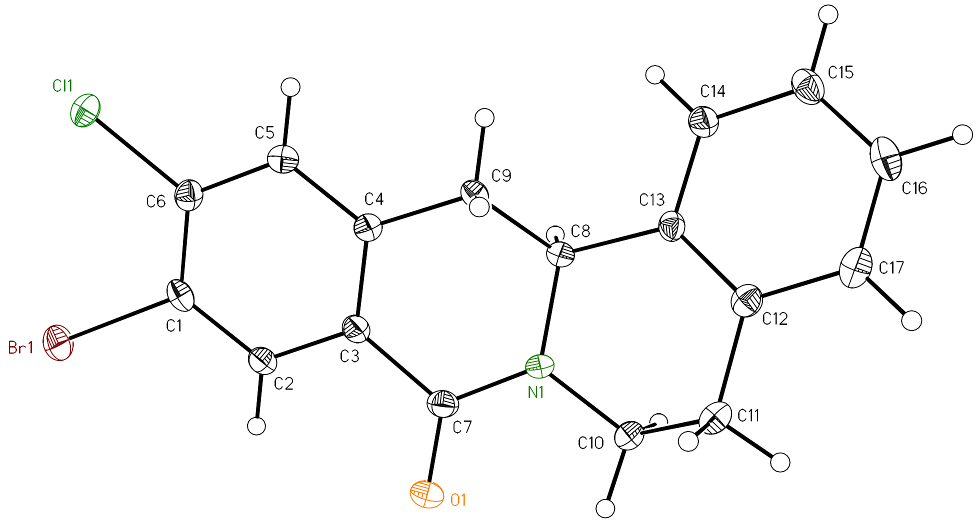


$C_{17}H_{13}BrClNO$

**Figure S1 (related to Figure 1):** Ortep plot of the molecular structure in the crystal of compound **3l**.[4] The displacement ellipsoids are drawn at 50% probability level. Numbering scheme of hydrogen atoms is omitted for clarity.

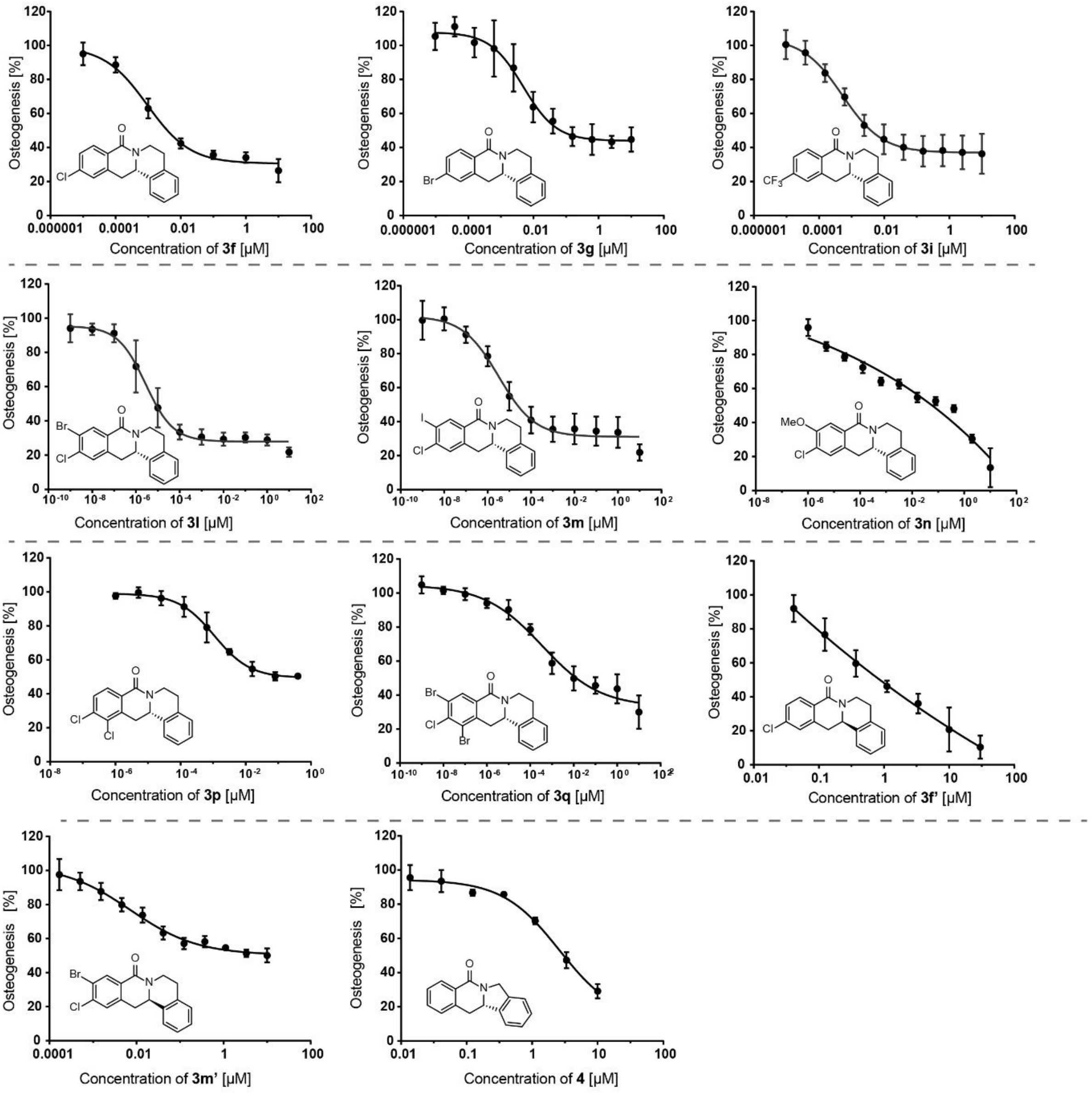


**Supporting Figure S2 (related to Table 1): Structure-activity relationship of 8-oxotetrahydroprotoberberines in Hh induced osteoblast differentiation.** C3H10T1/2 cells were treated with 1.5 µM purmorphamine and different concentrations of the compounds or DMSO as a control. Alkaline phosphatase activity was determined after 96h. The activity of alkaline phosphatase in samples that were treated with purmorphamine was set to 100 %. Data are mean values of at least three biological replicates and three technical replicates per biological replicate (N = 3, n ≥ 3).

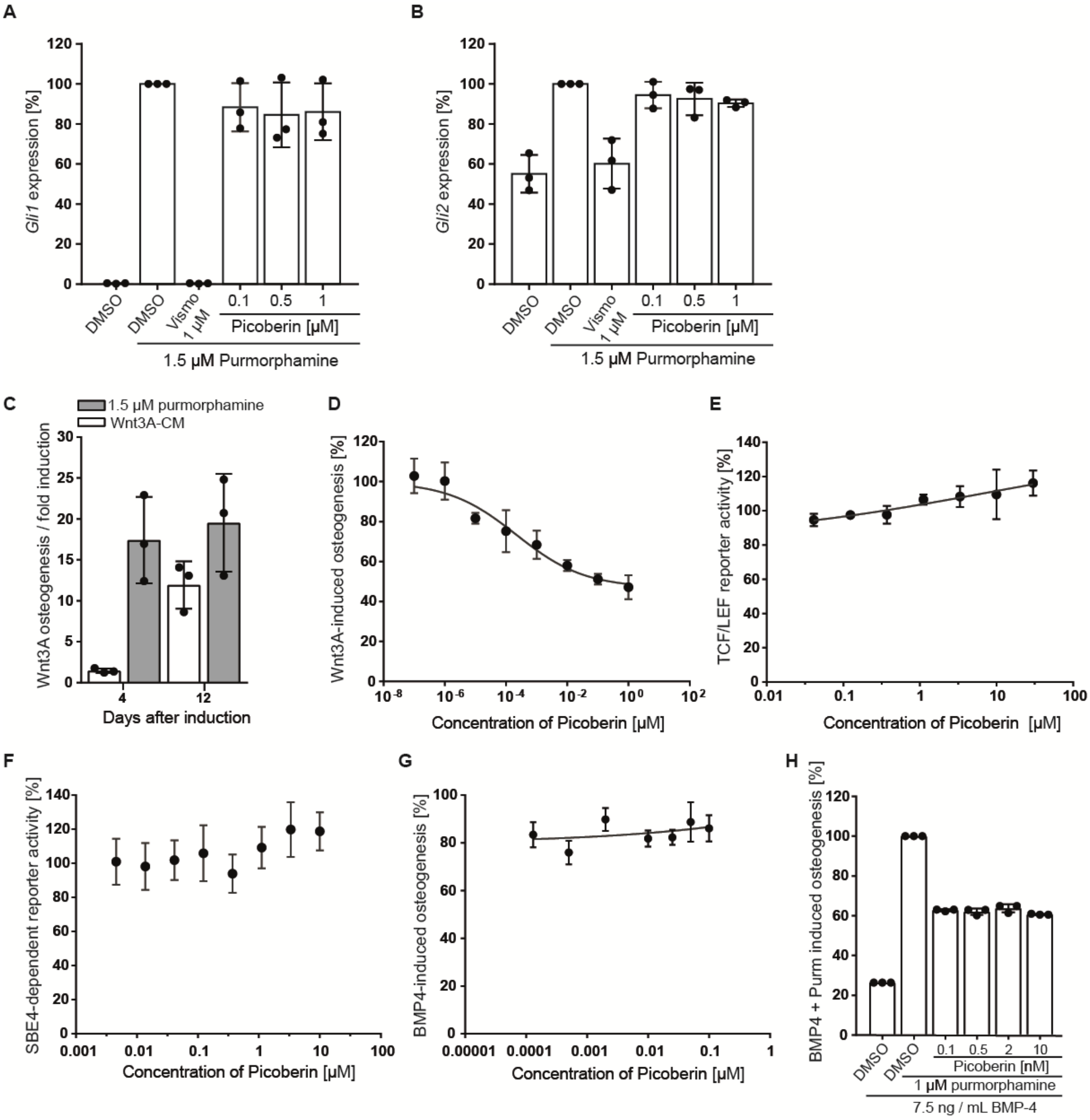


**Supporting Figure S3 (related to Figure 2): Influence of Picoberin on pathways involved in osteogenesis. A** and **B:** C3H10T1/2 cells were treated with 1.5 µM purmorphamine and different concentrations of Picoberin or DMSO as a control. After 96 h, *Gli1 and Gli2* gene expression levels were quantified by means of RT-qPCR. **C-D**: C3H10T1/2 cells were treated with Wnt3A-conditioned medium (Wnt3A-CM) or control medium, or with 1.5 µM purmorphamine or DMSO. Alkaline phosphatase levels were determined after 4 and 12 days and values of all samples were related to the values of samples that were treated with control medium (which were set to 1). **D:** C3H10T1/2 cells were treated with WCM and different concentrations of Picoberin or DMSO for 12 days. The activity of alkaline phosphatase was determined and values of WCM / DMSO-treated samples were set to 100 %. **E:** TCF/LEF-dependent reporter gene assay. HEK293T cells were transiently transfected with plasmids encoding for a TCF/LEF-dependent firefly luciferase (SuperTOPflash), pcDNA-Wnt3A and pRL-TK-*Renilla* luciferase for 6 h. Cells were then treated with different concentrations of Picoberin or DMSO for 24 h. Firefly luciferase activities were normalized to *Renilla* luciferase activities and all values were related to values obtained for DMSO (100 %). **F:** TGFβ-dependent reporter gene assay. HEK293T cells transiently transfected with plasmids encoding for a TGFβ-dependent firefly luciferase and *Renilla* luciferase control vector were treated with

20 ng/mL TGFβ-I and different concentrations of Picoberin or DMSO for 24 h. Firefly luciferase activities were normalized to *Renilla* luciferase activities and all values were related to values obtained for DMSO + TGFβ-I (100 %). **G:** Influence of Picoberin on 7.5 ng/mL BMP4-induced osteoblast differentiation of C2C12 cells. **H:** Influence of Picoberin on 7.5 ng/mL BMP4 and 1 µM purmorphamine induced osteoblast differentiation of C2C12 cells. Values were related to values obtained for cells treated with 7.5 ng/mL BMP4, 1 µM purmorphamine and DMSO were set to 100 %. Data are mean values of three biological replicates and three technical replicates per biological replicate (N = 3, n = 3).

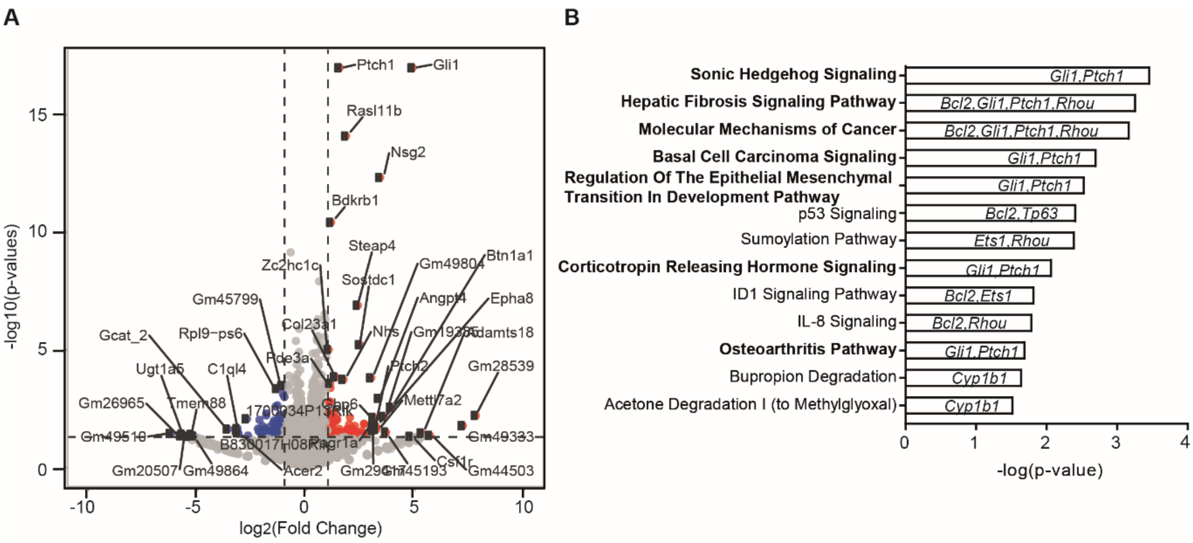


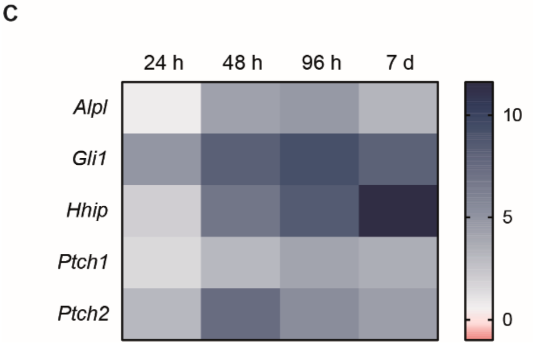


D

| Gene | Timepoint 24 h | 48 h | 96 h | 7d |
|---|---|---|---|---|
| *Alpl* | 0.454 | 0 | 0 | 0 |
| *Gli1* | 0 | 0 | 0 | 0 |
| *Hhip* | 0.066 | 1.42E-12 | 0 | 6.93E-07 |
| *Ptch1* | 0 | 0 | 0 | 0 |
| *Ptch2* | 0.022 | 0.002 | 1.76E-12 | 9.66E-09 |

**Supporting Figure S4 (related to Figure 3): Activation of Hh signaling in C3H10T1/2 cells upon treatment with purmorphamine.** C3H10T1/2 cells were treated with 1.5 µM purmorphamine or DMSO as a control. Total RNA was isolated after 24 h, 48 h, 96 h or 7 days and samples were subjected to Illumina sequencing to quantify global gene expression levels. **A:** Volcano plot of samples treated with 1.5 µM purmorphamine and DMSO compared to samples treated with DMSO for 24 h. Blue dots: significantly upregulated genes. Red dots: significantly down-regulated genes. **B:** Pathway over-representation analysis using the IPA software for samples that were treated with 1.5 µM purmorphamine compared to samples treated with DMSO for 24 h. Pathways regulated by the Hh target genes *Gli1* and *Ptch1* are marked in bold. Regulated genes detected in the transcriptome sequencing and assigned to the pathways are displayed in the respective bars. **C/D:** Heatmap of fold changes (**C**) and p-values (**D**) of Hh target genes and *Alpl* in cells that were treated for the indicated time with 1.5 µM purmorphamine compared to DMSO.

A

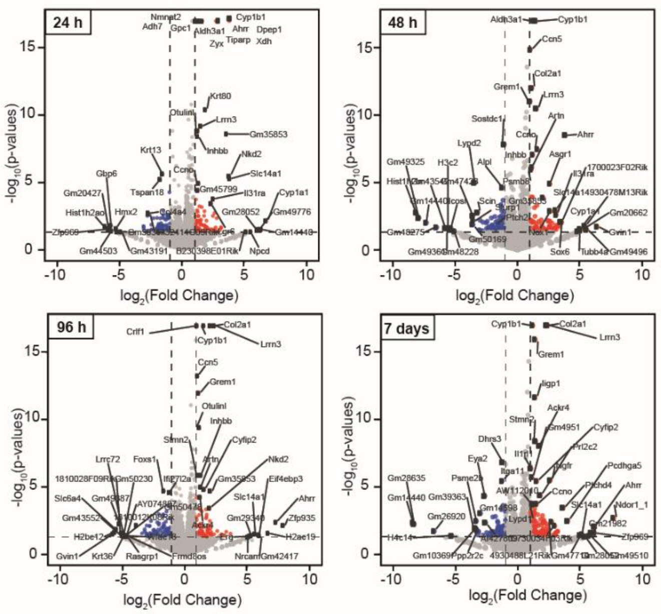


B

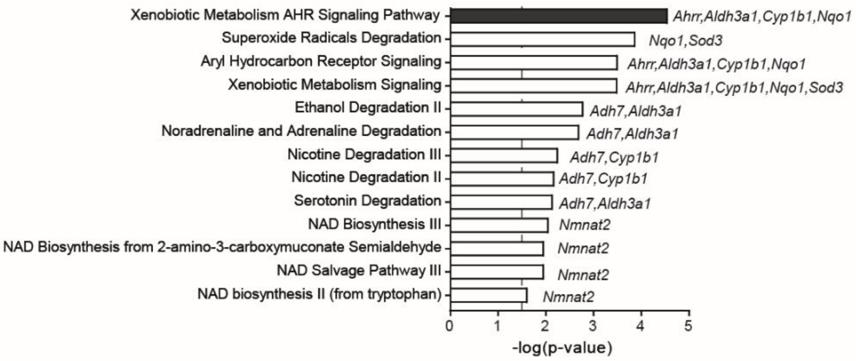


C

| Gene | Timepoint 24 h | | 48 h | | 96 h | | 7d | |
|---|---|---|---|---|---|---|---|---|
| | $log_2$(FC) | p-value | $log_2$(FC) | p-value | $log_2$(FC) | p-value | $log_2$(FC) | p-value |
| *Aldh3a1* | 6.68 | 0 | 1.34 | 0 | 0.86 | 1.1E-16 | 0.74 | 9.1E-09 |
| *Adh7* | 1.00 | 0 | 1.72 | 3.0E-14 | 0.58 | 1.4E-08 | 0.56 | 2.6E-08 |
| *Ahr* | -0.43 | 0.012 | -0.65 | 2.0E-03 | -0.75 | 8.8E-06 | -0.49 | 6.5E-03 |
| *Ahrr* | 3.75 | 6.0E-13 | 3.86 | 3.0E-09 | 7.28 | 3.9E-03 | 6.12 | 9.3E-03 |
| *Cyp1b1* | 1.59 | 0 | 1.47 | 0 | 1.50 | 0 | 1.19 | 0 |
| *Nqo1* | 0.87 | 2.0E-08 | 0.42 | 0.057 | 0.59 | 1.3E-04 | 0.36 | 0.02 |
| *Sod3* | 0.67 | 8.0E-11 | 0.47 | 4.0E-06 | 0.55 | 1.3E-07 | 0.48 | 2.0E-06 |
| *Tiparp* | 1.14 | 0 | 0.66 | 9.0E-10 | 0.52 | 1.2E-06 | 0.40 | 1.7E-03 |
| *Xdh* | 1.40 | 0 | 0.64 | 6.0E-07 | 0.42 | 4.3E-04 | 0.21 | 0.07 |

**Supporting Figure S5 (related to Figure 3): Influence of Picoberin on gene expression. NGS data.** C3H10T1/2 cells were treated with 1.5 µM purmorphamine and 1 nM Picoberin or DMSO as a control. Total mRNA was isolated after 24 h, 48 h, 96 h or 7 days and samples were subjected to Illumina sequencing to quantify global gene expression levels. Values of samples treated with 1.5 µM purmorphamine and 1 nM Picoberin are compared to values of samples treated with 1.5 µM purmorphamine and DMSO. **A:** Volcano plots for samples treated with 1.5 µM purmorphamine and 1 nM Picoberin compared to samples treated with 1.5 µM purmorphamine and DMSO. Red dots: significantly upregulated genes. Blue dots: significantly down-regulated genes. **B:** Pathway over-representation analysis using the Ingenuity Pathway Analysis (IPA) software for samples that were treated with 1.5 µM purmorphamine and 1 nM Picoberin compared to samples treated with 1.5 µM purmorphamine and DMSO for 24 h. **C:** Fold changes and p-values of AhR signaling-related gene levels in cells that were treated for the indicated time with 1.5 µM purmorphamine and 1 nM Picoberin compared to samples treated with 1.5 µM purmorphamine and DMSO.

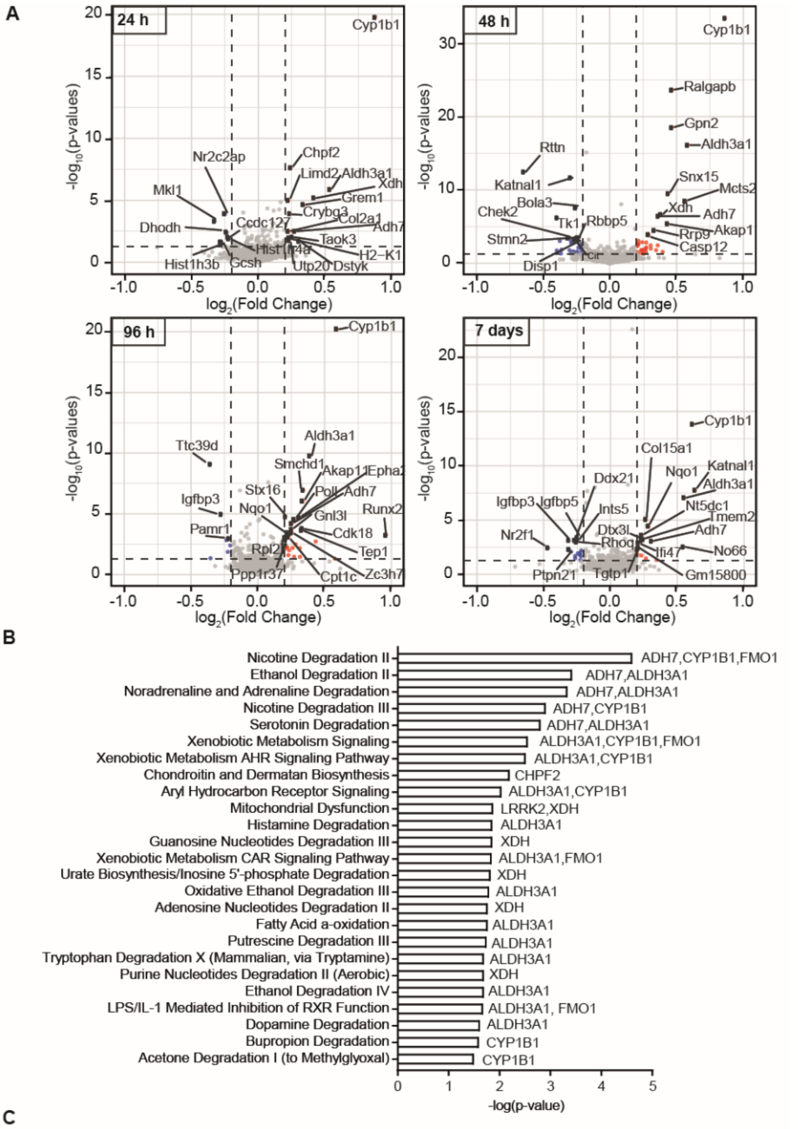


C

| Gene | 24 h | | 48 h | | 96 h | | 7d | |
|---|---|---|---|---|---|---|---|---|
| | $\log_2$(FC) | p-value | $\log_2$(FC) | p-value | $\log_2$(FC) | p-value | $\log_2$(FC) | p-value |
| *Aldh3a1* | 0.54 | 1.4E-06 | 0.58 | 7.4E-17 | 0.39 | 1.6E-10 | 0.55 | 9.3E-08 |
| *Adh7* | 0.27 | 3.6E-03 | 0.36 | 4.1E-07 | 0.25 | 6.4E-05 | 0.31 | 8.7E-04 |
| *Cyp1b1* | 0.88 | 1.6E-20 | 0.86 | 3.4E-34 | 0.59 | 6.2E-21 | 0.61 | 1.5E-14 |
| *Nqo1* | 0.17 | 5.0E-02 | 0.22 | 8.7E-04 | 0.22 | 3.9E-04 | 0.28 | 3.9E-05 |
| *Xdh* | 0.42 | 6.7E-06 | 0.38 | 2.4E-07 | 0.14 | 1.5E-02 | 0.31 | 8.7E-04 |

(Timepoint spans the 24 h, 48 h, 96 h and 7d columns.)

**Supporting Figure S6 (related to Figure 3): Influence of Picoberin on protein levels. Proteome Profiling data.** C3H10T1/2 cells were treated with 1.5 µM purmorphamine and 1 nM Picoberin or 1.5 µM purmorphamine and DMSO as a control. Cell lysates were collected after 24 h, 48 h, 96 h or 7 days and MS/MS analysis to quantify protein abundances. Values of samples treated with 1.5 µM purmorphamine and 1 nM Picoberin are compared to values of samples treated with 1.5 µM purmorphamine and DMSO. **A:** Volcano plots. Red dots: significantly upregulated proteins. Blue dots: significantly down-regulated proteins. **B:** Pathway overrepresentation analysis of samples treated with 1.5 µM purmorphamine and 1 nM Picoberin for 24 h compared to samples treated with 1.5 µM purmorphamine and DMSO for 24 h. **C:** Differential protein expression of AhR target gene-encoded proteins identified in the data set after comparison of samples treated with 1.5 µM purmorphamine and 1 nM Picoberin compared to samples treated with 1.5 µM purmorphamine and DMSO for the indicated times. Data are mean values of three biological replicates (n = 3).

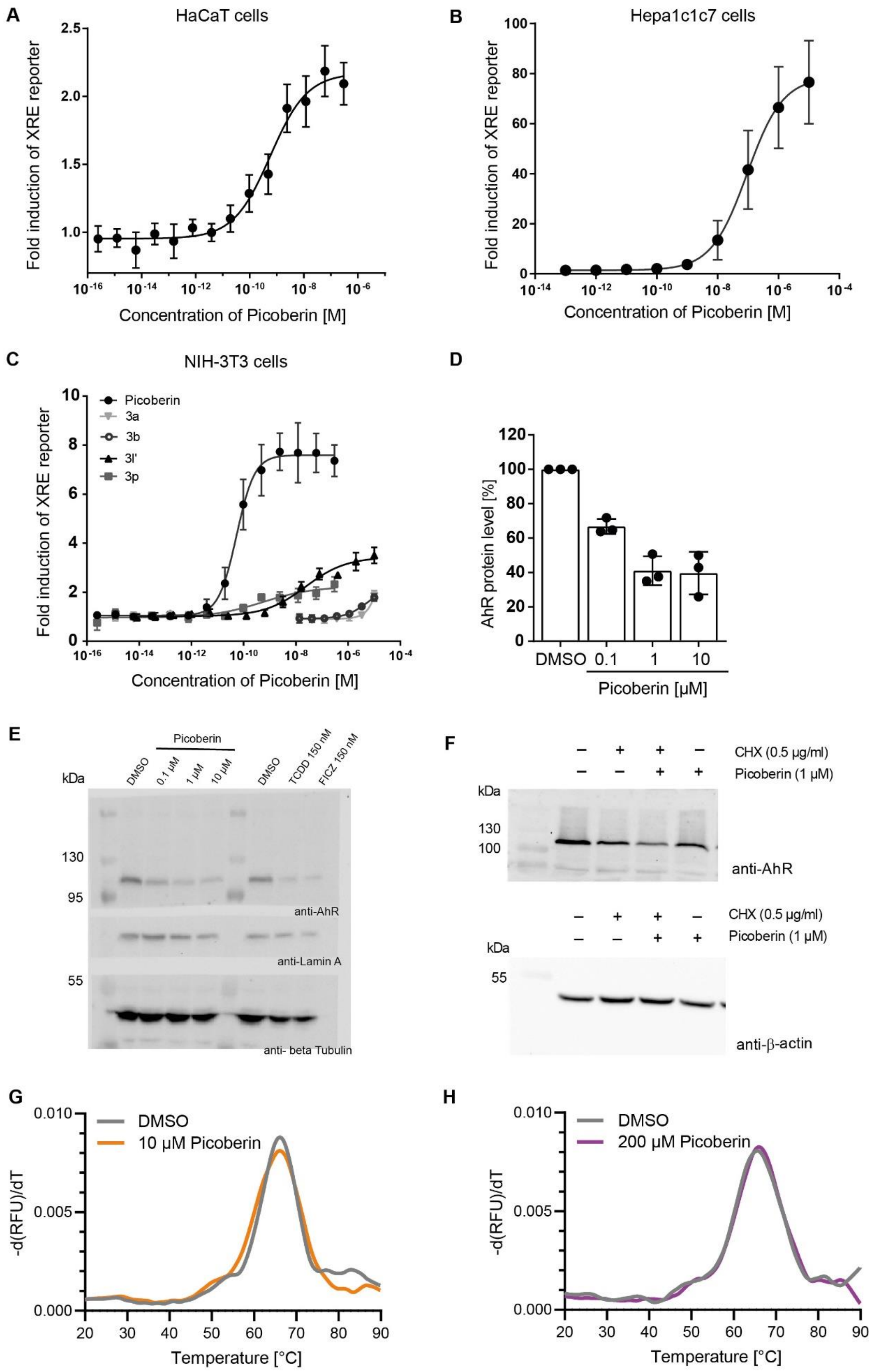
A
HaCaT cells
Fold induction of XRE reporter
Concentration of Picoberin [M]
B
Hepa1c1c7 cells
Fold induction of XRE reporter
Concentration of Picoberin [M]
C
NIH-3T3 cells
Picoberin
3a
3b
3l'
3p
Fold induction of XRE reporter
Concentration of Picoberin [M]
D
AhR protein level [%]
DMSO
0.1
1
10
Picoberin [µM]
E
Picoberin
kDa
DMSO
0.1 µM
1 µM
10 µM
DMSO
TCDD 150 nM
FICZ 150 nM
130
95
anti-AhR
anti-Lamin A
55
anti- beta Tubulin
F
CHX (0.5 µg/ml)
Picoberin (1 µM)
kDa
130
100
anti-AhR
CHX (0.5 µg/ml)
Picoberin (1 µM)
kDa
55
anti-β-actin
G
DMSO
10 µM Picoberin
-d(RFU)/dT
Temperature [°C]
H
DMSO
200 µM Picoberin
-d(RFU)/dT
Temperature [°C]

**Supporting Figure S7 (related to Figure 4): Influence of Picoberin on XRE-dependent reporter activity, AhR levels and on the melting of ARNT.** XRE-dependent reporter gene assay. HaCaT reporter cells (**A**), Hepa1c1c7 reporter cells (**B**) or NIH-3T3 reporter cells (**C**) were treated with different concentrations of Picoberin, its analogues or DMSO. Reporter gene activity was detected after 4 h. Data are mean values of three biological replicates (n = 3). **D**: Quantification of band intensities from 4F (n = 3). **E**: Uncropped immunoblot shown in Figure 4F. **F**: Uncropped immunoblots shown in Figure 4G. **G** and **H**: nanoDSF experiment using His-ARNT and 10 µM (G) or 200 µM (H) Picoberin. Data are representative of three biological replicates.

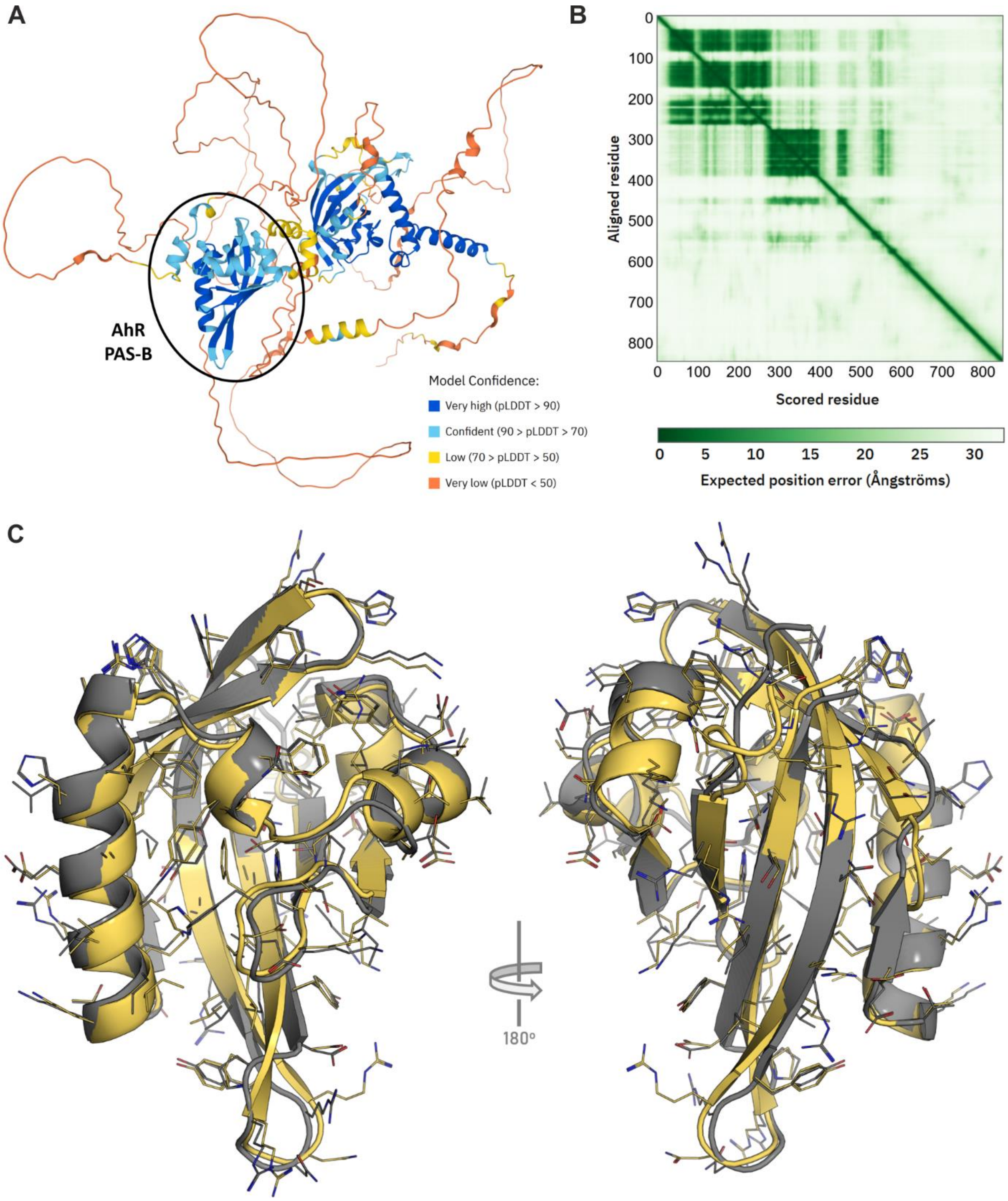


**Supporting Figure S8 (related to Figure 4): Predicted structure of murine AhR obtained from the AlphaFold Protein Structure Database. A:** 3D structure of AhR, with the protein sections labelled by the model confidence classification. **B:** Visualisation of the expected position error within the predicted model. The prediction of the PAS-B ligand binding domain structure (residue numbers: 276-380) is characterised by good model confidence and low expected position error. **C:** Superposition of the AlphaFold (yellow) and SWISS-MODEL (grey) murine AhR PAS-B models. RMSD between the structures: 0.842 Å.

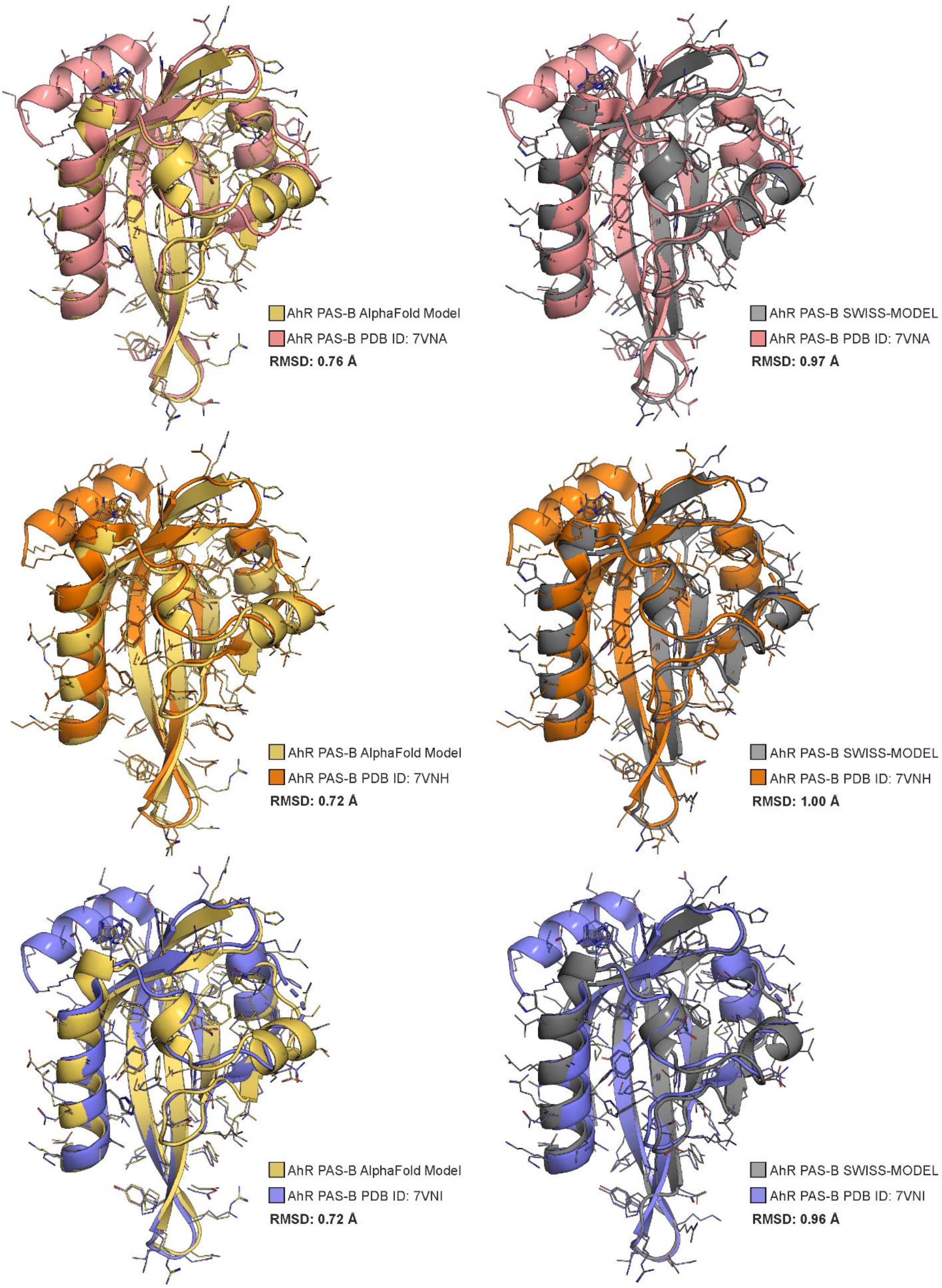


**Supporting Figure S9 (related to Figure 4): Comparison of the AhR PAS-B AlphaFold and SWISS-MODEL generated models for *Mus musculus* with the experimentally obtained crystal structures of PAS-B for *Drosophila melanogaster* available from the Protein Data Bank (PDB IDs: 7VNA, 7VNH and 7VNI).**

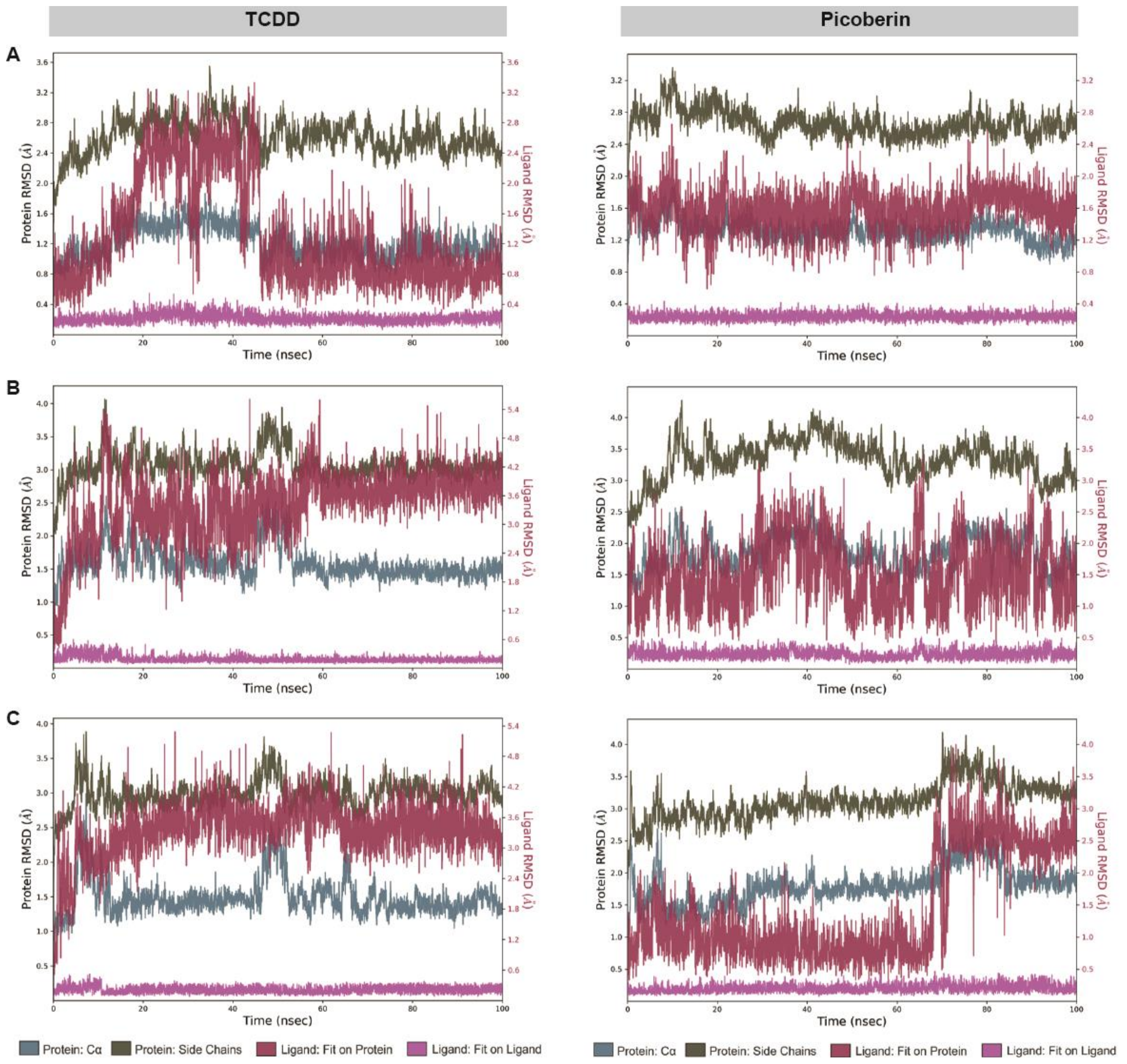


**Supporting Figure S10 (related to Figure 4): Protein and ligand root mean square deviation (RMSD) values during MD production runs with TCDD and Picoberin. A:** Production runs with the AlphaFold AhR PAS-B. **B:** Production runs with the homology model of PAS-B (SWISS-MODEL). **C:** Repeated production runs with the homology model of PAS-B (SWISS-MODEL).

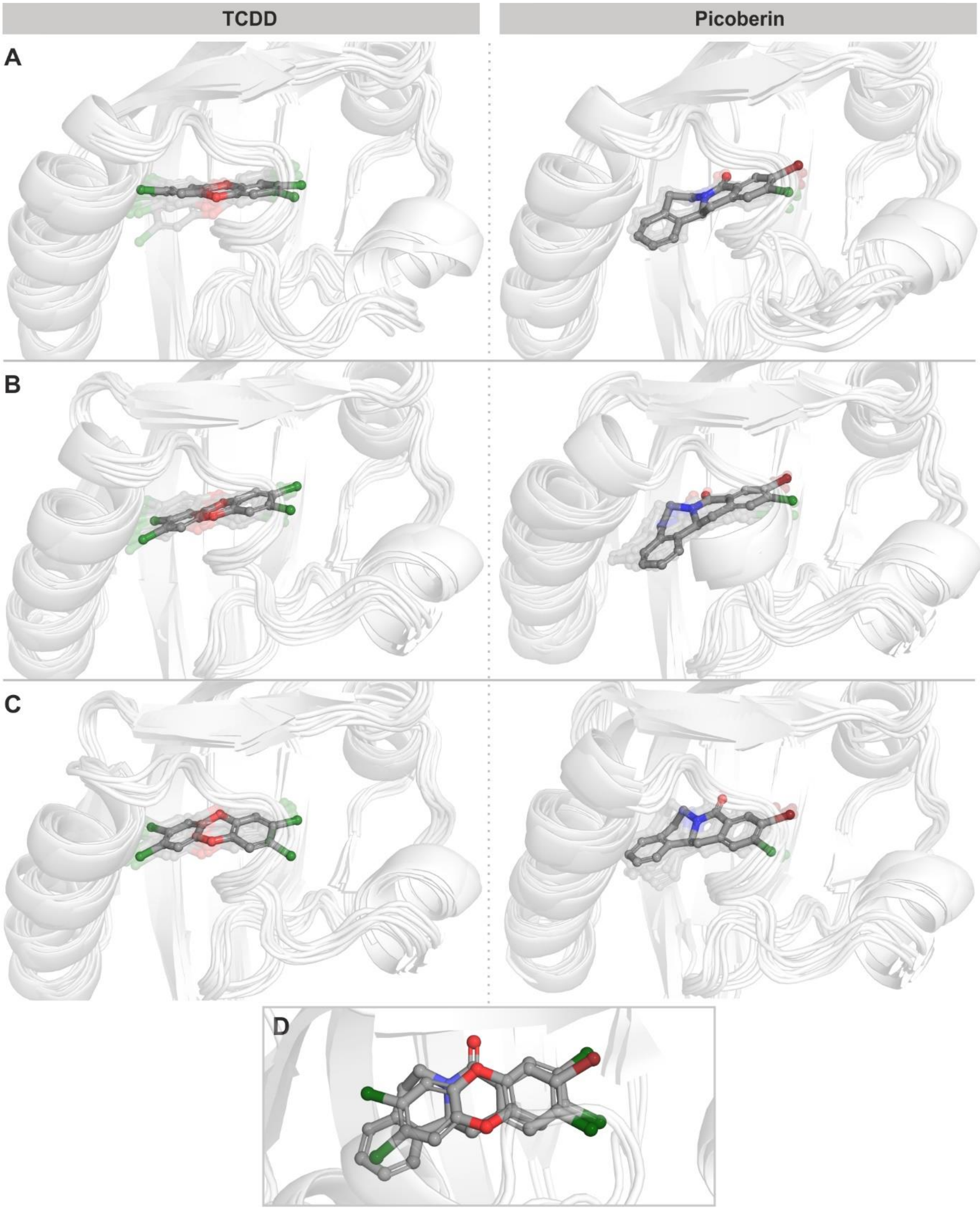


**Supporting Figure S11 (related to Figure 4): Dynamic view of the protein-ligand complexes during MD simulations.** Superpositions show 9 snapshots taken at even intervals from the final 20 ns of the MD simulations with AhR agonists TCDD and Picoberin. The solid ligand structures depict the poses assumed in the final simulation frames. **A:** Simulations performed with the AlphaFold protein model. **B and C:** Simulations performed with the homology model from SWISS-MODEL. **D:** Superposition of final frames from two MD simulations with TCDD and Picoberin, showing that the analysed ligands assume comparable positions within the binding pocket of the PAS-B domain.

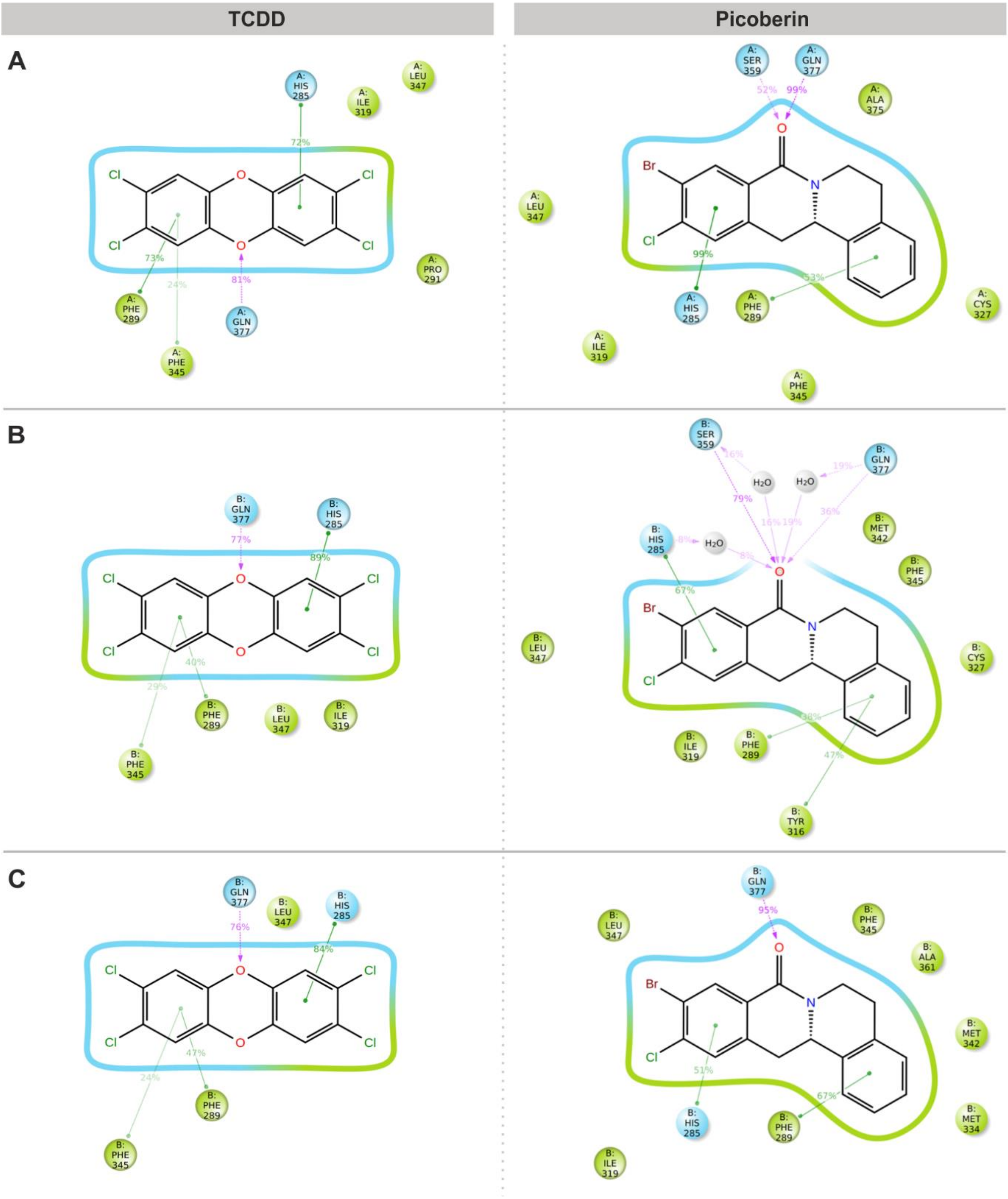


**Supporting Figure S12 (related to Figure 4): Schematic 2D representations of the TCDD- and Picoberin-AhR contacts.** Interactions occurring more than 5% of the simulation time during the final 20 ns of the simulations are shown. **A:** Data from simulation using the AlphaFold protein model. **B and C:** Data from simulations using the homology model obtained from SWISS-MODEL.

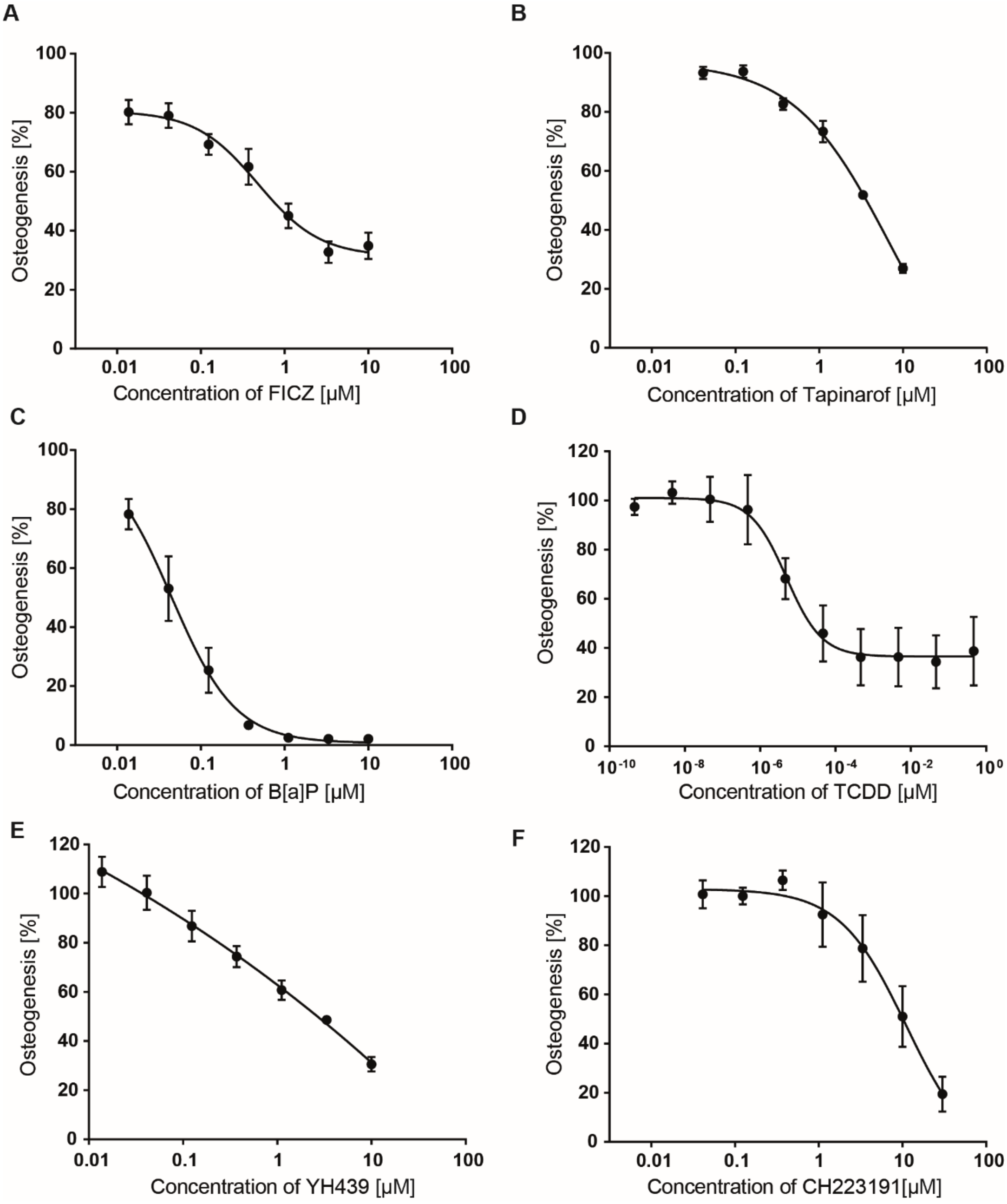


**Supporting Figure S13 (related to Figure 5): Influence of known AhR modulators on Hh-induced osteoblast differentiation. A:** FICZ, $IC_{50}$ = 510 ± 160 nM. **B:** Tapinarof, $IC_{50}$ ≈ 5.9 ± 0.2 µM. **C:** Benzo(a)pyrene (BaP), $IC_{50}$ = 47 ± 16 nM. **D:** TCDD, $IC_{50}$ = 5.2 ± 1.4 pM. **E:** YH439, $IC_{50}$ = 910 ± 250 nM. **F:** CH223191, $IC_{50}$ ≈ 9.4 ± 4.4 µM. C3H10T1/2 cells were treated with 1.5 µM purmorphamine to induce Hh signaling and thereby osteoblast differentiation and different concentrations of the compounds or DMSO as a control. After 96 h, alkaline phosphatase activity was determined by chemiluminescence readout. The obtained values of purmorphamine and compound treated samples were related to purmorphamine and DMSO treated samples. Values are mean values of three independent biological replicates and three technical replicates (N = 3, n = 3). To calculate $IC_{50}$ values, a non-linear regression was performed using a four-parameter fit using GraphPad Prism 7 (GraphPad Software, USA).

## Supporting Tables

**Table S1:** Influence of Picoberin on GPCR activities at a concentration of 500 nM in a GPCR panel run (GPCR MAX panel, Eurofins DiscoverX). Picoberin was tested in cell-based beta-arrestin enzyme fragment complementation (EFC) assays in agonist and antagonist mode.

| GPCR | % Activity | % Inhibition | GPCR | % Activity | % Inhibition |
|---|---|---|---|---|---|
| ADCYAP1R1 | 2 | 8 | GLP2R | 0 | -1 |
| ADORA3 | -3 | -10 | GPR1 | 0 | -5 |
| ADRA1B | -2 | 13 | GPR103 | -4 | -18 |
| ADRA2A | -1 | 8 | GPR109A | -3 | 6 |
| ADRA2B | -11 | 15 | GPR109B | -1 | 4 |
| ADRA2C | 4 | -13 | GPR119 | 2 | -8 |
| ADRB1 | -4 | 2 | GPR120 | -2 | 14 |
| ADRB2 | 0 | -12 | GPR35 | 0 | 17 |
| AGTR1 | 1 | 4 | GPR92 | 1 | 2 |
| AGTRL1 | -1 | -4 | GRPR | 0 | -4 |
| AVPR1A | -1 | 1 | HCRTR1 | 0 | -7 |
| AVPR1B | -2 | -3 | HCRTR2 | 0 | 1 |
| AVPR2 | 0 | 3 | HRH1 | -3 | -8 |
| BDKRB1 | -2 | -7 | HRH2 | -5 | -8 |
| BDKRB2 | 0 | 6 | HRH3 | -7 | -3 |
| BRS3 | 3 | 11 | HRH4 | -1 | -6 |
| C3AR1 | 0 | 5 | HTR1A | -2 | 3 |
| C5AR1 | 1 | -8 | HTR1B | 0 | 7 |
| C5L2 | 9 | -20 | HTR1E | 2 | -12 |
| CALCR | -2 | 6 | HTR1F | 1 | -1 |
| CALCRL-RAMP1 | 0 | 5 | HTR2A | 1 | 5 |
| CALCRL-RAMP2 | 2 | 5 | HTR2C | 2 | -3 |
| CALCRL-RAMP3 | 1 | 1 | HTR5A | 0 | 10 |
| CALCR-RAMP2 | 0 | 9 | KISS1R | 0 | 3 |
| CALCR-RAMP3 | 0 | -4 | LHCGR | -1 | 4 |
| CCKAR | -1 | -6 | LTB4R | 0 | 6 |
| CCKBR | -1 | -10 | MC1R | -3 | 19 |
| CCR10 | -1 | -3 | MC3R | -1 | 0 |
| CCR1 | -3 | 9 | MC4R | -1 | 4 |
| CCR2 | 0 | 10 | MC5R | -3 | -4 |
| CCR3 | -4 | 2 | MCHR1 | -1 | 5 |
| CCR4 | 0 | 8 | MCHR2 | -1 | -2 |
| CCR5 | 1 | -1 | MLNR | 0 | 3 |
| CCR6 | -1 | 3 | MRGPRX1 | 0 | 12 |
| CCR7 | 1 | 4 | MRGPRX2 | 0 | -3 |

| GPCR | % Activity | % Inhibition | GPCR | % Activity | % Inhibition |
|---|---|---|---|---|---|
| CCR8 | 0 | 1 | MTNR1A | -3 | 9 |
| CCR9 | 0 | -18 | NMBR | -4 | 8 |
| CHRM1 | -1 | 20 | NMU1R | 1 | -7 |
| CHRM2 | 0 | 3 | NPBWR1 | 1 | -17 |
| CHRM3 | 2 | -1 | NPBWR2 | -1 | 8 |
| CHRM4 | -11 | -10 | NPFFR1 | 2 | 25 |
| CHRM5 | 5 | -2 | NPSR1B | -6 | -1 |
| CMKLR1 | 0 | 10 | NPY1R | 5 | 3 |
| CNR1 | 0 | -5 | NPY2R | 0 | 1 |
| CNR2 | -28 | 24 | NTSR1 | 0 | 0 |
| CRHR1 | 0 | 6 | OPRD1 | -1 | -1 |
| CRHR2 | 0 | 2 | OPRK1 | -3 | 10 |
| CRTH2 | -1 | 1 | OPRL1 | -1 | 1 |
| CX3CR1 | -1 | 7 | OPRM1 | 1 | -3 |
| CXCR1 | 0 | 2 | OXER1 | -4 | 4 |
| CXCR2 | -3 | 5 | OXTR | 0 | 3 |
| CXCR3 | -4 | 9 | P2RY1 | 0 | -11 |
| **CXCR4** | 0 | **52** | P2RY11 | -5 | 6 |
| CXCR5 | 1 | 5 | P2RY12 | 2 | -10 |
| CXCR6 | 1 | -2 | P2RY2 | 2 | -3 |
| CXCR7 | -1 | -9 | P2RY4 | -4 | 7 |
| DRD1 | -1 | 3 | P2RY6 | 0 | -8 |
| DRD2L | 1 | -9 | PPYR1 | -1 | 13 |
| DRD2S | -1 | -5 | PRLHR | 1 | 26 |
| DRD3 | 8 | -3 | PROKR1 | 0 | 8 |
| DRD4 | -9 | -10 | PROKR2 | -1 | 28 |
| DRD5 | -1 | -12 | PTAFR | -2 | 11 |
| EBI2 | 1 | -15 | PTGER2 | 1 | 10 |
| EDG1 | 1 | 25 | PTGER3 | -3 | -6 |
| EDG3 | -2 | 6 | PTGER4 | 4 | 11 |
| EDG4 | 7 | -13 | PTGFR | 0 | -11 |
| EDG5 | -1 | -1 | PTGIR | -5 | -1 |
| EDG6 | -7 | -44 | PTHR1 | 0 | 9 |
| EDG7 | -2 | 19 | PTHR2 | 0 | 6 |
| EDNRA | 1 | -2 | RXFP3 | -3 | 21 |
| EDNRB | 0 | 0 | SCTR | 0 | 2 |
| F2R | -2 | 10 | SSTR1 | -12 | 6 |
| F2RL1 | 0 | -4 | SSTR2 | 0 | -11 |
| F2RL3 | -3 | 5 | SSTR3 | -1 | 2 |
| FFAR1 | 14 | 15 | SSTR5 | -4 | -2 |
| FPR1 | -2 | -20 | TACR1 | -1 | 1 |
| FPRL1 | 0 | 6 | TACR2 | 0 | 17 |
| FSHR | 0 | 6 | TACR3 | 0 | 11 |

| GPCR | % Activity | % Inhibition | GPCR | % Activity | % Inhibition |
|---|---|---|---|---|---|
| GALR1 | 0 | 1 | TBXA2R | -3 | 8 |
| GALR2 | -1 | 4 | TRHR | -1 | 5 |
| GCGR | 1 | 15 | TSHR(L) | -2 | 4 |
| GHSR | -3 | 3 | UTR2 | -7 | -3 |
| GIPR | -2 | 0 | VIPR1 | 0 | -3 |
| GLP1R | 0 | 2 | VIPR2 | -1 | 8 |

**Table S2:** Influence of Picoberin at a concentration of 500 nM in a nuclear receptor panel (nhr MAX panel, Eurofins DiscoverX). Activities of nuclear receptors were detected by means of cell-based nuclear hormone receptor assays in agonist and antagonist mode.

| Nuclear receptor | % Efficacy Agonist | % Efficacy Antagonist |
|---|---|---|
| AR | 0 .9 | 13 .5 |
| Era | -0 .6 | -28 .2 |
| FXR | 1 .1 | -10 .9 |
| GR | -0 .1 | 16 .6 |
| LXRa | -0 .5 | -3 .3 |
| LXRb | -2 .7 | 11 .6 |
| LXRB-NCOR1 | 7 .7 | -4 .6 |
| MR | 2 .1 | 1 .2 |
| PPARa | 4 | -23 |
| PPARd | 2 .5 | -14 .5 |
| PPARg | -2 .4 | -4 .1 |
| PRa | -6 | -21 .8 |
| PRb | -0 .3 | -0 .4 |
| RARa | -4 | 19 .7 |
| RARb | -14 | 15 .7 |
| RXRa | -2 .8 | 7 .2 |
| RXRg | -21 .7 | 34 .5 |
| THRa | 7 .3 | -6 .9 |
| THRb | 4 .8 | 7 .8 |

**Table S3 (related to Figure 4):** Key results of the model quality analysis for the AhR PAS-B structures.

| | Template | Residues | ProSA Z-Score | QMEANDisCo Global Score (±0.08) | PROCHECK Ramachandran (%) | | | | PROCHECK Overall G-factor |
|---|---|---|---|---|---|---|---|---|---|
| | | | | | Core | Allowed | Generous | Dis-allowed | |
| Alpha Fold | - | 276-380 | -4.72 (within experimental range) | 0.70 | 95.7 | 4.3 | 0.0 | 0.0 | 0.05 |
| Swiss-Model | 4f3l.1.B | 276-380 | -4.15 (within experimental range) | 0.70 | 91.3 | 7.6 | 1.1 | 0.0 | -0.08 |

**Table S4 (related to Figure 4):** RMSD values between reference HIF2α crystal structures and AhR PAS-B models.

| | **RMSD (Å)** | |
|---|---|---|
| Reference HIF2α X-ray Structure | **AhR AlphaFold Model** | **AhR Swiss-Model** |
| 3f1n | 0.834 | 0.672 |
| 3f1o | 0.943 | 0.684 |
| 3f1p | 0.889 | 0.674 |
| 3h7w | 0.860 | 0.706 |
| 3h82 | 1.054 | 0.808 |
| 4ghi | 0.849 | 0.714 |
| 4gs9 | 0.884 | 0.675 |
| 4xt2 | 1.078 | 0.826 |
| 5tbm | 0.969 | 0.663 |
| 5ufp | 0.944 | 0.601 |
| 6czw | 0.974 | 0.680 |
| 6d09 | 0.952 | 0.619 |
| 6d0b | 0.996 | 0.731 |
| 6d0c | 0.896 | 0.652 |
| 4zp4 | 0.975 | 0.787 |
| 4zph | 0.918 | 0.788 |
| 4zqd | 0.979 | 0.756 |
| 6e3u | 0.928 | 0.772 |

# NMR and HPLC spectra

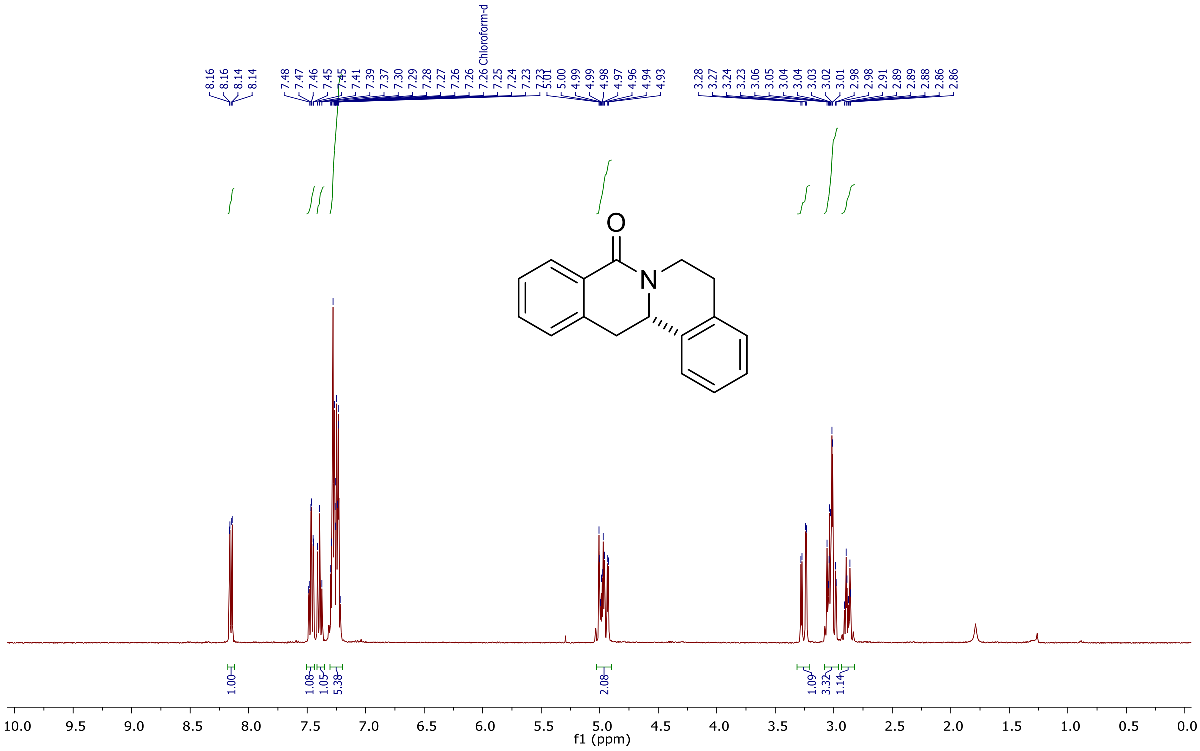

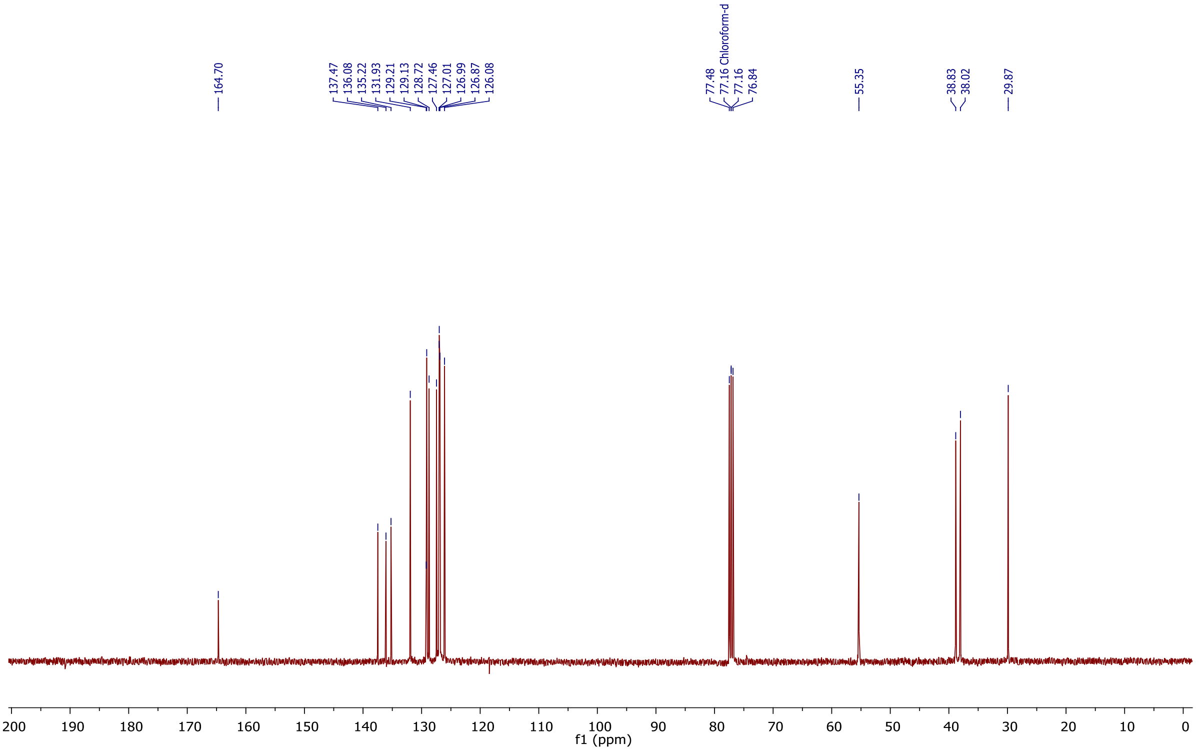

HPLC traces (**3a**): racemate top, enantiomer bottom:

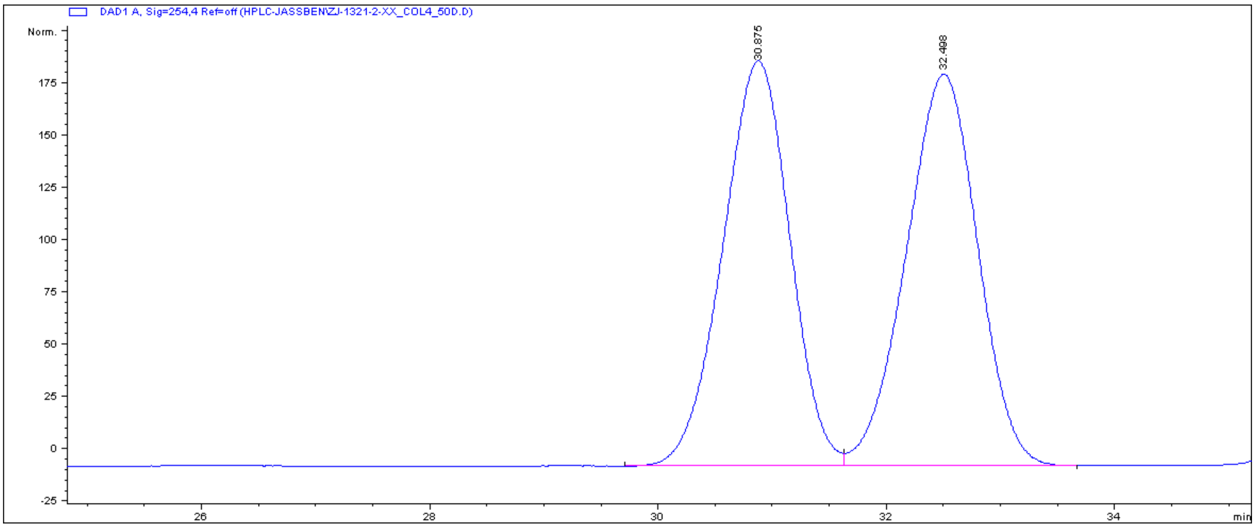


| Peak # | RetTime [min] | Type | Width [min] | Area [mAU*s] | Height [mAU] | Area % |
|---|---|---|---|---|---|---|
| 1 | 30.873 | BV | 0.6178 | 2855.87402 | 69.73338 | 48.9795 |
| 2 | 32.496 | VB | 0.6612 | 2974.87866 | 67.89524 | 51.0205 |
| Totals : | | | | 5830.75269 | 137.62862 | |

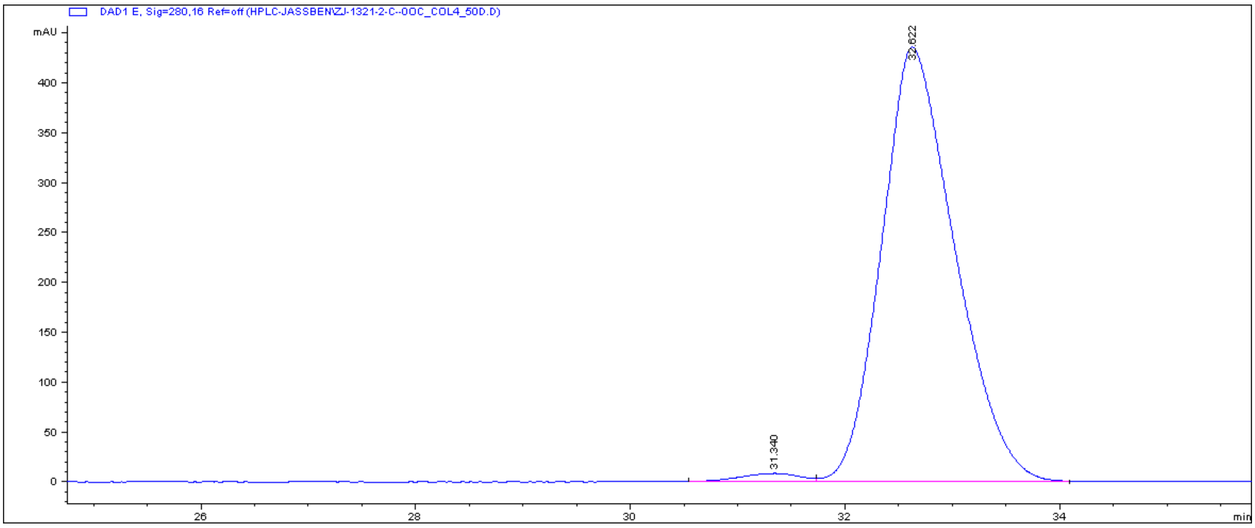


| Peak # | RetTime [min] | Type | Width [min] | Area [mAU*s] | Height [mAU] | Area % |
|---|---|---|---|---|---|---|
| 1 | 31.340 | BV | 0.4589 | 312.41339 | 8.19193 | 1.4889 |
| 2 | 32.622 | VB | 0.6879 | 2.06702e4 | 435.39661 | 98.5111 |
| Totals : | | | | 2.09826e4 | 443.58853 | |

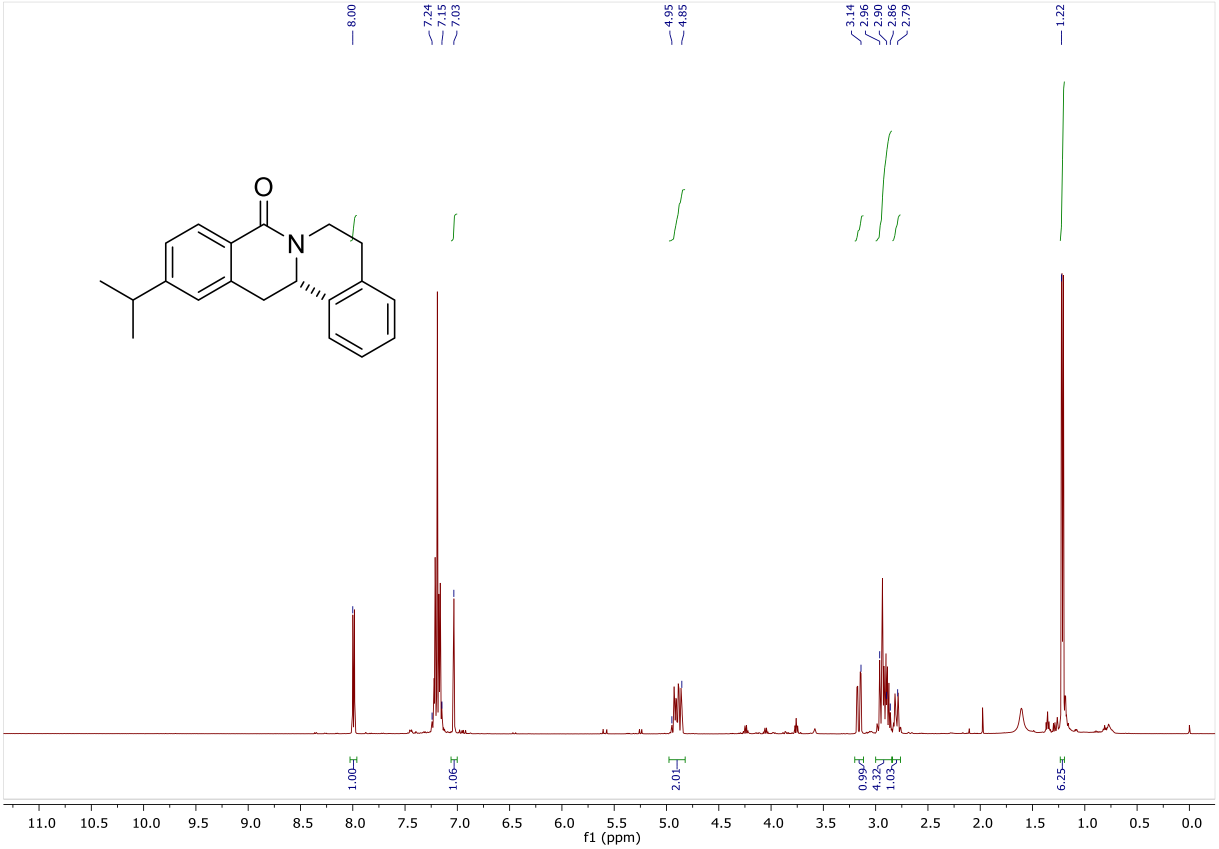
8.00
7.24
7.15
7.03
4.95
4.85
3.14
2.96
2.90
2.86
2.79
1.22
1.00
1.06
2.01
0.99
4.32
1.03
6.25
O
N
f1 (ppm)

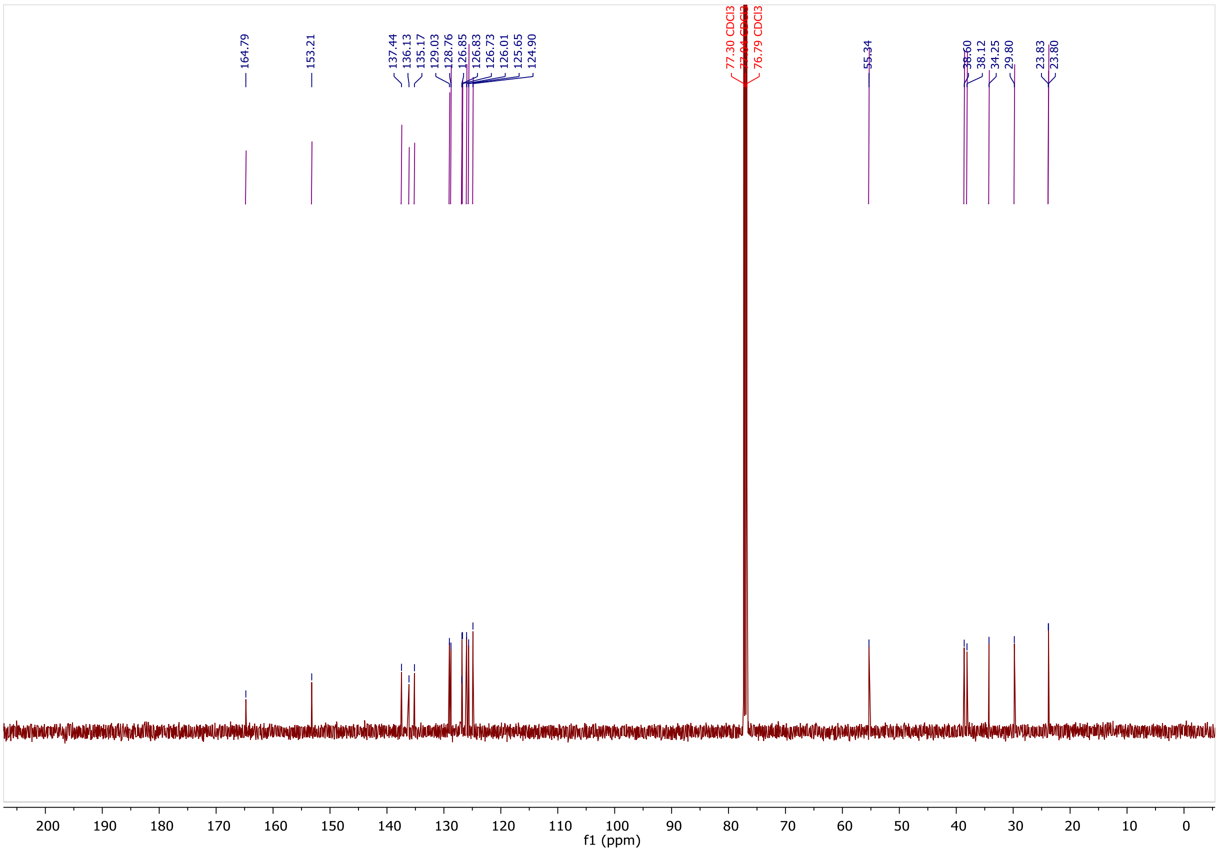
164.79
153.21
137.44
136.13
135.17
129.03
128.76
126.85
126.83
126.73
126.01
125.65
124.90
77.30 CDCl3
76.79 CDCl3
55.34
38.50
38.12
34.25
29.80
23.83
23.80
f1 (ppm)

HPLC traces (**3b**): racemate top, enantiomer bottom:

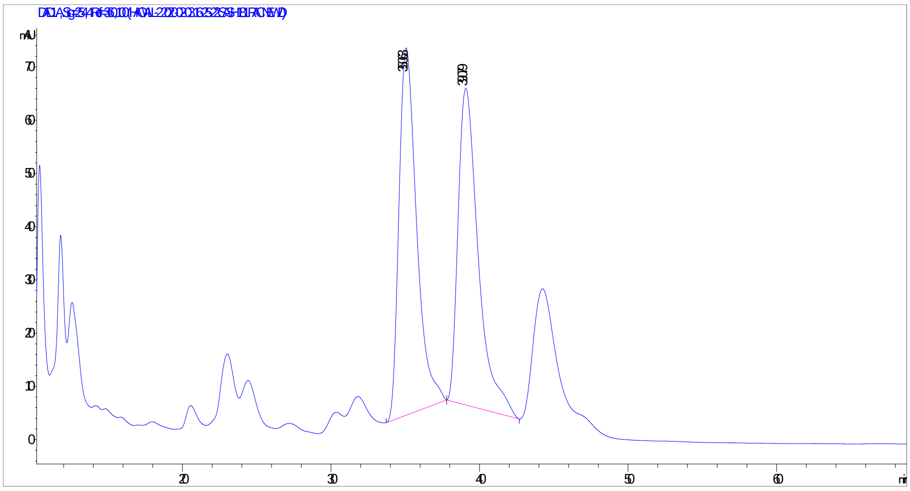


| # | Time | Area | Height | Width | Area% | Symmetry |
|---|---|---|---|---|---|---|
| 1 | 35.063 | 5711.5 | 69 | 1.2581 | 50.438 | 0.637 |
| 2 | 39.079 | 5612.4 | 59.5 | 1.4146 | 49.562 | 0.569 |

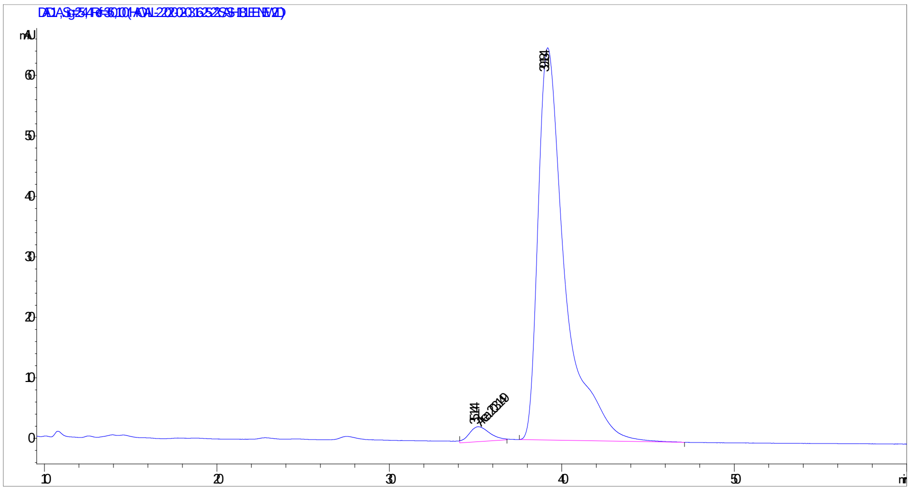


| # | Time | Area | Height | Width | Area% | Symmetry |
|---|---|---|---|---|---|---|
| 1 | 35.144 | 183.9 | 2.4 | 1.2727 | 2.629 | 0.795 |
| 2 | 39.184 | 6812.1 | 64.4 | 1.7624 | 97.371 | 0.547 |

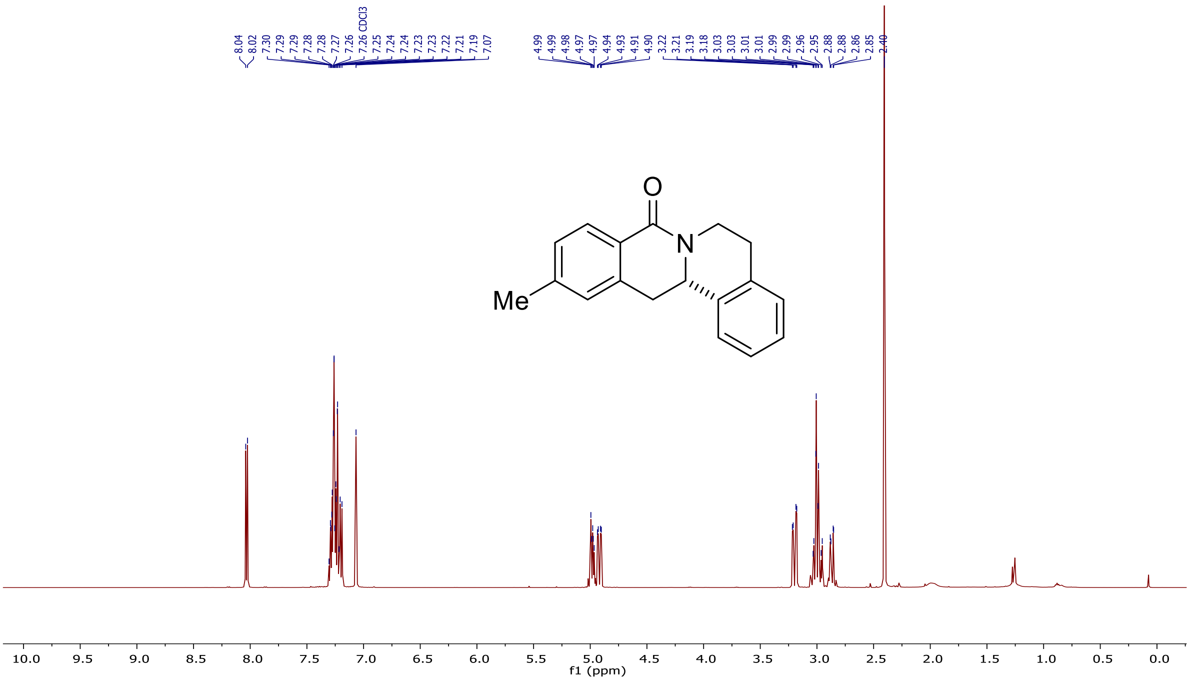
8.04
8.02
7.30
7.29
7.29
7.28
7.28
7.27
7.26 CDCl3
7.25
7.24
7.24
7.23
7.23
7.23
7.22
7.21
7.19
7.07
4.99
4.99
4.98
4.97
4.97
4.94
4.93
4.91
4.90
3.22
3.21
3.19
3.18
3.03
3.03
3.01
3.01
2.99
2.99
2.96
2.95
2.88
2.88
2.86
2.85
2.40
O
N
Me
f1 (ppm)

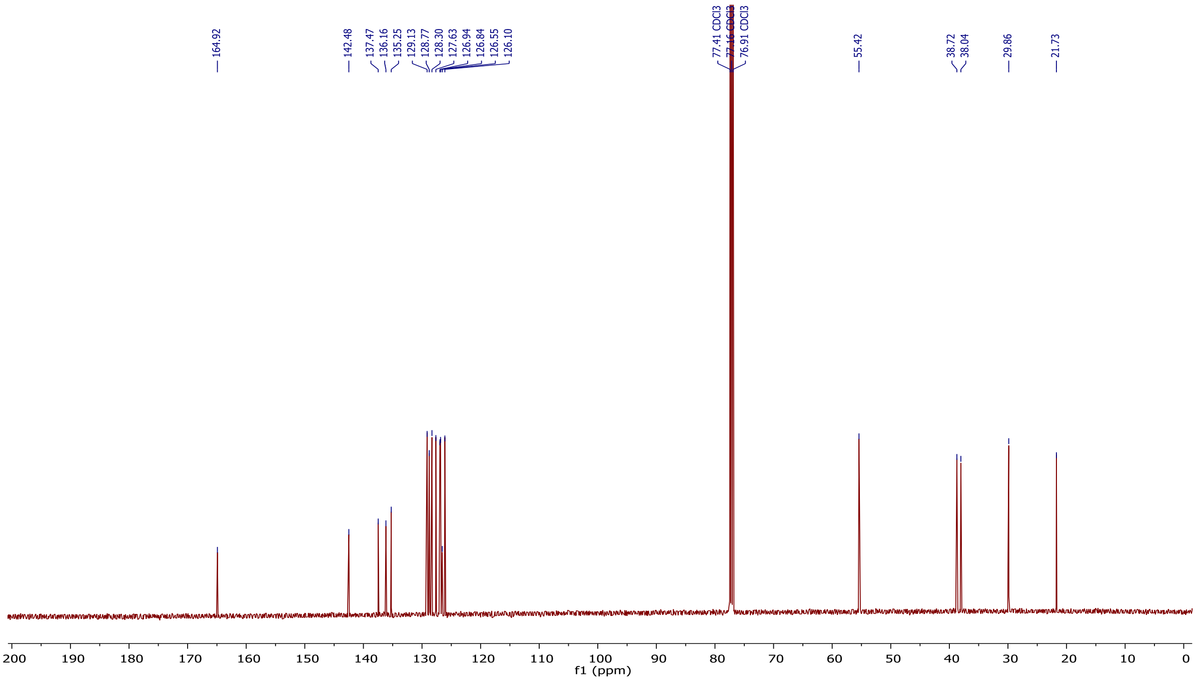
164.92
142.48
137.47
136.16
135.25
129.13
128.77
128.30
127.63
126.94
126.84
126.55
126.10
77.41 CDCl3
77.16 CDCl3
76.91 CDCl3
55.42
38.72
38.04
29.86
21.73
f1 (ppm)

HPLC traces (**3c**): racemate top, enantiomer bottom:

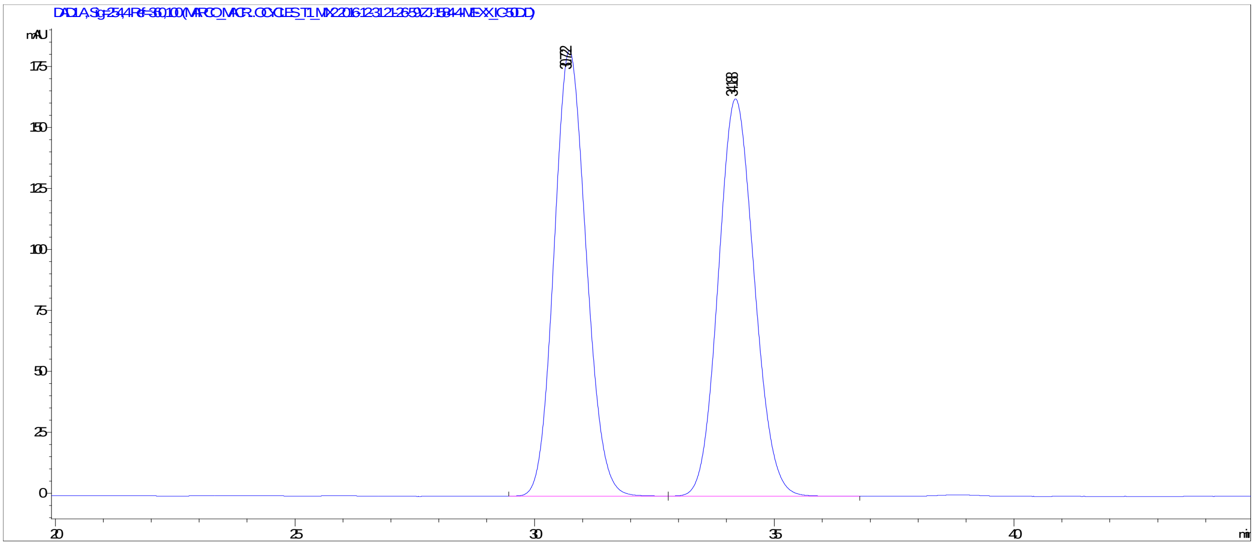

Signal 1: DAD1 A, Sig=254,4 Ref=360,100

| Peak # | RetTime [min] | Type | Width [min] | Area [mAU*s] | Height [mAU] | Area % |
|---|---|---|---|---|---|---|
| 1 | 30.722 | BB | 0.7234 | 8512.03906 | 182.73959 | 49.9837 |
| 2 | 34.188 | BB | 0.8120 | 8517.58398 | 162.89903 | 50.0163 |

Totals : 1.70296e4 345.63863

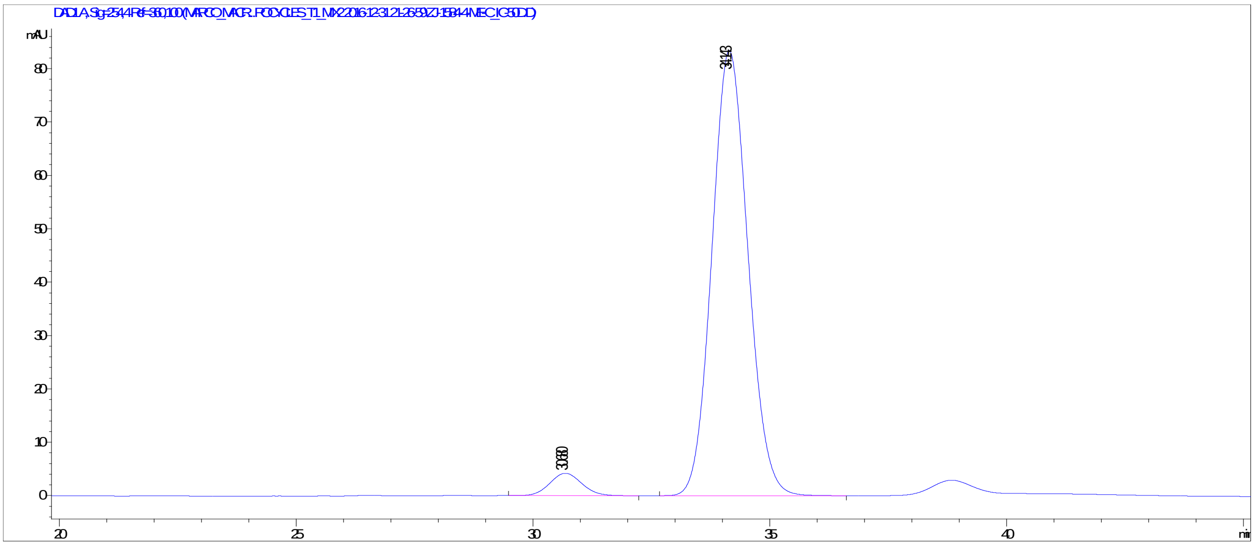

Signal 1: DAD1 A, Sig=254,4 Ref=360,100

| Peak # | RetTime [min] | Type | Width [min] | Area [mAU*s] | Height [mAU] | Area % |
|---|---|---|---|---|---|---|
| 1 | 30.680 | BB | 0.7240 | 198.98393 | 4.20552 | 4.3148 |
| 2 | 34.143 | BB | 0.8250 | 4412.63672 | 83.44163 | 95.6852 |

Totals : 4611.62065 87.64714

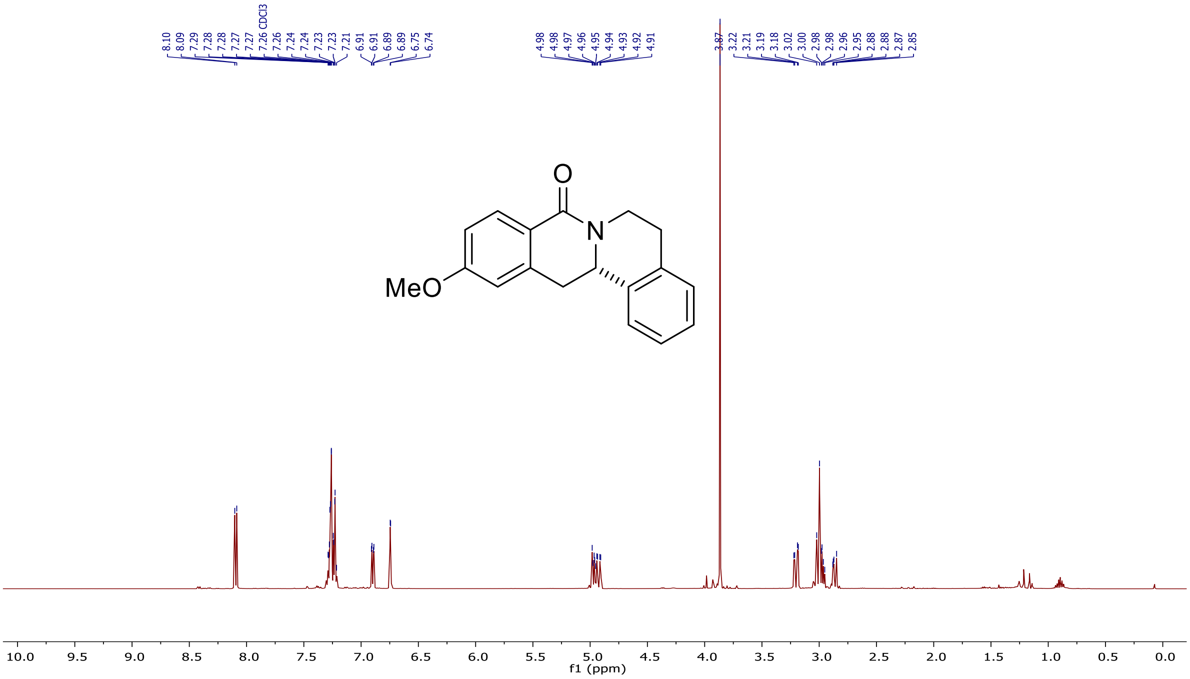
8.10 8.09 7.29 7.28 7.27 7.27 7.26 CDCl3 7.26 7.24 7.24 7.23 7.23 7.21 6.91 6.91 6.89 6.89 6.75 6.74
4.98 4.98 4.97 4.96 4.95 4.94 4.93 4.92 4.91
3.87 3.22 3.21 3.19 3.18 3.02 3.00 2.98 2.98 2.96 2.95 2.88 2.88 2.87 2.85
O
N
MeO
10.0 9.5 9.0 8.5 8.0 7.5 7.0 6.5 6.0 5.5 5.0 4.5 4.0 3.5 3.0 2.5 2.0 1.5 1.0 0.5 0.0
f1 (ppm)

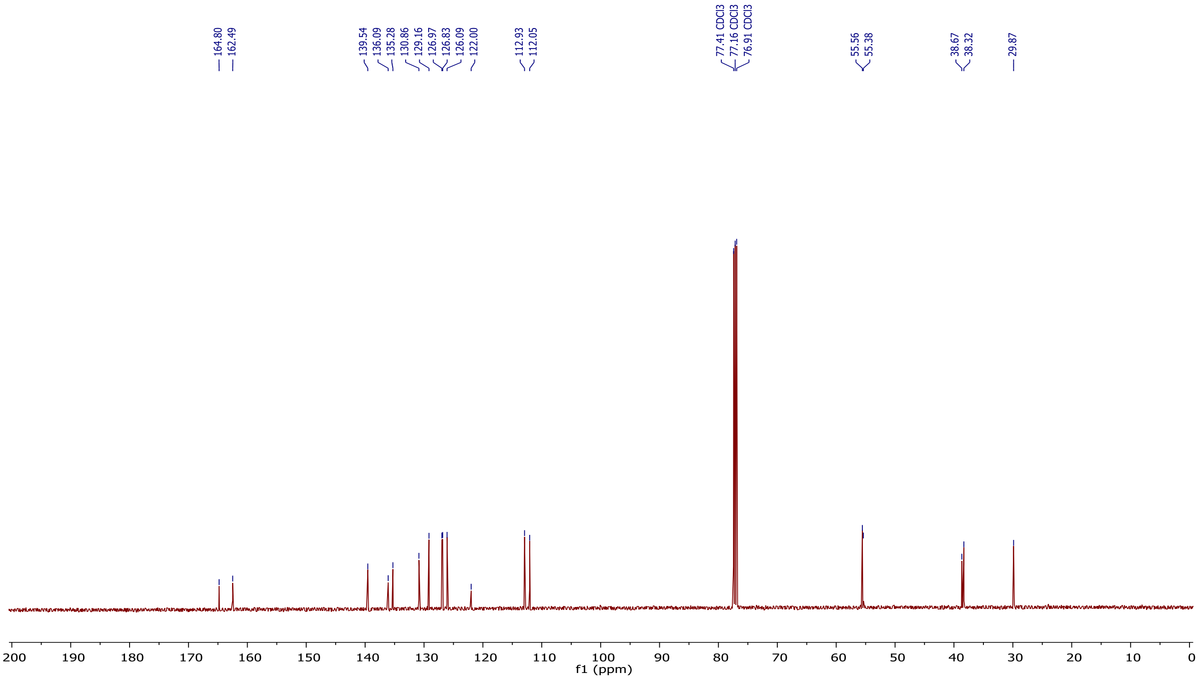
164.80 162.49
139.54 136.09 135.28 130.86 129.16 126.97 126.83 126.09 122.00
112.93 112.05
77.41 CDCl3 77.16 CDCl3 76.91 CDCl3
55.56 55.38
38.67 38.32
29.87
200 190 180 170 160 150 140 130 120 110 100 90 80 70 60 50 40 30 20 10 0
f1 (ppm)

HPLC traces (**3d**): racemate top, enantiomer bottom:

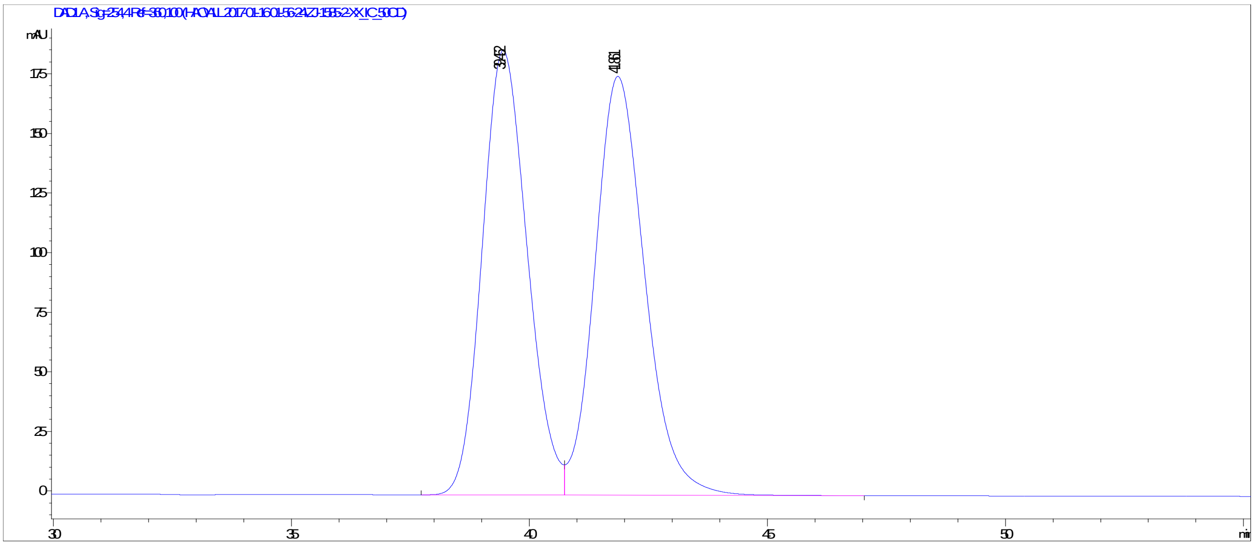

Signal 1: DAD1 A, Sig=254,4 Ref=360,100

| Peak # | RetTime [min] | Type | Width [min] | Area [mAU*s] | Height [mAU] | Area % |
|---|---|---|---|---|---|---|
| 1 | 39.452 | BV | 1.0247 | 1.22893e4 | 186.52448 | 48.9814 |
| 2 | 41.861 | VB | 1.1239 | 1.28004e4 | 175.71353 | 51.0186 |
| Totals : | | | | 2.50898e4 | 362.23801 | |

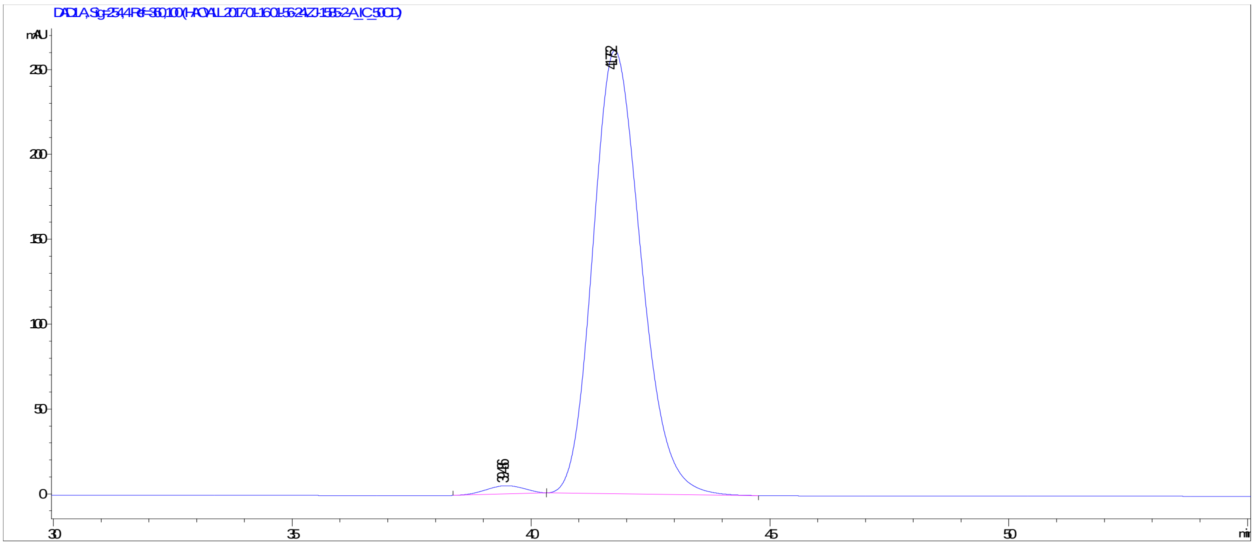

Signal 1: DAD1 A, Sig=254,4 Ref=360,100

| Peak # | RetTime [min] | Type | Width [min] | Area [mAU*s] | Height [mAU] | Area % |
|---|---|---|---|---|---|---|
| 1 | 39.486 | BB | 0.8612 | 265.90454 | 4.85366 | 1.4345 |
| 2 | 41.752 | BB | 1.0878 | 1.82703e4 | 261.27133 | 98.5655 |
| Totals : | | | | 1.85362e4 | 266.12499 | |

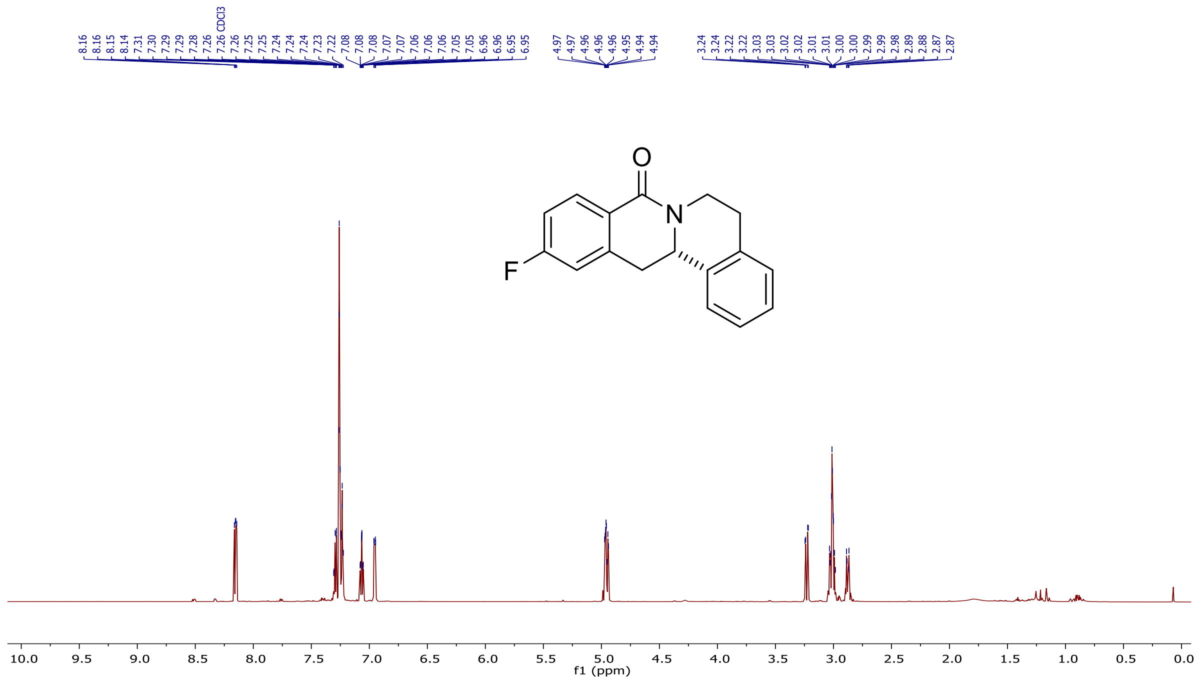
8.16
8.16
8.15
8.14
7.31
7.30
7.29
7.29
7.28
7.26
7.26 CDCl3
7.26
7.25
7.25
7.24
7.24
7.24
7.23
7.22
7.08
7.08
7.08
7.07
7.07
7.06
7.06
7.06
7.05
7.05
6.96
6.96
6.95
6.95
4.97
4.97
4.96
4.96
4.96
4.95
4.94
4.94
3.24
3.24
3.22
3.22
3.03
3.03
3.02
3.02
3.01
3.01
3.00
3.00
2.99
2.99
2.98
2.89
2.88
2.87
2.87
O
N
F
10.0 9.5 9.0 8.5 8.0 7.5 7.0 6.5 6.0 5.5 5.0 4.5 4.0 3.5 3.0 2.5 2.0 1.5 1.0 0.5 0.0
f1 (ppm)

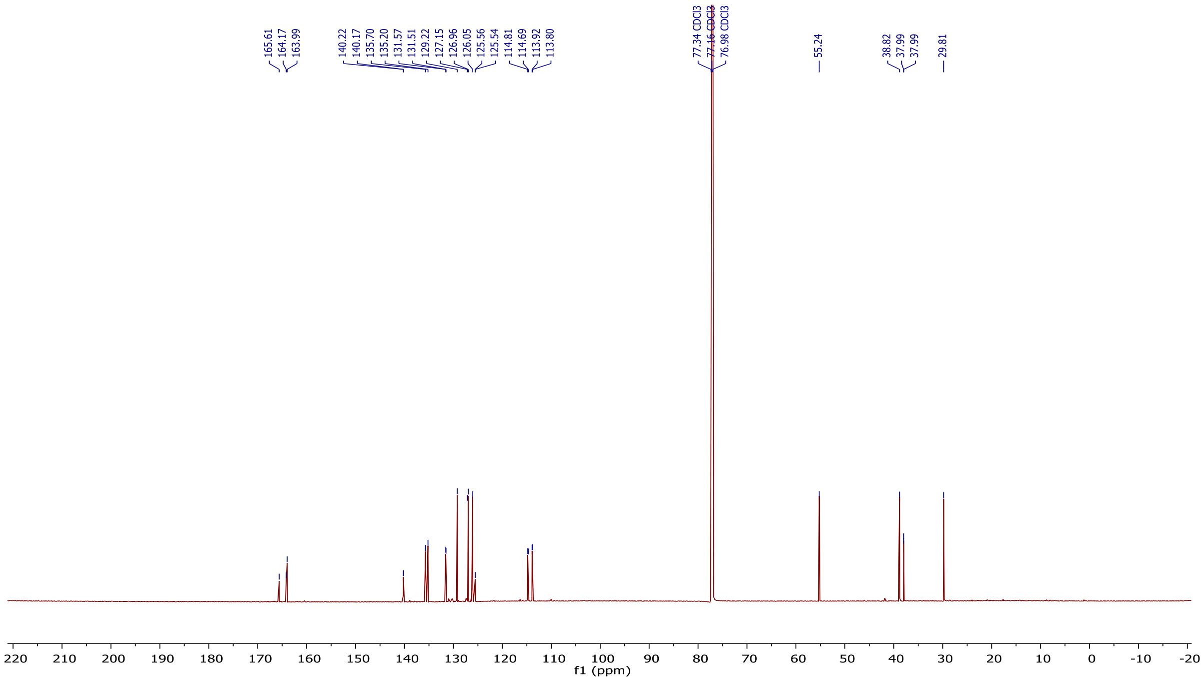
165.61
164.17
163.99
140.22
140.17
135.70
135.20
131.57
131.51
129.22
127.15
126.96
126.05
125.56
125.54
114.81
114.69
113.92
113.80
77.34 CDCl3
77.16 CDCl3
76.98 CDCl3
55.24
38.82
37.99
37.99
29.81
220 210 200 190 180 170 160 150 140 130 120 110 100 90 80 70 60 50 40 30 20 10 0 -10 -20
f1 (ppm)

HPLC traces (**3e**): racemate top, enantiomer bottom:

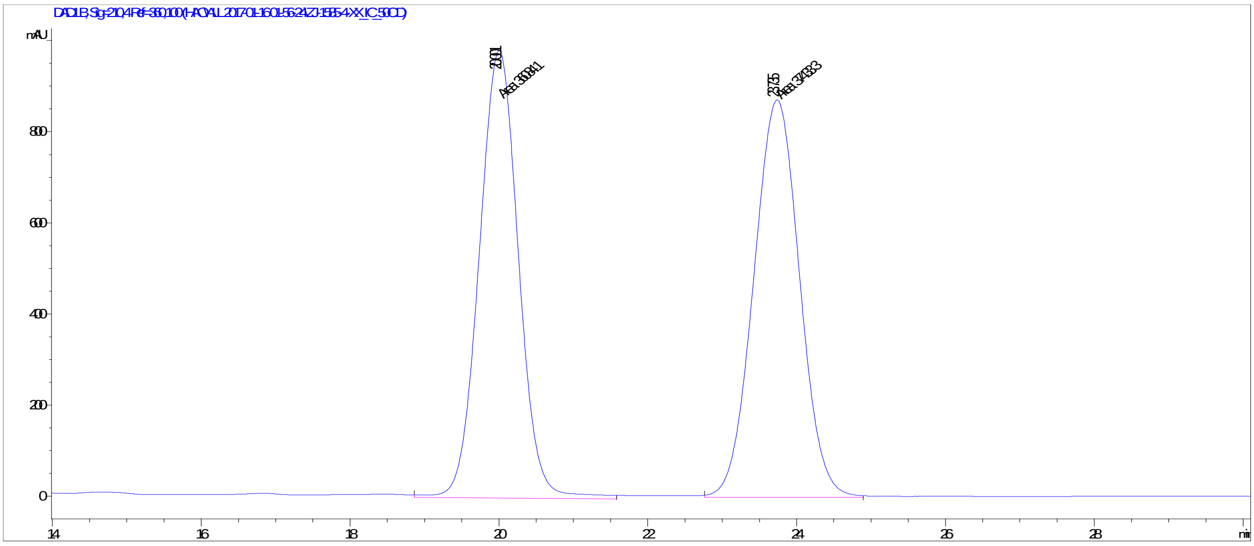

Signal 2: DAD1 B, Sig=210,4 Ref=360,100

| Peak # | RetTime [min] | Type | Width [min] | Area [mAU*s] | Height [mAU] | Area % |
|---|---|---|---|---|---|---|
| 1 | 20.001 | MM | 0.5991 | 3.52405e4 | 980.36365 | 48.8289 |
| 2 | 23.735 | MM | 0.7082 | 3.69309e4 | 869.11945 | 51.1711 |
| Totals : | | | | 7.21714e4 | 1849.48309 | |

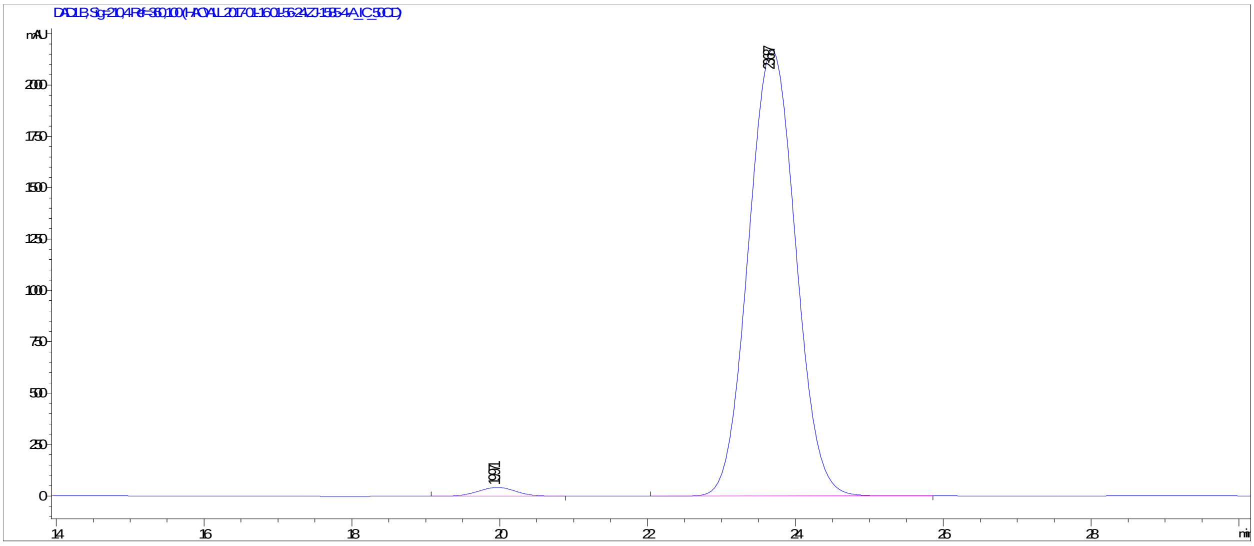

Signal 2: DAD1 B, Sig=210,4 Ref=360,100

| Peak # | RetTime [min] | Type | Width [min] | Area [mAU*s] | Height [mAU] | Area % |
|---|---|---|---|---|---|---|
| 1 | 19.971 | BB | 0.5556 | 1513.12476 | 42.67817 | 1.5468 |
| 2 | 23.687 | BB | 0.6979 | 9.63090e4 | 2169.55884 | 98.4532 |
| Totals : | | | | 9.78221e4 | 2212.23701 | |

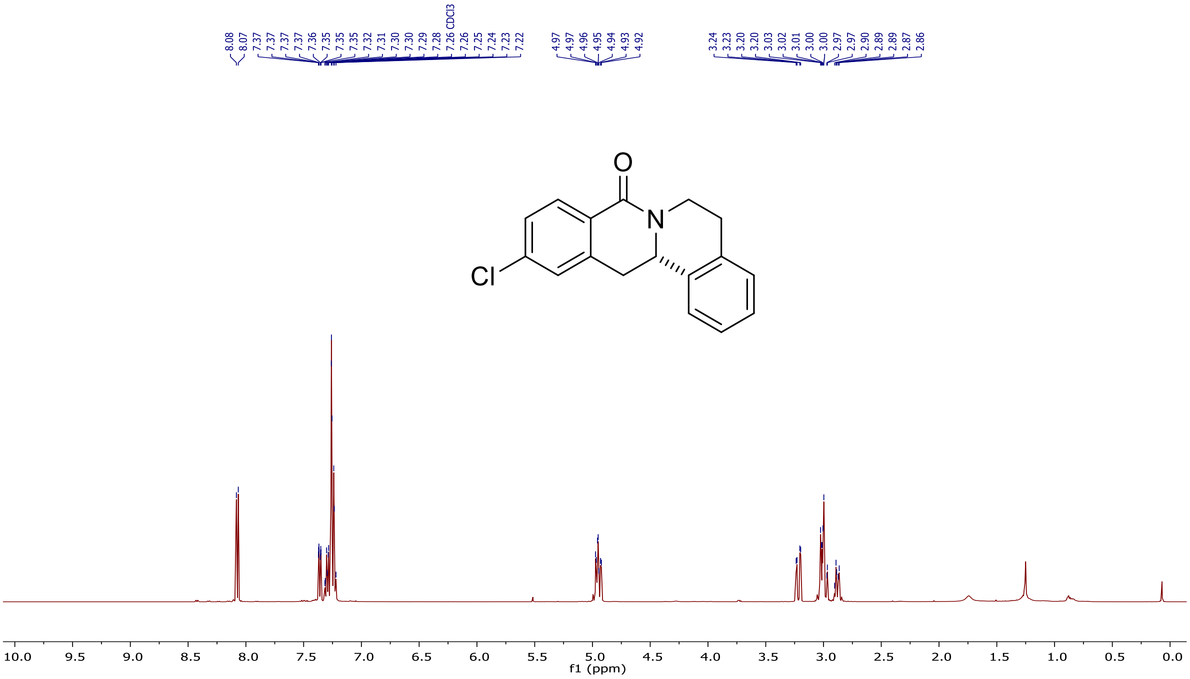
8.08
8.07
7.37
7.37
7.37
7.37
7.36
7.35
7.35
7.35
7.32
7.31
7.30
7.30
7.29
7.28
7.26 CDCl3
7.26
7.25
7.24
7.23
7.22
4.97
4.97
4.96
4.95
4.94
4.93
4.92
3.24
3.23
3.20
3.20
3.03
3.02
3.01
3.00
3.00
2.97
2.97
2.90
2.89
2.89
2.87
2.86
O
N
Cl
f1 (ppm)

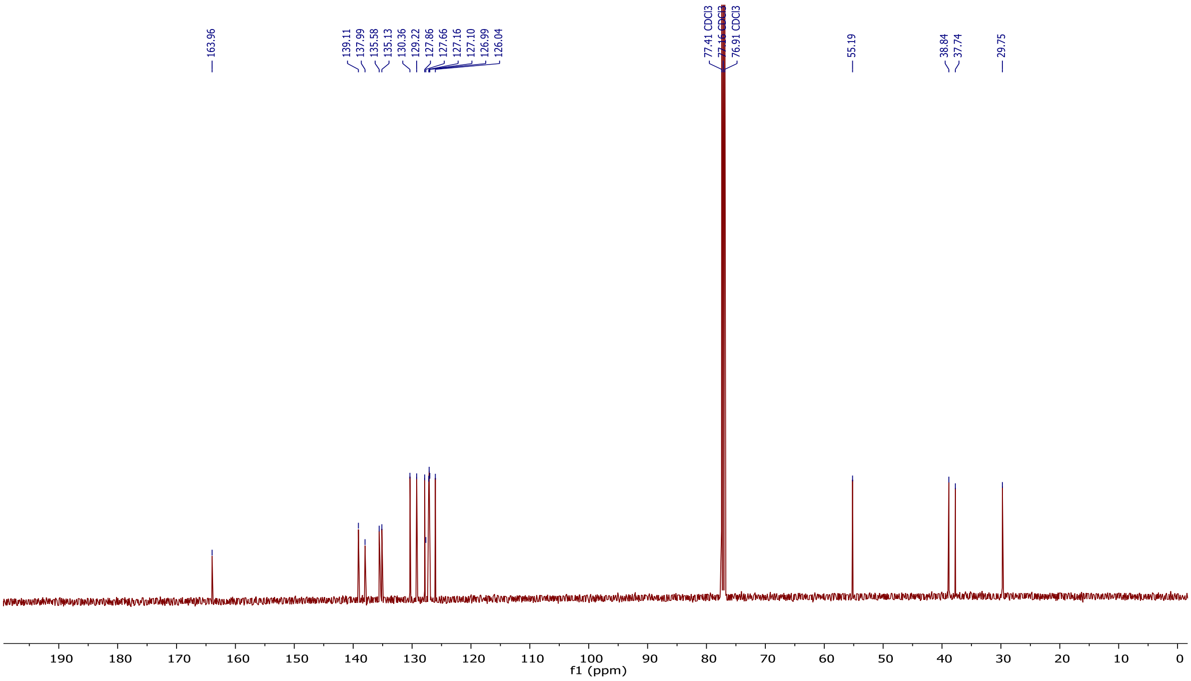
163.96
139.11
137.99
135.58
135.13
130.36
129.22
127.86
127.66
127.16
127.10
126.99
126.04
77.41 CDCl3
77.16 CDCl3
76.91 CDCl3
55.19
38.84
37.74
29.75
f1 (ppm)

HPLC traces (**3f**): racemate top, enantiomer bottom:

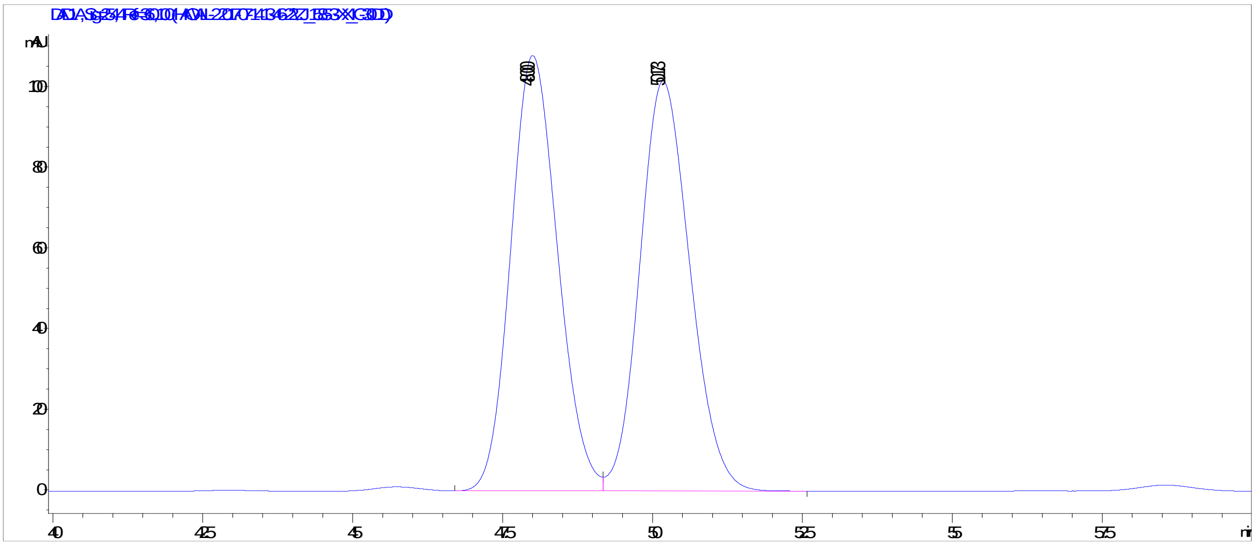

Signal 1: DAD1 A, Sig=254,4 Ref=360,100

| Peak # | RetTime [min] | Type | Width [min] | Area [mAU*s] | Height [mAU] | Area % |
|---|---|---|---|---|---|---|
| 1 | 47.750 | BV | 0.8434 | 6803.42383 | 125.70148 | 49.8158 |
| 2 | 49.825 | VB | 0.8996 | 6853.73340 | 118.00489 | 50.1842 |

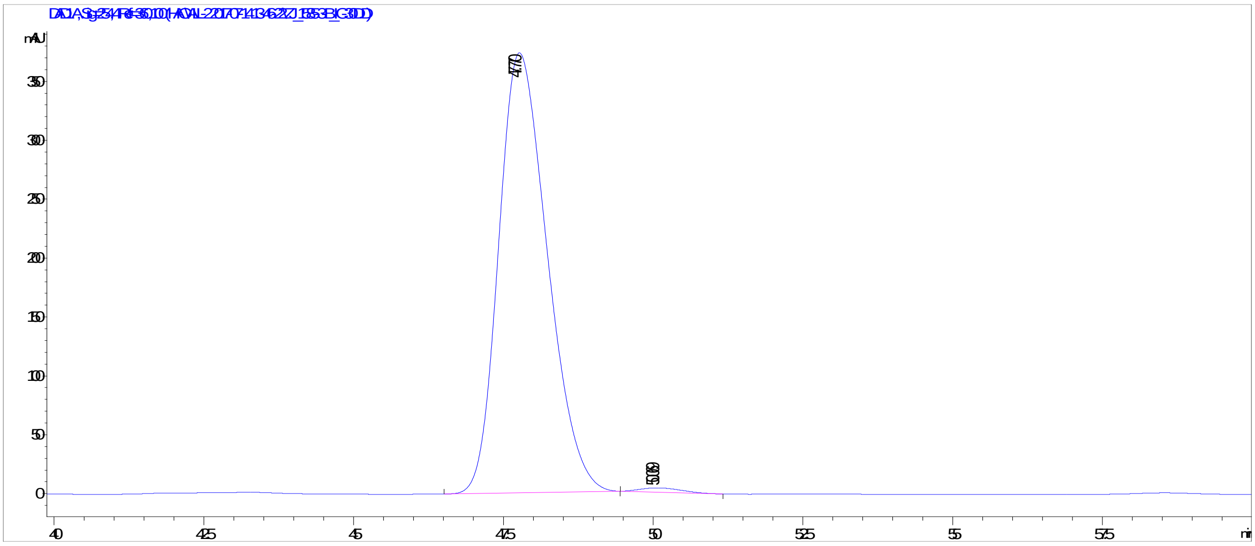

Signal 1: DAD1 A, Sig=254,4 Ref=360,100

| Peak # | RetTime [min] | Type | Width [min] | Area [mAU*s] | Height [mAU] | Area % |
|---|---|---|---|---|---|---|
| 1 | 47.770 | BB | 0.8740 | 2.10647e4 | 373.50006 | 99.1792 |
| 2 | 50.069 | BB | 0.7027 | 174.32448 | 3.69322 | 0.8208 |

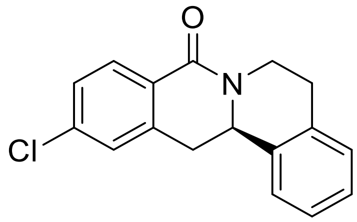

HPLC traces (**3f'**): racemate top, enantiomer bottom:

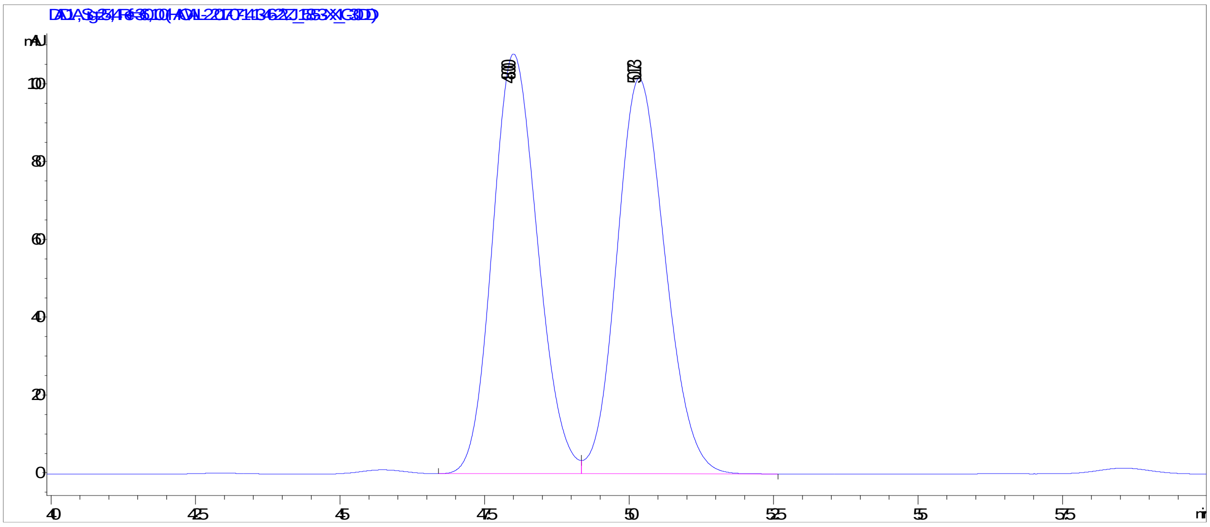

Signal 1: DAD1 A, Sig=254,4 Ref=360,100

| Peak # | RetTime [min] | Type | Width [min] | Area [mAU*s] | Height [mAU] | Area % |
|---|---|---|---|---|---|---|
| 1 | 47.750 | BV | 0.8434 | 6803.42383 | 125.70148 | 49.8158 |
| 2 | 49.825 | VB | 0.8996 | 6853.73340 | 118.00489 | 50.1842 |

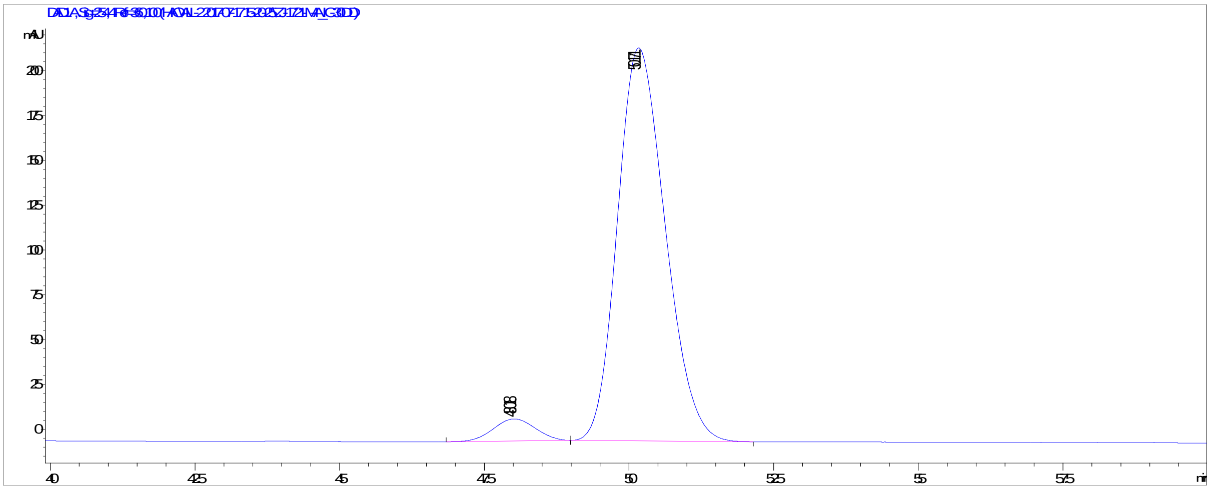

Signal 1: DAD1 A, Sig=254,4 Ref=360,100

| Peak # | RetTime [min] | Type | Width [min] | Area [mAU*s] | Height [mAU] | Area % |
|---|---|---|---|---|---|---|
| 1 | 48.018 | BB | 0.7606 | 620.27228 | 12.33708 | 4.6994 |
| 2 | 50.171 | BB | 0.8895 | 1.25787e4 | 219.20389 | 95.3006 |

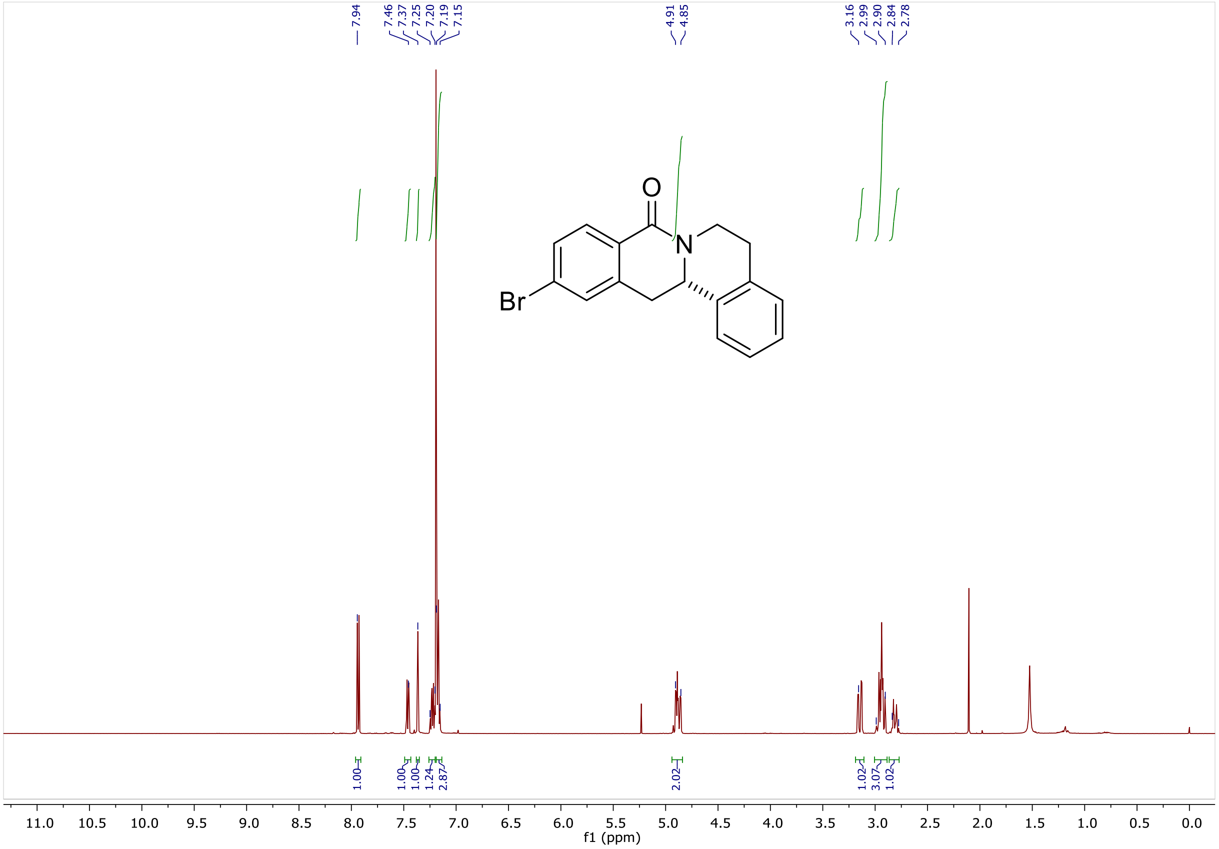
7.94
7.46
7.37
7.25
7.20
7.19
7.15
4.91
4.85
3.16
2.99
2.90
2.84
2.78
O
N
Br
1.00
1.00
1.00
1.24
2.87
2.02
1.02
3.07
1.02
f1 (ppm)

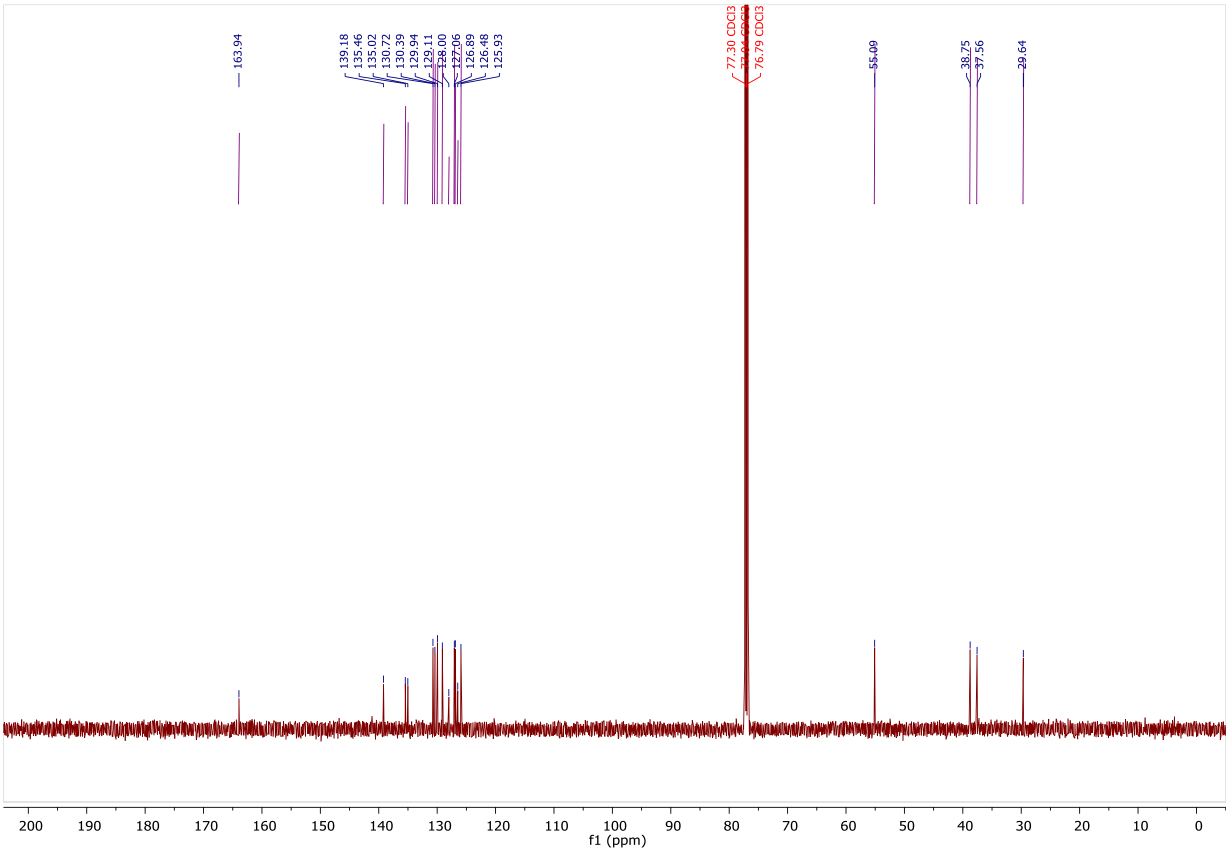
163.94
139.18
135.46
135.02
130.72
130.39
129.94
129.11
128.00
127.06
126.89
126.48
125.93
77.30 CDCl3
76.79 CDCl3
55.09
38.75
37.56
29.64
f1 (ppm)

HPLC traces (**3g**): racemate top, enantiomer bottom:

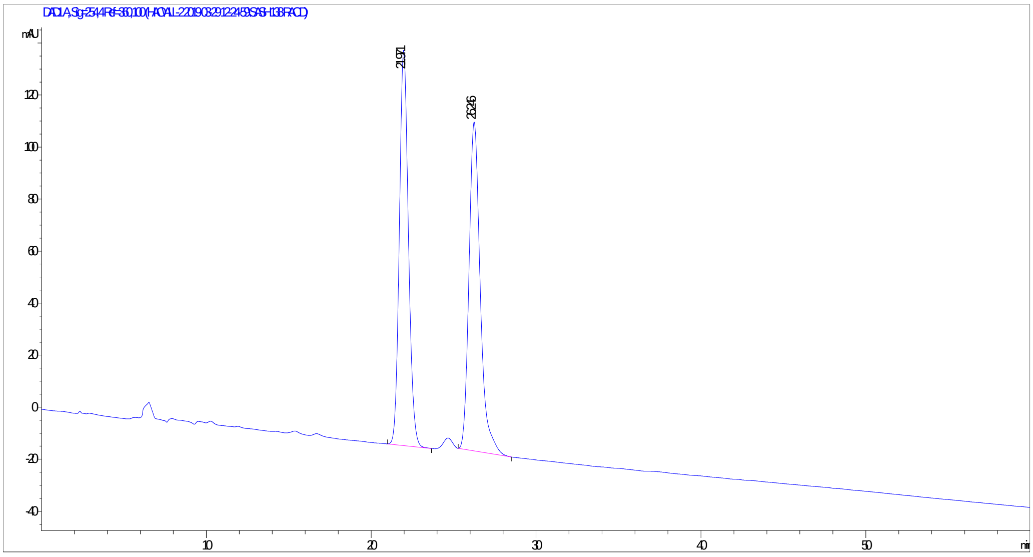


| # | Time | Area | Height | Width | Area% | Symmetry |
|---|---|---|---|---|---|---|
| 1 | 21.971 | 5869.2 | 152.6 | 0.6412 | 49.531 | 0.88 |
| 2 | 26.246 | 5980.3 | 126.4 | 0.7884 | 50.469 | 0.802 |

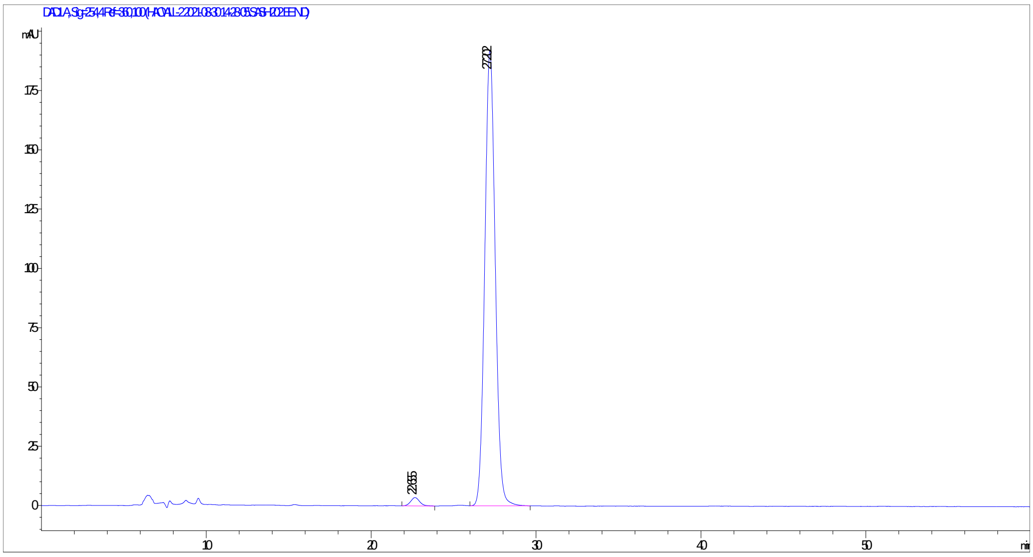


| # | Time | Area | Height | Width | Area% | Symmetry |
|---|---|---|---|---|---|---|
| 1 | 22.655 | 134.1 | 3.5 | 0.5159 | 1.563 | 0.859 |
| 2 | 27.202 | 8443 | 192.1 | 0.6828 | 98.437 | 0.909 |

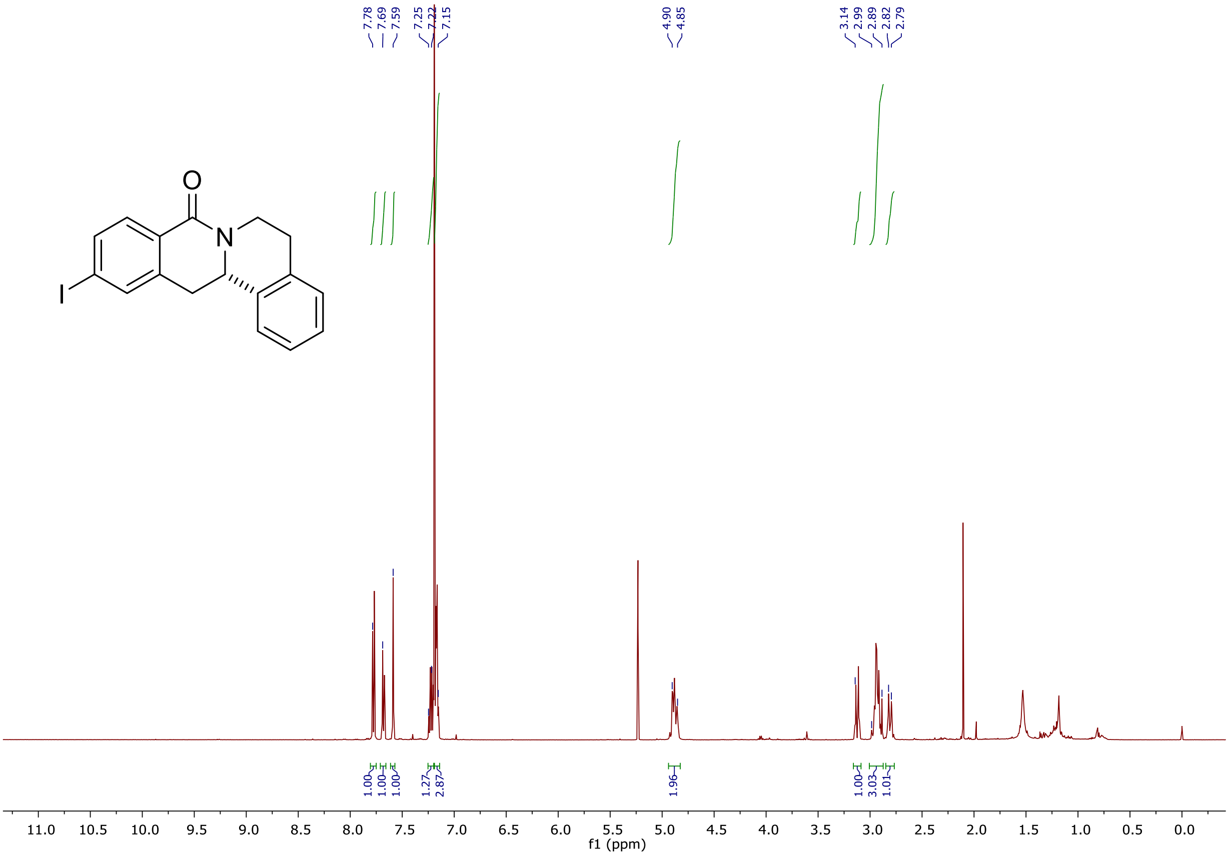

7.78
7.69
7.59
7.25
7.22
7.15
4.90
4.85
3.14
2.99
2.89
2.82
2.79
1.00
1.00
1.00
1.27
2.87
1.96
1.00
3.03
1.01
f1 (ppm)


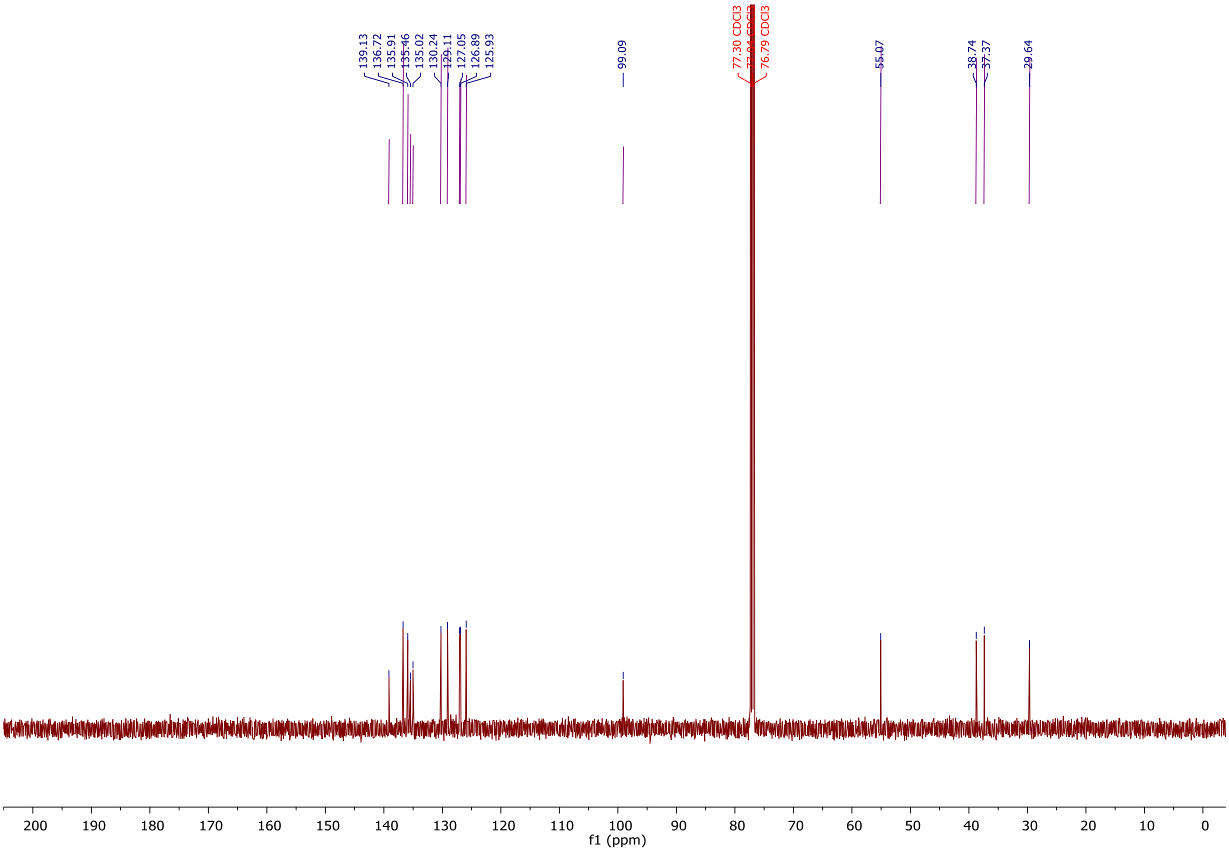

139.13
136.72
135.91
135.46
135.02
130.24
129.11
127.05
126.89
125.93
99.09
77.30 CDCl3
76.79 CDCl3
55.07
38.74
37.37
29.64
f1 (ppm)

HPLC traces (**3h**): racemate top, enantiomer bottom:

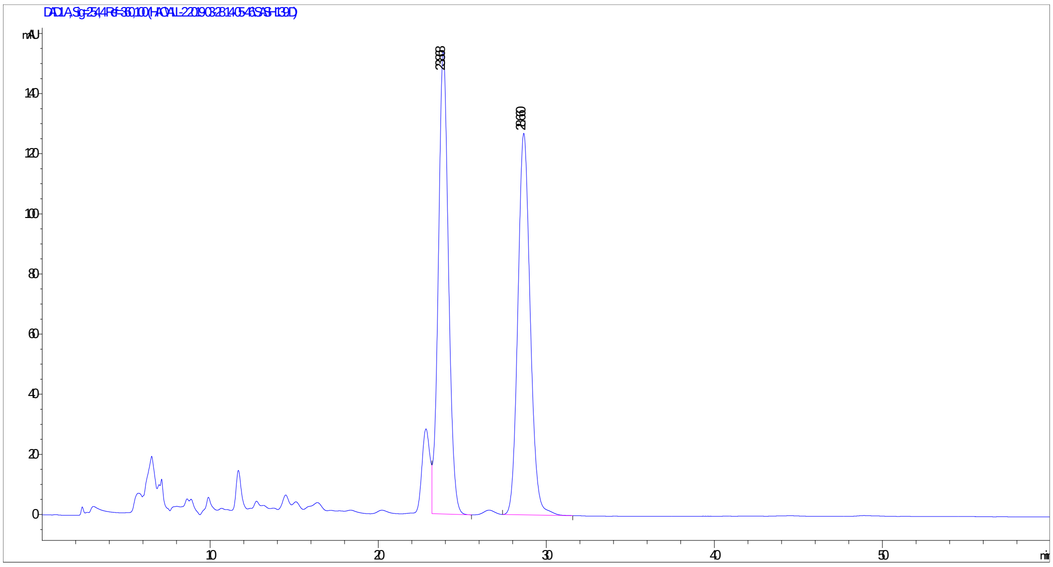

| # | Time | Area | Height | Width | Area% | Symmetry |
|---|---|---|---|---|---|---|
| 1 | 23.871 | 6346.3 | 147.2 | 0.7184 | 50.152 | 1.069 |
| 2 | 28.66 | 6307.9 | 126.3 | 0.8326 | 49.848 | 0.894 |

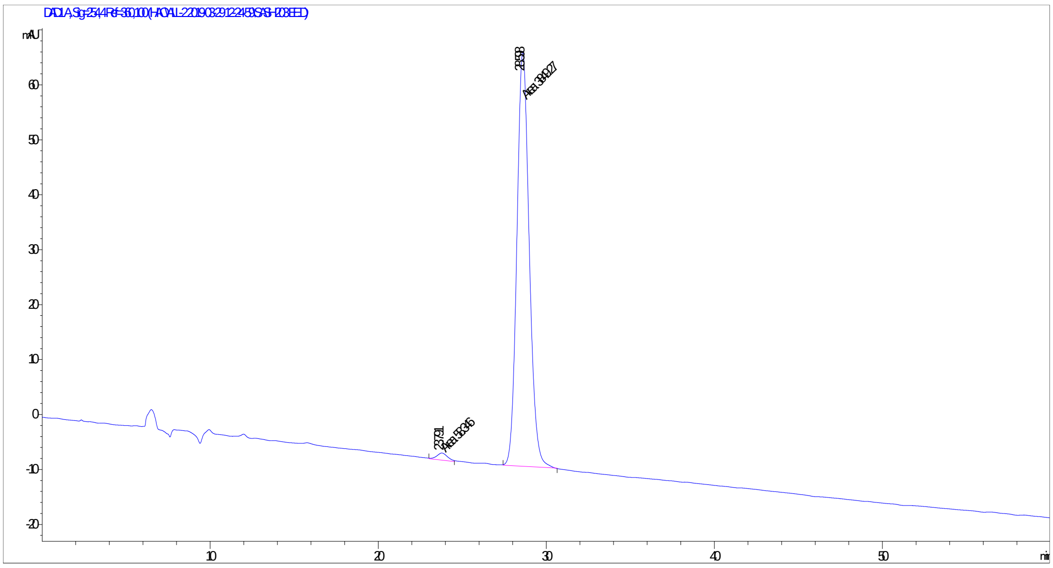

| # | Time | Area | Height | Width | Area% | Symmetry |
|---|---|---|---|---|---|---|
| 1 | 23.791 | 42.7 | 1.1 | 0.6203 | 1.099 | 0.866 |
| 2 | 28.598 | 3844.1 | 75.5 | 0.8491 | 98.901 | 0.865 |

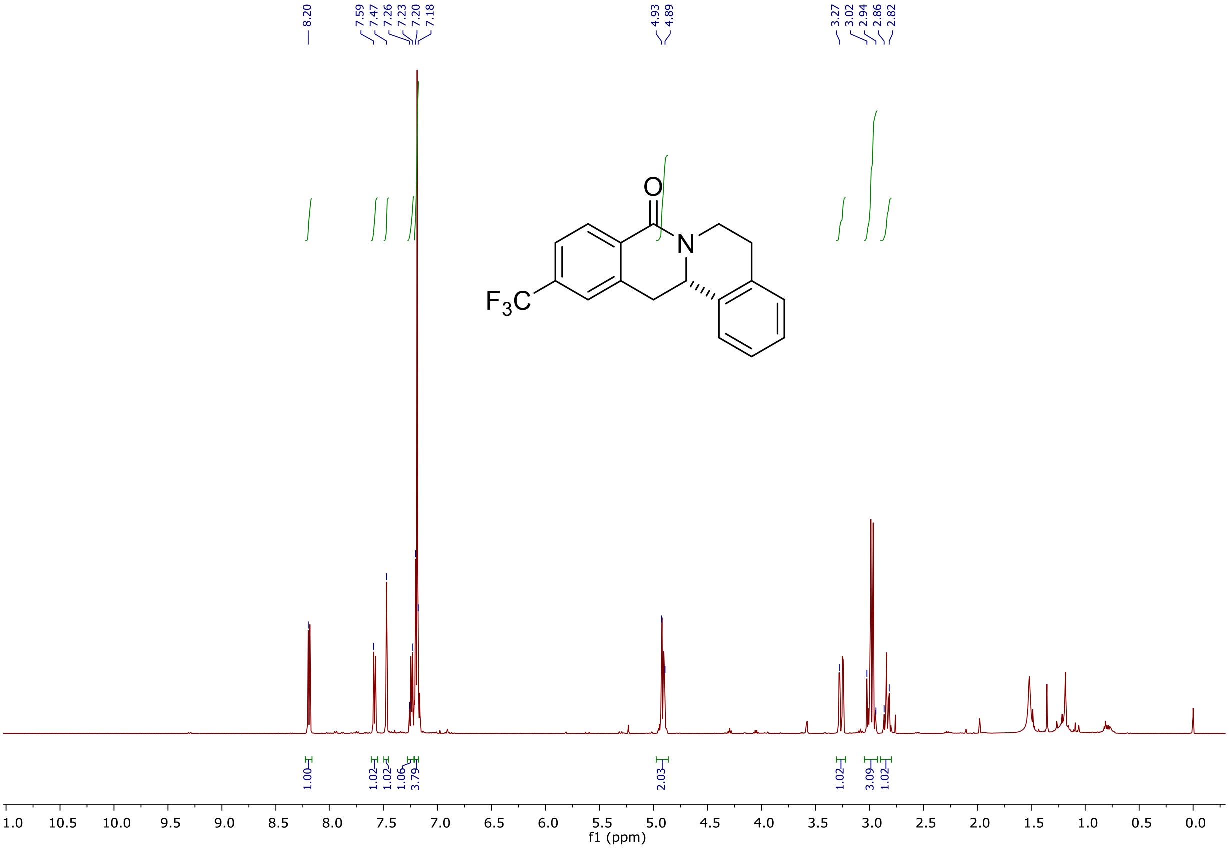
8.20
7.59
7.47
7.26
7.23
7.20
7.18
4.93
4.89
3.27
3.02
2.94
2.86
2.82
O
N
F3C
1.00
1.02
1.02
1.06
3.79
2.03
1.02
3.09
1.02
f1 (ppm)

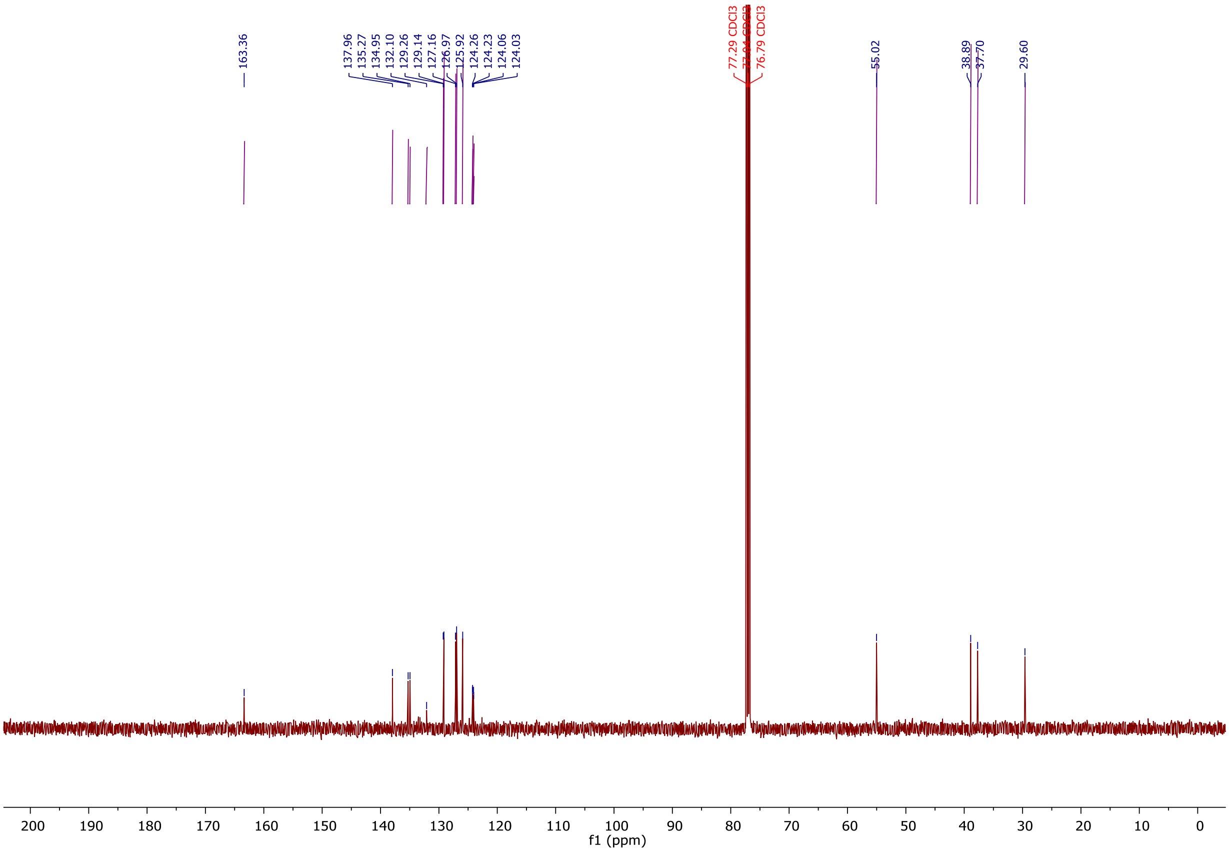
163.36
137.96
135.27
134.95
132.10
129.26
129.14
127.16
126.97
125.92
124.26
124.23
124.06
124.03
77.29 CDCl3
77.04 CDCl3
76.79 CDCl3
55.02
38.89
37.70
29.60
f1 (ppm)

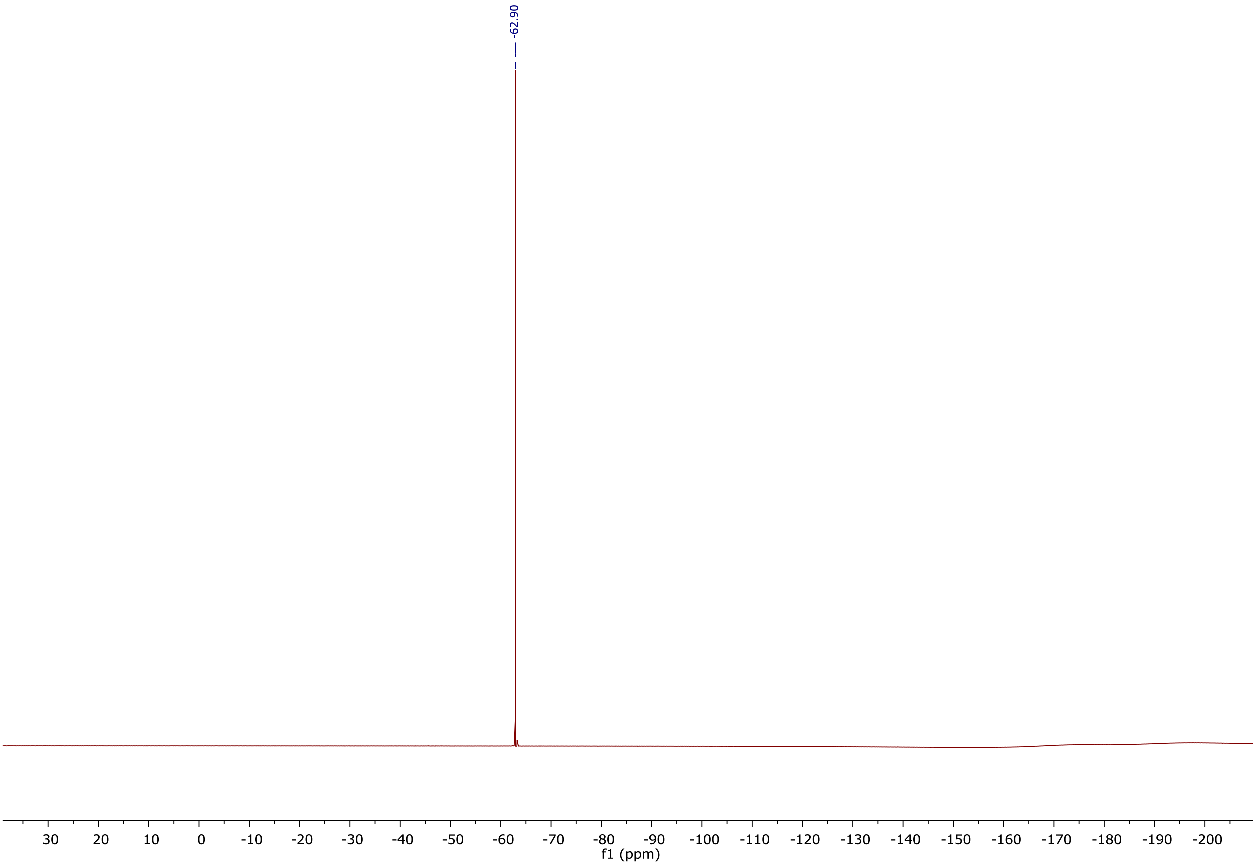
-62.90
30 20 10 0 -10 -20 -30 -40 -50 -60 -70 -80 -90 -100 -110 -120 -130 -140 -150 -160 -170 -180 -190 -200
f1 (ppm)

HPLC traces (**3i**): racemate top, enantiomer bottom:

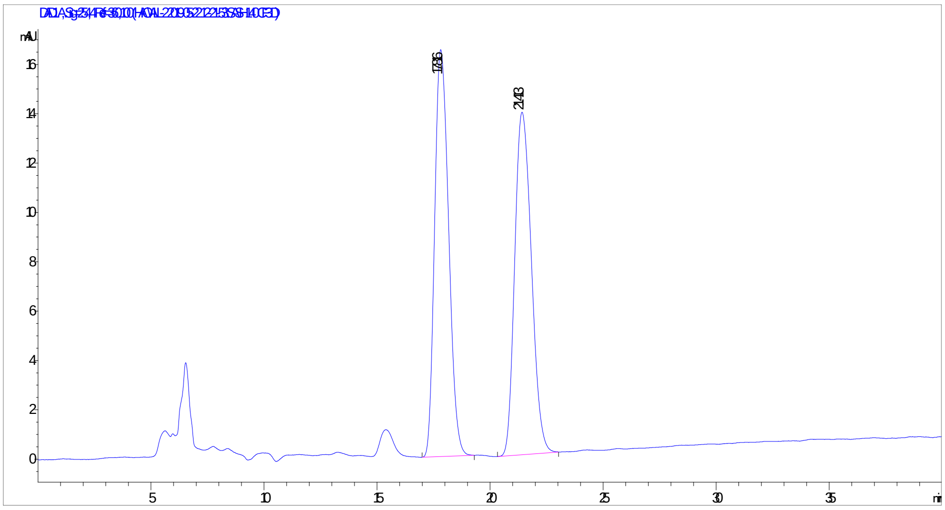

| # | Time | Area | Height | Width | Area% | Symmetry |
|---|---|---|---|---|---|---|
| 1 | 17.816 | 711 | 16.5 | 0.7176 | 49.992 | 0.749 |
| 2 | 21.413 | 711.2 | 13.9 | 0.853 | 50.008 | 0.764 |

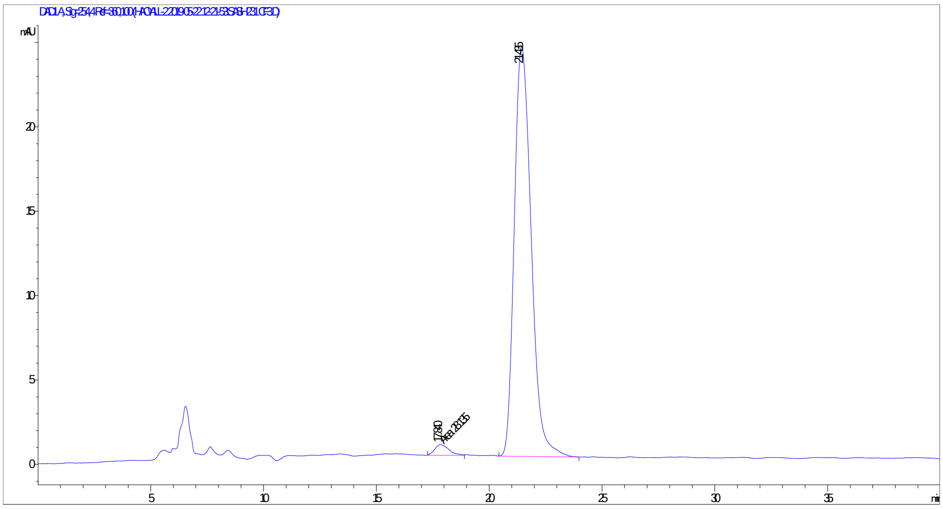

| # | Time | Area | Height | Width | Area% | Symmetry |
|---|---|---|---|---|---|---|
| 1 | 17.84 | 25.6 | 6.3E-1 | 0.6769 | 1.987 | 0.819 |
| 2 | 21.435 | 1261.8 | 24.3 | 0.8666 | 98.013 | 0.727 |

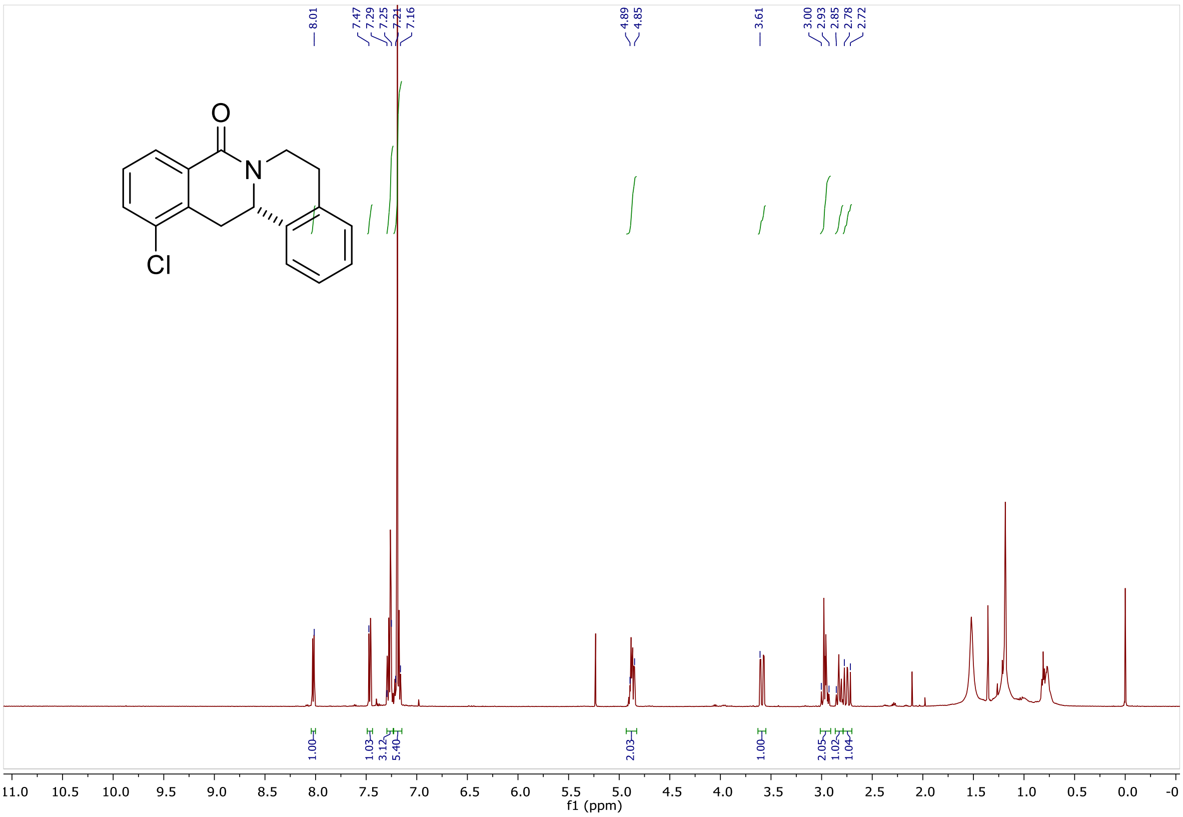

8.01
7.47
7.29
7.25
7.21
7.16
4.89
4.85
3.61
3.00
2.93
2.85
2.78
2.72
O
N
Cl
1.00
1.03
3.12
5.40
2.03
1.00
2.05
1.02
1.04
f1 (ppm)


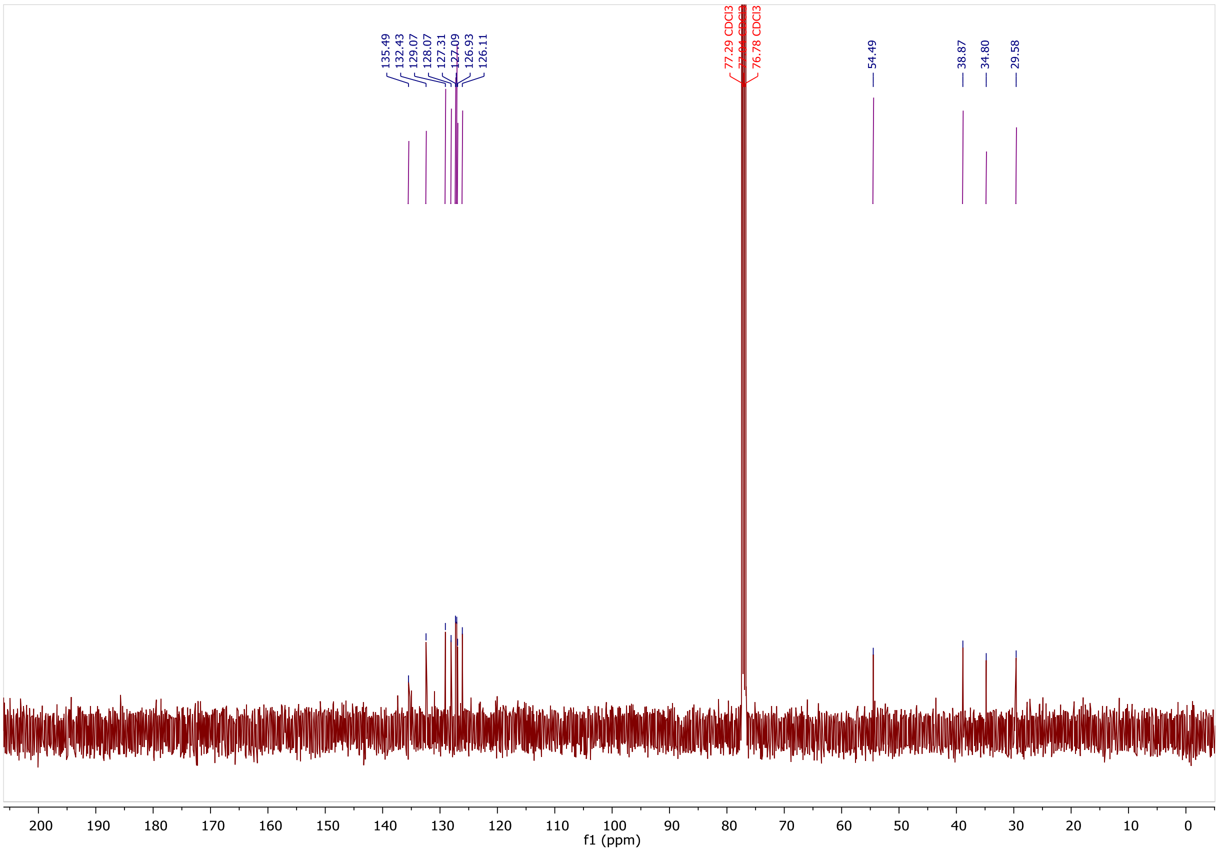

135.49
132.43
129.07
128.07
127.31
127.09
126.93
126.11
77.29 CDCl3
76.78 CDCl3
54.49
38.87
34.80
29.58
f1 (ppm)

HPLC traces (**3j**): racemate top, enantiomer bottom:

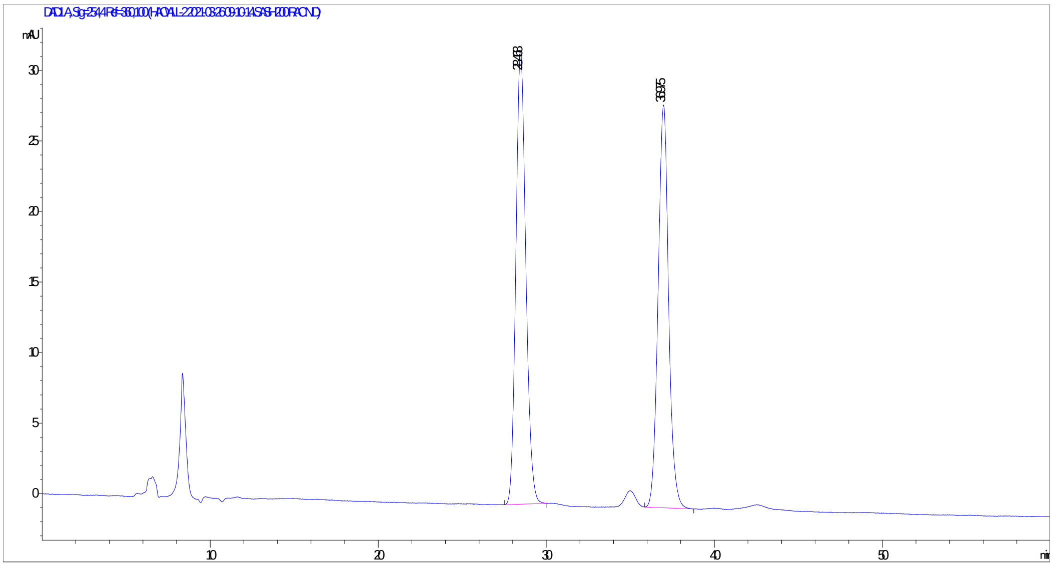


| # | Time | Area | Height | Width | Area% | Symmetry |
|---|---|---|---|---|---|---|
| 1 | 28.468 | 1320.2 | 32.1 | 0.6314 | 51.543 | 0.84 |
| 2 | 36.975 | 1241.1 | 28.5 | 0.6672 | 48.457 | 0.995 |

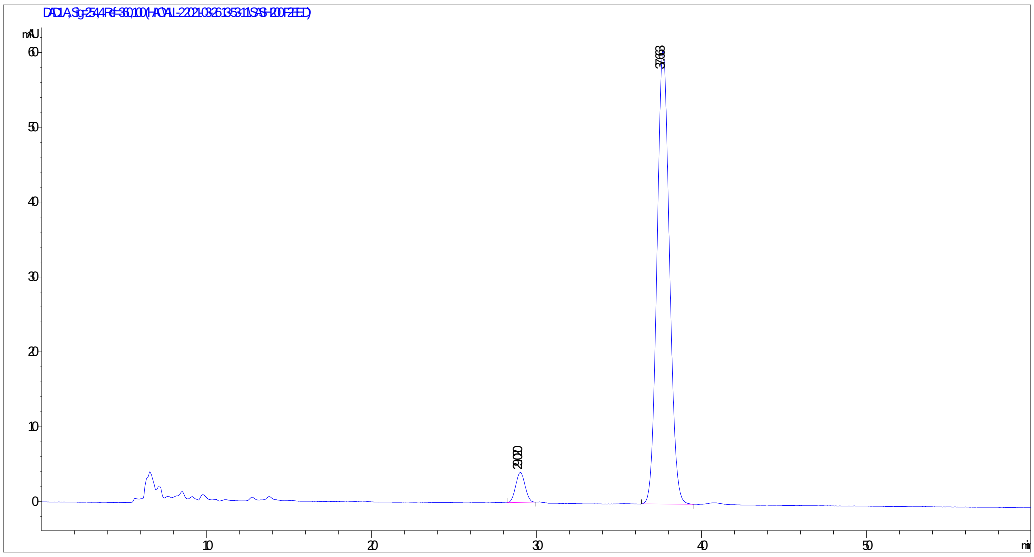


| # | Time | Area | Height | Width | Area% | Symmetry |
|---|---|---|---|---|---|---|
| 1 | 29.02 | 158.5 | 4 | 0.5593 | 4.754 | 0.983 |
| 2 | 37.663 | 3175.2 | 60.6 | 0.8135 | 95.246 | 0.9 |

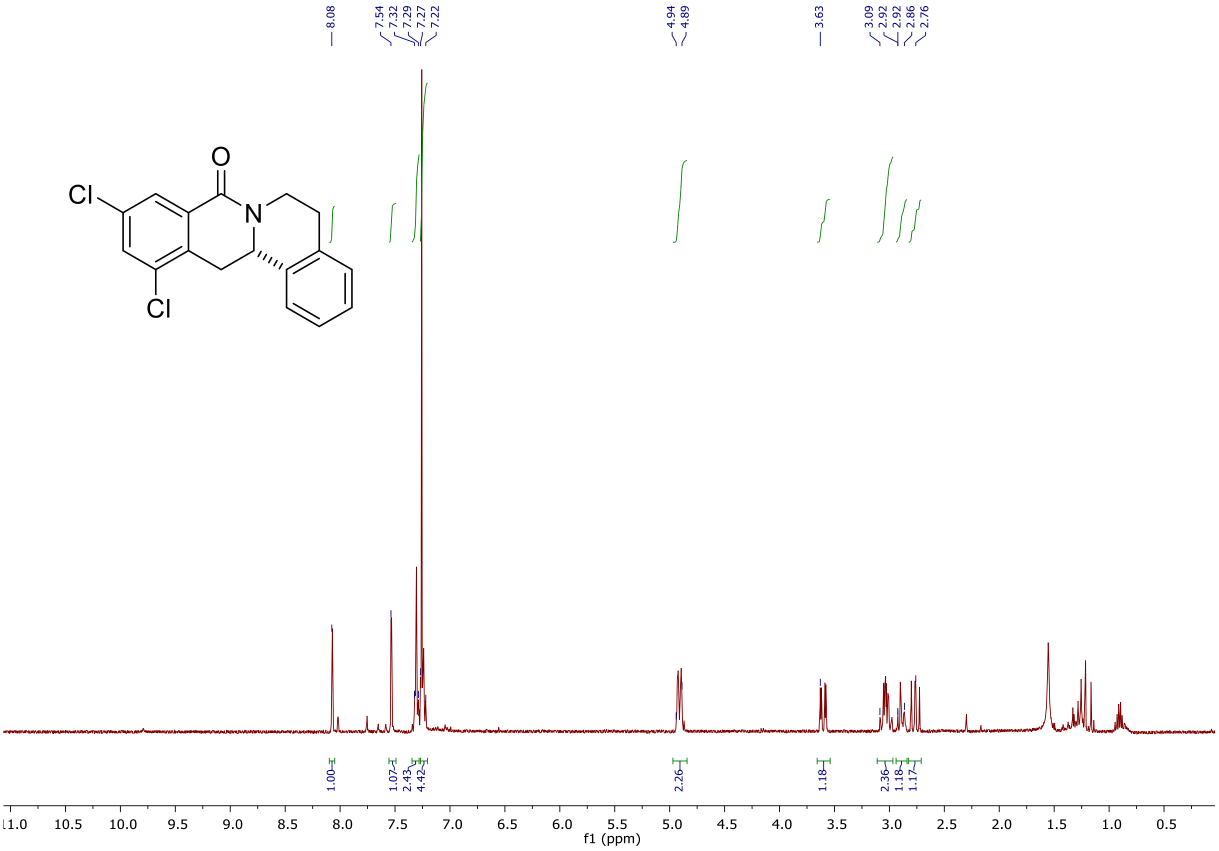

8.08
7.54
7.32
7.29
7.27
7.22
4.94
4.89
3.63
3.09
2.92
2.92
2.86
2.76
Cl
O
N
Cl
1.00
1.07
2.43
4.42
2.26
1.18
2.36
1.18
1.17
f1 (ppm)


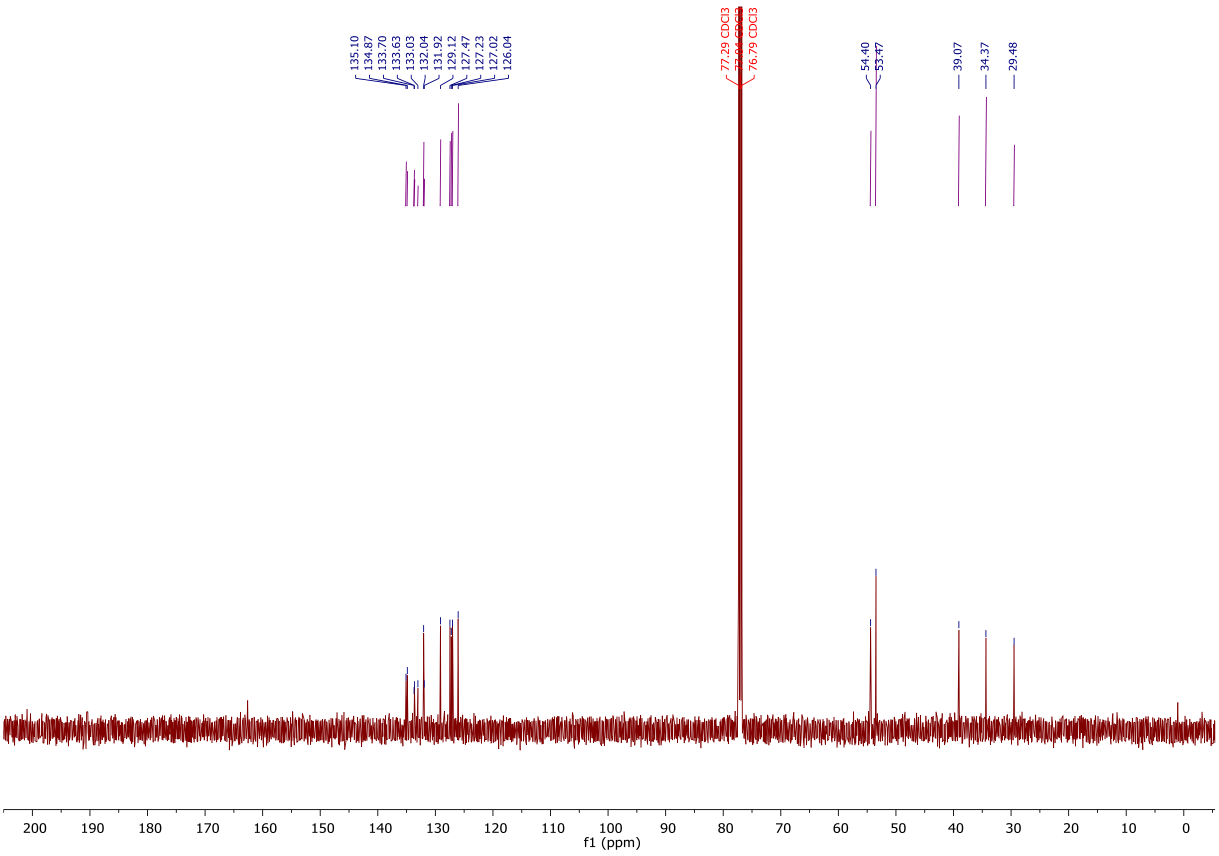

135.10
134.87
133.70
133.63
133.03
132.04
131.92
129.12
127.47
127.23
127.02
126.04
77.29 CDCl3
76.79 CDCl3
54.40
53.47
39.07
34.37
29.48
f1 (ppm)

HPLC traces (**3k**): racemate top, enantiomer bottom:

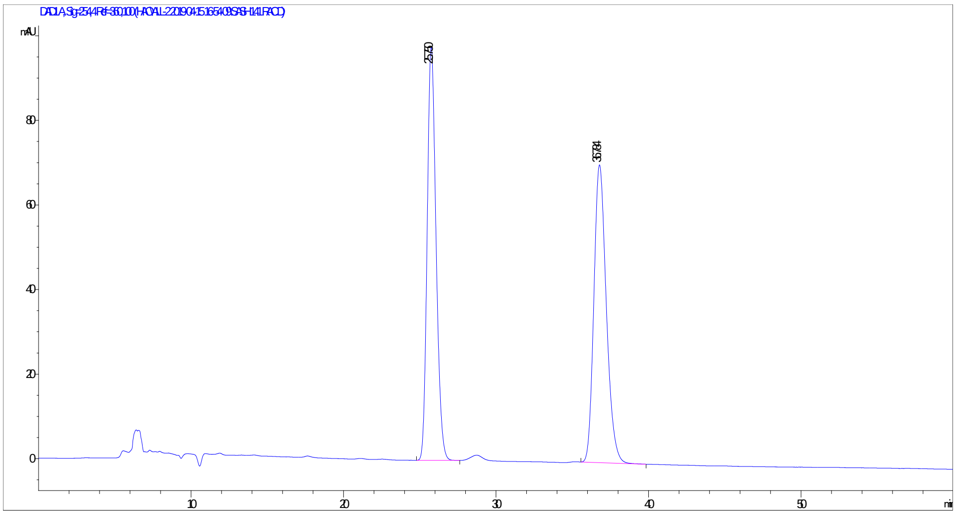

| # | Time | Area | Height | Width | Area% | Symmetry |
|---|---|---|---|---|---|---|
| 1 | 25.75 | 4002 | 98.2 | 0.6794 | 49.973 | 0.809 |
| 2 | 36.784 | 4006.4 | 70.6 | 0.9462 | 50.027 | 0.791 |

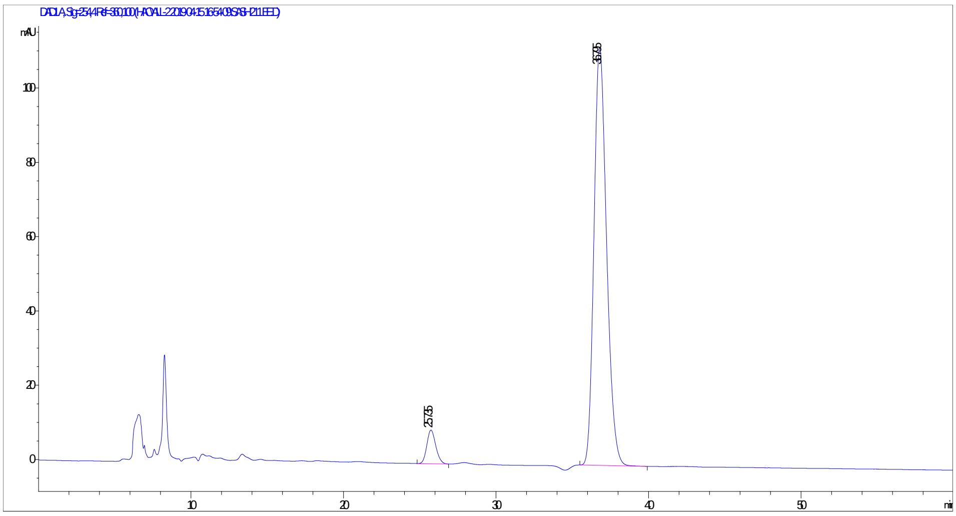

| # | Time | Area | Height | Width | Area% | Symmetry |
|---|---|---|---|---|---|---|
| 1 | 25.735 | 372.2 | 9.2 | 0.677 | 5.488 | 0.886 |
| 2 | 36.795 | 6409.5 | 112.4 | 0.9507 | 94.512 | 0.79 |

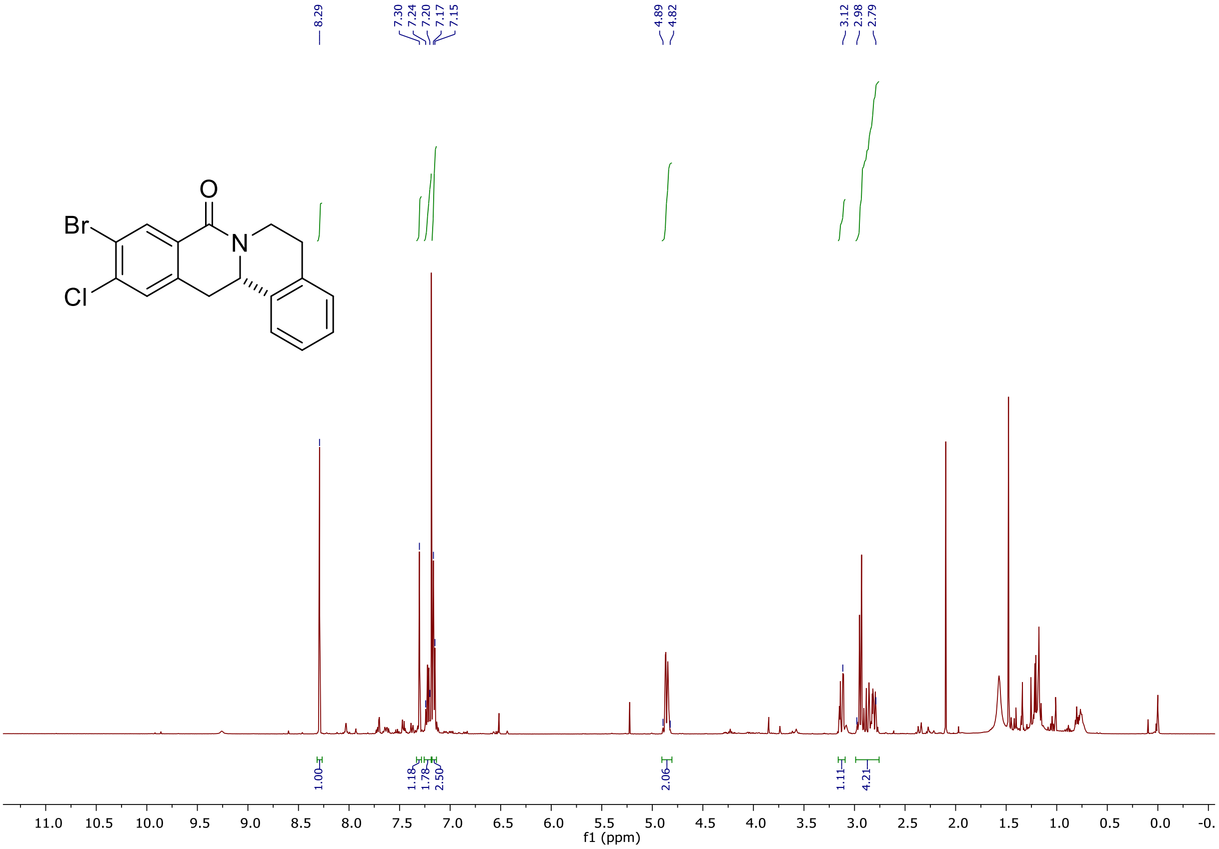
8.29
7.30
7.24
7.20
7.17
7.15
4.89
4.82
3.12
2.98
2.79
O
Br
N
Cl
1.00
1.18
1.78
2.50
2.06
1.11
4.21
f1 (ppm)

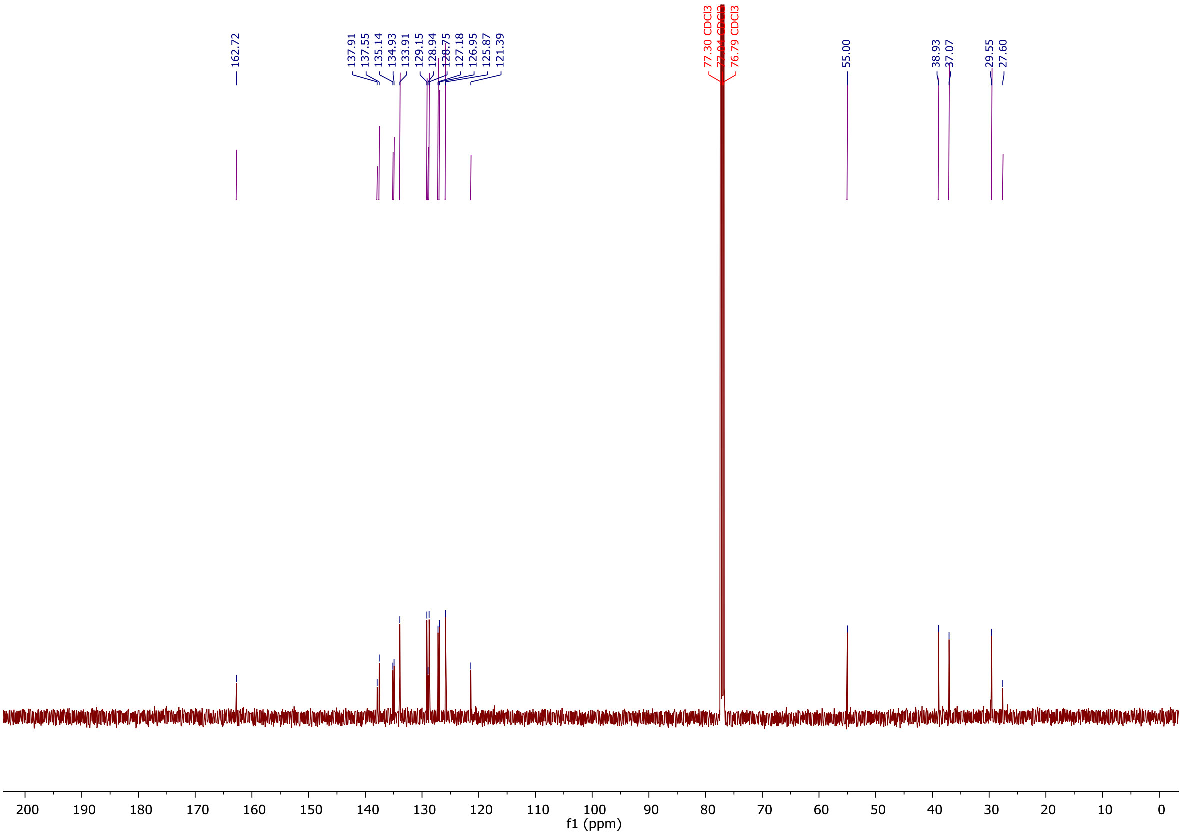
162.72
137.91
137.55
135.14
134.93
133.91
129.15
128.94
128.75
127.18
126.95
125.87
121.39
77.30 CDCl3
76.79 CDCl3
55.00
38.93
37.07
29.55
27.60
f1 (ppm)

HPLC traces (**3l**): racemate top, enantiomer bottom:

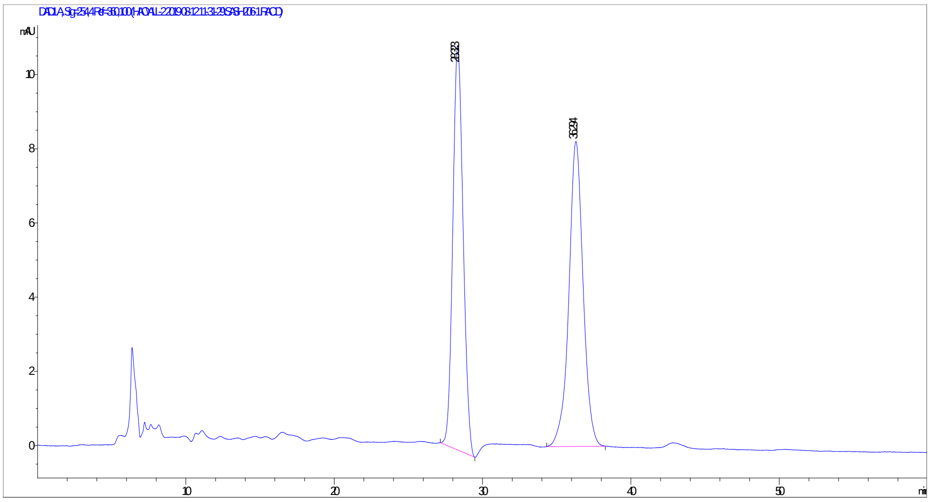

| # | Time | Area | Height | Width | Area% | Symmetry |
|---|---|---|---|---|---|---|
| 1 | 28.324 | 522.3 | 10.9 | 0.8016 | 48.898 | 0.861 |
| 2 | 36.294 | 545.9 | 8.2 | 1.1076 | 51.102 | 0.975 |

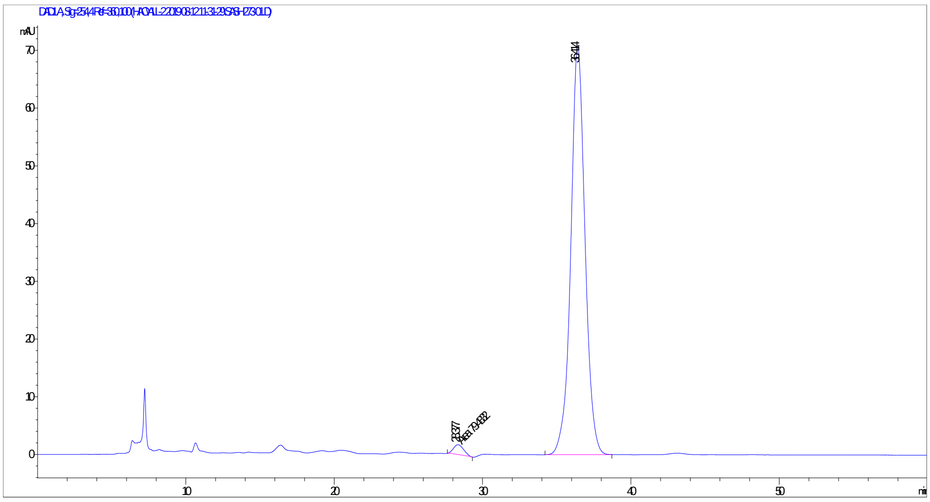

| # | Time | Area | Height | Width | Area% | Symmetry |
|---|---|---|---|---|---|---|
| 1 | 28.39 | 96.1 | 1.9 | 0.8491 | 2.000 | 0.576 |
| 2 | 36.414 | 4709.7 | 70.8 | 1.1093 | 98.000 | 0.915 |

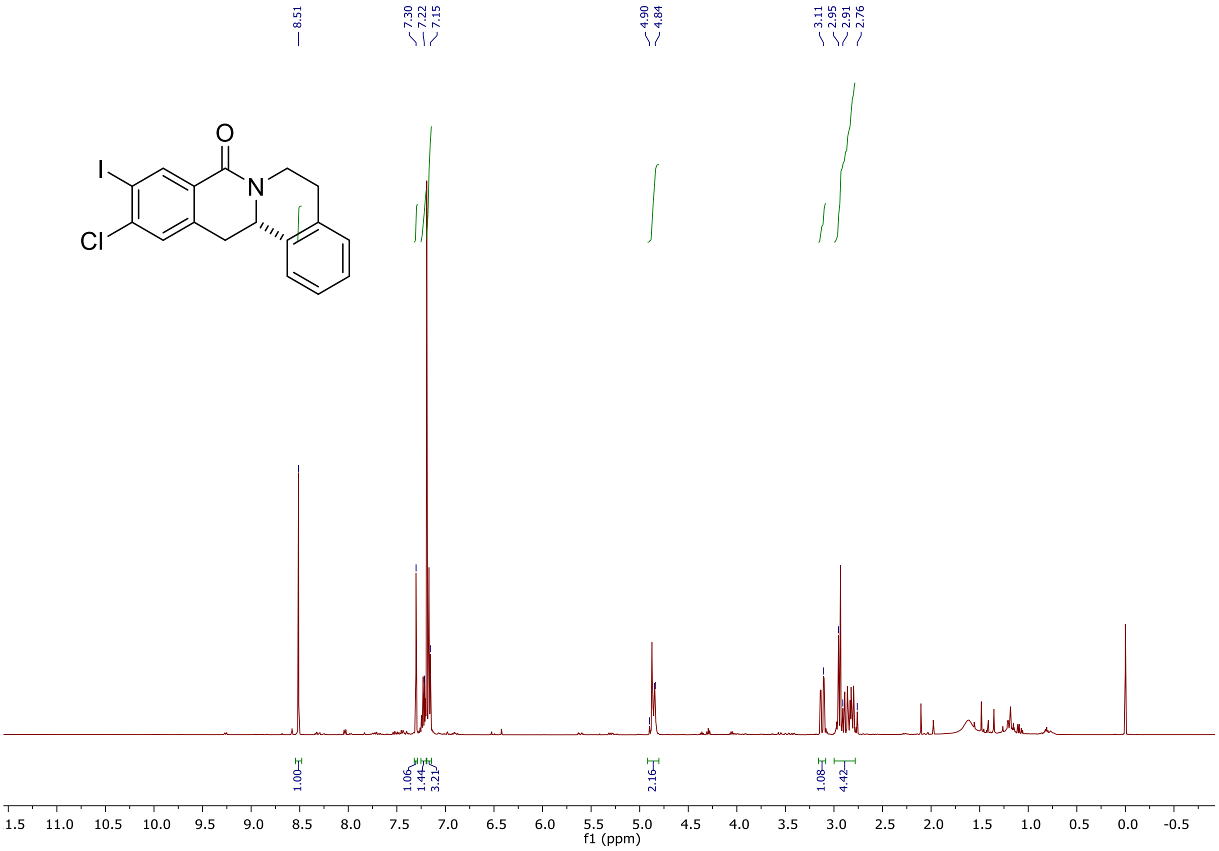

O
I
N
Cl
8.51
7.30
7.22
7.15
4.90
4.84
3.11
2.95
2.91
2.76
1.00
1.06
1.44
3.21
2.16
1.08
4.42
11.5 11.0 10.5 10.0 9.5 9.0 8.5 8.0 7.5 7.0 6.5 6.0 5.5 5.0 4.5 4.0 3.5 3.0 2.5 2.0 1.5 1.0 0.5 0.0 -0.5
f1 (ppm)


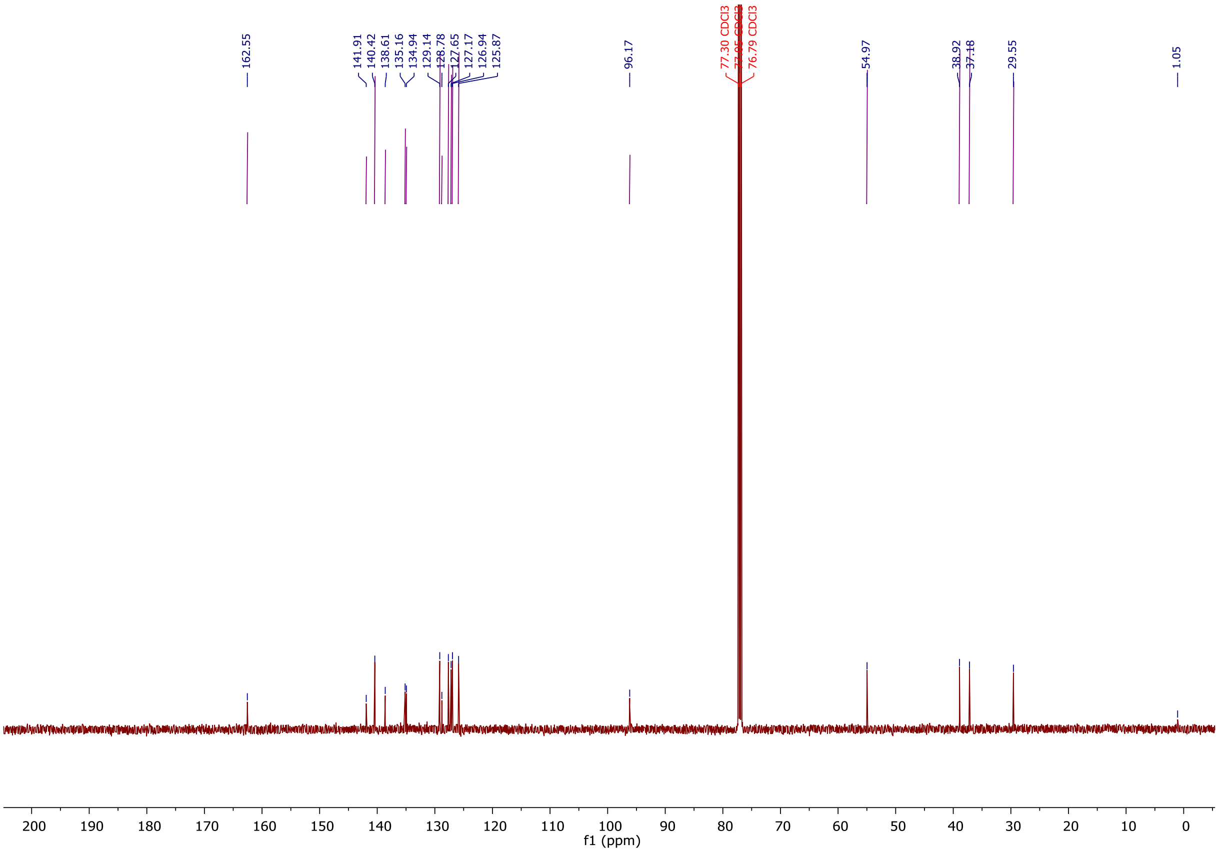

162.55
141.91
140.42
138.61
135.16
134.94
129.14
128.78
127.65
127.17
126.94
125.87
96.17
77.30 CDCl3
76.79 CDCl3
54.97
38.92
37.18
29.55
1.05
200 190 180 170 160 150 140 130 120 110 100 90 80 70 60 50 40 30 20 10 0
f1 (ppm)

HPLC traces (**3m**): racemate top, enantiomer bottom:

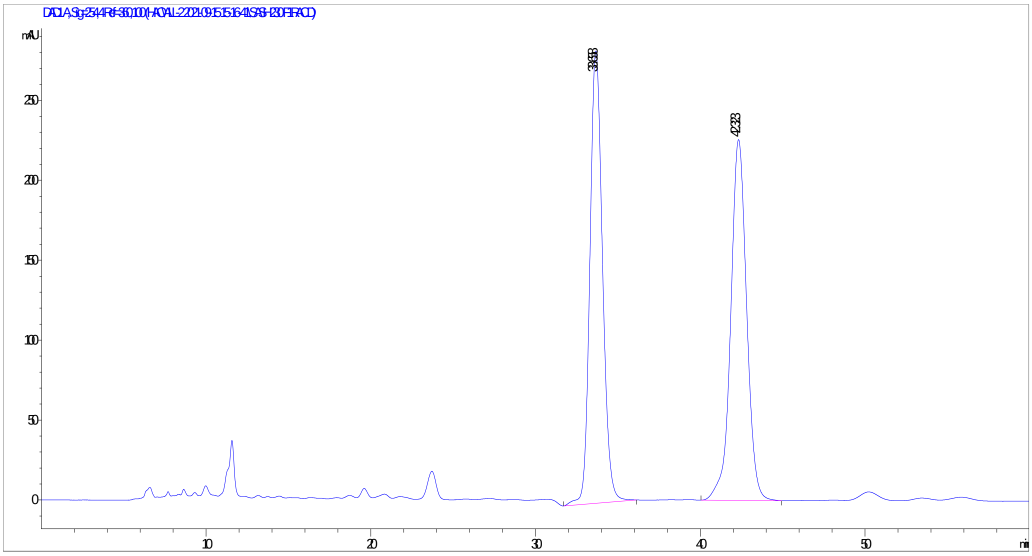

| # | Time | Area | Height | Width | Area% | Symmetry |
|---|---|---|---|---|---|---|
| 1 | 33.658 | 15064.5 | 282.9 | 0.8292 | 49.818 | 0.85 |
| 2 | 42.323 | 15174.8 | 225.8 | 1.0262 | 50.182 | 0.958 |

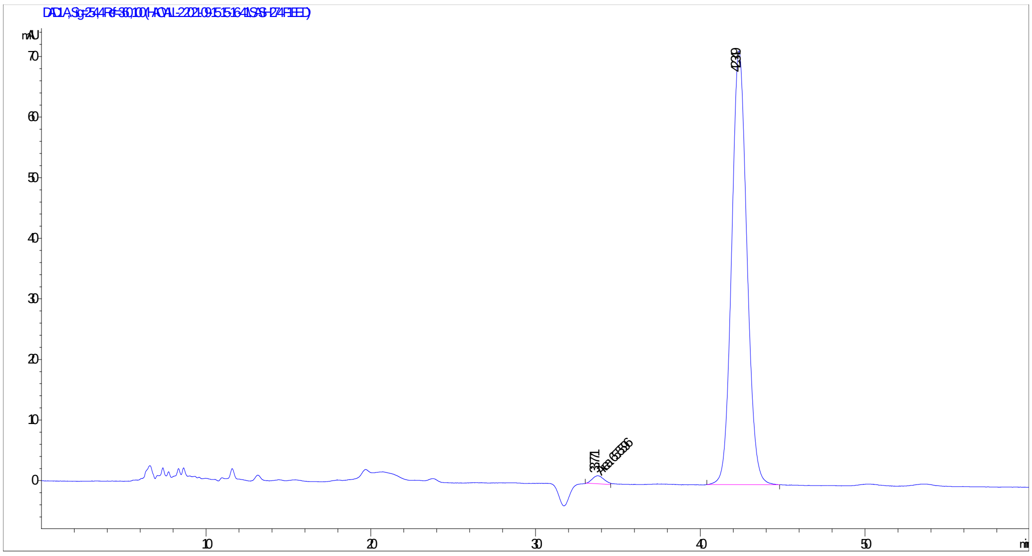

| # | Time | Area | Height | Width | Area% | Symmetry |
|---|---|---|---|---|---|---|
| 1 | 33.771 | 65.6 | 1.3 | 0.8213 | 1.398 | 0.774 |
| 2 | 42.349 | 4622.6 | 71.6 | 0.9911 | 98.602 | 0.889 |

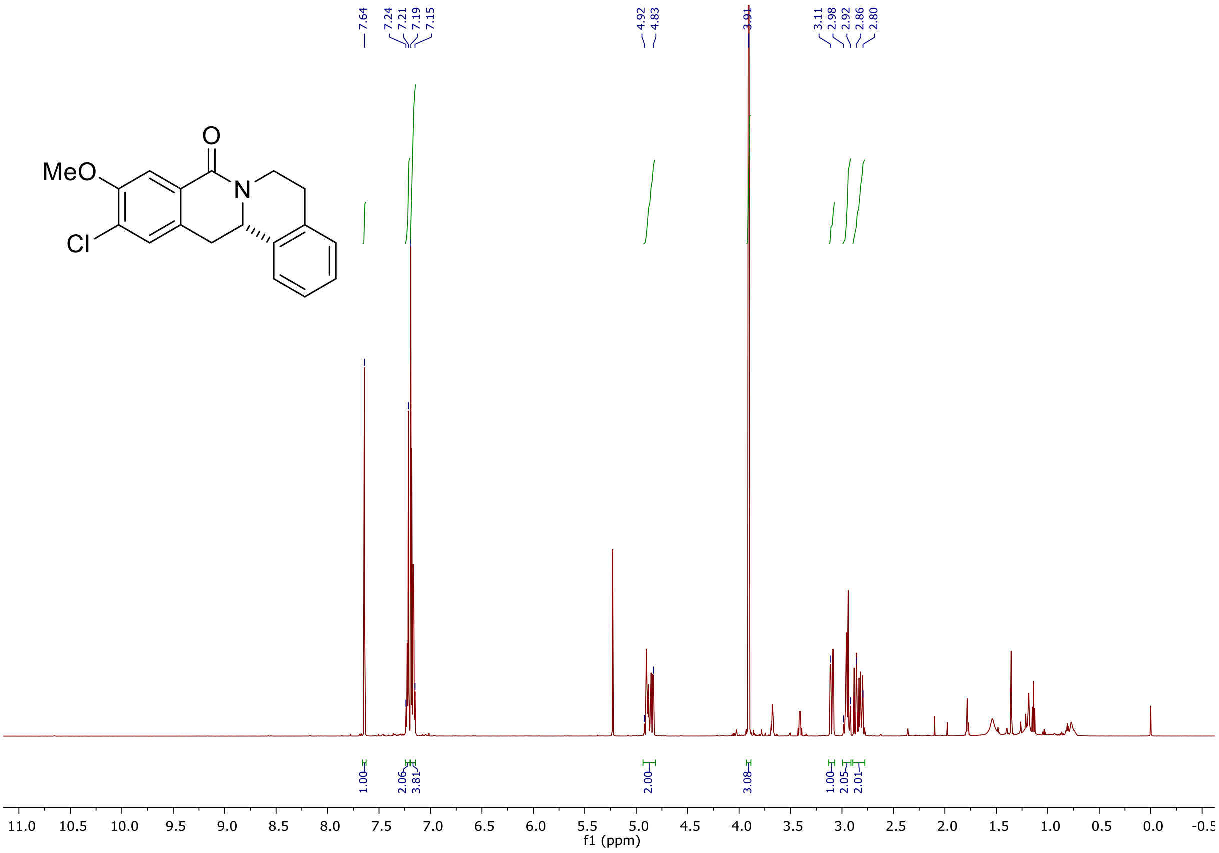

MeO
Cl
O
N
7.64
7.24
7.21
7.19
7.15
4.92
4.83
3.91
3.11
2.98
2.92
2.86
2.80
1.00
2.06
3.81
2.00
3.08
1.00
2.05
2.01
11.0 10.5 10.0 9.5 9.0 8.5 8.0 7.5 7.0 6.5 6.0 5.5 5.0 4.5 4.0 3.5 3.0 2.5 2.0 1.5 1.0 0.5 0.0
f1 (ppm)


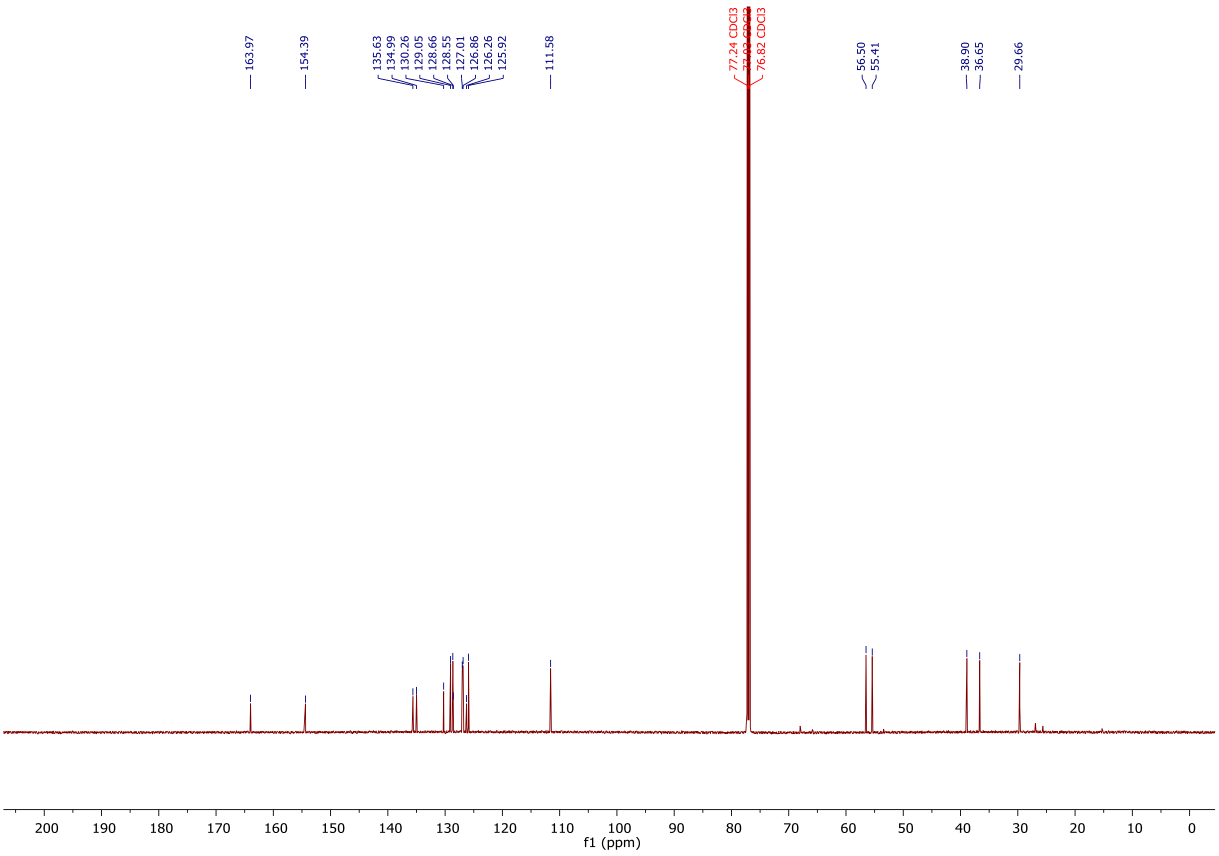

163.97
154.39
135.63
134.99
130.26
129.05
128.66
128.55
127.01
126.86
126.26
125.92
111.58
77.24 CDCl3
76.82 CDCl3
56.50
55.41
38.90
36.65
29.66
200 190 180 170 160 150 140 130 120 110 100 90 80 70 60 50 40 30 20 10 0
f1 (ppm)

HPLC traces (**3n**): racemate top, enantiomer bottom:

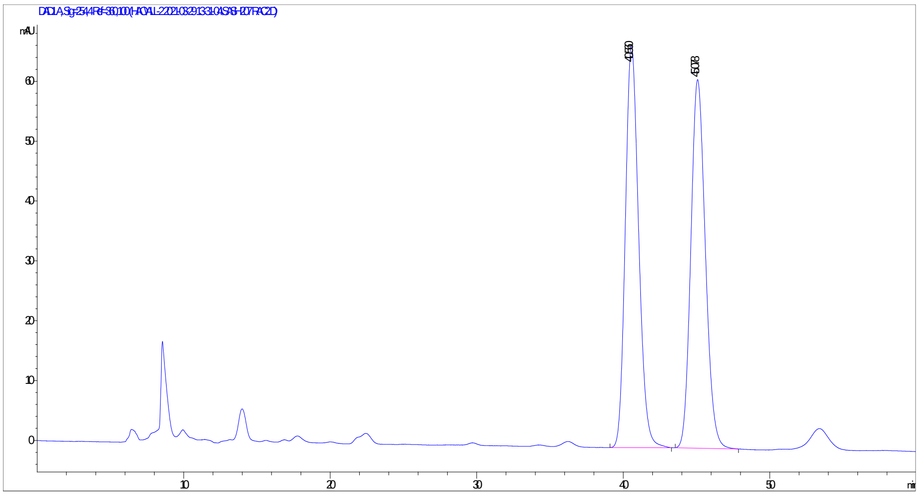


| # | Time | Area | Height | Width | Area% | Symmetry |
|---|---|---|---|---|---|---|
| 1 | 40.56 | 4252 | 67.3 | 0.9675 | 50.053 | 0.801 |
| 2 | 45.078 | 4243 | 61.6 | 1.0556 | 49.947 | 0.832 |

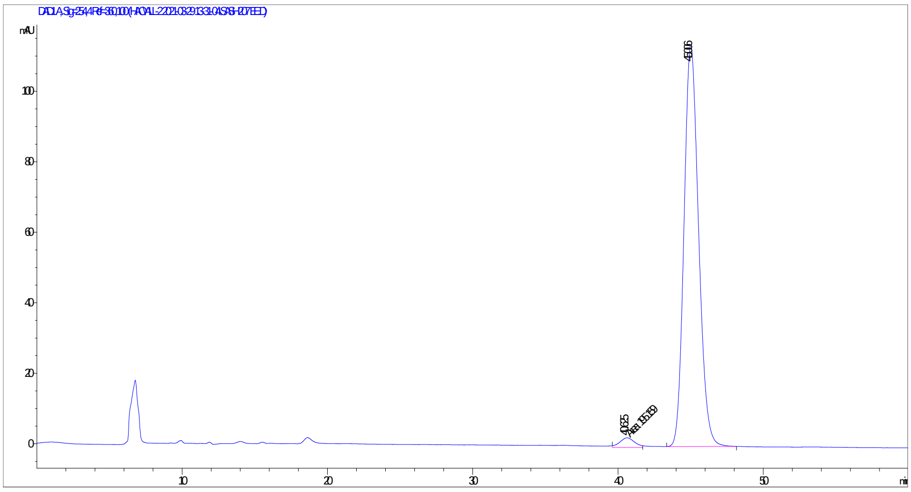


| # | Time | Area | Height | Width | Area% | Symmetry |
|---|---|---|---|---|---|---|
| 1 | 40.625 | 170.3 | 2.5 | 1.118 | 2.102 | 0.948 |
| 2 | 45.006 | 7931.8 | 113.9 | 1.0882 | 97.898 | 0.81 |

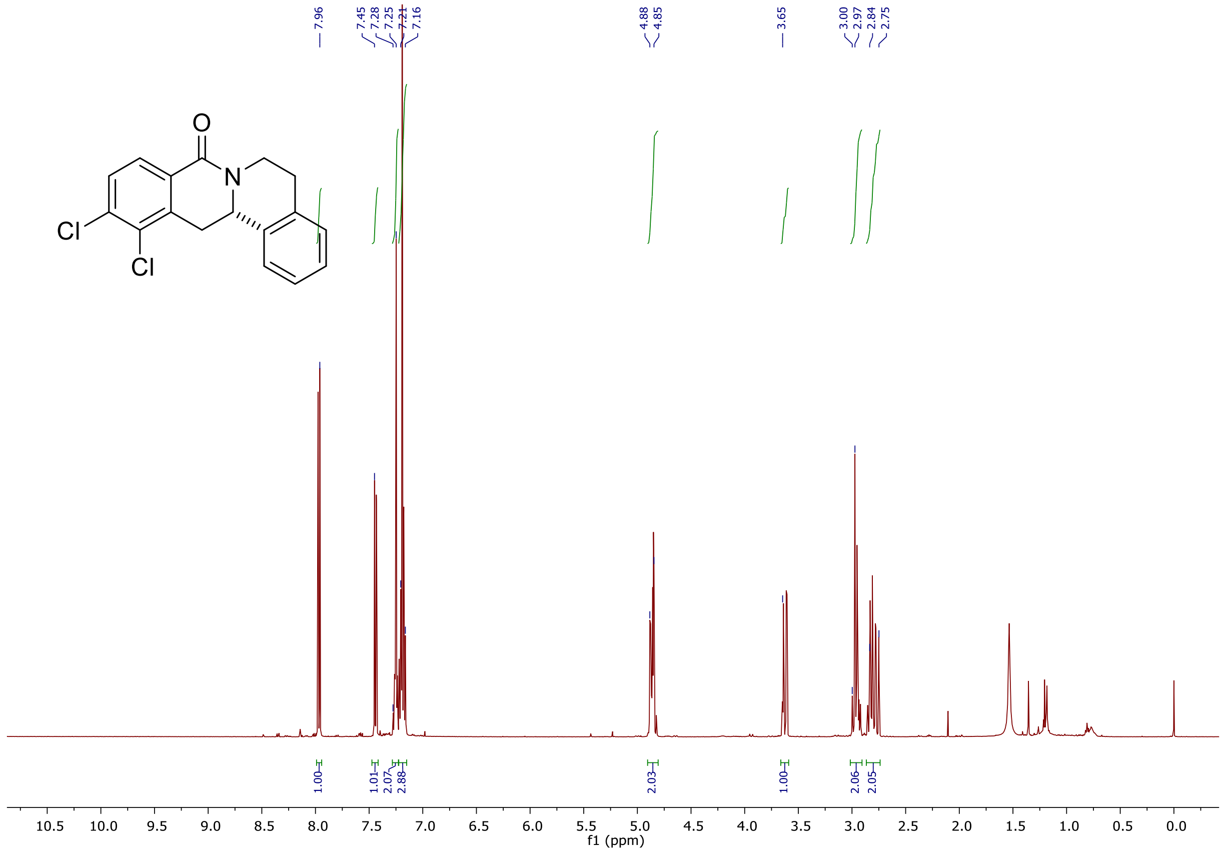
7.96
7.45
7.28
7.25
7.21
7.16
4.88
4.85
3.65
3.00
2.97
2.84
2.75
O
N
Cl
Cl
1.00
1.01
2.07
2.88
2.03
1.00
2.06
2.05
10.5 10.0 9.5 9.0 8.5 8.0 7.5 7.0 6.5 6.0 5.5 5.0 4.5 4.0 3.5 3.0 2.5 2.0 1.5 1.0 0.5 0.0
f1 (ppm)

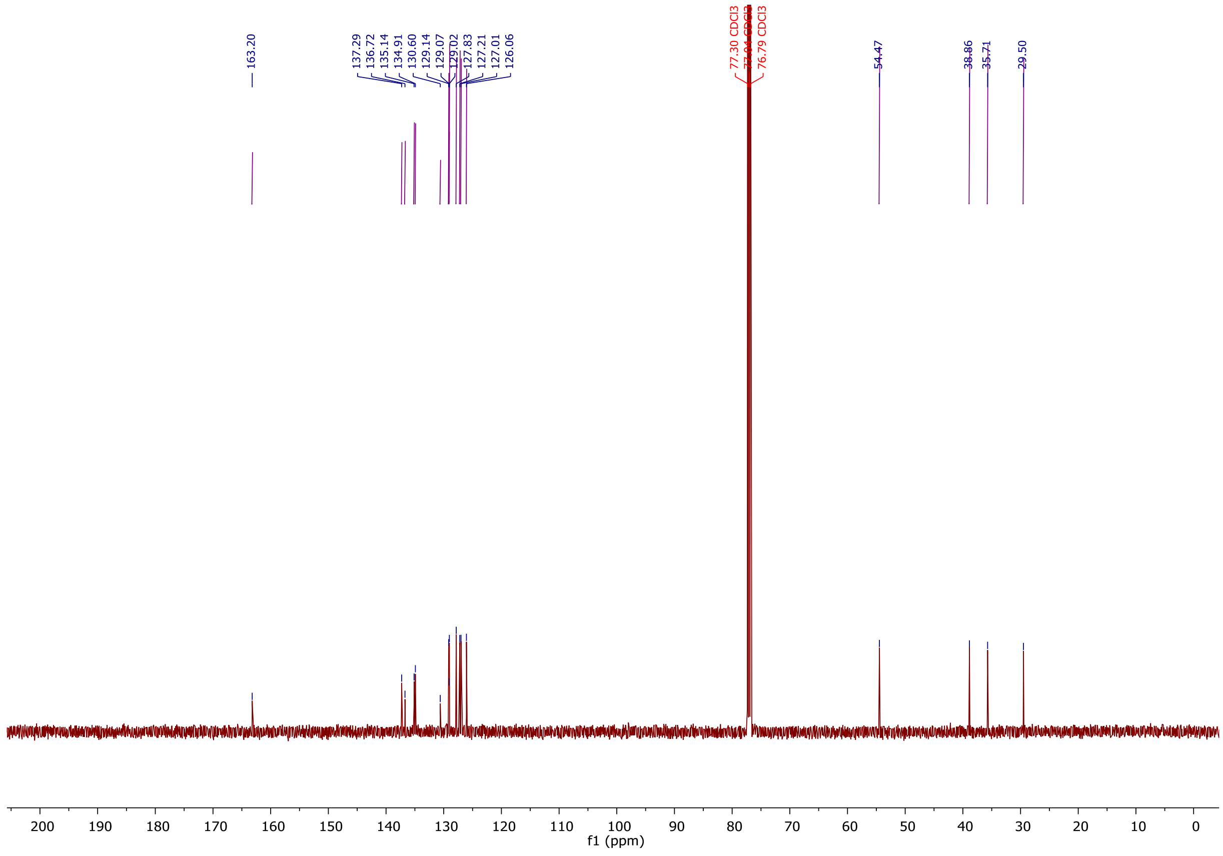
163.20
137.29
136.72
135.14
134.91
130.60
129.14
129.07
129.02
127.83
127.21
127.01
126.06
77.30 CDCl3
76.79 CDCl3
54.47
38.86
35.71
29.50
200 190 180 170 160 150 140 130 120 110 100 90 80 70 60 50 40 30 20 10 0
f1 (ppm)

HPLC traces (**3p**): racemate top, enantiomer bottom:

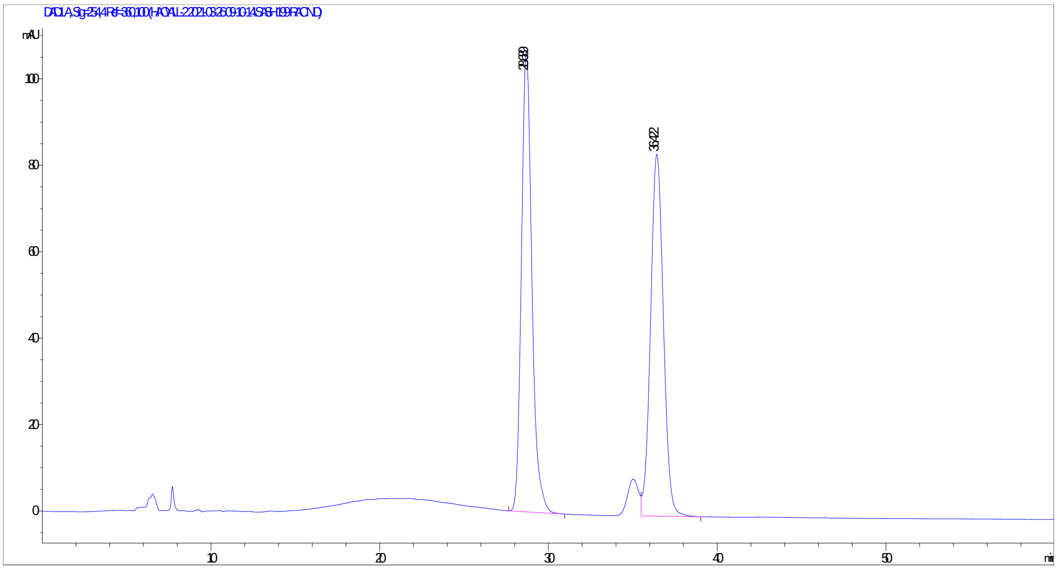

| # | Time | Area | Height | Width | Area% | Symmetry |
|---|---|---|---|---|---|---|
| 1 | 28.689 | 4591.7 | 106.6 | 0.6606 | 50.899 | 0.819 |
| 2 | 36.422 | 4429.5 | 83.8 | 0.825 | 49.101 | 0.909 |

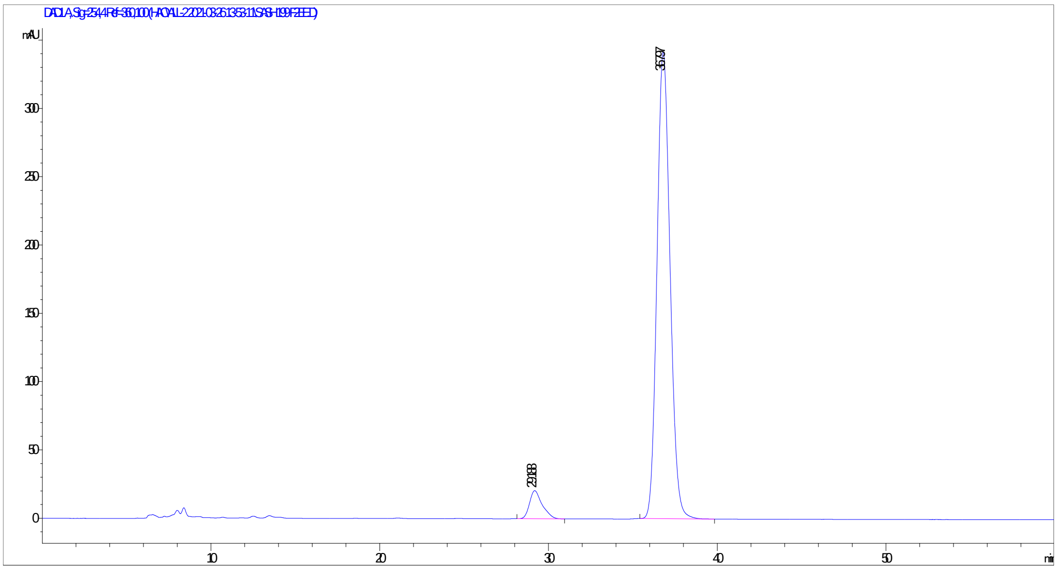

| # | Time | Area | Height | Width | Area% | Symmetry |
|---|---|---|---|---|---|---|
| 1 | 29.188 | 1072.8 | 20.6 | 0.7576 | 5.521 | 0.664 |
| 2 | 36.797 | 18358 | 342 | 0.8363 | 94.479 | 0.845 |

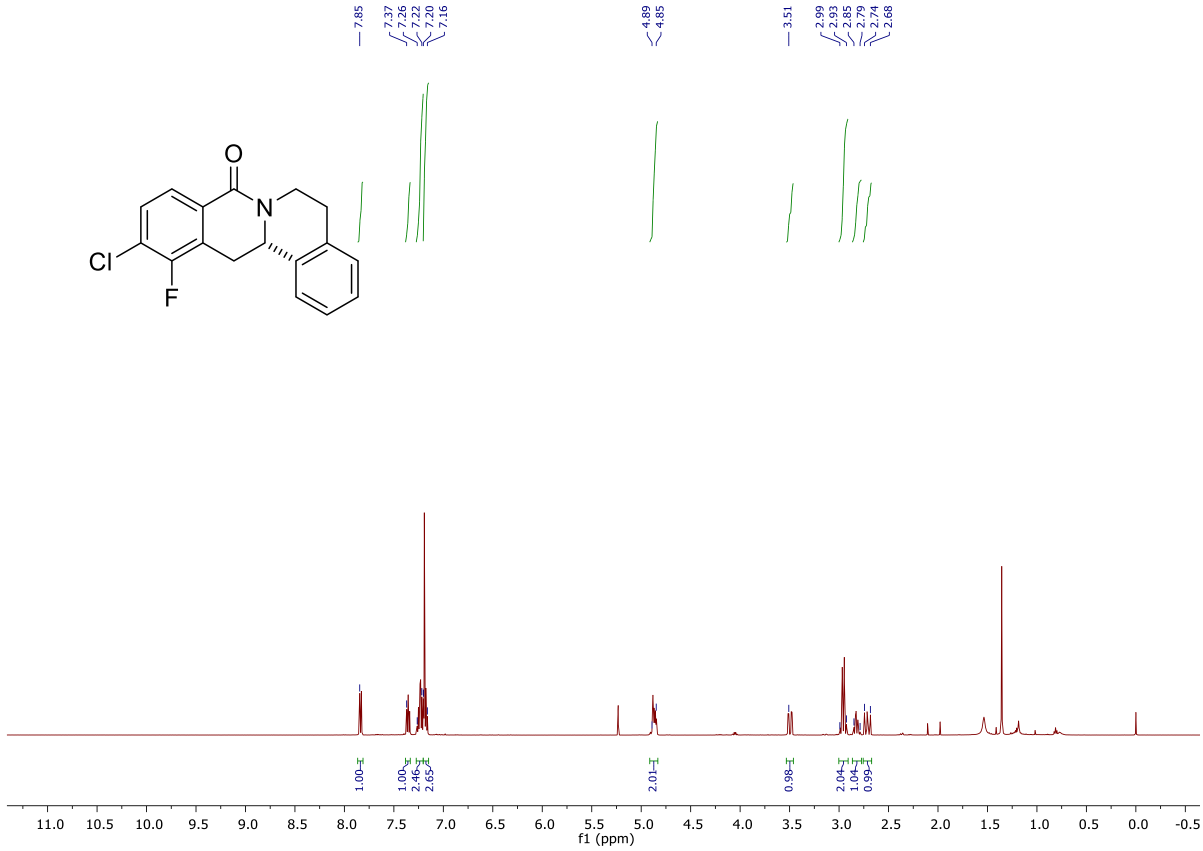
7.85
7.37
7.26
7.22
7.20
7.16
4.89
4.85
3.51
2.99
2.93
2.85
2.79
2.74
2.68
O
N
Cl
F
1.00
1.00
2.46
2.65
2.01
0.98
2.04
1.04
0.99
11.0 10.5 10.0 9.5 9.0 8.5 8.0 7.5 7.0 6.5 6.0 5.5 5.0 4.5 4.0 3.5 3.0 2.5 2.0 1.5 1.0 0.5 0.0 -0.5
f1 (ppm)

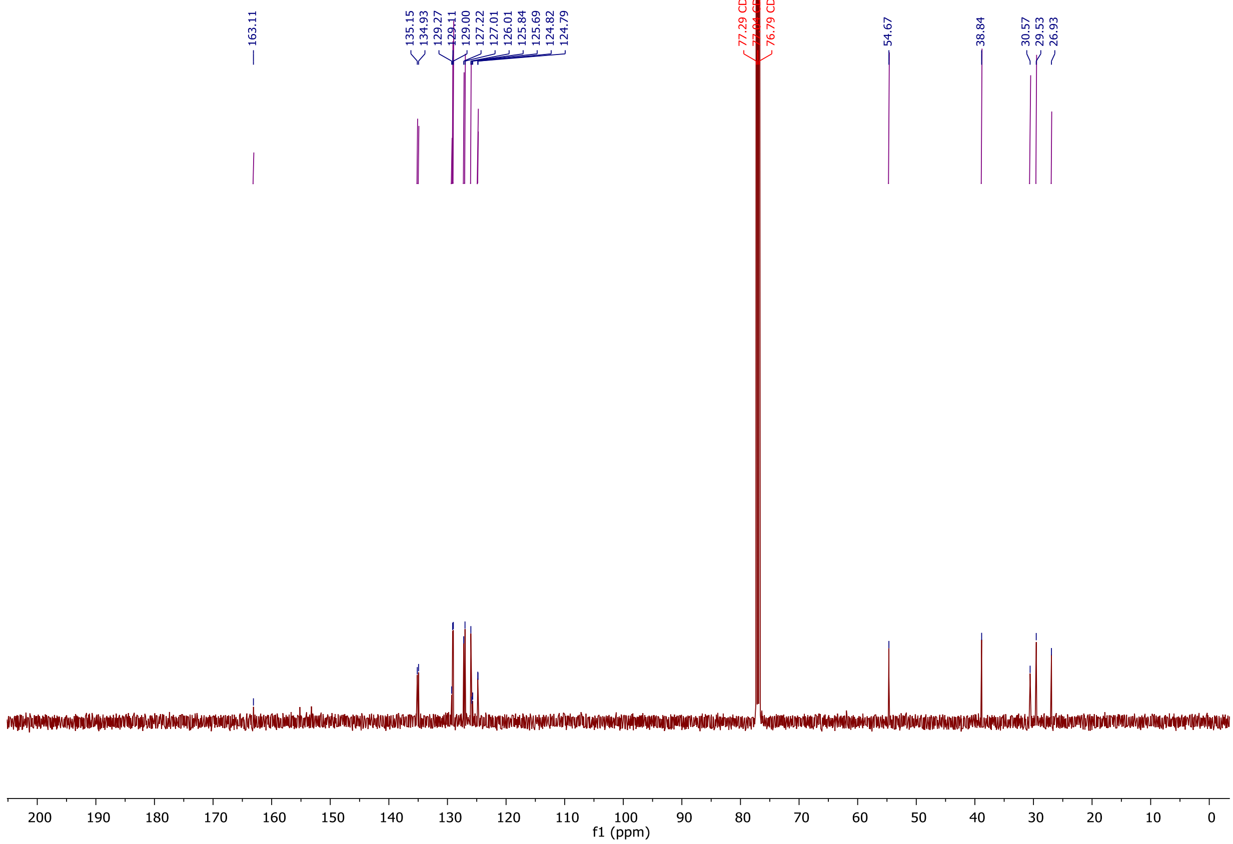
163.11
135.15
134.93
129.27
129.11
129.00
127.22
127.01
126.01
125.84
125.69
124.82
124.79
77.29 CDCl3
76.79 CDCl3
54.67
38.84
30.57
29.53
26.93
200 190 180 170 160 150 140 130 120 110 100 90 80 70 60 50 40 30 20 10 0
f1 (ppm)

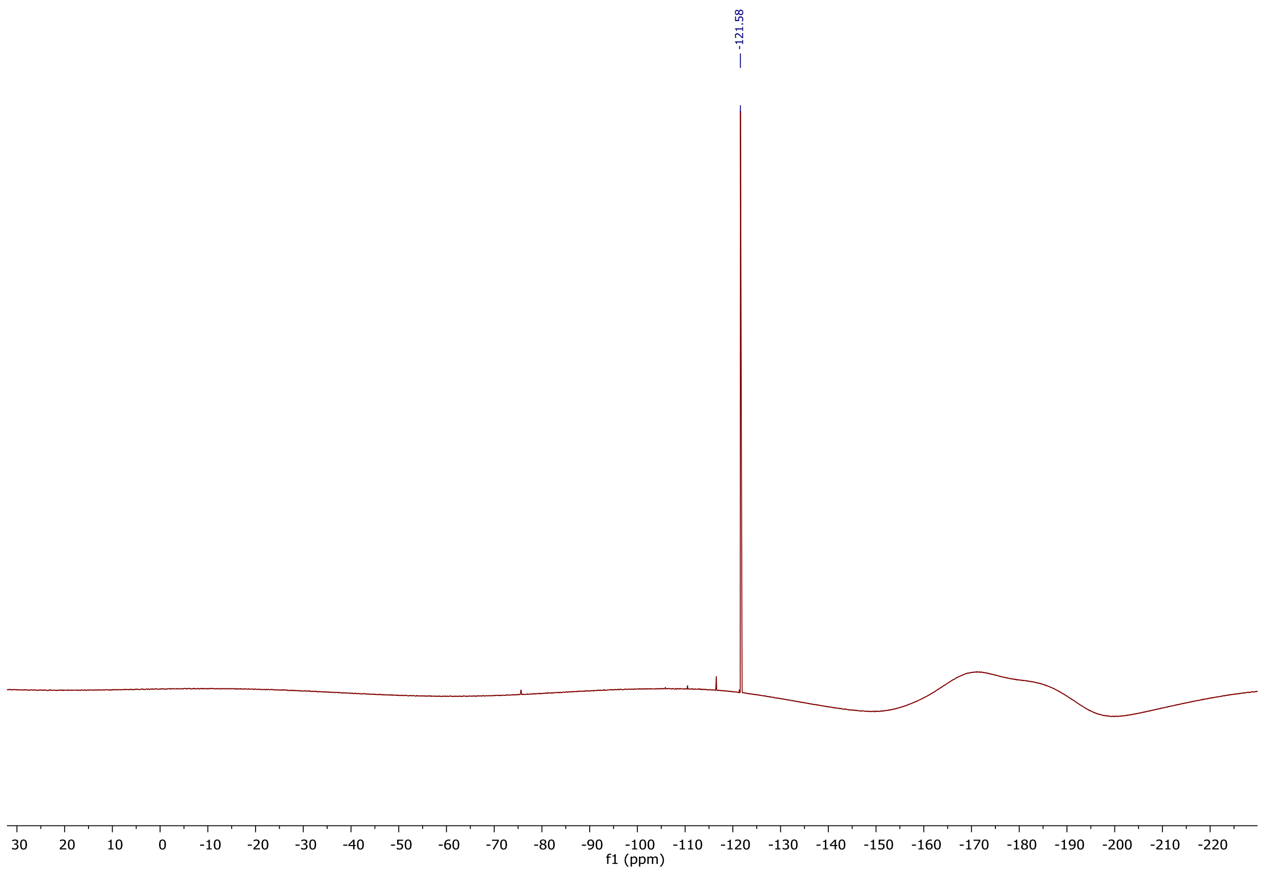

-121.58
30 20 10 0 -10 -20 -30 -40 -50 -60 -70 -80 -90 -100 -110 -120 -130 -140 -150 -160 -170 -180 -190 -200 -210 -220
f1 (ppm)

HPLC traces (**3o**): racemate top, enantiomer bottom:

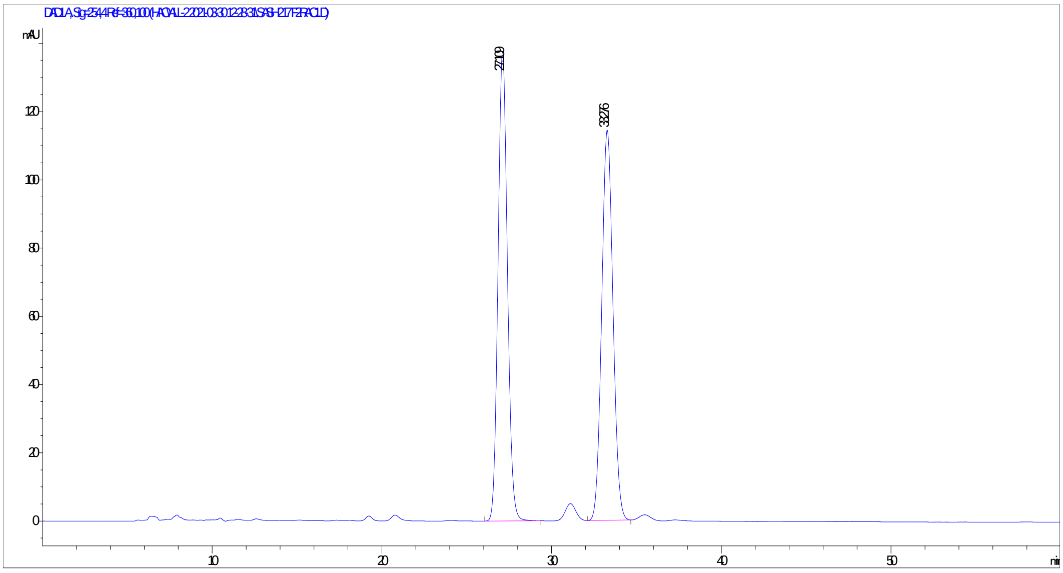


| # | Time | Area | Height | Width | Area% | Symmetry |
|---|---|---|---|---|---|---|
| 1 | 27.109 | 5297.1 | 137.6 | 0.5951 | 50.088 | 0.875 |
| 2 | 33.276 | 5278.5 | 114.4 | 0.7165 | 49.912 | 0.914 |

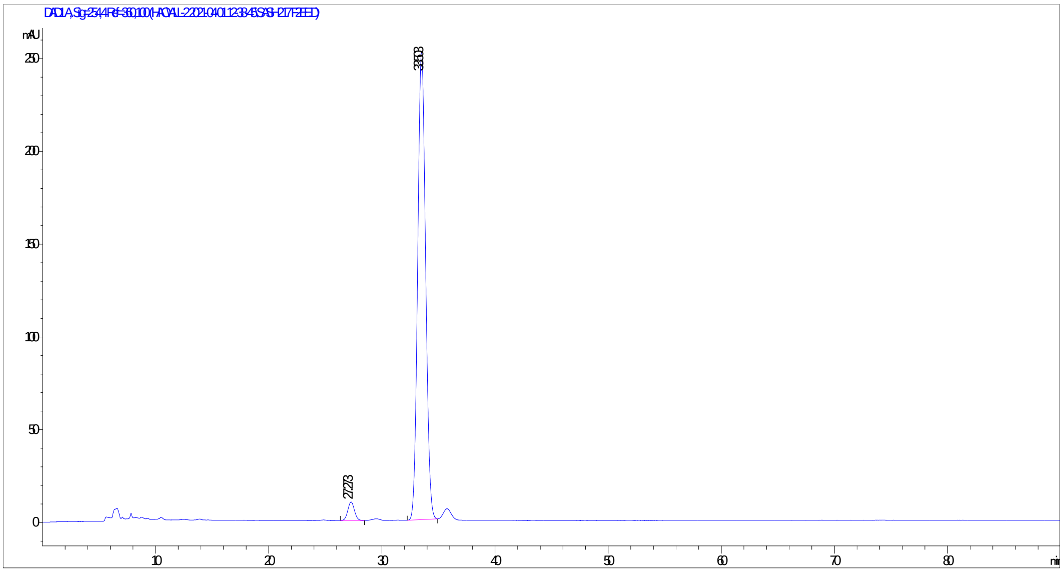


| # | Time | Area | Height | Width | Area% | Symmetry |
|---|---|---|---|---|---|---|
| 1 | 27.28 | 343.9 | 9.3 | 0.6188 | 2.693 | 0.862 |
| 2 | 33.503 | 12425.5 | 253.6 | 0.8167 | 97.307 | 0.907 |

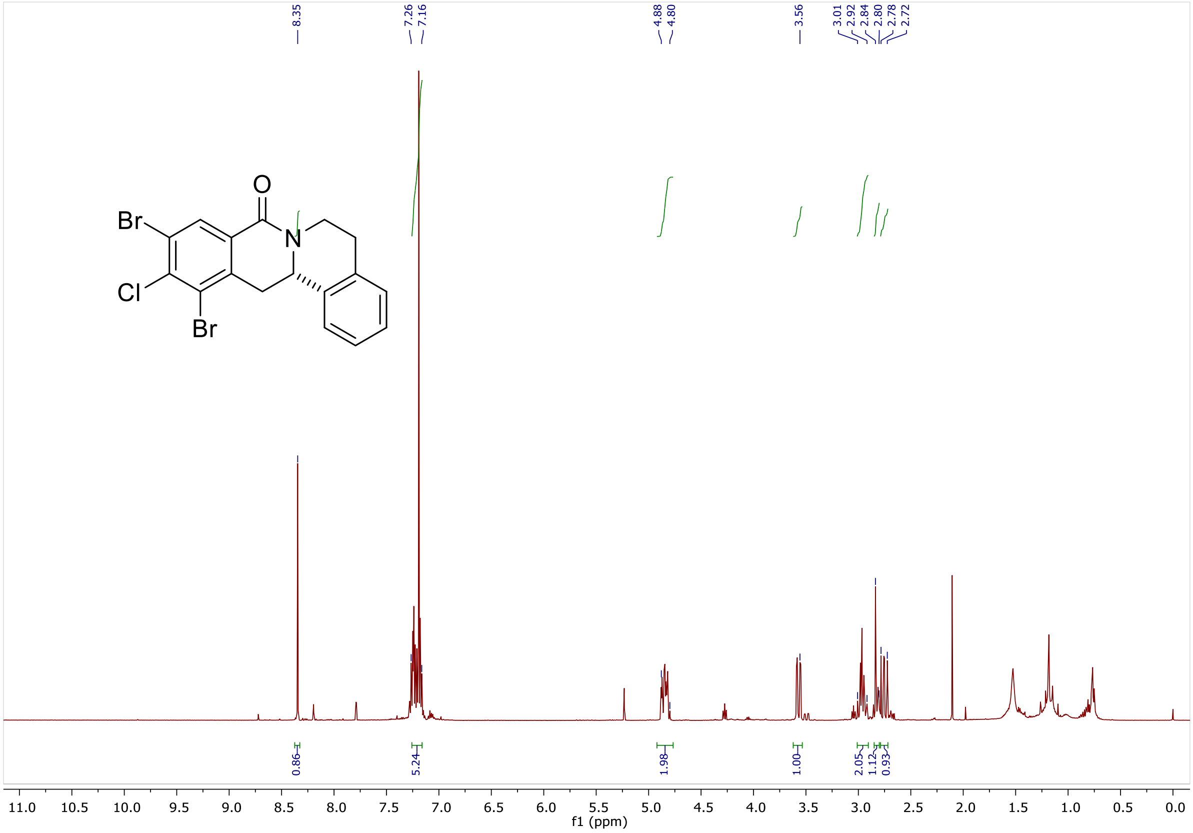

8.35
7.26
7.16
4.88
4.80
3.56
3.01
2.92
2.84
2.80
2.78
2.72
O
Br
N
Cl
Br
0.86
5.24
1.98
1.00
2.05
1.12
0.93
11.0 10.5 10.0 9.5 9.0 8.5 8.0 7.5 7.0 6.5 6.0 5.5 5.0 4.5 4.0 3.5 3.0 2.5 2.0 1.5 1.0 0.5 0.0
f1 (ppm)


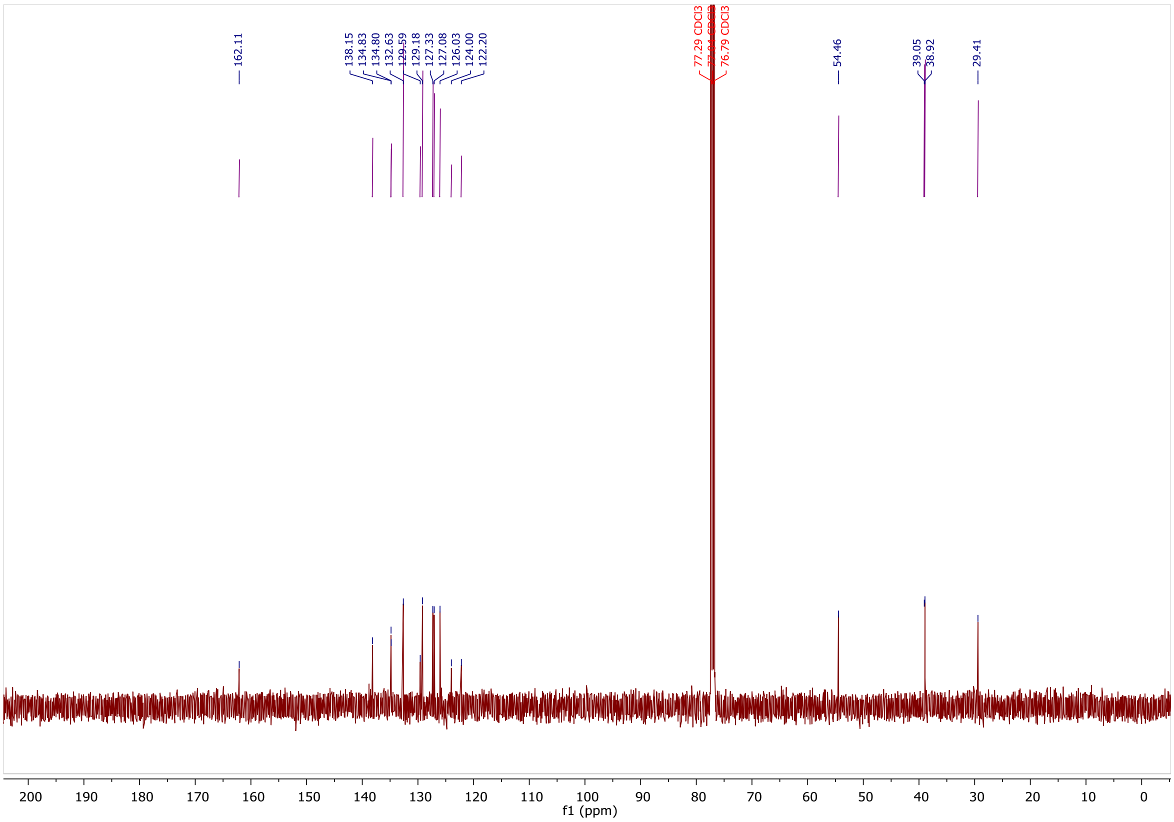

162.11
138.15
134.83
134.80
132.63
129.59
129.18
127.33
127.08
126.03
124.00
122.20
77.29 CDCl3
76.79 CDCl3
54.46
39.05
38.92
29.41
200 190 180 170 160 150 140 130 120 110 100 90 80 70 60 50 40 30 20 10 0
f1 (ppm)

HPLC traces (**3q**): racemate top, enantiomer bottom:

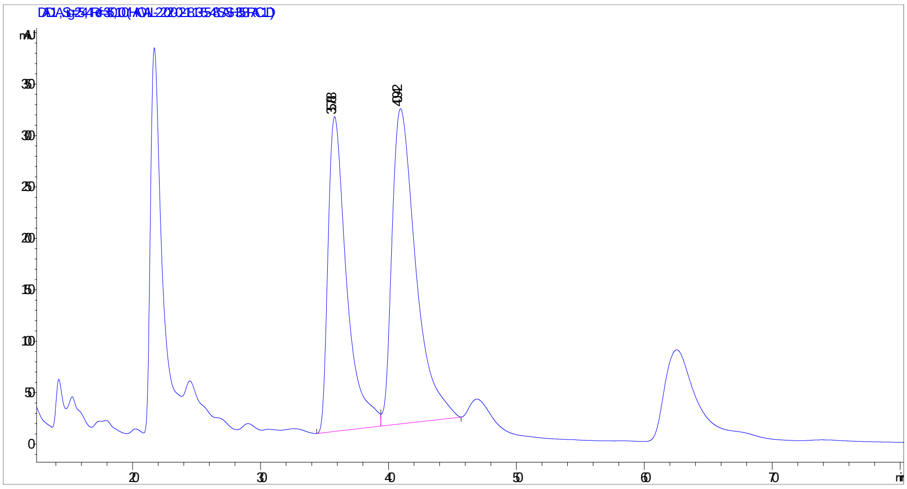

| # | Time | Area | Height | Width | Area% | Symmetry |
|---|---|---|---|---|---|---|
| 1 | 35.788 | 31091 | 306.1 | 1.5189 | 43.812 | 0.489 |
| 2 | 40.942 | 39873.1 | 306.5 | 1.9857 | 56.188 | 0.517 |

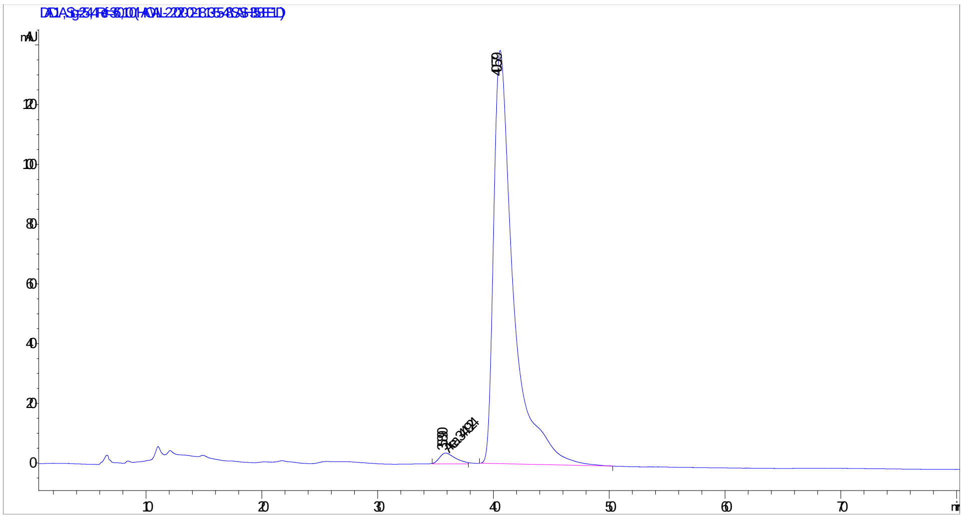

| # | Time | Area | Height | Width | Area% | Symmetry |
|---|---|---|---|---|---|---|
| 1 | 35.867 | 424.1 | 4.2 | 1.218 | 2.596 | 0.729 |
| 2 | 40.579 | 15913.5 | 138.1 | 1.921 | 97.404 | 0.491 |

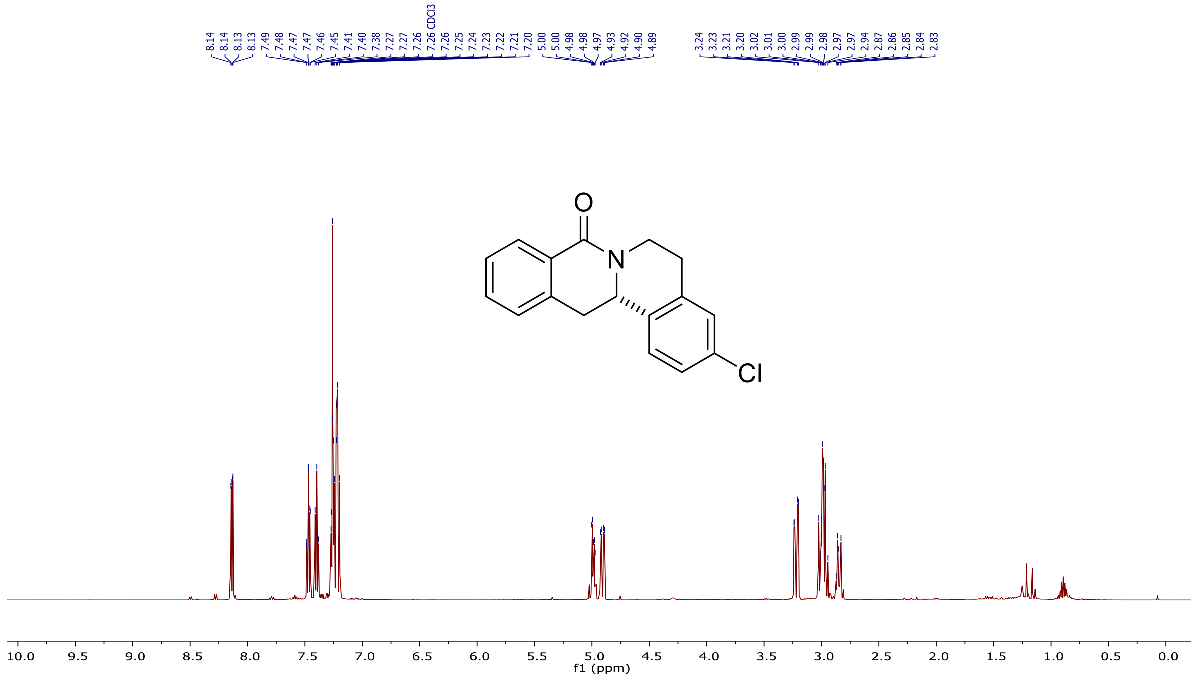

8.14
8.14
8.13
8.13
7.49
7.48
7.47
7.47
7.46
7.45
7.41
7.40
7.38
7.27
7.27
7.26 CDCl3
7.26
7.25
7.24
7.23
7.22
7.21
7.20
5.00
5.00
4.98
4.98
4.97
4.93
4.92
4.90
4.89
3.24
3.23
3.21
3.20
3.02
3.01
3.00
2.99
2.99
2.98
2.97
2.97
2.94
2.87
2.86
2.85
2.84
2.83
O
N
Cl
f1 (ppm)


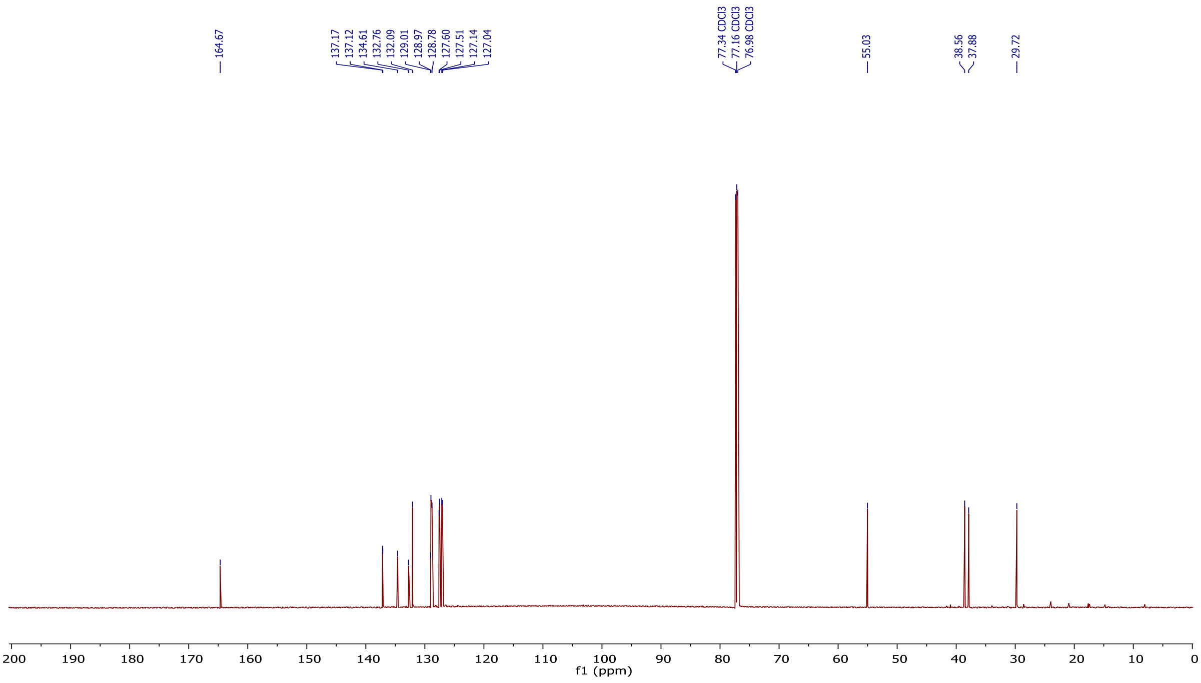

164.67
137.17
137.12
134.61
132.76
132.09
129.01
128.97
128.78
127.60
127.51
127.14
127.04
77.34 CDCl3
77.16 CDCl3
76.98 CDCl3
55.03
38.56
37.88
29.72
f1 (ppm)

HPLC traces (**3s**): racemate top, enantiomer bottom:

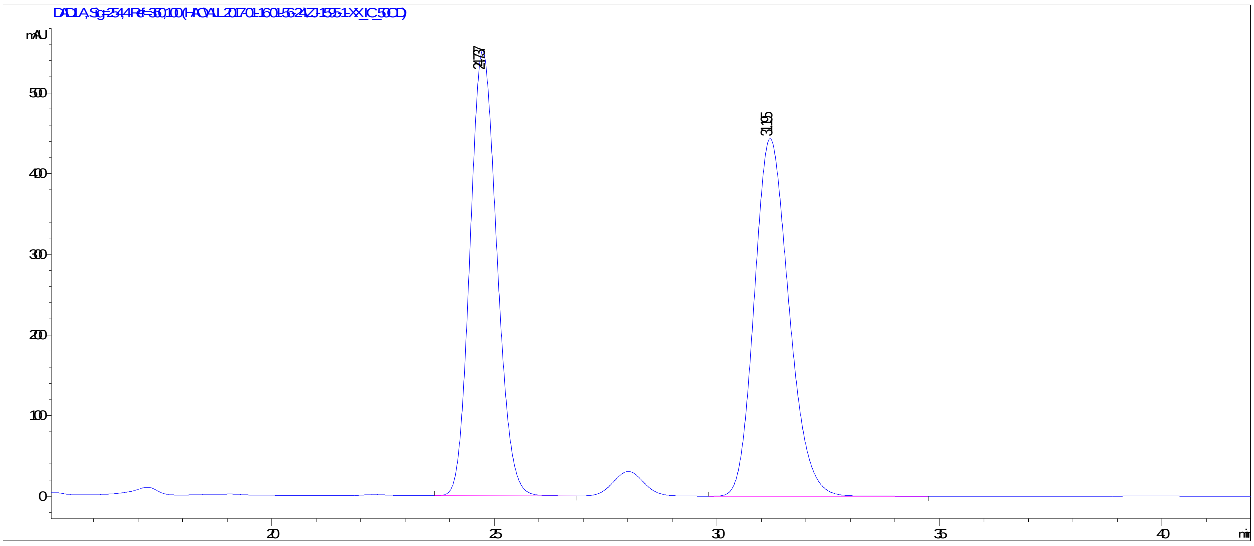


Signal 1: DAD1 A, Sig=254,4 Ref=360,100

| Peak # | RetTime [min] | Type | Width [min] | Area [mAU*s] | Height [mAU] | Area % |
|---|---|---|---|---|---|---|
| 1 | 24.737 | BB | 0.6557 | 2.30645e4 | 551.71753 | 49.0898 |
| 2 | 31.195 | BB | 0.8375 | 2.39199e4 | 443.32202 | 50.9102 |

Totals : 4.69844e4 995.03955

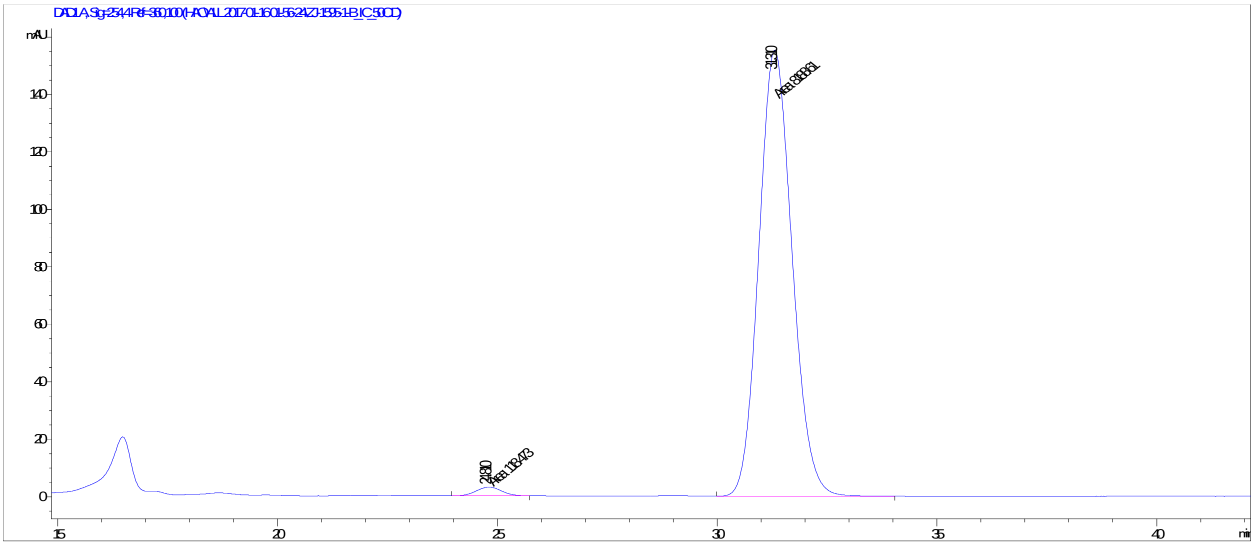


Signal 1: DAD1 A, Sig=254,4 Ref=360,100

| Peak # | RetTime [min] | Type | Width [min] | Area [mAU*s] | Height [mAU] | Area % |
|---|---|---|---|---|---|---|
| 1 | 24.810 | MM | 0.6592 | 118.47342 | 2.99554 | 1.4262 |
| 2 | 31.310 | MM | 0.8787 | 8188.61035 | 155.31525 | 98.5738 |

Totals : 8307.08377 158.31078

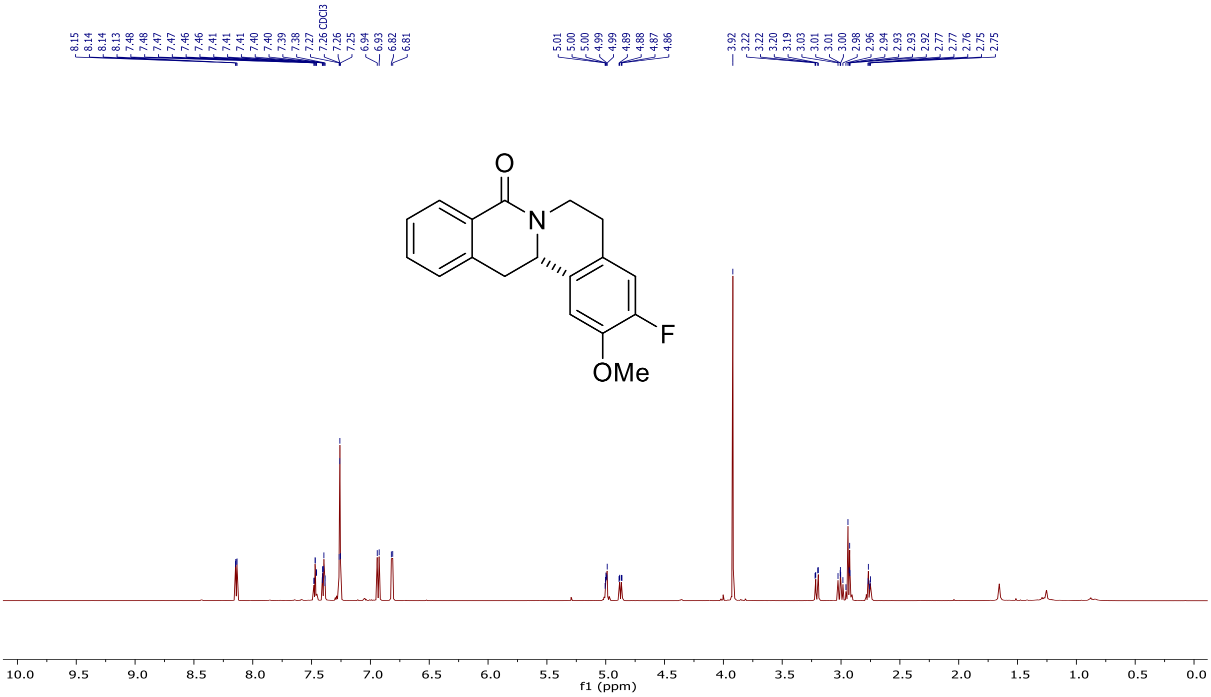
8.15
8.14
8.14
8.13
7.48
7.48
7.47
7.47
7.46
7.46
7.41
7.41
7.41
7.40
7.40
7.39
7.38
7.27
7.26 CDCl3
7.26
7.25
6.94
6.93
6.82
6.81
5.01
5.00
5.00
4.99
4.99
4.89
4.88
4.87
4.86
3.92
3.22
3.22
3.20
3.19
3.03
3.01
3.01
3.00
2.98
2.96
2.94
2.93
2.93
2.92
2.77
2.77
2.76
2.75
2.75
O
N
F
OMe
10.0
9.5
9.0
8.5
8.0
7.5
7.0
6.5
6.0
5.5
5.0
4.5
4.0
3.5
3.0
2.5
2.0
1.5
1.0
0.5
0.0
f1 (ppm)

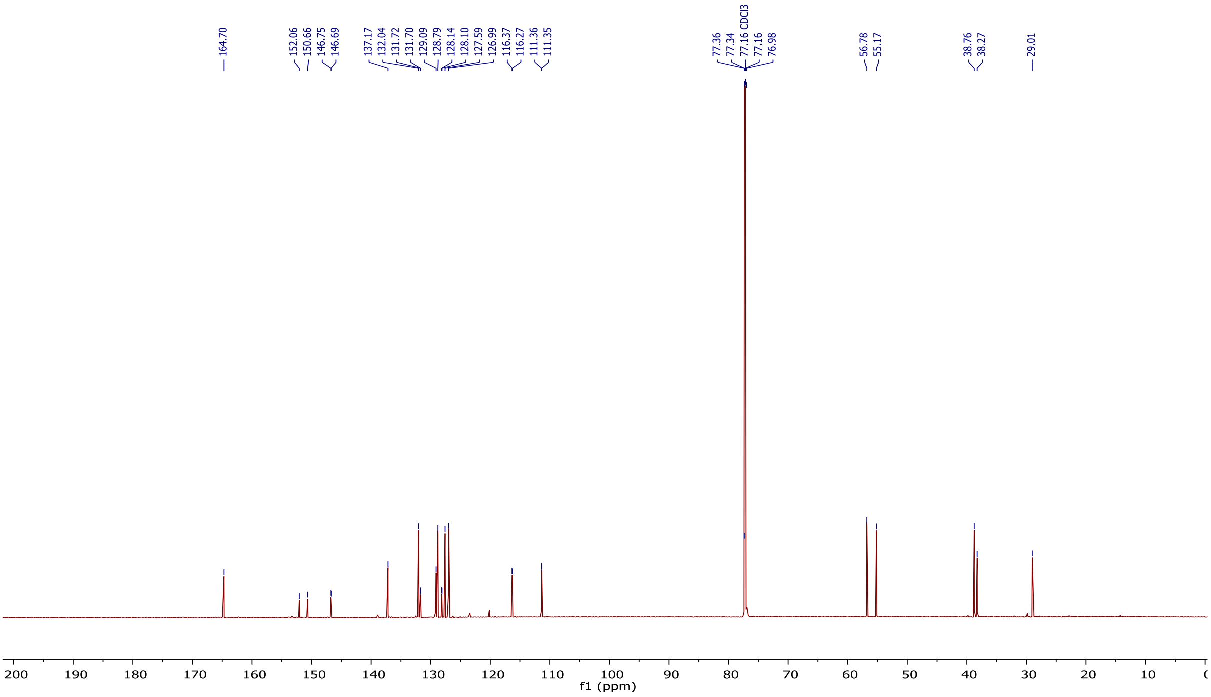
164.70
152.06
150.66
146.75
146.69
137.17
132.04
131.72
131.70
129.09
128.79
128.14
128.10
127.59
126.99
116.37
116.27
111.36
111.35
77.36
77.34
77.16 CDCl3
77.16
76.98
56.78
55.17
38.76
38.27
29.01
200
190
180
170
160
150
140
130
120
110
100
90
80
70
60
50
40
30
20
10
f1 (ppm)

HPLC traces (**3r**): racemate top, enantiomer bottom:

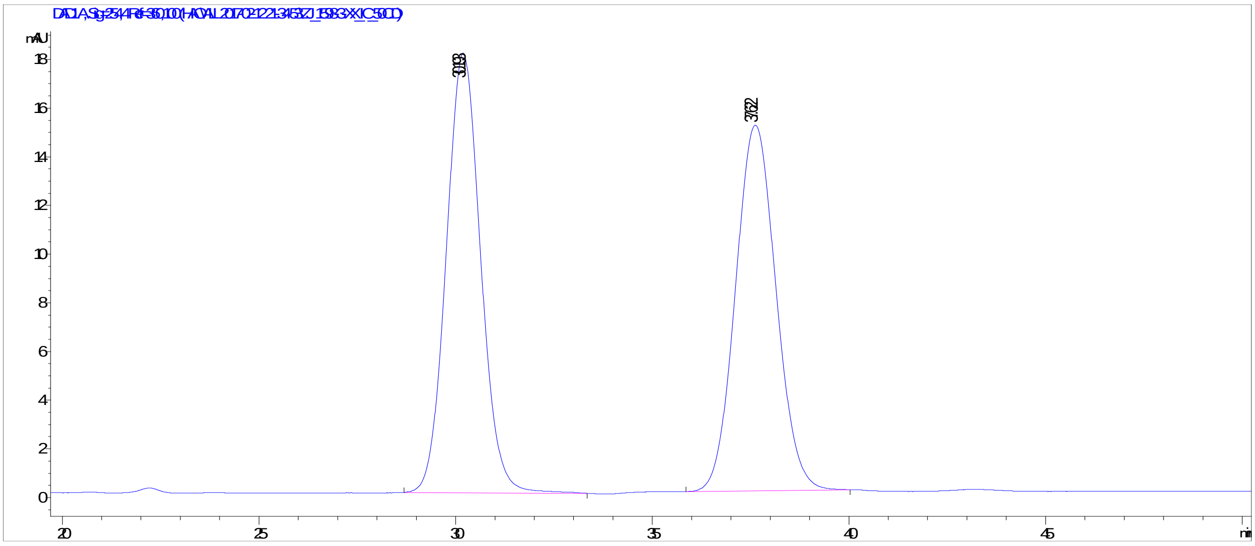

Signal 1: DAD1 A, Sig=254,4 Ref=360,100

| Peak # | RetTime [min] | Type | Width [min] | Area [mAU*s] | Height [mAU] | Area % |
|---|---|---|---|---|---|---|
| 1 | 30.193 | BB | 0.9401 | 1096.48987 | 18.06606 | 50.4640 |
| 2 | 37.622 | BB | 1.1062 | 1076.32764 | 15.01708 | 49.5360 |
| Totals : | | | | 2172.81750 | 33.08315 | |

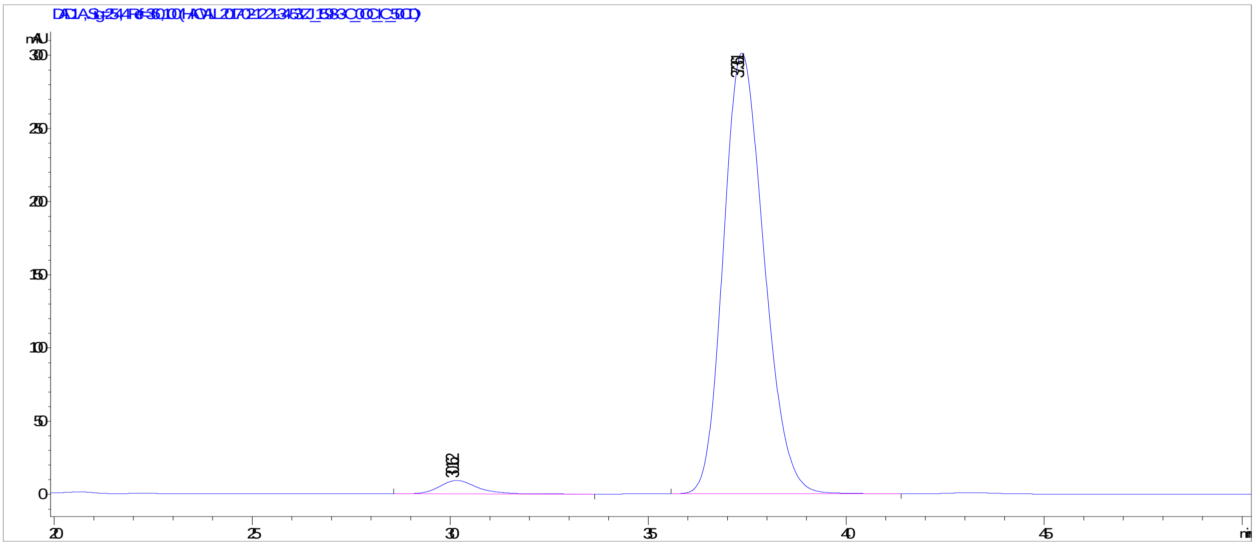

Signal 1: DAD1 A, Sig=254,4 Ref=360,100

| Peak # | RetTime [min] | Type | Width [min] | Area [mAU*s] | Height [mAU] | Area % |
|---|---|---|---|---|---|---|
| 1 | 30.162 | BB | 1.0102 | 630.13043 | 9.23384 | 2.8512 |
| 2 | 37.361 | BB | 1.1182 | 2.14706e4 | 300.98801 | 97.1488 |
| Totals : | | | | 2.21007e4 | 310.22184 | |

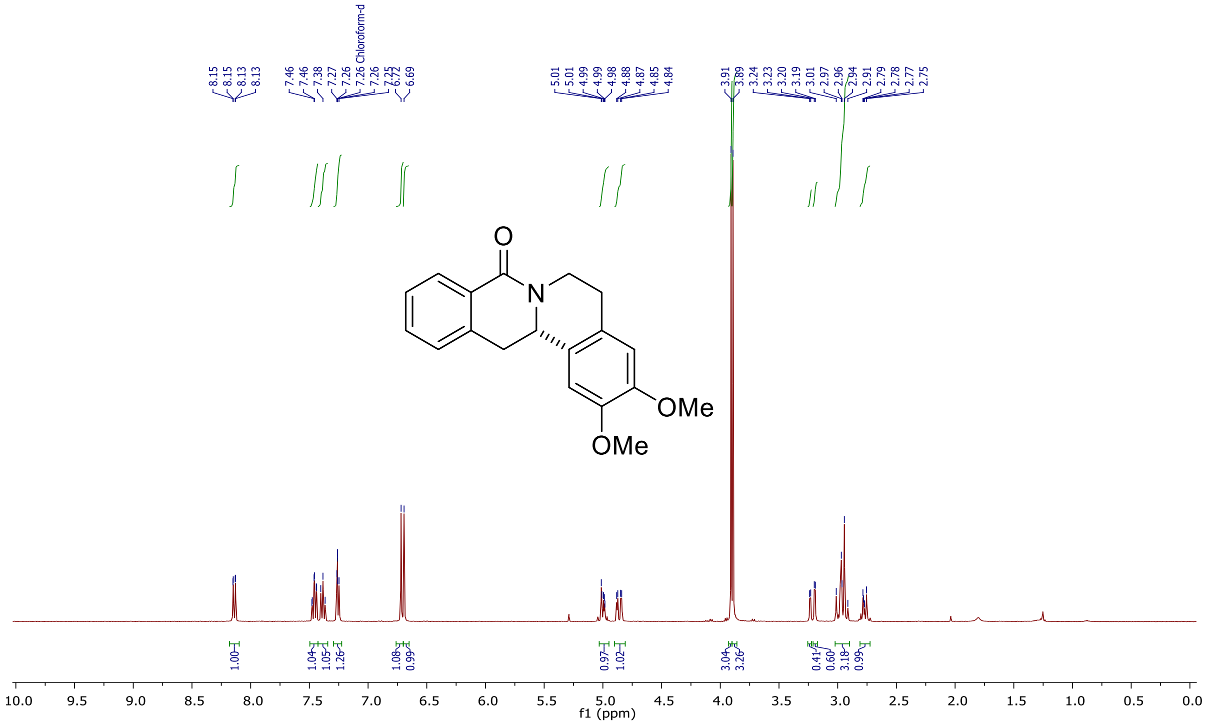
8.15
8.15
8.13
8.13
7.46
7.46
7.38
7.27
7.26
7.26 Chloroform-d
7.26
6.75
6.72
6.69
5.01
5.01
4.99
4.99
4.98
4.88
4.87
4.85
4.84
3.91
3.89
3.24
3.23
3.20
3.19
3.01
2.97
2.96
2.94
2.91
2.79
2.78
2.77
2.75
O
N
OMe
OMe
1.00
1.04
1.05
1.26
1.08
0.99
0.97
1.02
3.04
3.26
0.41
0.60
3.18
0.99
f1 (ppm)

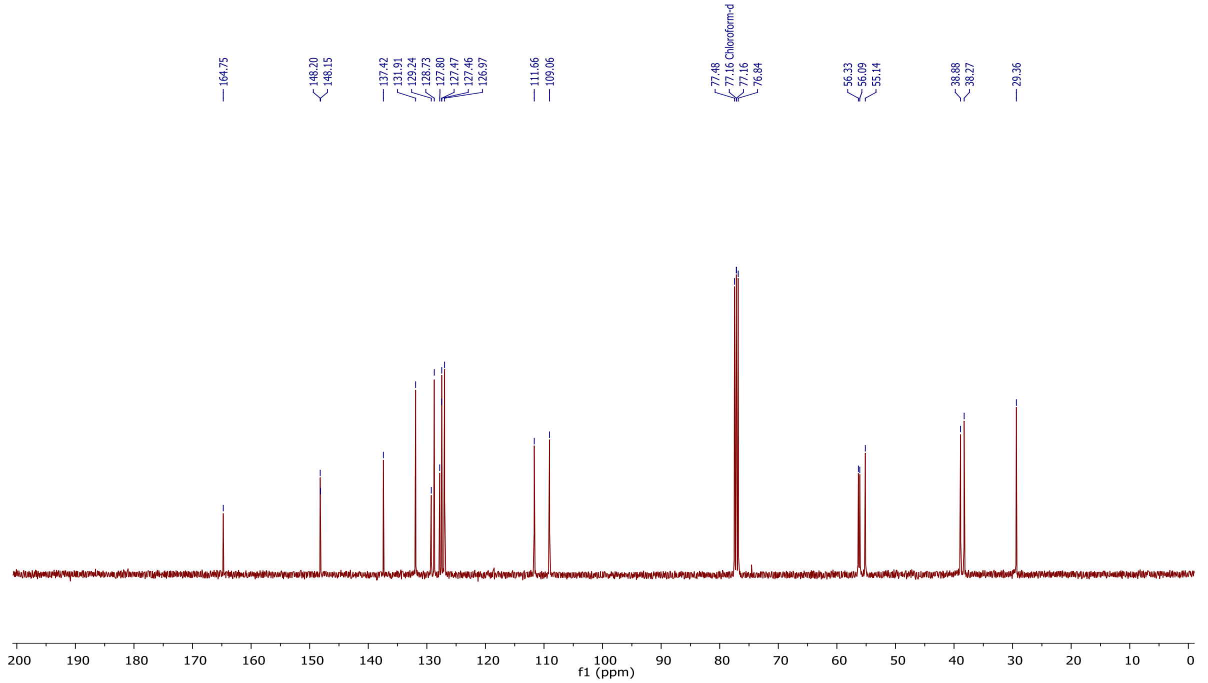
164.75
148.20
148.15
137.42
131.91
129.24
128.73
127.80
127.47
127.46
126.97
111.66
109.06
77.48
77.16 Chloroform-d
77.16
76.84
56.33
56.09
55.14
38.88
38.27
29.36
f1 (ppm)

HPLC traces (**3t**): racemate top, enantiomer bottom:

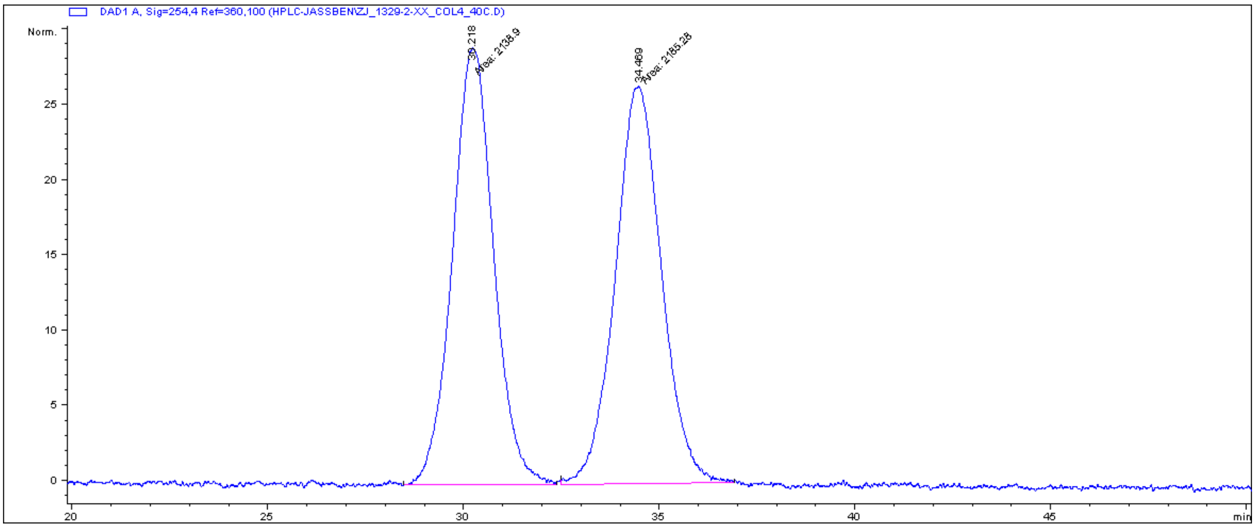


| Peak # | RetTime [min] | Type | Width [min] | Area [mAU*s] | Height [mAU] | Area % |
|---|---|---|---|---|---|---|
| 1 | 30.218 | MM | 1.2256 | 2138.90479 | 29.08594 | 49.4638 |
| 2 | 34.469 | MM | 1.3782 | 2185.27588 | 26.42747 | 50.5362 |
| Totals : | | | | 4324.18066 | 55.51341 | |

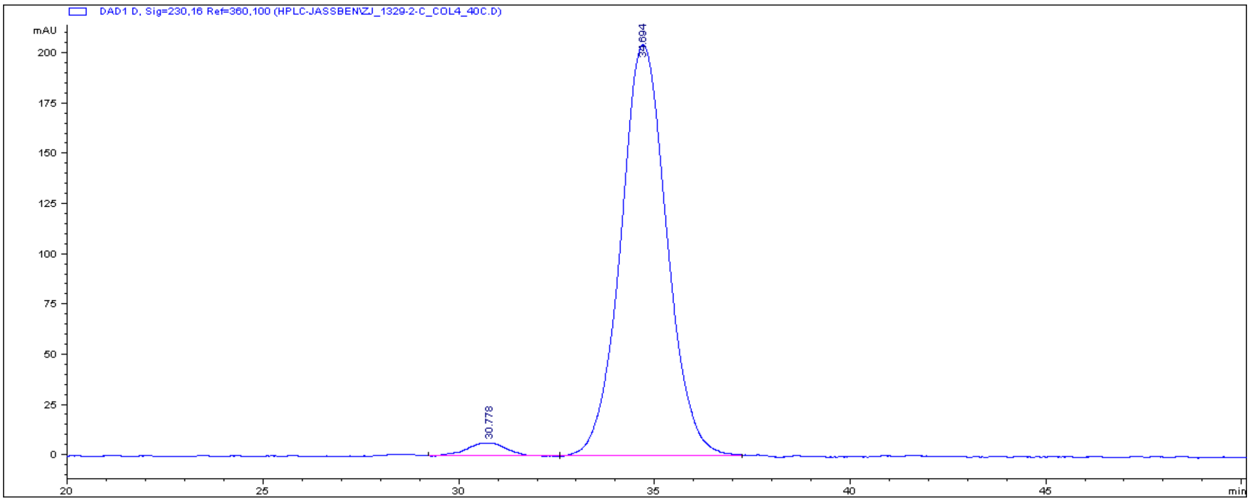


| Peak # | RetTime [min] | Type | Width [min] | Area [mAU*s] | Height [mAU] | Area % |
|---|---|---|---|---|---|---|
| 1 | 30.778 | BV | 0.9878 | 466.19125 | 6.38499 | 2.6882 |
| 2 | 34.694 | VB | 1.2414 | 1.68759e4 | 204.79214 | 97.3118 |
| Totals : | | | | 1.73421e4 | 211.17713 | |

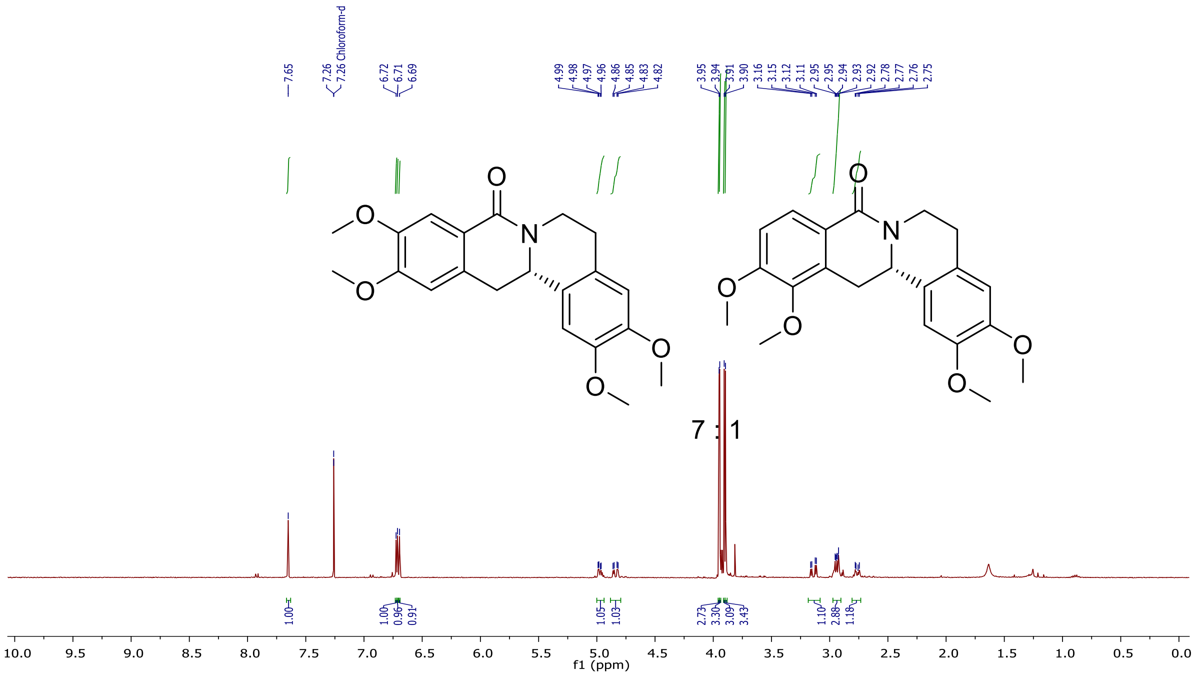

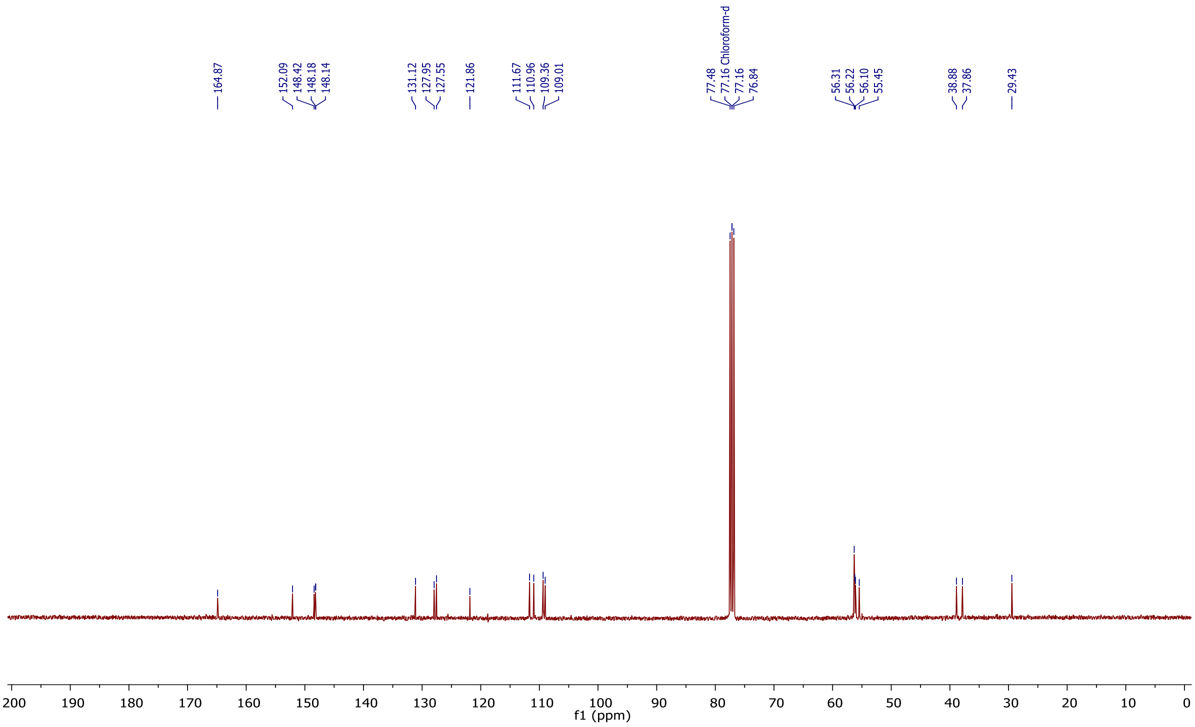

HPLC traces (**3u**): racemate top, enantiomer bottom:

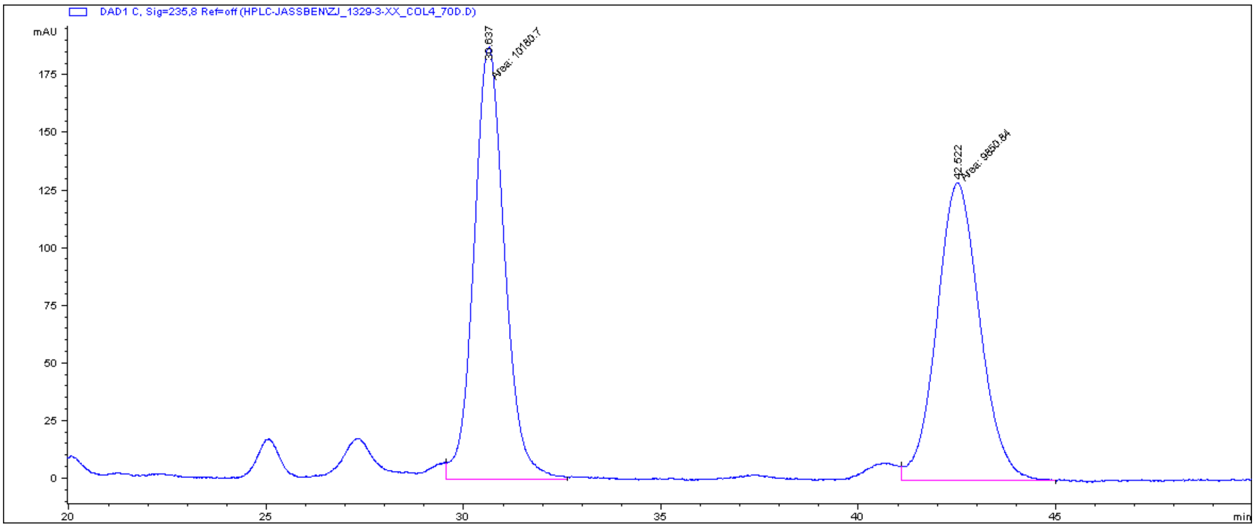


| Peak # | RetTime [min] | Type | Width [min] | Area [mAU*s] | Height [mAU] | Area % |
|---|---|---|---|---|---|---|
| 1 | 30.637 | MM | 0.8923 | 1.00228e4 | 187.21204 | 50.5306 |
| 2 | 42.522 | MM | 1.2689 | 9812.28418 | 128.88506 | 49.4694 |

Totals : 1.98350e4 316.09709

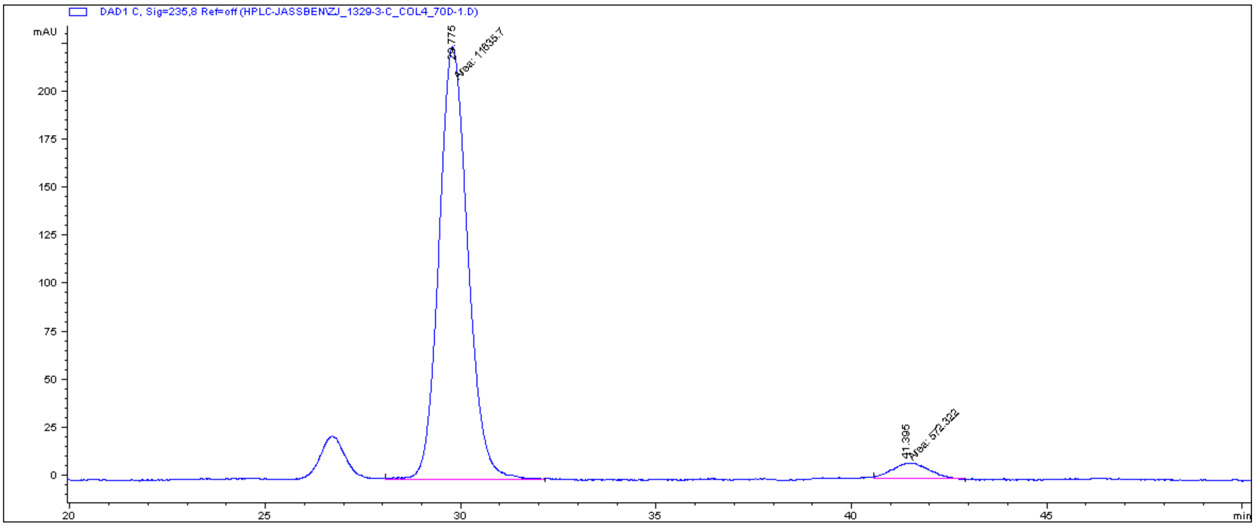


| Peak # | RetTime [min] | Type | Width [min] | Area [mAU*s] | Height [mAU] | Area % |
|---|---|---|---|---|---|---|
| 1 | 29.775 | MM | 0.8583 | 1.16357e4 | 225.93462 | 95.3119 |
| 2 | 41.395 | MM | 1.1320 | 572.32166 | 8.42605 | 4.6881 |

Totals : 1.22080e4 234.36067

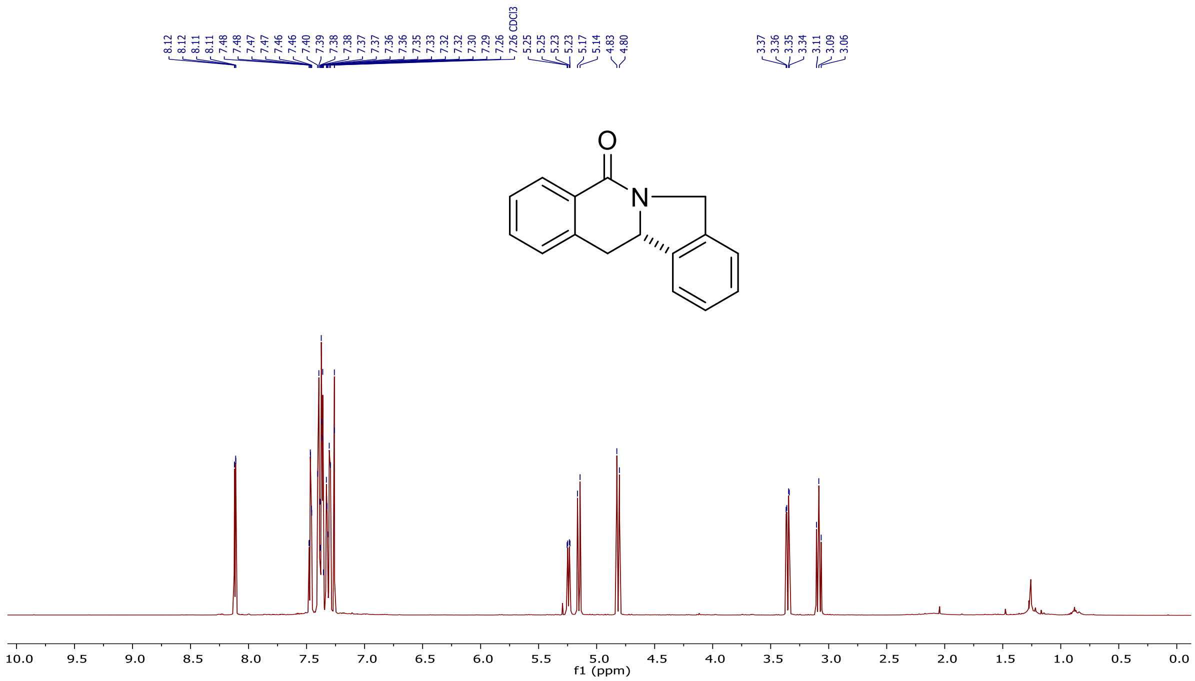
8.12
8.12
8.11
8.11
7.48
7.47
7.47
7.46
7.46
7.40
7.39
7.38
7.38
7.37
7.37
7.36
7.36
7.35
7.33
7.32
7.32
7.30
7.29
7.26
7.26 CDCl3
5.25
5.25
5.23
5.23
5.17
5.14
4.83
4.80
3.37
3.36
3.35
3.34
3.11
3.09
3.06
O
N
10.0 9.5 9.0 8.5 8.0 7.5 7.0 6.5 6.0 5.5 5.0 4.5 4.0 3.5 3.0 2.5 2.0 1.5 1.0 0.5 0.0
f1 (ppm)

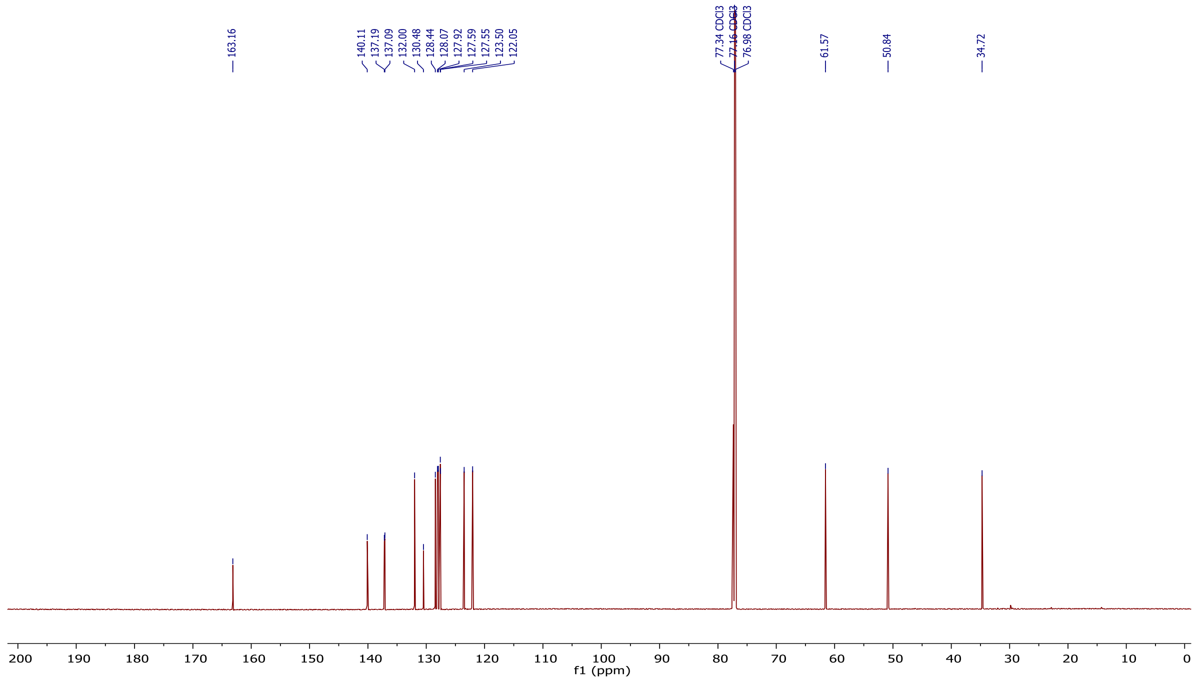
163.16
140.11
137.19
137.09
132.00
130.48
128.44
128.07
127.92
127.59
127.55
123.50
122.05
77.34 CDCl3
77.16 CDCl3
76.98 CDCl3
61.57
50.84
34.72
200 190 180 170 160 150 140 130 120 110 100 90 80 70 60 50 40 30 20 10 0
f1 (ppm)

HPLC traces (**4**): racemate top, enantiomer bottom:

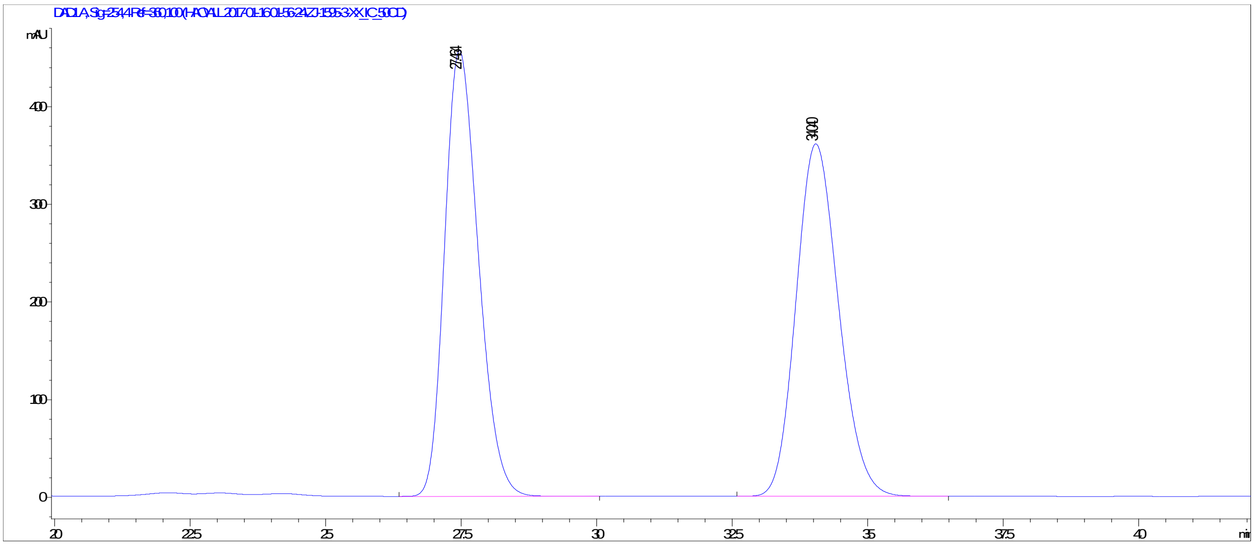

Signal 1: DAD1 A, Sig=254,4 Ref=360,100

| Peak # | RetTime [min] | Type | Width [min] | Area [mAU*s] | Height [mAU] | Area % |
|---|---|---|---|---|---|---|
| 1 | 27.464 | BB | 0.6730 | 1.97615e4 | 456.52097 | 50.1123 |
| 2 | 34.040 | BB | 0.8503 | 1.96729e4 | 360.72922 | 49.8877 |
| Totals : | | | | 3.94345e4 | 817.25018 | |

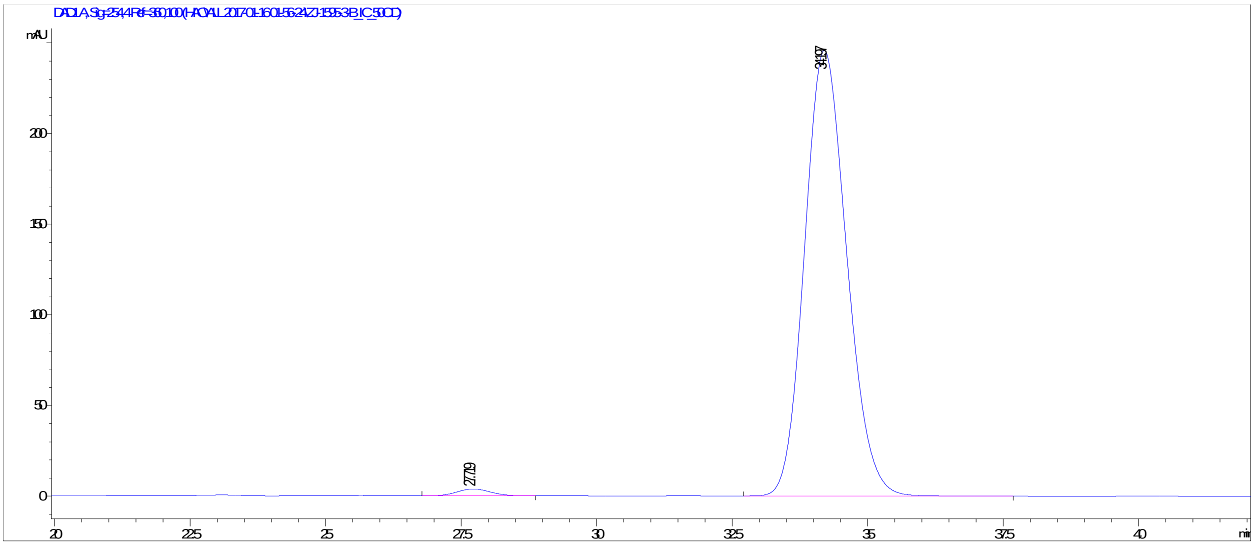

Signal 1: DAD1 A, Sig=254,4 Ref=360,100

| Peak # | RetTime [min] | Type | Width [min] | Area [mAU*s] | Height [mAU] | Area % |
|---|---|---|---|---|---|---|
| 1 | 27.719 | BB | 0.6560 | 160.59401 | 3.76191 | 1.1940 |
| 2 | 34.197 | BB | 0.8407 | 1.32890e4 | 245.83856 | 98.8060 |
| Totals : | | | | 1.34496e4 | 249.60047 | |

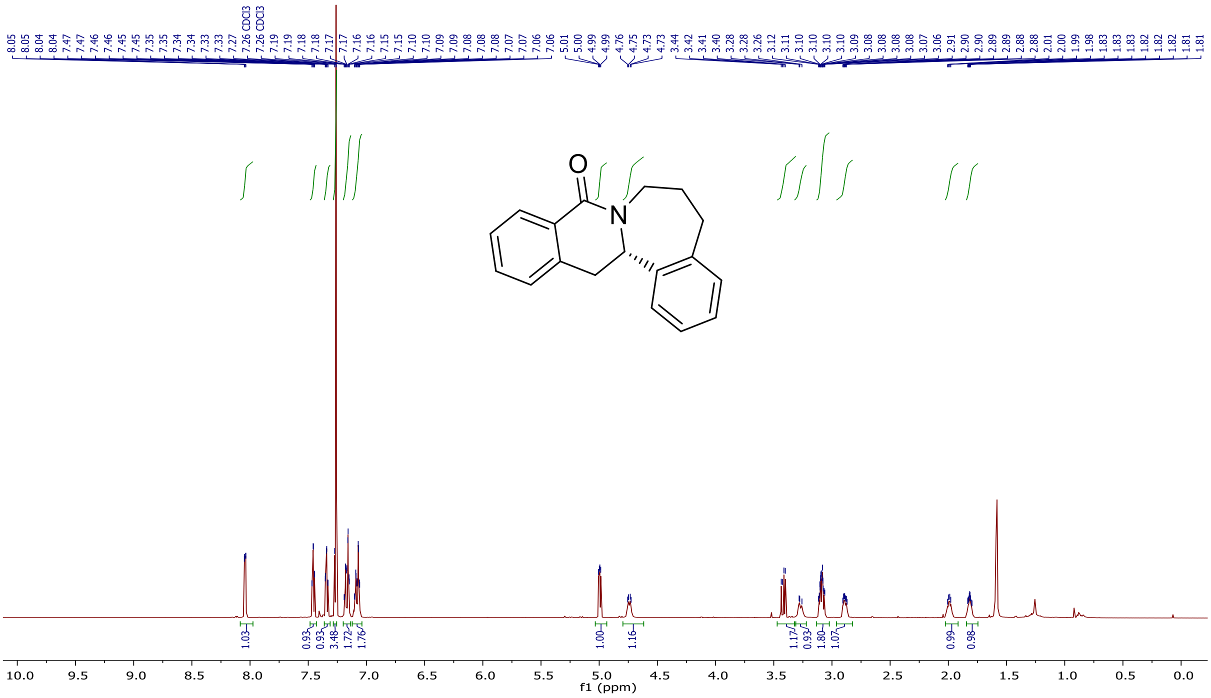

8.05
8.05
8.04
8.04
7.47
7.46
7.46
7.45
7.45
7.35
7.35
7.34
7.34
7.33
7.33
7.27 CDCl3
7.26 CDCl3
7.19
7.19
7.18
7.18
7.17
7.17
7.16
7.16
7.15
7.15
7.10
7.10
7.09
7.09
7.08
7.08
7.08
7.07
7.07
7.06
7.06
5.01
5.00
4.99
4.99
4.76
4.75
4.73
4.73
3.44
3.42
3.41
3.40
3.28
3.28
3.26
3.12
3.11
3.10
3.10
3.10
3.10
3.09
3.08
3.08
3.08
3.08
3.07
3.06
2.91
2.90
2.90
2.89
2.89
2.88
2.88
2.01
2.00
1.99
1.98
1.83
1.83
1.83
1.82
1.82
1.81
1.81
O
N
1.03
0.93
0.93
3.48
1.72
1.76
1.00
1.16
1.17
0.93
1.80
1.07
0.99
0.98
10.0
9.5
9.0
8.5
8.0
7.5
7.0
6.5
6.0
5.5
5.0
4.5
4.0
3.5
3.0
2.5
2.0
1.5
1.0
0.5
0.0
f1 (ppm)


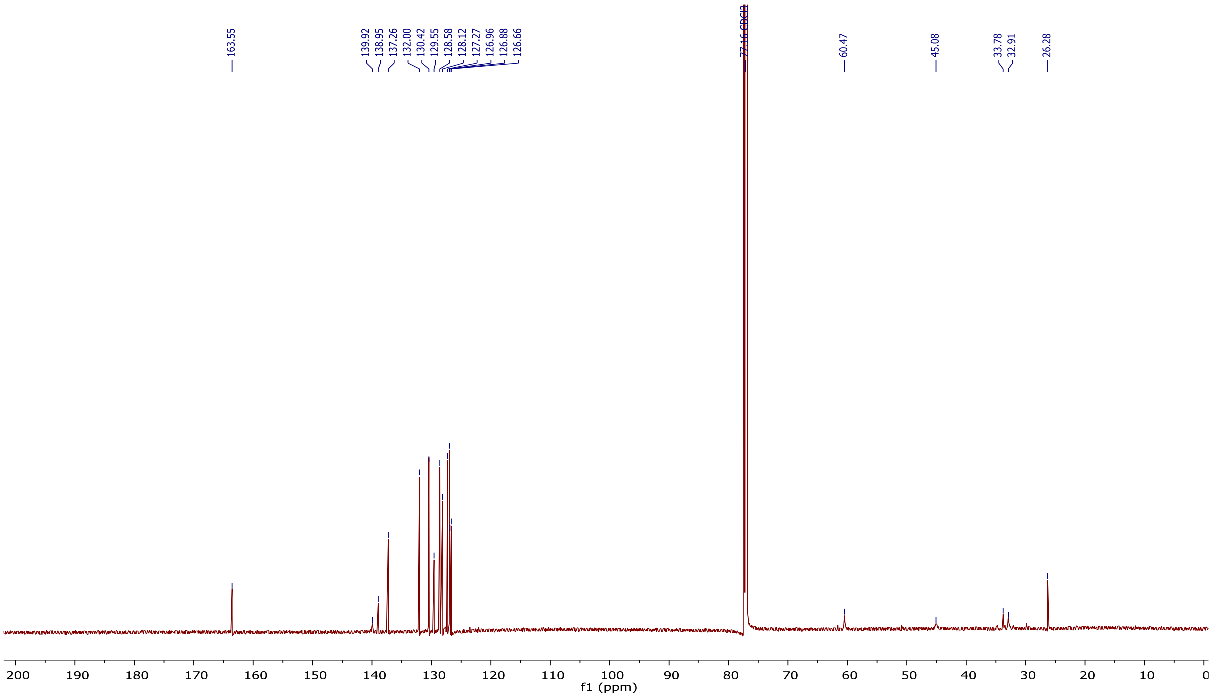

163.55
139.92
138.95
137.26
132.00
130.42
129.55
128.58
128.12
127.27
126.96
126.88
126.66
77.16 CDCl3
60.47
45.08
33.78
32.91
26.28
200
190
180
170
160
150
140
130
120
110
100
90
80
70
60
50
40
30
20
10
0
f1 (ppm)

HPLC traces (**5**): racemate top, enantiomer bottom:

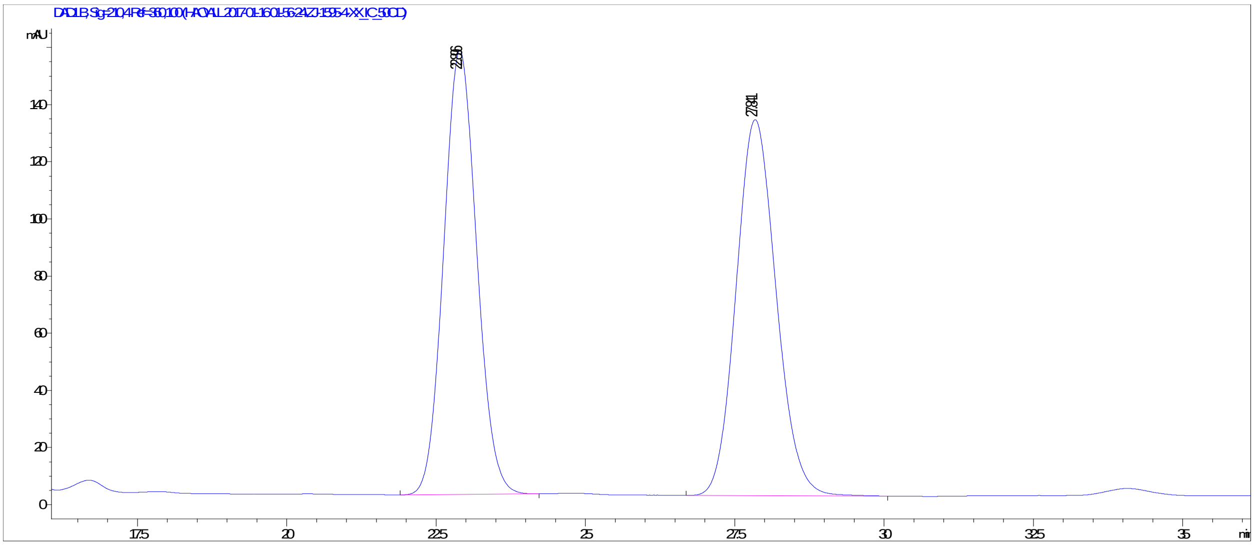

Signal 2: DAD1 B, Sig=210,4 Ref=360,100

| Peak # | RetTime [min] | Type | Width [min] | Area [mAU*s] | Height [mAU] | Area % |
|---|---|---|---|---|---|---|
| 1 | 22.896 | BB | 0.5989 | 5980.50732 | 155.42070 | 49.3448 |
| 2 | 27.841 | BB | 0.7263 | 6139.33350 | 131.60069 | 50.6552 |

Totals : 1.21198e4 287.02139

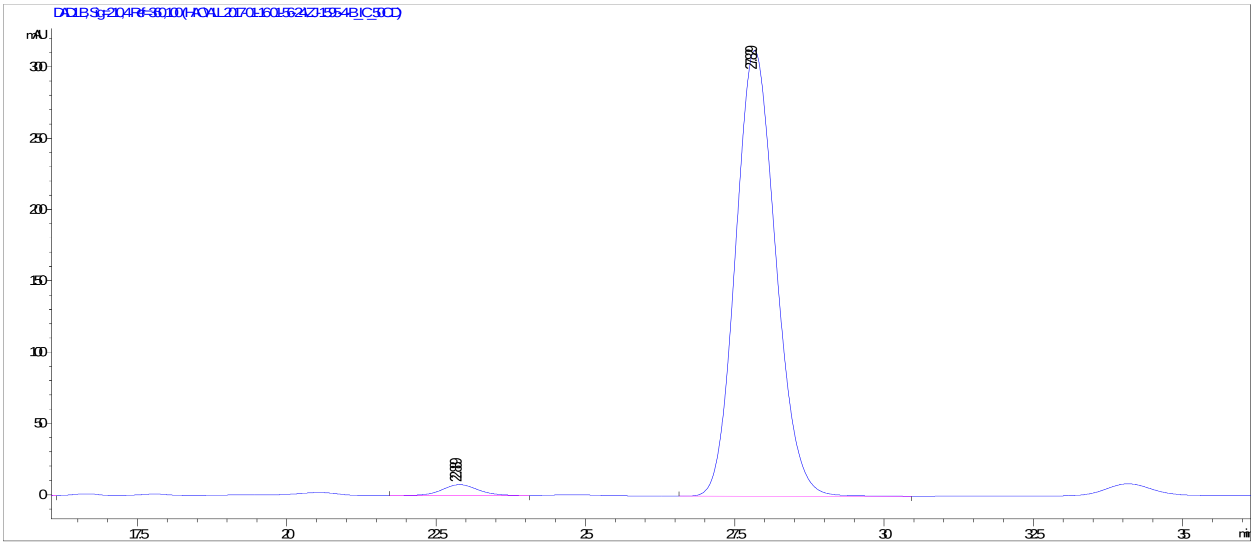

Signal 2: DAD1 B, Sig=210,4 Ref=360,100

| Peak # | RetTime [min] | Type | Width [min] | Area [mAU*s] | Height [mAU] | Area % |
|---|---|---|---|---|---|---|
| 1 | 22.889 | BB | 0.6341 | 330.54788 | 7.71068 | 2.2126 |
| 2 | 27.829 | BB | 0.7230 | 1.46091e4 | 312.75531 | 97.7874 |

Totals : 1.49396e4 320.46599
